# New 2-[(4-Amino-6-*N*-substituted-1,3,5-triazin-2-yl)methylthio]-*N*-(imidazolidin-2-ylidene)-4-chloro-5-methylbenzenesulfonamide Derivatives, Design, Synthesis and Anticancer Evaluation

**DOI:** 10.3390/ijms23137178

**Published:** 2022-06-28

**Authors:** Łukasz Tomorowicz, Beata Żołnowska, Krzysztof Szafrański, Jarosław Chojnacki, Ryszard Konopiński, Ewa A. Grzybowska, Jarosław Sławiński, Anna Kawiak

**Affiliations:** 1 Department of Organic Chemistry, Faculty of Pharmacy, Medical University of Gdańsk, Al. Gen. J. Hallera 107, 80-416 Gdańsk, Poland; ltomorowicz@gumed.edu.pl (Ł.T.); zolnowska@gumed.edu.pl (B.Ż.); k.szafranski@gumed.edu.pl (K.S.); 2 Department of Inorganic Chemistry, Faculty of Chemistry, Gdańsk University of Technology, G. Narutowicza 11/12, 80-233 Gdańsk, Poland; jaroslaw.chojnacki@pg.edu.pl; 3 Maria Skłodowska-Curie National Research Institute of Oncology, ul. Roentgena 5, 02-781 Warszawa, Poland; ryszard.konopinski@pib-nio.pl (R.K.); ewa.grzybowska@pib-nio.pl (E.A.G.); 4 Department of Biotechnology, Intercollegiate Faculty of Biotechnology, University of Gdańsk and Medical University of Gdańsk, ul. Abrahama 58, 80-307 Gdańsk, Poland

**Keywords:** benzenesulfonamide, synthesis, 1,3,5-triazines, imidazole, QSAR, anticancer activity, cell cycle arrest, proliferation, apoptosis

## Abstract

In the search for new compounds with antitumor activity, new potential anticancer agents were designed as molecular hybrids containing the structures of a triazine ring and a sulfonamide fragment. Applying the synthesis in solution, a base of new sulfonamide derivatives **20**–**162** was obtained by the reaction of the corresponding esters **11**–**19** with appropriate biguanide hydrochlorides. The structures of the compounds were confirmed by spectroscopy (IR, NMR), mass spectrometry (HRMS or MALDI-TOF/TOF), elemental analysis (C,H,N) and X-ray crystallography. The cytotoxic activity of the obtained compounds toward three tumor cell lines, HCT-116, MCF-7 and HeLa, was examined. The results showed that some of the most active compounds belonged to the R^1^ = 4-trifluoromethylbenzyl and R^1^ = 3,5-bis(trifluoromethyl)benzyl series and exhibited IC_50_ values ranging from 3.6 µM to 11.0 µM. The SAR relationships were described, indicating the key role of the R^2^ = 4-phenylpiperazin-1-yl substituent for the cytotoxic activity against the HCT-116 and MCF-7 lines. The studies regarding the mechanism of action of the active compounds included the assessment of the inhibition of MDM2-p53 interactions, cell cycle analysis and apoptosis induction examination. The results indicated that the studied compounds did not inhibit MDM2-p53 interactions but induced G0/G1 and G2/M cell cycle arrest in a p53-independent manner. Furthermore, the active compounds induced apoptosis in cells harboring wild-type and mutant p53. The compound design was conducted step by step and assisted by QSAR models that correlated the activity of the compounds against the HCT-116 cell line with molecular descriptors.

## 1. Introduction

Cancers are the leading cause of death worldwide. The WHO reports that, in 2020, the number of deaths from cancer was almost 10 million. The most common cancers in terms of new cases in 2020 were: breast cancer (2.26 million cases), lung cancer (2.21 million cases), colon and rectal cancer (1.93 million cases), prostate cancer (1.41 million cases), skin cancer (non-melanoma) (1.20 million cases) and stomach cancer (1.09 million cases). The most common causes of cancer deaths in 2020 were lung cancer (1.80 million deaths), colon and rectal cancer (935,000 deaths), liver cancer (830,000 deaths), stomach cancer (769,000 deaths), and breast cancer (685,000 deaths) [1]. The most common cancers are colorectal cancers, including colon and breast cancer. This fact has led us to focus our research, among others, on the cancer cell lines of breast cancer (MCF-7) and colon cancer (HCT-116).

Chemotherapy, radiotherapy and surgery are the most popular methods of cancer treatment available nowadays [2]. Multi-drug resistance (MDR) is one of the main problems of long-term chemotherapy failure [3,4]. For some types of cancer, e.g., pancreatic cancer, no effective chemotherapeutic agents have been found so far, and the only method of choice with a minimum survival of 5 years is surgical resection, which is less than 5% effective [5]. On the other hand, in the case of surgically inoperable cancers (which include the majority of infiltrating cancers and those located in places inaccessible for surgical treatment), chemotherapy [6,7], hormone therapy [7], immunotherapy [8] and radiotherapy [7] are the main treatment options.

**Figure 1:**
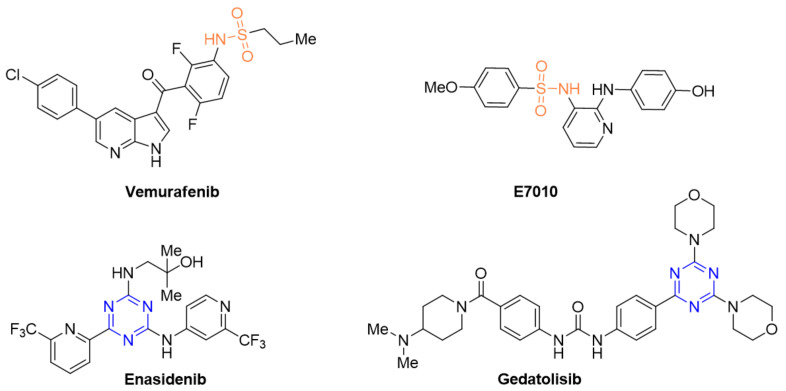
Structures of exemplary anticancer drugs and compounds containing a sulfonamide or 1,3,5-triazine moiety which are in clinical trials, among others, for breast or colorectal cancer.

Undoubtedly, compounds containing a sulfonamide moiety belong to a wide group of chemotherapeutic agents that have been used as anticancer agents. One good example can be Vemurafenib, which has been used since 2011 in the chemotherapy of melanomas with the BRAF V600 mutation [9]. Vemurafenib is currently in phase two clinical trials for colorectal cancer patients with the BRAF mutation in the VIC regimen [10] and for non-small cell lung cancer [11]. Another sulfonamide compound, E7010 (Figure 1), is presently in phase two clinical trials in colorectal cancer patients [12]. In turn, Enasidenib, approved by the FDA in 2017 [13], containing the 1,3,5-triazine moiety, is used in relapsed or refractory acute myeloid leukemia (AML), and Gedatolisib (Figure 1) is used in combination either with Talazoparib, at present in phase two clinical trials for breast cancer [14], or Palbociclib, in phase one clinical trials for patients with head and neck, pancreatic, lung and solid tumors [15].

**Figure 2:**
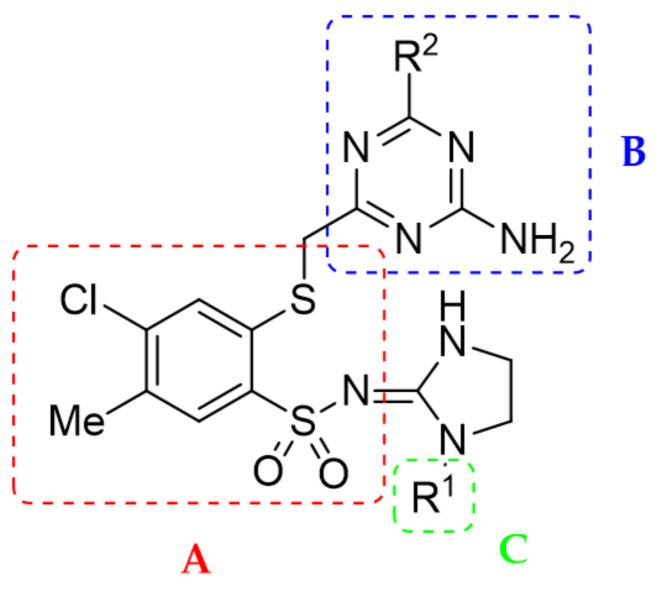
The general structure of a designed molecular hybrid consisting of two pharmacophores: a 2-mercaptobenzenesulfonamide fragment (**A**) and a 4-amino-6-R^2^-1,3,5-triazin-2-yl ring (**B**), modified by the R^1^ substituent (**C**) on the *N*-1 imidazolidine ring.

Our previous reports indicated that 4-chloro-2-arylmethylthio-5-methylbenzenesulfonamide derivatives show cytotoxic activity against numerous human cancer cell lines, including colon, breast and cervical cancer, among others [16,17,18,19]. We have also proved that some of these compounds showed an apoptotic effect in cancer cells. In this work, we developed a series of 2-[(4-amino-6-R^2^-1,3,5-triazin-2-yl)methylthio]-*N*-(1-R^1^-imidazolidin-2-ylidene)-4-chloro-5-methylbenzenesulfonamides (Figure 2), applying the molecular hybrids strategy. Molecular hybrids were designed as a combination of 2-mercaptobenzenesulfonamide fragments (A) with a 4-amino-6-R^2^-1,3,5-triazin-2-yl ring (B). The structures of the designed compounds were also diversified with the substituent R^1^ (C) (Figure 2) on the imidazolidine ring in position *N*-1 to investigate their influence on cytotoxic activity.

**Figure 3:**
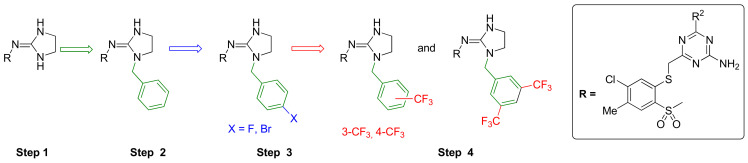
A pathway of the designing of 2-[(4-amino-6-R^2^-1,3,5-triazin-2-yl)methylthio]-*N*-(imidazolidin-2-ylidene)-4-chloro-5-methylbenzenesulfonamide derivatives based on QSAR models.

The quantitative structure–activity relationship (QSAR) computational method is helpful in the design and development of new anticancer active compounds [20,21,22]. Thus, a series of 2-[(4-amino-6-R^2^-1,3,5-triazin-2-yl)methylthio]-*N*-(1-R^1^-imidazolidin-2-ylidene)-4-chloro-5-methylbenzenesulfonamide derivatives (**20**–**157**) were synthesized in four separate steps, which required the building of QSAR models and introducing structural modifications, leading to better cytotoxic activities (Figure 3). All synthesized compounds were tested in a series designed sequentially for their cytotoxic activity against three human cancer cell lines, i.e., HCT-116, MCF-7, and HeLa. The resulting experimental data for the HCT-116 cell lines were consequently used for QSAR analysis. QSAR models were constructed step by step for a series of structurally modified compounds with R^1^ = H (**Step 1**), Bn (**Step 2**), and 4-F-Bn (**Step 3**). The predictive performance of the models built for the compounds from **Step 3** allowed for the virtual design of a subsequent series modified in the *N*-1 position of imidazolidine ring with R^1^ = 3-F_3_C-Bn, 4-F_3_C-Bn, and 3,5-bis(F_3_C)Bn (**Step 4**).

## 2. Results and Discussion

### 2.1. Chemistry

**Scheme 1:**
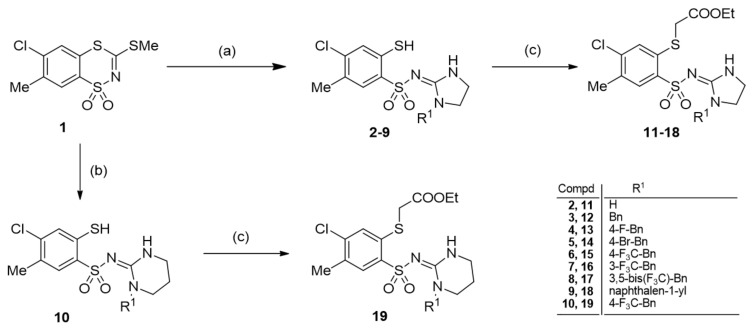
Synthesis of ethyl 2-{2-[*N*-(1-R^1^-imidazolidin-2-ylidene)sulfamoyl]-5-chloro-4-methylphenylthio}acetate **11**–**18** and ethyl 2-{2-[*N*-{1-[4-(trifluoromethyl)benzyl]tetrahydropyrimidin-2(1*H*)-ylidene}sulfamoyl]-5-chloro-4-methylphenylthio}acetate **19**. Reagents and conditions: (a) appropriate diamine (0.04 mol), anhydrous MeOH, reflux 70–240 h; (b) *N*-(4-trifluoromethylbenzyl)propane-1,3-diamine (0.04 mol), anhydrous MeOH, reflux 123 h; (c) ethyl 2-bromoacetate (0.024 mol), TEA (0.02 mol), DCM.

The necessary substrates, i.e., 4-chloro-2-mercapto-5-methyl-*N*-(1-R^1^-benzylimidazolidin-2-ylidene)benzenesulfonamide (**2**, **3**), were synthesized by the methods previously described [23,24]. The novel substrates **4**–**9** and **10** were obtained analogously to the reaction of **1** with either ethane-1,2-diamine, *N*-benzylethane-1,2-diamine (**4**–**9**) or *N*-(4-trifluoromethylbenzyl)propane-1,3-diamine (**10**). Subsequent reaction of **2**–**10** with ethyl bromoacetate and triethylamine (TEA) in methylene chloride (DCM), led to the appropriate novel ethyl 2-{2-[*N*-(imidazolidin-2-ylidene)sulfamoyl or *N*-(tetrahydropyrimidin-2(1*H*)-ylidene)sulfamoyl]-5-chloro-4-methylphenylthio}acetate derivatives **11**–**18** and **19**, respectively (Scheme 1).

**Scheme 2:**
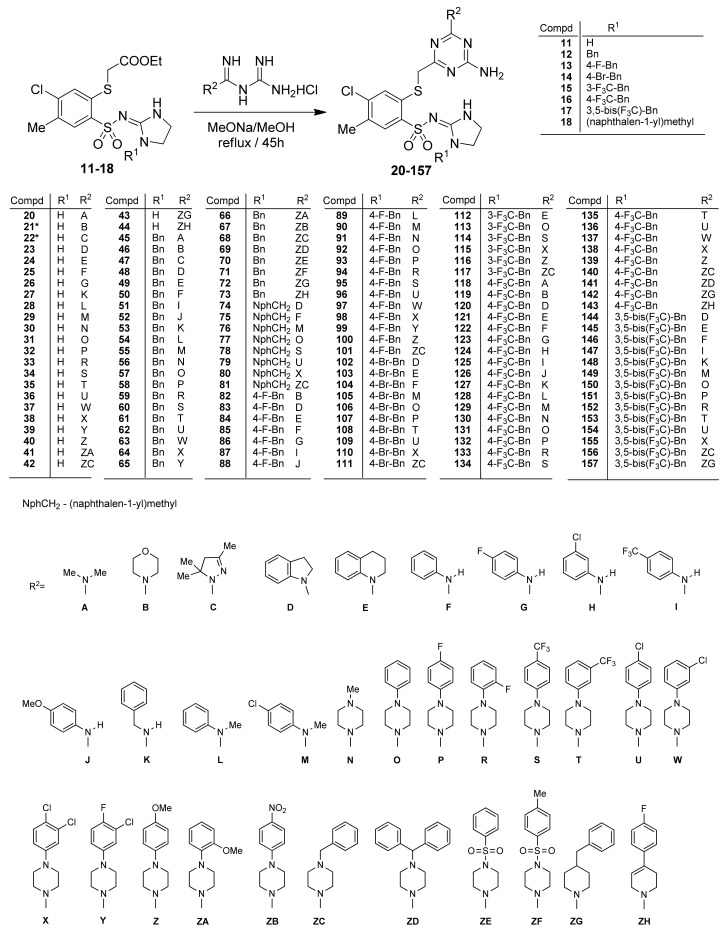
Synthesis of 2-[(4-amino-6-R^2^-1,3,5-triazin-2-yl)methylthio]-*N*-(1-R^1^-imidazolidin-2-ylidene)-4-chloro-5-methylbenzenesulfonamides (**20**–**157**). Compounds synthesized previously [31].

**Scheme 3:**
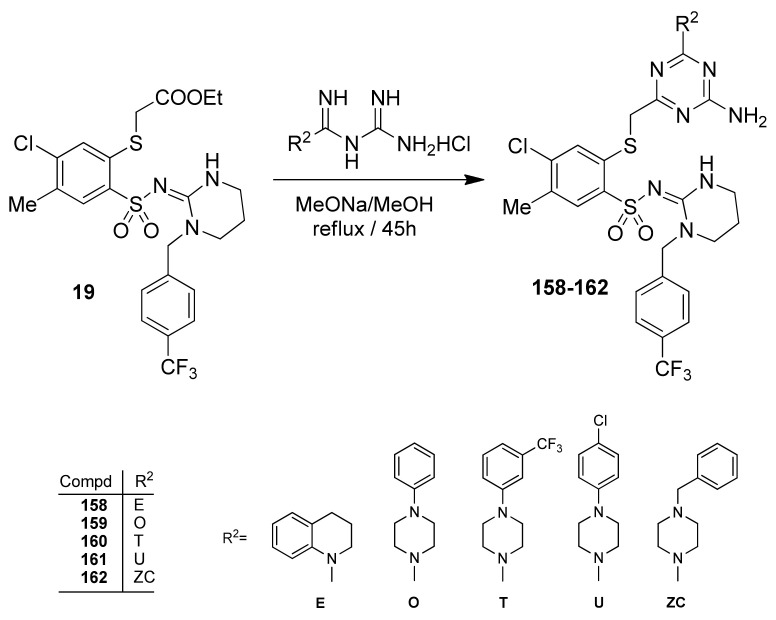
Synthesis of 2-[(4-amino-6-R^2^-1,3,5-triazin-2-yl)methylthio]-*N*-{1-[4-(trifluoromethyl)benzyl]tetrahydropyrimidin-2(1*H*)-ylidene}-4-chloro-5-methylbenzenesulfonamides (**158**–**162**).

Aiming for the synthesis of the final 6-substituted 4-amino-1,3,5-triazin-2-yl derivatives **20**–**157** (Scheme 2) and **158**–**162** (Scheme 3), the esters **11**–**18** or **19** were reacted with the appropriate biguanide hydrochlorides [25,26,27,28,29,30] in MeONa/MeOH solution at reflux for 45 h.

The structures of the final compounds **20**–**162** were confirmed with the IR and NMR methods, as well as HRMS (ESI-TOF) or MALDI-TOF/TOF, elemental analysis (C,H,N) and X-ray crystallography of the representative compound **27**.

**Figure 4:**
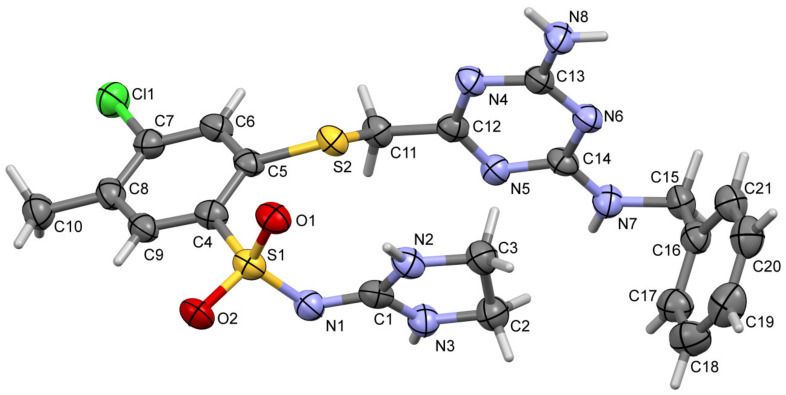
Molecular structure of compound **27**, showing the atom-labelling scheme. Solvent molecules omitted. Displacement ellipsoids are shown at 50% probability.

**Figure 5:**
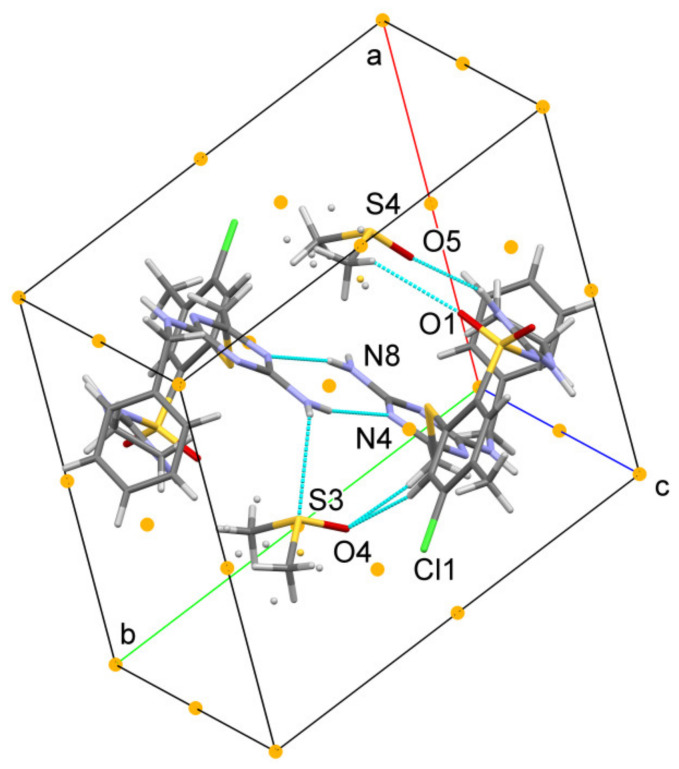
Crystal packing and hydrogen bonding in **27**. Molecules of the main component are linked by the ring-type hydrogen bond motif $R_{2}^{2}\left(8\right)$ NH^…^N (using N4 and N8 atoms) located at the inversion center (drawn as the orange ball). The two dimethylsulfoxide molecules are interacting with the sulfonamide through NH^…^O, CH^…^O or NH^…^S hydrogen bonds.

X-ray crystallography was undertaken to study the structure of the representative compound **27**. Compound **27** forms transparent crystals satisfying the symmetry of the triclinic system, space group $P\bar{1}$ (no. 2). An asymmetric unit contains one molecule, and the whole unit cell is built from two molecules of the sulfonamide and two (disordered) molecules of solvent, DMSO, Z = 2. Most of the bond lengths and angles are in the expected ranges. The obtained molecular structure is presented in Figure 4 and Figure 5. The report and details on data collection, structure solution, refinement geometry parameters, and hydrogen bonding details for **27** are given in Supplementary Materials (Tables S1 and S2).

### 2.2. Cytotoxic Activity

Compounds **20**–**162** were evaluated in vitro for their effects on the viability of three cancer cell lines: HCT-116 (colon cancer), MCF-7 (breast cancer) and HeLa (cervical cancer), as well as the non-cancerous keratinocyte cell line (HaCaT). The concentration required for 50% inhibition of cell viability IC_50_ was calculated and, as a positive control, cisplatin was used. Analysis was performed using an MTT assay after 72 h of incubation. The results are shown in Table 1, Table 2, Table 3, Table 4 and Table 5.

The most active compounds **135**, **143**, **150** and **156** belonged to the R^1^ = 4-trifluoromethylbenzyl (**135**, **143**) and R^1^ = 3,5-bis(trifluoromethyl)benzyl (**150**, **156**) series and showed high activity (3.6–5.0 µM, 4.5–7.5 µM and 5.5–11.0 µM, respectively) against all tested cell lines, HCT-116, MCF-7 and HeLa. The selectivity indexes of the most active compounds ranged from 6.4 to 1.7 (Table 6). In turn, twelve compounds, **20**–**23**, **30**, **67**, **69**–**71**, **79**–**80** and **118**, were inactive for all tested cell lines, which is about eight percent of the tested compounds (Table 1, Table 2, Table 3, Table 4 and Table 5).

As shown in Table 1, Table 2, Table 3, Table 4 and Table 5 the HCT-116 cell line presented the relatively highest susceptibility (IC_50_ < 6.0 µM) towards the tested compounds, with the highest activity displayed by the seventeen compounds **73**, **111**, **114**–**115**, **134**–**136**, **140**, **142**–**143**, **150**, **154**–**158** and **162**. Whereas, activity against the MCF-7 cell line, with IC_50_ in the range 4.5–6.0 µM, was observed for eight compounds (**96**, **136**, **143**, **150**, **151**, **155**, **157** and **162**), and against HeLa by five compounds (**111**, **135**, **143**, **156** and **158**), with IC_50_ values ranging between 5.5 and 7.5 µM.

### 2.3. QSAR Analysis

**Figure 6:**
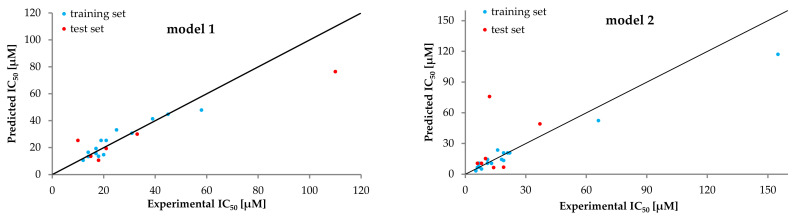

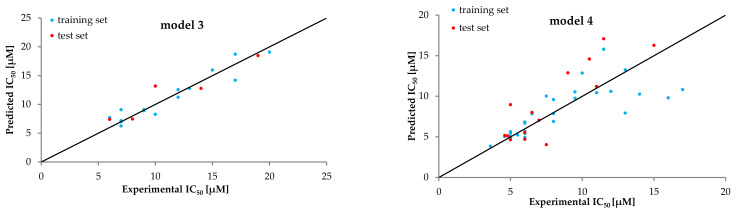
Scatter plots for *N*-(imidazolidin-2-ylidene)benzenesulfonamides **20**–**44** (**model 1**), *N*-(1-benzylimidazolidin-2-ylidene)benzenesulfonamides **45**–**73** (**model 2**) and *N*-[1-(4-fluorobenzyl)imidazolidin-2-ylidene]benzenesulfonamides **82**–**101** (**model 3**), as well as *N*-[1-(3-trifluoromethylbenzyl)imidazolidin-2-ylidene]benzenesulfonamides **112**–**117**, *N*-[1-(4-trifluoromethylbenzyl)imidazolidin-2-ylidene]benzenesulfonamides **118**–**143** and *N*-{1-[3,5-bis(trifluoromethyl)benzyl]imidazolidin-2-ylidene}benzenesulfonamides **144**–**157** (**model 4**) with experimental IC_50_ and predicted IC_50_ values of the training set (blue) and test set (red) for the HCT-116 cell line.

QSAR models were constructed to find a correlation between the activity against the HCT-116 cell line and the parameters of the molecules. On the basis of the QSAR models, successive series of derivatives were designed (mainly by modifying the R^1^ substituent), synthesized and evaluated in MTT tests step by step (Figure 6). As a result, the four QSAR models 1–4 have been developed in the four steps 1–4 (Table 7).

To compute the molecular descriptors, the energy of the molecules was minimized in the MOE software [32], using the MMFF94X force field and PM6 methods consecutively. Then, the energy was finally optimized in the GAMES software [33] using the STO3G HF method. Such optimized structures were used to calculate 365 molecular descriptors in the MOE software. QSAR models were obtained by stepwise linear progressive regression (MLR) in the STATISTICA software [34]. Compounds with IC_50_ outliers were removed from the analysis.

The statistical quality predictive ability of the QSAR models was evaluated using the coefficient of determination R^2^ and modified coefficients R^2^_adj_ and Q^2^_Loo_. Additionally, for each model, the residuals were normally distributed. The minimal value of the parameters were: R^2^_adj_ greater than 0.85 and Q^2^_LOO_ greater than 0.72, where, in the test set for each model, there was a minimum of 25% compounds.

The QSAR **Model 1** for compounds **20**–**44** (R^1^ = H) (Table 7, Figure 6) shows that the activity for the HCT-116 cell line correlates with higher values of descriptors related to atom counts and bond counts, *ast_violation*, and partial charge *PEOE_VSA-0* as well as the number of rings in the molecule *rings* (Table 8). Taking into account a residue analysis for the obtained model, it is noted that a high correlation coefficient of 89% is observed for the *rings* descriptor and the IC_50_ value for the HCT-116 cell line. To conclude, the model shows that, if the number of rings in the molecule increases, the IC_50_ value for the HCT-116 cell line will decrease. Based on this observation, compounds with one and two more rings, **45**–**73** (R^1^ = Bn) and **74**–**81** (R^1^ = 2-(naphtalen-2-yl)methyl group), were synthesized and investigated for their cytotoxic activity. The obtained experimental data were used to build a following model describing the quantitative structure–activity relationships.

In the second step, QSAR **Model 2** for compounds **45**–**73** (R^1^ = Bn) (Table 7, Figure 6) indicated that the IC_50_ value depends on the number of rotatable single bonds *b_1rotN*, the distance between the atoms and the charge of atoms *GCUT_PEOE_0*, the number of oxygen and nitrogen atoms *lip_acc* and the descriptor related to the distance between atoms and the surrounding atoms by other atoms *radius* (Table 8). It was observed that the predictor that corresponded most with antitumor activity for the HCT-116 model was the *lip_acc* descriptor, with a correlation coefficient of 70%. Based on this information, it was decided to synthesize compounds with one fluorine atom and one bromide atom in benzyl ring, compounds **82**–**101** (R^1^ = 4-F-Bn) and **102**–**111** (R^1^ = 4-Br-Bn), respectively. **Model 2** allowed us to obtain series of compounds with interesting cytotoxic effects that were used to perform new QSAR calculations in **Step 3**.

The following QSAR **Model 3** for compounds **82**–**101** (R^1^ = 4-F-Bn) (Table 7, Figure 6) paid attention to low values of descriptors related to adjacency and distance matrix *BCUT_SMR_1* as well as to total hydrophobic van der Waals surface area *Q_VSA_HYD*, which have beneficial impacts on high anticancer activity. The negative coefficient of the *vsurf_S* descriptor shows that a high value for the interaction field surface area is valuable for anticancer activity (Table 8). The residual analysis for **Model 3** indicated the best correlation coefficient for the *Q_VSA_HYD* descriptor. Based on this conclusion, in **Step 4** we decided to increase the hydrophobicity of compounds by replacing the fluorine atom with one and two trifluoromethyl groups in the benzyl ring. Consequently, in the next steps, compounds **112**–**117** (R^1^ = 3-F_3_C-Bn), **118**–**143** (R^1^ = 4-F_3_C-Bn) and **144**–**157** (R^1^ = 3,5-bis(F_3_C)Bn) were synthesized.

Based on the analysis of the QSAR equations, the series of above-described derivatives were designed and synthesized consistently step by step. Each synthesized series of compounds followed the previously drawn conclusions from the generated QSAR models. In this way, the most active series against the HCT-116 cell line **112**–**117** (R^1^ = 3-F_3_C-Bn), **118**–**143** (R^1^ = 4-F_3_C-Bn) and **144**–**157** (R^1^ = 3,5-bis(F_3_C)Bn) were achieved successfully. Additionally, compound **150** turned out to be the most prominent anticancer agent towards colorectal and breast tumors, exhibiting IC_50_ values of 3.6 μM for HCT-116 and 4.5 μM for the MCF-7 cell line.

The presented QSAR **Model 4** for compounds **112**–**117** (R^1^ = 3-F_3_C-Bn), **118**–**143** (R^1^ = 4-F_3_C-Bn) and **144**–**157** (R^1^ = 3,5-bis(F_3_C)Bn) (Table 7, Figure 6) indicated three descriptors connected with the hydrophobicity of compounds, *vsurf_DD12*, *vsurf_IW4* and *vsurf_IW5* (Table 8). High values of *vsurf_IW4* and low values of *vsurf_IW5* and *vsurf_DD12* favorably influence the cytotoxic activity toward the HCT-116 cell line.

### 2.4. SAR Analysis

Compounds containing 1,4-disubstituted piperazine rings at the R^2^ position are generally more active for the HCT-116 and MCF-7 cell lines. Comparison of a series containing the R^2^ = 4-phenylpiperazin-1-yl substituent (**31**, **57**, **92**, **106**, **131** and **150**) with a similar series bearing the R^2^ = PhNH group (**25**, **50**, **85**, **104**, **122** and **146**) clearly shows this advantage in activity (Table 9). We also found that the nature of the R^1^ substituent exerted a strong influence on the anticancer activity of the discussed compounds. Thus, replacement in both series of R^1^ = H (**31**, **25**) by the R^1^ = benzyl, 4-F-benzyl, 4-Br-benzyl, 4-(trifluoromethyl)benzyl or 3,5-bis(trifluoromethyl)benzyl moieties consequently leads to a significant increase in activity (Table 9).

Moreover, the activity of compounds containing the R^2^ = 4-phenylpiparazin-1-yl moiety depends significantly on the position and nature of the substituent of the phenyl ring. For example, compounds bearing the R^2^ = 4-(4-trifluoromethyl)phenylpiparazin-1-yl moiety (**34**, **60** and **134**) show slightly weaker activity compared to the series comprising the trifluoromethyl group in 3 position (**35**, **61** and **135**) (Table 10). Although the presence of the substituted phenyl moiety attached to the piperazine ring positively affects the activity, it should be noted that, in some cases, a loss of activity was noted, e.g., **67** (R^2^ = 4-(4-nitrophenyl)piperaizn-1-yl), **70** (R^2^ = 4-(phenylsulfonyl)piperaizn-1-yl) or **71** (R^2^ = 4-tosylpiperazin-1-yl) with IC_50_ (HCT-116) 168.0, 300.0 and 155.0 µM, IC_50_ (MCF-7) 153.0, 490.0 and 205.0 µM and IC_50_ (HeLa) 140.0, 550.0 and 265.0 µM, respectively (Table 1, Table 2, Table 3, Table 4 and Table 5).

### 2.5. Investigation of the Mechanism of Antiprolifeartive Activity

#### 2.5.1. MDM2 and p53 Protein Levels

**Figure 7:**
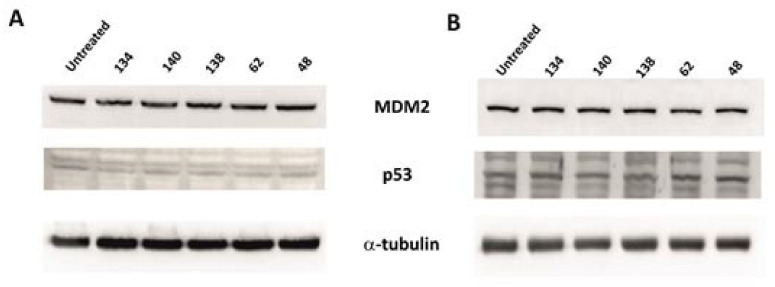
Effects on MDM2 and p53 protein levels upon treatment. Western blot analysis showing MDM2 and p53 protein levels in MCF-7 cells, untreated and treated with the indicated compounds in concentrations 4 µM (**A**) and 10 µM (**B**) for 24 h. Reference: β-tubulin.

Structural analogs of the examined compounds were found to bind into the p53-binding pocket of the MDM2 protein. Thus, the effects of the examined compounds on the inhibition of MDM2-p53 interactions were examined. MDM2 and p53 protein levels were determined with Western blot analysis after the treatment of MCF-7 cells with compounds selected for their high selectivity index (**48**, **62**, **134**, **138**, and **140**). Compounds were examined at different concentrations (4 µM and 10 µM) following a 24-h incubation (Figure 7). Western blot analysis revealed no changes in either MDM2 or p53 levels, indicating that p53 stability does not change upon compound treatment.

#### 2.5.2. Cell Cycle Analysis

**Figure 8:**
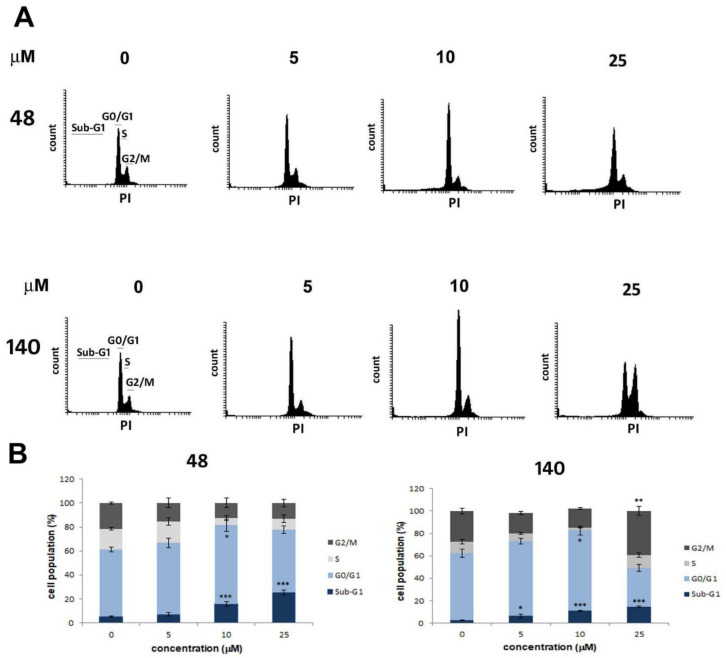
Cell cycle arrest in MCF-7 cells induced by compounds **48** and **140**. Cells were treated with the indicated concentrations of the compounds, and the cell cycle distribution was analyzed with flow cytometry. (**A**) Histograms showing the distribution of cell populations in the sub-G1, G0/G1, S and G2/M phases of the cell cycle. (**B**) Graphs demonstrating the percentages of cells in the depicted phases of the cell cycle. Values represent the mean ± SD of three independent experiments. Data were analyzed by one-way ANOVA with Tukey’s post hoc test [*p* < 0.05 (*), *p* < 0.01 (**), *p* < 0.001 (***)].

P53 controls cell proliferation through the regulation of the cell cycle. In order to determine p53-independent cell cycle arrest, the influence of selected compounds on the cell cycle distribution in MCF-7 cells was analyzed. Cells were treated with a concentration range of two selected compounds (**140** and **48**) for 48 h. Cell cycle distribution was analyzed with flow cytometry. In the case of compound **48**, a significant increase in the G0/G1 phase of the cell cycle was observed at a concentration of 10 µM, while a concentration-dependent increase in the sub-G1 population of the cell cycle was observed, indicating possible apoptosis induction. Compound **140** at the lower concentration of 10 µM increased the G0/G1 population, whereas at the higher examined concentration of 25 µM, an increase in the G2/M phase of the cell cycle was observed. Alongside G0/G1 and G2/M arrest, a concentration-dependent increase in the sub-G1 fraction of the cell cycle was observed upon treatment with compound **140**. However, a higher increase in the sub-G1 population was observed in compound-**48**-treated cells (Figure 8).

#### 2.5.3. Cytotoxic Activity toward Wild-Type and Mutant p53 Cell Lines

Cell cycle analysis showed a p53-independent mechanism of action for the examined compounds. Thus, the ability of the selected compounds to exert cytotoxic activity toward cell lines harboring mutant p53 was examined. Compounds displaying a high selectivity index (Table 6) were selected for the analysis of their activity toward ovarian and breast cancer cells with wild-type p53 (A2780, MCF-7) and mutant p53 (SKOV-3, T47D). The results of the MTT assay showed cytotoxic activity exerted by the compounds toward both types of cells (Table 11).

#### 2.5.4. Apoptosis Induction Analysis

**Figure 9:**
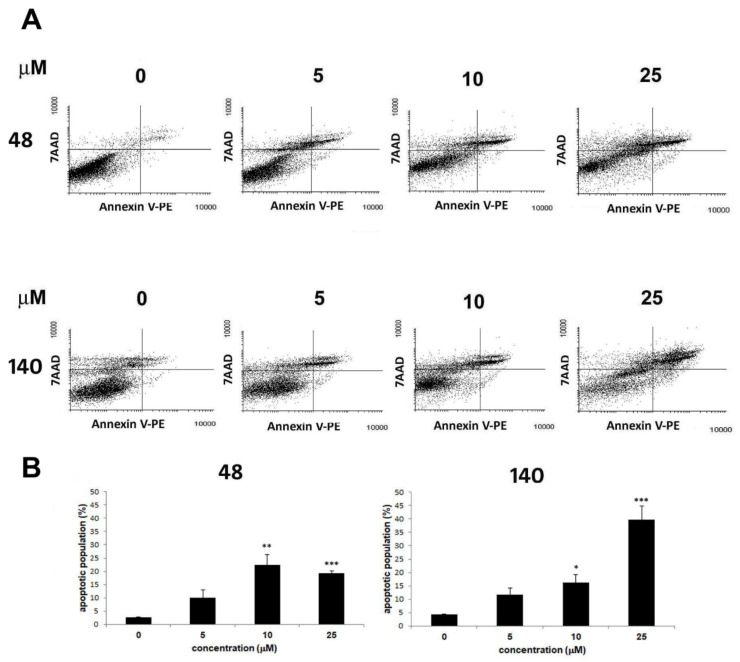
Induction of apoptosis by compounds **48** and **140** in MCF-7 cells. Cells were treated with the indicated concentrations of compounds **48** and **140** for 72 h, stained with Annexin V-PE and 7-AAD and analyzed with flow cytometry. (**A**) Dot plots showing early apoptotic (bottom right quadrant), late apoptotic (upper right quadrant), viable (lower left quadrant) and necrotic cell populations (upper left quadrant). (**B**) Graphs indicating early and late apoptotic cells. Values represent the mean ± SD of three repetitions. Data were analyzed by one-way ANOVA with Tukey’s post hoc test [*p* < 0.05 (*), *p* < 0.01 (**), *p* < 0.001 (***)].

The increase in the sub-G1 fraction of the cell cycle by compounds **140** and **48** suggested apoptosis induction. To confirm this, MCF-7 cells were treated with a concentration range of compounds **140** and **48** for 72 h. Flow cytometric analysis was performed with Annexin V-PE staining. The results showed a concentration-dependent increase in the apoptotic population of cells induced by the treatment with both compounds. The increase in apoptotic cells was mainly evident in the late apoptotic stage (upper right quadrant) (Figure 9).

**Figure 10:**
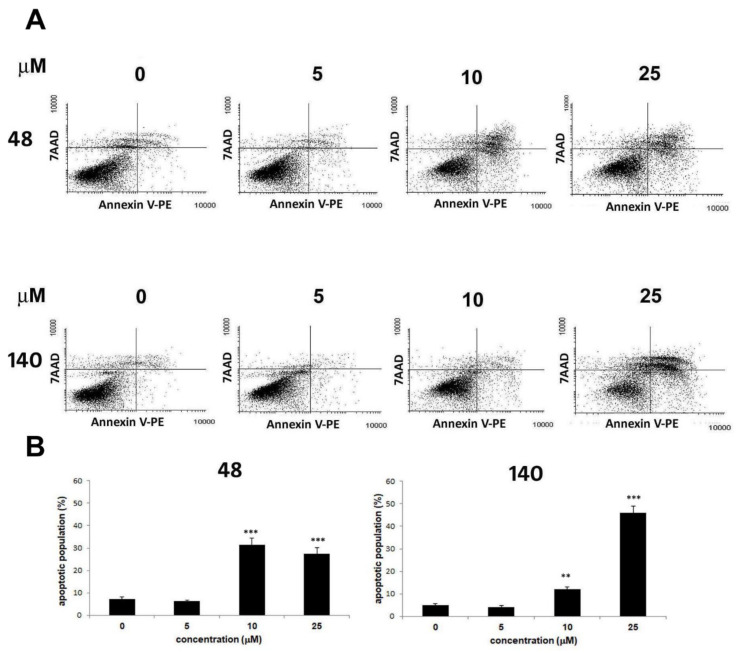
Induction of apoptosis by compounds **48** and **140** in T47D cells. Cells were treated with the indicated concentrations of compounds **48** and **140** for 72 h, stained with Annexin V-PE and 7-AAD, and analyzed with flow cytometry. (**A**) Dot plots showing early apoptotic (bottom right quadrant), late apoptotic (upper right quadrant), viable (lower left quadrant) and necrotic cell populations (upper left quadrant). (**B**) Graphs indicating early and late apoptotic cells. Values represent the mean ± SD of three repetitions. Data were analyzed by one-way ANOVA with Tukey’s post hoc test [*p* < 0.01 (**), *p* < 0.001 (***)].

Since the examined compounds exerted cytotoxic activity toward cell lines harboring mutant p53, the ability of compounds **140** and **48** to induce apoptosis in T47D cells with mutant p53 was examined. Similarly, as in the case of MCF-7 cells, a concentration-dependent increase in apoptotic cells was induced in T47D cells. Cell death was induced to a similar extent in cells harboring wild-type and mutant p53 (Figure 10).

#### 2.5.5. Discussion

To discern the mechanism of activity of the studied compounds, cell cycle arrest and apoptosis induction, associated with the activation of the p53 tumor suppressor protein, were analyzed. Our previous research showed that structural analogs of the compounds examined in the presented research bind into the p53-binding pocket of MDM2 protein [17]. This suggests possible induction of apoptosis by a mechanism similar to that of Nutlin-3a, a selective inhibitor of MDM2-p53 interactions. MDM2 is a promising target for developing anti-cancer therapies since it is as an important negative regulator of the p53 protein [35,36]. MDM2, a p53-specific E3 ubiquitin ligase, is the principal cellular antagonist of p53, limiting the p53 growth-suppressive function in unstressed cells. In these cells, MDM2 constantly monoubiquitinates p53 and thus is a critical step in mediating its degradation by nuclear and cytoplasmic proteasomes [37]. To explore the possibility that the observed cytotoxicity of the analyzed compounds results from their ability to block MDM2-p53 interactions and subsequently inhibit MDM2-mediated p53 degradation, p53 protein levels were examined by Western blot. An increase in p53 levels after treatment would signify its stabilization and promotion of cell death. The results of our analysis showed no change in p53 stability. However, cell cycle arrest and apoptosis were determined upon treatment with the examined compounds. These results pointed to a p53-independent mechanism of cell death induction.

The p53 pathway is an important target in cancer drug design. In cancers with wild-type p53, MDM2 inhibitors have found clinical application. However, in the case of tumors with mutant p53, targeting strategies are more challenging. Fifty percent of tumors harbor mutated or deleted p53, which leads to not only loss of p53 tumor suppressor functions, but also to gain-of-function driving tumor cell proliferation. Mutant p53 tumors display a more aggressive phenotype than their counterpart tumors harboring wild-type 53. Targeting strategies for mutant p53 tumors include reactivation of p53. However, direct targeting of mutant p53 is challenging due to its structural diversity. Thus, compounds inducing cell death in mutant p53 tumors can be considered important candidates in the treatment of cancers [38,39,40].

The results of the presented research showed cell death induction by compounds **48** and **140** in breast cancer cell lines harboring wild-type p53 (MCF-7 cells) and mutant p53 (T47D cells). Compounds **48** and **140** induced apoptosis in T47D cells to a similar extent as in MCF-7 cells. Research has shown that the p53 mutation in T47D cells facilitates cell survival [41]. This is associated with the disruption of signaling pathways controlled by wild-type p53 and induced by a p53 mutation, which leads to the activation of various compensatory mechanisms required to support tumor cell survival [40]. Thus, targeting signaling pathways activated by mutant p53 is a promising therapeutic strategy. Further research regarding the mechanism of cell death induced by the examined compounds could address the pharmacological value of these compounds.

## 3. Materials and Methods

### 3.1. General Information

The following instruments and parameters were used: Melting points were measured using a Stuart SMP30 (Bibby Scientific Limited, Stone Staffordshire UK) apparatus. IR spectra: KBr pellets, spectra were made on a Thermo Mattson Satellite FTIR spectrophotometer in the range 400–4000 cm^−1^. ^1^H NMR and ^13^C NMR spectra were obtained on a Varian Unity Plus 500 apparatus (Varian, Palo Alto, CA, USA) at 500 MHz, chemical shifts are expressed in parts per million (ppm) relative to (Me_4_Si) TMS as an internal standard. Elemental analyses for C, H and N were obtained on a CHN Elemental Analyzer (Perkin Elmer, Shelton, CT, USA) and obtained values are in agreement with the theoretical values within the ±0.4% range. Thin-Layer chromatography (TLC) was performed on Merck Kieselgel 60 F254 Plates (Merck, Darmstadt, Germany) and visualized with UV. High-resolution mass spectrometry (HRMS) was conducted on a TripleTOF 5600+ mass spectrometer (AB SCIEX, Framingham, MA, USA) equipped with a DuoSprayTM Ion Source and coupled with a Micro HPLC system Ekspert™ microLC 200 (Eksigent Redwood City, CA, USA); Column: HALO Fused-Core C18 (50 × 0.5 mm, 2.7 μm) (Eksigent), thermostated at 50 °C; Flow: 30 μL/min; Mobile Phase: A: 0.1% formic acid in water, B: 0.1% formic acid in acetonitrile; Isocratic program 100% B, 4 min. Moreover, a MALDI-TOF/TOF 5800 Sciex spectrometer was used to analyze the remaining obtained compounds. Samples were dissolved in methanol with 5% water content, and ferulic acid (FA, concentration 10 mg/mL in 33% acetonitrile/17% formic acid in water) was used as the matrix. Briefly, 0.8 microl of the sample was mixed on a measuring plate (Opti-TOF 384 MALDI plate insert) with 0.8 microl of the matrix and was allowed to crystallize freely at room temperature. Then, the plate was introduced into the spectrometer, and the measurements were carried out in reflector positive ion mode at a constant laser intensity. The commercially unavailable starting materials were obtained according to the following methods previously described: **1** [42], **2** [23] and **3** [24].

### 3.2. Synthesis

The procedures for the preparation of compounds **4**–**162** are provided in the Supplementary Materials (S.3.2. Synthesis).

### 3.3. X-ray Structure Determination

Crystal structures were investigated on an IPDS 2T dual beam diffractometer (STOE & Cie GmbH, Darmstadt, Germany). The crystal structure was solved using intrinsic phasing implemented in SHELXT and refined anisotropically using the program packages Olex2 [43] and SHELX-2015 [44,45]. Crystallographic data reported in this paper have been deposited with the Cambridge Crystallographic Data Centre as supplementary publication No. CCDC 2154790 for **27**. The data can be obtained free of charge from The Cambridge Crystallographic Data Centre via www.ccdc.cam.ac.uk/structures (accessed on 25 February 2022).

### 3.4. Cytotoxic Activity Screening and Determination of Mode Cell Death Induction

#### 3.4.1. Cell Culture

All chemicals, if not stated otherwise, were obtained from Sigma–Aldrich (St. Louis, MO, USA). The HCT-116 cell line was purchased from ATCC (ATCC-No: CCL-247), while the MCF-7, HeLa and HaCaT cell lines were purchased from Cell Lines Services (Eppelheim, Germany). Cells were cultured in Dulbecco’s modified Eagle’s medium (DMEM) supplemented with 10% fetal bovine serum, 2 mM glutamine, 100 units/mL penicillin and 100 µg/mL streptomycin. Cultures were maintained in a humidified atmosphere with 5% carbon dioxide at 37 °C in an incubator (Heraceus, HeraCell).

#### 3.4.2. Cell Viability Assay

Cell viability was examined using the MTT [3-(4,5-dimethylthiazol-2-yl)-2,5-diphenyltetrazolium bromide] assay. Cells were seeded in 96-well plates at a density of 3 × 10^3^ cells/well and treated for 72 h with the tested compounds in the concentration range of 1–100 µM. Then, MTT (0.5 mg/mL) was added to the medium and cells were further incubated for 2 h at 37 °C. In the next stage, cells were lysed with DMSO and the absorbance of the formazan solution was measured at 550 nm with a plate reader (1420 multilabel counter, Victor, Jügesheim, Germany). The experiment was performed in triplicate. Values are expressed as the mean ± SD of at least three independent experiments. Cisplatin was used as a positive control.

Compounds **48**, **62**, **134**, **138** and **140** were further examined toward MCF7 (breast cancer cell line, ATCC, HTB-22), T47D (breast cancer cell line, DSMZ ACC 739), A2780 (ovarian carcinoma cell line, ECACC, 93112519), and SKOV3 (ovarian carcinoma cell line, ATCC, HTB-77) cells. Cells were grown in Dulbecco’s Modified Eagle Medium (DMEM) supplemented with 10% fetal bovine serum (Thermo Fisher Scientific).

#### 3.4.3. Determination of Protein Levels with Western Blot

Protein extracts were heat-denaturated (95 °C) in Laemmli buffer (50 mM Tris/HCl, 0.01% Bromophenol Blue, 1.75% 2-mercaptoethanol, 11% glycerol, 2% SDS) and separated by 10–12% SDS/PAGE electrophoresis. Proteins were transferred to an Immobilon-P PVDF membrane (Merck Millipore, Burlington, MA, USA). The membranes were incubated for 1 h using 5% low-fat milk solution in 1× TBS (50 mM Tris-Cl, pH 7.5, 150 mM NaCl) as a blocking buffer and then overnight at 4 °C in the same blocking solution containing one of the following antibodies: anti-MDM2 (rabbit, Sigma-Aldrich AB-166, Burlington, MA, USA), anti-p53 (rabbit, Sigma-Aldrich S15) or α-tubulin (rabbit, 11H10, Cell Signaling, Danvers, MA, USA). After washing (3 × 10 min in TBS), membranes were incubated for 2 h in room temperature with the adequate HRP-conjugated secondary antibody: goat anti-rabbit IgG (1:5000, Abcam, Cambridge, UK; cat. 97051). Membranes were developed using an HRP detection kit WesternBright Quantum (Advansta, San Jose, CA, USA; cat. K-12042).

#### 3.4.4. Cell Cycle Analysis

Cell cycle distribution was analyzed with flow cytometry analysis with PI staining. MCF-7 cells were treated with compounds 48 and 140 (5, 10, 25 µM) for 48 h. Following treatment, the cells were collected and fixed in cold 70% ethanol for 24 h. The fixed cells were washed with PBS and incubated with 100 µg/mL RNAse (Invitrogen, Germany) and stained with 10 µg/mL PI (Invitrogen, Germany) for 30 min at RT. A total of 10^4^ cells were examined with a FACSCalibur cytometer (BD) and data was analyzed with Flowing software (version 2.5).

#### 3.4.5. Apoptosis Analysis

Levels of apoptosis were measured by flow cytometry using the Annexin V-PE Apoptosis Detection Kit I (BD Biosciences, Belgium) according to the manufacturer’s instructions. Apoptosis was assessed after a 72 h treatment of MCF7 and T47D cells with compounds **48** and **140** in the concentrations of 5, 10 and 25 µM. Proceeding cell treatment, cells were collected, washed with PBS and stained with Annexin V-phycoerythrin (PE) and 7-amino-actinomycin (7-AAD) in Annexin V binding buffer for 15 min at RT in the dark. After staining, cells were diluted in Annexin V binding buffer and analyzed on a BD FACSCalibur flow cytometer (BD Biosciences). At total of 10^4^ cells were analyzed from each sample and data were analyzed with Flowing software (version 2.5).

#### 3.4.6. Statistical Analysis

Values are expressed as means ± SD of at least three independent experiments. Statistical analysis was performed using GraphPad Prism 5.0 (GraphPad software). Differences between control and treated samples were analyzed by one-way ANOVA with Tukey’s post hoc tests. A *p* value of <0.05 was considered as statistically significant in each experiment.

### 3.5. Calculation of Molecular Descriptors and Generation of QSAR Models

Molecular descriptors were calculated in MOE 2019.01 software. Statistical analysis was carried out used TIBCO STATISTICA software 13.3.

## 4. Conclusions

We have synthesized a series of 2-[(4-amino-6-*N*-substituted-1,3,5-triazin-2-yl)methylthio]-*N*-(imidazolidin-2-ylidene)-4-chloro-5-methylbenzenesulfonamide **20**–**157** and 2-[(4-amino-6-substituted-1,3,5-triazin-2-yl)methylthio]-*N*-{1-[4-(trifluoromethyl)benzyl]tetrahydropyrimidin-2(1*H*)-ylidene}-4-chloro-5-methylbenzenesulfonamides derivatives **158**–**162** using cyclocondensation reactions of the 1,3,5-triazine moiety from previously obtained ethyl 2-{2-[*N*-(imidazolidin-2-ylidene)sulfamoyl]-5-chloro-4-methylphenylthio}acetate **11**–**18** and appropriate biguanide hydrochloride. The molecular structures of most compounds were confirmed by elemental analyses, and, for all compounds, NMR and IR spectroscopic methods were employed. For the representative compound **27**, an X-ray structure was determined.

The designing and development of new structures were supported with QSAR analysis. QSAR models were constructed in four steps and led to modification of the *N*-1 position of imidazolidine ring with benzyl, halogen-substituted benzyl and trifluoromethylbenzyl (3-F_3_C-Bn, 4-F_3_C-Bn, and 3,5-bis(F_3_C)Bn) groups consistently. Consequently, improvement of cytotoxic activity against the HCT-116 cell line was achieved and the highest cytotoxic activity was presented by series **112**–**117** (R^1^ = 3-F_3_C-Bn), **118**–**143** (R^1^ = 4-F_3_C-Bn) and **144**–**157** (R^1^ = 3,5-bis(F_3_C)Bn).

All obtained compounds **20**–**162** were investigated on three tumor cell lines: colon cancer (HCT-116), breast cancer (MCF-7), cervical cancer (HeLa) and non-cancerous keratinocyte cell line (HaCaT). Compounds containing the substituent R^1^ = 3,5-bis(trifluoromethyl)benzyl showed the highest cytotoxic activity against the tested cancer lines. Compounds from the series of derivatives containing the substituents R^1^ = 3,5-bis(trifluoromethyl)benzyl and R^1^ = 4-trifluoromethylbenzyl showed the highest cytotoxic activity against the tested cancer lines, with IC_50_ values ranging from 3.6 µM to 11.0 µM. Compounds **48** R^1^ = benzyl (IC_50 HCT-116, MCF-7_ = 6.0 and 7.0 µM; SI _HCT-116, MCF-7_ = 5.5 and 4.7) and **140** R^1^ = 4-trifloromethylbenzyl (IC_50 HCT-116, HeLa_ = 5.0 and 8.0 µM; SI _HCT-116, HeLa_ = 4.4 and 2.7) were shown to induce cell cycle arrest in the G0/G1 and G2/M phases of the cell cycle. Furthermore, apoptosis was induced in wt-p53 and mutant p53 cells in a p53-independent manner. The indicated selectivity index in comparison to the control compound as well as the established mechanism of activity shows the promising potential of the utilized scaffolds in this research for anticancer compound design.

## Figures and Tables

**Table 1:** Cytotoxicity of *N*-(imidazolidin-2-ylidene)benzenesulfonamides **20**–**44** toward human cancer cell lines and non-cancerous line HaCaT. Cell lines: colon cancer (HCT-116), breast cancer (MCF-7), cervical cancer (HeLa), and the human keratinocyte cell line (HaCaT).

Compd	Substituent	IC_50_ [µM]			
		HCT-116	MCF-7	HeLa	HaCaT
**20**		110.0 ± 2.0	220.0 ± 6.0	115.0 ± 7.0	NT
**21**		350.0 ± 7.0	190.0 ± 2.0	510.0 ± 5.0	NT
**22**		140.0 ± 3.0	130.0 ± 4.0	110.0 ± 4.0	NT
**23**		142.0 ± 4.0	130.0 ± 3.0	150.0 ± 2.0	NT
**24**		20.0 ± 0.4	27.0 ± 0.3	22.0 ± 0.2	41.0 ± 1.0
**25**		45.0 ± 2.0	46.0 ± 2.0	46.0 ± 2.0	54.0 ± 2.0
**26**		33.0 ± 2.0	33.0 ± 1.0	34.0 ± 1.0	45.0 ± 2.0
**27**		39.0 ± 2.0	44.0 ± 1.0	74.0 ± 2.0	75.0 ± 3.0
**28**		58.0 ± 0.6	45.0 ± 0.5	80.0 ± 1.0	NT
**29**		25.0 ± 0.5	24.0 ± 0.2	32.0 ± 0.3	43.0 ± 2.0
**30**	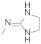	170.0 ± 3.0	145.0 ± 7.0	153.0 ± 5.0	NT
**31**		10.0 ± 0.4	16.0 ± 0.2	16.0 ± 0.1	26.0 ± 1.0
**32**		14.0 ± 0.5	16.0 ± 0.5	14.0 ± 0.5	28.0 ± 1.0
**33**		19.0 ± 0.5	26.0 ± 1.0	25.0 ± 1.0	41.0 ± 2.0
**34**		18.0 ± 1.0	12.0 ± 0.5	24.0 ± 2.0	41.0 ± 1.0
**35**		14.0 ± 0.7	16.0 ± 1.0	16.0 ± 1.0	32.0 ± 2.0
**36**		12.0 ± 0.1	13.0 ± 0.1	12.0 ± 0.1	28.0 ± 1.0
**37**		18.0 ± 0.5	19.0 ± 1.0	24.0 ± 1.0	37.0 ± 2.0
**38**		15.0 ± 0.5	28.0 ± 1.0	19.0 ± 1.0	35.0 ± 1.0
**39**		15.0 ± 1.0	17.0 ± 1.0	18.0 ± 1.0	37.0 ± 2.0
**40**		21.0 ± 0.5	28.0 ± 2.0	23.0 ± 1.0	45.0 ± 3.0
**41**		21.0 ± 1.0	46.0 ± 1.0	39.0 ± 2.0	51.0 ± 3.0
**42**		31.0 ± 1.0	31.0 ± 2.0	30.0 ± 1.5	61.0 ± 4.0
**43**		17.0 ± 1.0	22.0 ± 0.5	19.0 ± 0.5	43.0 ± 2.0
**44**		14.0 ± 0.5	16.0 ± 0.5	14.0 ± 0.5	28.0 ± 1.0
**cisplatin**		**3.8 ± 0.1**	**3.0 ± 0.1**	**2.2 ± 0.1**	**7.7 ± 0.2**

NT–not tested; IC_50_ values are expressed as the mean ± SD of at last three independent experiments.

**Table 2:** Cytotoxicity of *N*-(1-benzylimidazolidin-2-ylidene)benzenesulfonamides **45**–**73** and *N*-(1-naphthylimidazolidin-2-ylidene)benzenesulfonamides **74**–**81** toward human cancer cell lines and non-cancerous line HaCaT. Cell lines: colon cancer (HCT-116), breast cancer (MCF-7), cervical cancer (HeLa), and the human keratinocyte cell line (HaCaT).

Compd	Substituent	IC_50_ [µM]			
		HCT-116	MCF-7	HeLa	HaCaT
**45**		19.0 ± 0.4	22.0 ± 0.2	34.0 ± 1.0	43.0 ± 2.0
**46**		21.0 ± 0.2	23.0 ± 0.7	37.0 ± 0.4	NT
**47**		19.0 ± 0.2	20.0 ± 0.4	34.0 ± 0.3	45.0 ± 2.0
**48**		6.0 ± 0.1	7.0 ± 0.2	25.0 ± 0.3	33.0 ± 1.0
**49**		14.0 ± 0.1	17.0 ± 0.3	24.0 ± 0.5	38.0 ± 1.0
**50**		18.0 ± 0.2	43.0 ± 1.0	35.0 ± 2.0	51.0 ± 2.0
**51**		19.0 ± 0.6	10.0 ± 0.5	15.0 ± 1.0	220.0 ± 12.0
**52**		37.0 ± 0.5	64.0 ± 2.0	61.0 ± 2.0	92.0 ± 3.0
**53**		10.0 ± 0.5	11.0 ± 0.5	85.0 ± 2.0	81.0 ± 4.0
**54**		11.0 ± 0.1	12.0 ± 0.1	16.0 ± 0.2	28.0 ± 1.0
**55**		7.0 ± 0.1	10.0 ± 0.2	13.0 ± 0.1	39.0 ± 2.0
**56**		22.0 ± 1.0	54.0 ± 1.0	37.0 ± 1.0	58.0 ± 2.0
**57**	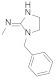	8.0 ± 0.1	8.0 ± 0.2	102.0 ± 2.0	48.0 ± 2.0
**58**		11.0 ± 0.3	17.0 ± 1.0	14.0 ± 2.0	29.0 ± 1.0
**59**		13.0 ± 0.3	18.0 ± 1.0	15.0 ± 1.0	30.0 ± 1.0
**60**		8.0 ± 0.3	14.0 ± 0.3	9.0 ± 0.5	23.0 ± 1.0
**61**		6.5 ± 0.1	9.0 ± 0.3	10.0 ± 0.2	21.0 ± 1.0
**62**		6.0 ± 0.1	7.0 ± 0.1	107.0 ± 1.0	32.0 ± 1.0
**63**		7.0 ± 0.1	9.0 ± 0.5	12.0 ± 0.5	24.0 ± 1.0
**64**		11.0 ± 0.4	14.0 ± 0.5	15.0 ± 1.0	28.0 ± 1.0
**65**		7.0 ± 0.2	11.0 ± 0.5	11.0 ± 0.3	23.0 ± 1.0
**66**		12.0 ± 0.4	29.0 ± 1.0	20.0 ± 1.0	36.0 ± 2.0
**67**		168.0 ± 8.0	153.0 ± 5.0	140.0 ± 7.0	NT
**68**		16.0 ± 0.5	16.0 ± 0.5	14.0 ± 0.5	32.0 ± 2.0
**69**		66.0 ± 2.0	122.0 ± 7.0	100.0 ± 6.0	128.0 ± 5.0
**70**		300.0 ± 18.0	490.0 ± 10.0	550.0 ± 21.0	NT
**71**		155.0 ± 6.0	205.0 ± 4.0	265.0 ± 8.0	27.0 ± 1.0
**72**		6.5 ± 0.1	9.0 ± 0.5	17.0 ± 0.5	28.0 ± 1.0
**73**		5.2 ± 0.2	7.5 ± 0.3	14.0 ± 0.3	29.0 ± 1.0
**74**		44.0 ± 2.0	12.0 ± 0.6	13.0 ± 0.5	81.0 ± 4.0
**75**		30.0 ± 2.0	8.0 ± 0.5	77.0 ± 5.0	110.0 ± 5.0
**76**	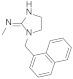	46.0 ± 1.0	25.0 ± 1.0	28.0 ± 2.0	99.0 ± 4.0
**77**		40.0 ± 1.0	29.0 ± 1.0	110.0 ± 5.0	220.0 ± 11.0
**78**		19.5 ± 0.6	11.0 ± 0.5	130.0 ± 8.0	280.0 ± 14.0
**79**		94.0 ± 2.0	98.0 ± 5.0	88.0 ± 3.0	NT
**80**		79.0 ± 2.0	89.0 ± 4.0	98.0 ± 5.0	NT
**81**		8.4 ± 0.3	8.0 ± 0.5	63.0 ± 1.0	240.0 ± 12.0
**cisplatin**		**3.8 ± 0.1**	**3.0 ± 0.1**	**2.2 ± 0.1**	**7.7 ± 0.2**

NT–not tested; IC_50_ values are expressed as the mean ± SD of at last three independent experiments.

**Table 3:** Cytotoxicity of *N*-[1-(4-fluoro or bromo)benzylimidazolidin-2-ylidene]benzenesulfonamides **82**–**111** toward human cancer cell lines and non-cancerous line HaCaT. Cell lines: colon cancer (HCT-116), breast cancer (MCF-7), cervical cancer (HeLa), and the human keratinocyte cell line (HaCaT).

Compd	Substituent	IC_50_ [µM]			
		HCT-116	MCF-7	HeLa	HaCaT
**82**		19.0 ± 0.8	12.0 ± 0.1	30.0 ± 1.0	54.0 ± 2.0
**83**		10.0 ± 0.5	14.0 ± 1.0	12.0 ± 3.0	32.0 ± 1.0
**84**		9.0 ± 0.5	7.0 ± 0.1	15.0 ± 0.6	27.0 ± 1.0
**85**		15.0 ± 0.5	23.0 ± 1.0	21.0 ± 4.0	38.0 ± 1.0
**86**		13.0 ± 0.5	15.0 ± 0.2	16.0 ± 0.2	35.0 ± 2.0
**87**		10.0 ± 0.4	7.0 ± 0.3	15.0 ± 1.0	34.0 ± 1.0
**88**		17.0 ± 0.5	31.0 ± 1.0	25.0 ± 2.0	43.0 ± 2.0
**89**		14.0 ± 0.7	18.0 ± 1.0	17.0 ± 0.6	31.0 ± 1.0
**90**		12.0 ± 0.2	17.0 ± 1.0	15.0 ± 0.6	30.0 ± 2.0
**91**	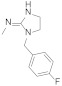	20.0 ± 1.0	13.0 ± 0.4	34.0 ± 2.0	54.0 ± 2.0
**92**		7.0 ± 0.3	8.0 ± 0.4	8.0 ± 0.5	18.0 ± 1.0
**93**		7.0 ± 0.3	7.0 ± 0.5	8.0 ± 0.4	18.0 ± 1.0
**94**		9.0 ± 0.2	14.0 ± 0.5	12.0 ± 1.0	25.0 ± 1.0
**95**		7.0 ± 0.3	7.0 ± 0.1	8.0 ± 0.4	18.0 ± 1.0
**96**		6.0 ± 0.3	6.0 ± 0.2	9.0 ± 0.3	22.0 ± 1.0
**97**		6.0 ± 0.2	7.0 ± 0.4	9.0 ± 0.3	24.0 ± 1.0
**98**		7.0 ± 0.1	7.0 ± 0.4	8.0 ± 0.4	18.0 ± 1.0
**99**		17.0 ± 1.0	17.0 ± 1.0	18.0 ± 0.5	30.0 ± 1.0
**100**		8.0 ± 0.2	7.0 ± 0.1	8.0 ± 0.5	18.0 ± 1.0
**101**		12.0 ± 0.5	11.0 ± 0.2	10.0 ± 0.6	21.0 ± 1.0
**102**		8.0 ± 0.4	7.5 ± 0.4	17.0 ± 1.0	33.0 ± 1.0
**103**		7.0 ± 0.1	6.5 ± 0.3	16.0 ± 0.5	28.0 ± 1.0
**104**		17.0 ± 1.0	13.0 ± 0.4	25.0 ± 1.0	44.0 ± 2.0
**105**	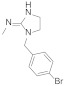	13.0 ± 0.8	10.0 ± 0.2	17.0 ± 0.5	35.0 ± 2.0
**106**		8.5 ± 0.1	6.5 ± 0.4	82.0 ± 4.0	110.0 ± 4.0
**107**		8.5 ± 0.3	7.0 ± 0.2	58.0 ± 2.0	76.0 ± 4.0
**108**		10.5 ± 0.5	11.0 ± 0.5	11.0 ± 0.5	24.0 ± 1.0
**109**		23.0 ± 1.0	22.0 ± 0.5	36.0 ± 2.0	55.0 ± 2.0
**110**		20.0 ± 0.6	23.0 ± 1.0	85.0 ± 5.0	123.0 ± 6.0
**111**		5.6 ± 0.3	7.0 ± 0.2	7.5 ± 0.3	18.0 ± 1.0
**cisplatin**		**3.8 ± 0.1**	**3.0 ± 0.1**	**2.2 ± 0.1**	**7.7 ± 0.2**

IC_50_ values are expressed as the mean ± SD of at last three independent experiments.

**Table 4:** Cytotoxicity of *N*-[1-(3- or 4-trifluoromethyl)benzylimidazolidin-2-ylidene]benzenesulfonamides **112**–**143** and *N*-[1-(3,5-bis(trifluoromethyl)benzylimidazolidin-2-ylidene]benzenesulfonamides **144**–**157** toward human cancer cell lines and non-cancerous line HaCaT. Cell lines: colon cancer (HCT-116), breast cancer (MCF-7), cervical cancer (HeLa), and the human keratinocyte cell line (HaCaT).

Compd	Substituent	IC_50_ [µM]			
		HCT-116	MCF-7	HeLa	HaCaT
**112**		15.0 ± 0.3	17.0 ± 1.0	15.5 ± 1.0	29.0 ± 1.0
**113**	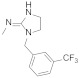	6.0 ± 0.1	6.5 ± 0.3	15.0 ± 1.0	31.0 ± 2.0
**114**		5.0 ± 0.2	6.5 ± 0.3	12.0 ± 0.3	27.0 ± 1.0
**115**		5.5 ± 0.2	6.5 ± 0.2	12.0 ± 1.0	23.0 ± 0.5
**116**		6.0 ± 0.2	8.5 ± 0.3	17.0 ± 1.0	31.0 ± 2.0
**117**		16.0 ± 0.5	17.0 ± 1.0	12.0 ± 0.2	36.0 ± 2.0
**118**		105.0 ± 4.0	10.0 ± 0.5	115.0 ± 2.0	>500
**119**		13.0 ± 0.4	13.5 ± 1.0	18.0 ± 1.0	41.0 ± 2.0
**120**		9.0 ± 0.5	11.0 ± 0.1	12.0 ± 0.5	24.0 ± 1.0
**121**		6.0 ± 0.1	14.0 ± 1.0	13.0 ± 1.0	24.0 ± 1.0
**122**		14.0 ± 0.4	20.0 ± 1.0	19.0 ± 0.5	35.0 ± 1.0
**123**		12.0 ± 0.5	21.0 ± 0.6	18.0 ± 1.0	30.0 ± 1.0
**124**		9.5 ± 0.1	10.0 ± 0.5	15.0 ± 0.2	31.0 ± 1.0
**125**		7.5 ± 0.2	7.5 ± 0.2	13.0 ± 0.5	25.0 ± 1.0
**126**		11.0 ± 0.6	21.0 ± 1.0	20.0 ± 1.0	37.0 ± 1.0
**127**		17.0 ± 0.5	17.5 ± 1.0	18.0 ± 1.0	39.0 ± 2.0
**128**		10.0 ± 0.3	14.0 ± 0.5	15.0 ± 0.4	33.0 ± 1.0
**129**	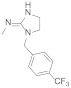	11.0 ± 0.1	14.0 ± 0.5	15.0 ± 1.0	31.0 ± 1.0
**130**		10.5 ± 0.4	8.0 ± 0.3	17.0 ± 0.5	36.0 ± 1.0
**131**		7.0 ± 0.2	15.0 ± 0.5	13.0 ± 0.5	25.0 ± 1.0
**132**		8.0 ± 0.2	17.0 ± 0.4	16.0 ± 1.0	26.0 ± 1.0
**133**		8.0 ± 0.3	16.0 ± 0.5	14.0 ± 1.0	25.0 ± 1.0
**134**		5.0 ± 0.2	11.0 ± 0.1	10.0 ± 0.6	22.0 ± 1.0
**135**		5.0 ± 0.2	6.5 ± 0.3	7.0 ± 0.2	19.0 ± 1.0
**136**		5.0 ± 0.1	6.0 ± 0.3	10.0 ± 0.4	21.0 ± 1.0
**137**		6.0 ± 0.2	7.0 ± 0.4	12.0 ± 0.6	21.0 ± 1.0
**138**		6.0 ± 0.2	9.0 ± 0.5	9.0 ± 0.6	22.0 ± 1.0
**139**		6.0 ± 0.1	13.0 ± 1.0	12.0 ± 1.0	24.0 ± 1.0
**140**		5.0 ± 0.2	10.0 ± 0.2	8.0 ± 0.5	22.0 ± 1.0
**141**		7.5 ± 0.2	9.5 ± 0.5	45.0 ± 2.0	180.0 ± 9.0
**142**		4.6 ± 0.1	6.5 ± 0.3	14.0 ± 0.2	29.0 ± 1.0
**143**		4.6 ± 0.1	6.0 ± 0.3	7.0 ± 0.4	19.0 ± 1.0
**144**		8.0 ± 0.5	8.0 ± 0.2	13.0 ± 0.2	27.0 ± 1.0
**145**		13.0 ± 0.5	13.0 ± 0.8	16.5 ± 1.0	36.0 ± 1.0
**146**		11.5 ± 0.5	12.5 ± 0.6	15.0 ± 1.0	32.0 ± 1.0
**147**		6.5 ± 0.3	8.0 ± 0.2	15.0 ± 1.0	28.0 ± 1.0
**148**	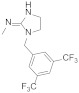	9.5 ± 0.2	8.5 ± 0.5	15.0 ± 1.0	29.0 ± 1.0
**149**		11.5 ± 0.6	11.0 ± 0.5	18.0 ± 1.0	33.0 ± 1.0
**150**		3.6 ± 0.2	4.5 ± 0.2	11.0 ± 0.3	23.0 ± 1.0
**151**		6.0 ± 0.1	6.0 ± 0.1	15.0 ± 1.0	28.0 ± 1.0
**152**		6.5 ± 0.3	6.5 ± 0.3	15.5 ± 1.0	29.0 ± 1.0
**153**		6.0 ± 0.3	8.0 ± 0.2	9.0 ± 0.5	19.0 ± 0.5
**154**		5.0 ± 0.1	6.5 ± 0.2	12.0 ± 1.0	26.0 ± 1.0
**155**		5.0 ± 0.1	6.0 ± 0.1	10.0 ± 0.1	19.0 ± 1.0
**156**		4.8 ± 0.1	7.5 ± 0.4	5.5 ± 0.2	17.0 ± 1.0
**157**		5.0 ± 0.1	6.0 ± 0.3	13.0 ± 0.5	23.0 ± 1.0
**cisplatin**		**3.8 ± 0.1**	**3.0 ± 0.1**	**2.2 ± 0.1**	**7.7 ± 0.2**

**Table 5:** Cytotoxicity of *N*-[1-(4-trifluoromethyl)benzyltetrahydropyrimidin-2(*1H*)-ylidene]benzenesulfonamides **158**–**162** toward human cancer cell lines and non-cancerous line HaCaT. Cell lines: colon cancer (HCT-116), breast cancer (MCF-7), cervical cancer (HeLa), and the human keratinocyte cell line (HaCaT).

Compd	Substituent	IC_50_ [µM]			
		HCT-116	MCF-7	HeLa	HaCaT
**158**		5.0 ± 0.3	8.0 ± 0.4	6.0 ± 0.3	17.0 ± 1.0
**159**	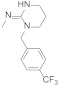	9.0 ± 0.5	10.0 ± 0.3	15.5 ± 0.5	29.0 ± 1.0
**160**		10.5 ± 0.4	12.0 ± 0.5	8.5 ± 0.3	23.0 ± 1.0
**161**		6.5 ± 0.1	6.5 ± 0.2	14.0 ± 0.5	27.0 ± 1.0
**162**		5.5 ± 0.3	6.0 ± 0.2	12.5 ± 0.5	25.0 ± 0.7
**cisplatin**		**3.8 ± 0.2**	**3.0 ± 0.1**	**2.2 ± 0.1**	**7.7 ± 0.2**

IC_50_ values are expressed as the mean ± SD of at last three independent experiments.

**Table 6:** Selectivity indexes (SI) of selected compounds toward cell lines: colon cancer (HCT-116), breast cancer (MCF-7), cervical cancer (HeLa).

Compd	Selectivity Index (SI)		
	HCT-116	MCF-7	HeLa
**48**	5.5	4.7	1.3
**55**	5.5	3.9	3.0
**62**	5.3	4.5	0.3
**73**	5.5	3.8	2.0
**114**	5.4	4.1	2.2
**134**	4.4	2.0	2.2
**140**	4.4	2.2	2.7
**150**	6.4	5.1	2.0
**157**	4.6	3.8	1.7
**162**	4.5	4.1	2.0
**cisplatin**	**2.0**	**2.5**	**3.5**

Selectivity index: IC_50_ value toward HaCaT cells/IC_50_ value toward cancer cell lines.

**Table 7:** Summary of the QSAR equations.

***STEP 1***	
**Model 1**	R^1^ = H, compounds: **20**–**44**
IC_50_ = −8.783228 ast_violation − 0.242646 PEOE_VSA-0 − 28.231708 rings + 212.156433 R^2^ = 0.88; R^2^_adj_ = 0.85; Q^2^_LOO_ = 0.80; F(3,11) = 26.76; *p* = 0.2 × 10^−4^; *N*_(train)_ = 15; *N*_(test)_ = 6	
***STEP 2***	
**Model 2**	R^1^ = Bn, compounds: **45**–**73**
IC_50_ = 10^0.345937 b_1rotN + 10.490686 GCUT_PEOE_0 + 0.507081 lip_acc − 0.318859 radius + 6.112894^ R^2^ = 0.88; R^2^_adj_ = 0.85; Q^2^_LOO_ = 0.72; F(4,15) = 28.17; *p* = 0.8 × 10^−6^; *N*_(train)_ = 20; *N*_(test)_ = 7	
***STEP 3***	
**Model 3**	R^1^ = 4-F-Bn, compounds: **82**–**101**
IC_50_= 256.006020 BCUT_SMR_1 + 0.139099 Q_VSA_HYD − 0.120301 vsurf_V + 201.402742 R^2^ = 0.91; R^2^_adj_ = 0.89; Q^2^_LOO_ = 0.85; F(3,11) = 37.67; *p* = 0.4 × 10^−5^; *N*_(train)_ = 15; *N*_(test)_ = 5	
***STEP 4***	
**Model 4**	R^1^ = 3-F_3_C-Bn, 4-F_3_C-Bn, 3,5-bis(F_3_C)Bn, compounds: **112**–**157**
IC_50_ = 10^[1/(−2.44368 vsurf_CW6 + 1.59455 VDistEq − 0.02515 vsurf_DD12 + 0.24868 vsurf_IW4 − 0.11745 vsurf_IW5 − 5.49371)] R^2^ = 0.84; R^2^_adj_ = 0.81; Q^2^_LOO_ = 0.74; F(5,25) = 26.27; *p* = 0.3 × 10^−8^; *N*_(train)_ = 31; *N*_(test)_ = 14	

R^2^_adj_—modified form R^2^ adj in the test set; Q^2^_LOO_—squared correlation coefficient for the test set; F—Fisher’s test; *p*—*p*-value for Fisher’s test for the whole equation; *N*_(train)_—training set; *N*_(test)_—test set.

**Table 8:** Type and chemical meaning of molecular descriptors in QSAR models.

	Descriptor	Type	Definition
**Model 1**	ast_violation	The atom count and bond count descriptors	Number of Astex fragment-likeness violations
	PEOE_VSA-0	Partial charge descriptor	Sum of the van der Waals area, where the partial charge is in the range −0.05 to 0.50
	rings	The atom count and bond count descriptors	The number of rings in molecule
**Model 2**	b_1rotN	The atom count and bond count descriptors	Number of rotatable single bonds. Conjugated single bonds are not included (e.g. ester and peptide bonds)
	GCUT_PEOE_0	Adjacency and distance matrix descriptors	The smallest GCUT descriptor using partial PEOE loads. The descriptor is related to the distance between the atoms and the charge of the atoms
	lip_acc	The atom count and bond count descriptors	The number of oxygen and nitrogen atoms
	radius	Adjacency and distance matrix descriptors	The descriptor is related to the distance between atoms and the surrounding atoms by other atoms
**Model 3**	BCUT_SMR_1	Adjacency and distance matrix descriptors	The BCUT descriptors using atomic contribution to molar refractivity (using the Wildman and Crippen SMR method) instead of partial charge.
	Q_VSA_HYD	Partial charge descriptors	Total hydrophobic van der Waals surface area.
	vsurf_S	Surface area, volume and shape descriptors	Interaction field surface area
**Model 4**	vsurf_CW6	Capacity factor	The descriptor is depend on the structure of molecule, connectivity and configuration of atoms
	VDistEq	Adjacency and distance matrix descriptor	The descriptor VDistEq is information content index of vertex distance equality.
	vsurf_DD12	Adjacency and distance matrix descriptor	The descriptor quantifies the contact distance of lowest and the 2nd lowest hydrophobic energy points of a molecule
	vsurf_IW4	Partial charge descriptor	Hydrophilic interaction energy moment
	vsurf_IW5	Partial charge descriptor	Hydrophilic interaction energy moment

**Table 9:** Comparison of IC_50_ value for the HCT-116 and MCF-7 cell lines for compounds **31**, **57**, **92**, **106**, **131**, **150**, with the substituent R^2^ = 4-phenylpiperazin-1-yl, and compounds **25**, **50**, **85**, **104**, **122**, **146**, with the substituent R^2^ = phenylamino.

Compd	31	57	92	106	131	150
R^1^ =	H	Bn	4-F-Bn	4-Br-Bn	4-F_3_C-Bn	3,5-bis(F_3_C)Bn
R^2^ =	4-Ph-piperazin-1-yl					
	IC_50_ [µM]					
HCT-116	45.0	8.0	7.0	8.5	7.0	3.6
MCF-7	46.0	8.0	8.0	6.5	15.0	4.5
Compd	**25**	**50**	**85**	**104**	**122**	**146**
R^1^ =	H	Bn	4-F-Bn	4-Br-Bn	4-F_3_C-Bn	3,5-bis(F_3_C)Bn
R^2^ =	PhNH					
	IC_50_ [µM]					
HCT-116	45.0	18.0	15.0	17.0	14.0	11.5
MCF-7	46.0	43.0	23.0	13.0	20.0	12.5

**Table 10:** Comparison of IC_50_ values for the HCT-116, MCF-7 and HeLa cell lines for compounds **34**, **60**, **134**, with R^2^ = S = 4-[4-(trifluoromethyl)phenyl]piperazin-1-yl, and compounds **35**, **61**, **135**, with R^2^ = T = 4-[3-(trifluoromethyl)phenyl]piperazin-1-yl.

Compd	34	35	60	61	134	135
R^1^ =	H	H	Bn	Bn	4-F_3_C-Bn	4-F_3_C-Bn
R^2^ =	S	T	S	T	S	T
	IC_50_ [µM]					
HCT-116	18.0	14.0	8.0	6.5	5.0	5.0
MCF-7	12.0	16.0	14.0	9.0	11.0	6.5
HeLa	24.0	16.0	9.0	10.0	10.0	7.0
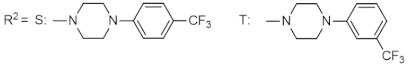						

**Table 11:** Cytotoxicity of compounds **48**, **62**, **134**, **138** and **140** toward ovarian cancer (A2780, SKOV-3) and breast cancer (MCF-7, T47D) cell lines.

Compd	Substituent	IC_50_ [µM]				
		A2780	SKOV3	MCF-7	T47D	HaCaT
**48**	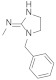	4.0 ± 0.1	8.1 ± 0.1	7.8 ± 0.1	4.8 ± 0.1	25.0 ± 1.0
**62**		3.9 ± 0.1	10.1 ± 0.1	10.4 ± 0.1	4.9 ± 0.1	32.0 ± 1.0
**134**	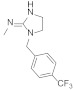	3.9 ± 0.1	5.7 ± 0.1	7.8 ± 0.1	3.2 ± 0.1	22.0 ± 1.0
**138**		2.2 ± 0.1	5.8 ± 0.1	8.2 ± 0.1	4.4 ± 0.1	22.0 ± 1.0
**140**		4.5 ± 0.1	6.6 ± 0.1	6.9 ± 0.1	5.3 ± 0.1	22.0 ± 1.0
**cisplatin**		**1.4 ± 0.1**	**71.0 ± 0.1**	**3.0 ± 0.1**	**3.1 ± 0.1**	**7.7 ± 0.2**

IC_50_ was determined following 72 h of incubation. IC_50_ values are expressed as the mean ± SD of at last three independent experiments.

## Data Availability

All data are available as Supplementary Materials.

## Supplementary Materials

The following supporting information can be downloaded at: https://www.mdpi.com/article/10.3390/ijms23137178/s1.
