# Supplementary material for: New 2-[(4-Amino-6-N-substituted-1,3,5-triazin-2-yl)methylthio]-N-(imidazolidin-2-ylidene)-4-chloro-5-methylbenzenesulfonamide Derivatives, Design, Synthesis and Anticancer Evaluation
Source: Int J Mol Sci. 2022 Jun 28;23(13):7178. doi: 10.3390/ijms23137178 (PMC9267128; doi:10.3390/ijms23137178)
Supplement: Supplementary file 1 [file ijms-23-07178-s001.zip › ijms-1757787-supplementary.pdf]

Supplementary materials

New 2-[(4-Amino-6-*N*-substituted-1,3,5-triazin-2-yl)methylthio]-*N*-(imidazolidin-2-ylidene)-4-chloro-5-methylbenzenesulfonamide Derivatives, Design, Synthesis and Anticancer Evaluation

Łukasz Tomorowicz [1], Beata Żołnowska [1], Krzysztof Szafrański [1], Jarosław Chojnacki [2], Ryszard Konopiński [3], Ewa A. Grzybowska [3], Jarosław Sławiński [1,*] and Anna Kawiak [4,*]

[1] Department of Organic Chemistry, Faculty of Pharmacy, Medical University of Gdańsk, Al. Gen. J. Hallera 107, 80-416 Gdańsk, Poland; ltomorowicz@gumed.edu.pl (Ł.T.); zolnowska@gumed.edu.pl (B.Ż.); k.szafranski@gumed.edu.pl (K.S.)
[2] Department of Inorganic Chemistry, Faculty of Chemistry, Gdańsk University of Technology, G. Narutowicza 11/12, 80-233 Gdańsk, Poland; jaroslaw.chojnacki@pg.edu.pl
[3] Maria Skłodowska-Curie National Research Institute of Oncology, ul. Roentgena 5, 02-781 Warszawa, Poland; ryszard.konopinski@pib-nio.pl (R.K.); ewa.grzybowska@pib-nio.pl (E.A.G.)
[4] Department of Biotechnology, Intercollegiate Faculty of Biotechnology, University of Gdańsk and Medical University of Gdańsk, ul. Abrahama 58, 80-307 Gdańsk, Poland
* Correspondence: jaroslaw@gumed.edu.pl (J.S.); anna.kawiak@biotech.ug.edu.pl (A.K.); Tel.: +48-58-349-10-98 (J.S.); +48-58-523-63-08 (A.K.)

Table of Contents

S.3. Materials and Methods……………………………………………………………..…………3
S.3.1. General Information……………………………………………………………………………..3
S.3.2. Synthesis……………………………………………………………………………………..3

Spectrum 1. ^{1}H NMR of compound **12** (500 MHz, DMSO-d_6). 44
Spectrum 2. ^{13}C NMR of compound **12** (125 MHz, DMSO-d_6). 45
Spectrum 3. ^{1}H NMR of compound **20** (500 MHz, DMSO-d_6). 46
Spectrum 4. ^{13}C NMR of compound **20** (125 MHz, DMSO-d_6). 47
Spectrum 5. ^{1}H NMR of compound **21** (500 MHz, DMSO-d_6). 48
Spectrum 6. ^{13}C NMR of compound **21** (125 MHz, DMSO-d_6). 49
Spectrum 7. ^{1}H NMR of compound **22** (500 MHz, DMSO-d_6). 50
Spectrum 8. ^{13}C NMR of compound **22** (125 MHz, DMSO-d_6). 51
Spectrum 9. ^{1}H NMR of compound **28** (500 MHz, DMSO-d_6). 52
Spectrum 10. ^{13}C NMR of compound **28** (125 MHz, DMSO-d_6). 53
Spectrum 11. ^{1}H NMR of compound **29** (500 MHz, DMSO-d_6). 54
Spectrum 12. ^{13}C NMR of compound **29** (125 MHz, DMSO-d_6). 55
Spectrum 13. ^{1}H NMR of compound **31** (500 MHz, DMSO-d_6). 56
Spectrum 14. ^{13}C NMR of compound **31** (125 MHz, DMSO-d_6). 57
Spectrum 15. ^{1}H NMR of compound **36** (500 MHz, DMSO-d_6). 58
Spectrum 16. ^{13}C NMR of compound **36** (125 MHz, DMSO-d_6). 59
Spectrum 17. ^{1}H NMR of compound **45** (500 MHz, DMSO-d_6). 60
Spectrum 18. ^{13}C NMR of compound **45** (125 MHz, DMSO-d_6). 61
Spectrum 19. ^{1}H NMR of compound **46** (500 MHz, DMSO-d_6). 62

Spectrum 20. ^{13}C NMR of compound **46** (125 MHz, DMSO-d$_6$). 63
Spectrum 21. ^{1}H NMR of compound **47** (500 MHz, DMSO-d$_6$). 64
Spectrum 22. ^{13}C NMR of compound **47** (125 MHz, DMSO-d$_6$). 65
Spectrum 23. ^{1}H NMR of compound **48** (500 MHz, DMSO-d$_6$)..66
Spectrum 24. ^{13}C NMR of compound **48** (125 MHz, DMSO-d$_6$). 67
Spectrum 25. ^{1}H NMR of compound **49** (500 MHz, DMSO-d$_6$). 68
Spectrum 26. ^{13}C NMR of compound **49** (125 MHz, DMSO-d$_6$). 69
Spectrum 27. ^{1}H NMR of compound **54** (500 MHz, DMSO-d$_6$). 70
Spectrum 28. ^{13}C NMR of compound **54** (125 MHz, DMSO-d$_6$). 71
Spectrum 29. ^{1}H NMR of compound **55** (500 MHz, DMSO-d$_6$). 72
Spectrum 30. ^{13}C NMR of compound **55** (125 MHz, DMSO-d$_6$). 73
Spectrum 31. ^{1}H NMR of compound **57** (500 MHz, DMSO-d$_6$). 74
Spectrum 32. ^{13}C NMR of compound **57** (125 MHz, DMSO-d$_6$). 75
Spectrum 33. ^{1}H NMR of compound **62** (500 MHz, DMSO-d$_6$). 76
Spectrum 34. ^{13}C NMR of compound **62** (125 MHz, DMSO-d$_6$). 77
Spectrum 35. ^{1}H NMR of compound **76** (500 MHz, DMSO-d$_6$). 78
Spectrum 36. ^{13}C NMR of compound **76** (125 MHz, DMSO-d$_6$). 79
Spectrum 37. ^{1}H NMR of compound **96** (500 MHz, DMSO-d$_6$). 80
Spectrum 38. ^{13}C NMR of compound **96** (125 MHz, DMSO-d$_6$). 81
Spectrum 39. ^{1}H NMR of compound **109** (500 MHz, DMSO-d$_6$)..82
Spectrum 40. ^{13}C NMR of compound **109** (125 MHz, DMSO-d$_6$). 83
Spectrum 41. ^{1}H NMR of compound **112** (500 MHz, DMSO-d$_6$). 84
Spectrum 42. ^{13}C NMR of compound **112** (125 MHz, DMSO-d$_6$). 85
Spectrum 43. ^{1}H NMR of compound **123** (500 MHz, DMSO-d$_6$..86
Spectrum 44. ^{13}C NMR of compound **123** (125 MHz, DMSO-d$_6$). 87
Spectrum 45. ^{1}H NMR of compound **140** (500 MHz, DMSO-d$_6$..88
Spectrum 46. ^{13}C NMR of compound **140** (125 MHz, DMSO-d$_6$). 89
Spectrum 47. ^{1}H NMR of compound **153** (500 MHz, DMSO-d$_6$). 90
Spectrum 48. ^{13}C NMR of compound **153** (125 MHz, DMSO-d$_6$). 91
Spectrum 49. ^{1}H NMR of compound **154** (500 MHz, DMSO-d$_6$..92
Spectrum 50. ^{13}C NMR of compound **154** (125 MHz, DMSO-d$_6$). 93

Crystallographic details..94
Crystal data and structure refinement of **27** (Table S1) 95
Hydrogen-bond geometry (Table S2) 96
References..97

S.3. Materials and Methods

S.3.1. General Information

The following instruments and parameters were used: melting points were measured using Stuart SMP30 (Bibby Scientific Limited, Stone Staffordshire UK) apparatus; IR spectra: KBr pellets, spectra were made on Thermo Mattson Satellite FTIR spectrophotometer in the range 400–4000 cm^{-1}; ^{1}H NMR and ^{13}C NMR spectra were obtained on Varian Unity Plus 500 apparatus (Varian, Palo Alto, CA, USA) at 500 MHz, chemical shifts are expressed in parts per million (ppm) relative to (Me_4Si) TMS as internal standard; elemental analyses for C, H and N were obtained on CHN Elemental Analyzer (Perkin Elmer, Shelton, CT, USA) and obtained values are in agreement with the theoretical values within ± 0.4% range. Thin-Layer chromatography (TLC) was performed on Merck Kieselgel 60 F254 Plates (Merck, Darmstadt, Germany) and visualized with UV. High resolution mass spectrometry (HRMS) was conducted on a TripleTOF 5600+ mass spectrometer (AB SCIEX, Framingham, MA, USA) equipped with a DuoSprayTM Ion Source and coupled with Micro HPLC system Ekspert™ microLC 200 (Eksigent Redwood City, CA, USA); Column: HALO Fused-Core C18 (50 × 0.5 mm, 2.7 μm) (Eksigent), thermostated at 50 °C; Flow: 30 μL/min; Mobile Phase: A: 0.1% formic acid in water, B: 0.1% formic acid in acetonitrile; Isocratic program 100% B, 4 min. Moreover, the MALDI-TOF/TOF 5800 Sciex spectrometer was used to analyze the remaining obtained compounds. Samples were dissolved in methanol with 5% water content and ferulic acid (FA, concentration 10 mg / ml in 33% acetonitrile / 17% formic acid in water) was used as the matrix. Briefly, 0.8 microl of the sample was mixed on a measuring plate (Opti-TOF 384 MALDI plate insert) with 0.8 microl of the matrix and was allowed to crystallize freely at room temperature. Then, the plate was introduced into the spectrometer and the measurements were carried out in reflector positive ion mode at a constant laser intensity.The commercially unavailable starting materials were obtained according to the following methods previously described: **1** [42], **2** [23] and **3** [24].

S.3.2. Synthesis

S.3.2.1. General Procedure for the preparation of 4-chloro-2-mercapto-5-methyl-*N*-(imidazolidin-2-ylidene)benzenesulfonamide **4–9**

To the solution of 6-chloro-7-methyl-3-methylthio-1,4,2-benzodithiazine 1,1-dioxide (0.040 mol) **1** in anhydrous methanol an appropriate diamine (0.040 mol) was added. Then, the reaction mixture was stirred under reflux for 70-240 h. After cooling, the precipitate was filtered off and washed with methanol.

4-Chloro-2-mercapto-5-methyl-N-[1-(4-fluorobenzyl)imidazolidin-2-ylidene]benzenesulfonamide (**4**).

Starting from *N*-(4-fluorobenzyl)ethane-1,2-diamine (6.728 g, 0.040 mol). Reaction mixture was heated for 120 h. The title compound was obtained after refluxing with methanol (1:5) in an ultrasonic bath for 10 minutes . Yield 11.755 g (71%); m.p. 192–193 °C: IR (KBr): 3389 (N-H), 2991, 2954, 2885 (C-H), 2565 (SH), 1578, 1522, 1511, 1491 (C=N, $C=C_{Ar}$), 1276, 1137 (SO_2) cm^{-1}.

4-Chloro-2-mercapto-5-methyl-N-[1-(4-bromobenzyl)imidazolidin-2-ylidene]benzenesulfonamide (**5**).

Starting from *N*-(4-bromobenzyl)ethane-1,2-diamine (9.165 g, 0.040 mol). Reaction mixture was heated for 240 h. The title compound was obtained. Yield 14.435 g (76%); m.p. 163–165 °C. IR (KBr): 3397 (N-H), 2946, 2917, 2888, 2855 (C-H), 2554 (SH), 1588, 1573, 1523, 1489 (C=N, $C=C_{Ar}$), 1268, 1136 (SO_2) cm^{-1}.

4-Chloro-2-mercapto-5-methyl-N-[1-(4-trifluoromethylbenzyl)imidazolidin-2-ylidene]benzenesulfonamide (**6**).

Starting from *N*-[4-(trifluoromethyl)benzyl]ethane-1,2-diamine (8.729 g, 0.040 mol). Reaction mixture was heated for 72 h. The title compound was obtained. Yield 17.900 g (95%); m.p. 195–196 °C. IR (KBr): 3394 (N-H), 2952, 2898 (C-H), 2560 (SH), 1597, 1580, 1523, 1495 (C=N, $C=C_{Ar}$), 1281, 1137 (SO_2) cm^{-1}.

4-Chloro-2-mercapto-5-methyl-N-[1-(3-trifluoromethylbenzyl)imidazolidin-2-ylidene]benzenesulfonamide (**7**).

Starting from *N*-[3-(trifluoromethyl)benzyl]ethane-1,2-diamine (8.729 g, 0.040 mol). Reaction mixture was heated for 240 h. The title compound was obtained. Yield 11.789 g (67%); m.p. 147–148 °C. IR (KBr): 3395 (N-H), 2983, 2948, 2887 (C-H), 2546 (SH), 1588, 1522, 1489 (C=N, $C=C_{Ar}$), 1271, 1164 (SO_2) cm^{-1}.

4-Chloro-2-mercapto-5-methyl-N-{1-[3,5-bis(trifluoromethyl)benzyl]imidazolidin-2-ylidene}benzenesulfonamide (**8**).

Starting from *N*-[3,5-bis(trifluoromethyl)benzyl]ethane-1,2-diamine (11.449 g, 0.040 mol). Reaction mixture was heated for 214 h. The title compound was obtained. Yield 14.256 g (67%); m.p. 147–148 °C. IR (KBr): 3379 (N-H), 2947, 2928, 2894 (C-H), 2568 (SH), 1577, 1522, 1494 (C=N, $C=C_{Ar}$), 1285, 1170 (SO_2) cm^{-1}.

4-Chloro-2-mercapto-5-methyl-N-[1-(naphthalen-1-ylmethyl)imidazolidin-2-ylidene]benzenesulfonamide (**9**).

Starting from *N*-(naphthalen-1-ylmethyl)ethane-1,2-diamine (8.011 g, 0.040 mol). Reaction mixture was heated for 70 h. The title compound was obtained. Yield 14.093 g (79%); m.p. 184–185 °C.

4-Chloro-2-mercapto-5-methyl-N-{1-[4-(trifluoromethyl)benzyl]tetrahydropyrimidin-2(1H)-ylidene}benzenesulfonamide (**10**).

To the 6-chloro-7-methyl-3-methylthio-1,4,2-benzodithiazine-1,1-dioxide (11.753 g, 0.040 mol) **1** in 100 mL anhydrous methanol, *N*-[4-(trifluoromethyl)benzyl]propane-1,3-diamine (9.290 g, 0.040 mol) was added. Then, the reaction mixture was stirred under reflux for 123 h. After cooling, the precipitate was filtered off and washed with methanol. The title compound was obtained. Yield 15.103 g (79%); m.p. 195–196 °C. IR (KBr): 3350 (N-H), 2951, 2923, 2895, 2863 (C-H), 2566 (SH), 1563, 1558 (C=N, $C=C_{Ar}$), 1261, 1135 (SO_2) cm^{-1}.

S.3.2.2. General procedure for the preparation of ethyl 2-{2-[*N*-(imidazolidin-2-ylidene)sulfamoyl]-5-chloro-4-methylphenylthio}acetate **11–18**

To the solution an appropriate 4-chloro-2-mercapto-5-methyl-*N*-[imidazolidin-2-ylidene]benzenesulfonamide **2–9** (0.020 mol) in dichloromethane, triethylamine (0.020 mol) and ethyl 2-bromoacetate (0.024 mol) were added sequentially. Then, the reaction mixture was stirred under reflux for 7-24 h and evaporate to dryness. Next to the obtained oil dichloromethane (40 mL) and water (150 mL) was added and stirred for 3 h. Organic layer was separated and washed with water (3 × 50 mL). Organic layer was dried over anhydrous magnesium sulfate and evaporated to dryness to give the pure product.

Ethyl 2-{5-chloro-2-[N-(1-imidazolidin-2-ylidene)sulfamoyl]-4-methylphenylthio}acetate (**11**).

Reaction mixture was heated for 20 h. The title compound was obtained. Yield 6.270 g (80%); m.p. 196–198 °C. IR (KBr): 3442 (N-H), 2991, 2906 (C-H), 1733 (C=O), 1610, 1533, 1492 (C=N, $C=C_{Ar}$), 1304, 1159 (SO_2) cm^{-1}; ^1H NMR (500 MHz, DMSO-d_6) δ: 1.17 (t, *J=7.1* Hz, 3H, $CH_2\mathbf{CH_3}$), 2.31 (s, 3H, CH_3), 3.41 (m, 4H, imidazolidin), 3.95 (s, 2H, S-CH_2), 4.11 (quartet, *J=6.84 Hz*, 2H, $C\mathbf{H_2}CH_3$), 7.40 (s, 1H, H-3), 7.48-7.50 (m, 2H, NH, imidazolidin), 7.87 (s, 1H, H-6) ppm; Anal. calcd. for $C_{14}H_{18}ClN_3O_4S_2$ (391.89); C, 49.92; H, 4.63; N, 10.72. Found: C, 42.90; H, 4.97; N, 10.70.

Ethyl 2-{2-[N-(1-benzylimidazolidin-2-ylidene)sulfamoyl]-5-chloro-4-methylphenylthio}acetate (**12**).

Reaction mixture was heated for 7 h. The title compound was obtained. Yield (78%) oil; IR (KBr): 3382 (N-H), 2977, 2925, 2901 (C-H), 1730 (C=O), 1588, 1576, 1530 (C=N, $C=C_{Ar}$), 1285, 1130 (SO_2) cm^{-1}; ^1H NMR (500 MHz, DMSO-d_6) δ: 1.14 (t, *J=7.1 Hz*, 3H, CH_3), 2.30 (s, 3H, CH_3), 3.29 (t, *J=8.55 Hz*, 2H,

imidazolidin), 3.44 (t, *J=8.8 Hz*, 2H, imidazolidin), 3.98 (s, 2H, S-CH_2), 4.09 (quartet, *J=7.2 Hz*, 2H, **CH_2**CH_3), 4.39 (s, 2H, (Ph-**CH_2**)), 7.21-7.44 (m, 5H, H_{Ar} and 1H, NH, imidazolidin and 1H, H-3), 7.89 (s, 1H, H-6) ppm; ^{13}C NMR (125 MHz, DMSO-d_6) δ: 14.64, 19.65, 35.13, 41.51, 44.98, 47.85, 61.82, 128.13, 128.23, 128.58, 129.20, 131.22, 132.87, 135.22, 136.93, 137.27, 140.15, 158.80, 169.71 ppm; Anal. calcd. for $C_{21}H_{24}ClN_3O_4S_2$ (482.02); C, 52.33; H, 5.02; N, 8.72. Found: C, 52.30; H, 5.08; N, 8.70.

Ethyl 2-[5-chloro-2-{N-[1-(4-fluorobenzyl)imidazolidin-2-ylidene]sulfamoyl}-4-methylphenylthio]acetate (**13**).

Reaction mixture was heated for 7 h. The title compound was obtained. Yield 9.000 g (90%); m.p. 98–99 °C. IR (KBr): 3383 (N-H), 2982, 2903 (C-H), 1740 (C=O), 1576, 1509 (C=N, C=C_{Ar}), 1270, 1174 (SO_2) cm^{-1}; ^{1}H NMR (500 MHz, DMSO-*d_6*) δ: 1.16 (t, *J=7.1 Hz*, 3H, CH_2**CH_3**), 2.32 (s, 3H, CH_3), 3.28-3.33 (m, 2H, imidazolidin), 3.43-3.47 (m, 2H, imidazolidin), 4.01 (s, 2H, S-**CH_2**), 4.10 (quartet, *J=7.0 Hz*, 2H, **CH_2**CH_3), 4.39 (s, 2H, (4-F-C_6H_4-**CH_2**)), 7.12-7.16 (m, 2H, $H_{Ar,\ 4\text{-}F\text{-}Ph}$), 7.28-7.31 (m, 2H, $H_{Ar,\ 4\text{-}F\text{-}Ph}$), 7.43-7.45 (m, 1H, NH, imidazolidin and 1H, H-3), 7.89 (s, 1H, H-6) ppm.

Ethyl 2-[2-{N-[1-(4-bromobenzyl)imidazolidin-2-ylidene]sulfamoyl}-5-chloro-4-methylphenylthio]acetate (**14**).

Reaction mixture was heated for 18 h. The title compound was obtained. Yield 9.872 g (88%); m.p. 110–111 °C. IR (KBr): 3379 (N-H), 3073 (C-H_{Ar}), 2982, 2919, 2900, 2855 (C-H), 1751 (C=O), 1581, 1533, 1487 (C=N, C=C_{Ar}), 1272, 1128 (SO_2) cm^{-1}; ^{1}H NMR (500 MHz, DMSO-*d_6*) δ: 1.16 (t, *J=7.35 Hz*, 3H, CH_2**CH_3**), 2.32 (s, 3H, CH_3), 3.29-3.33 (m, 2H, imidazolidin), 3.44-3.47 (m, 2H, imidazolidin), 4.01 (s, 2H, S-**CH_2**), 4.10 (quartet, *J=7.0 Hz*, 2H, **CH_2**CH_3), 4.37 (s, 2H, CH_2(4-Br-C_6H_4-**CH_2**)), 7.16-7.52 (m, 4H, H_{Ar} and 1H, NH, imidazolidin and 1H, H-3), 7.88 (s, 1H, H-6) ppm; Anal. calcd. for $C_{21}H_{23}BrClN_3O_4S_2$ (560.91); C, 44.97; H, 4.13; N, 7.49. Found: C, 44.84; H, 4.10; N, 7.54.

Ethyl 2-[5-chloro-2-{N-[1-(4-trifluoromethylbenzyl)imidazolidin-2-ylidene]sulfamoyl}-4-methylphenylthio]acetate (**15**).

Reaction mixture was heated for 24 h. The title compound was obtained. Yield 9.020 g (82%); m.p. 128–129 °C. IR (KBr): 3382 (N-H), 3074 (C-H_{Ar}), 2984, 2905 (C-H), 1715 (C=O), 1577, 1534, 1488 (C=N, C=C_{Ar}), 1380, 1173 (SO_2) cm^{-1}; ^{1}H NMR (500 MHz, DMSO-*d_6*) δ: 1.15 (t, *J=7.05 Hz*, 3H, CH_2**CH_3**), 2.32 (s, 3H, CH_3), 3.34-3.37 (m, 2H, imidazolidin), 3.46-3.50 (m, 2H, imidazolidin), 4.01 (s, 2H, S-**CH_2**), 4.09 (quartet, *J=7.2 Hz*, 2H, **CH_2**CH_3), 4.50 (s, 2H, CH_2(4-$CF_3C_6H_4$)), 7.43-7.69 (m, 4H, H_{Ar} and 1H, NH, imidazolidin and 1H, H-3), 7.88 (s, 1H, H-6) ppm.

Ethyl 2-[5-chloro-4-methyl-2-{N-[1-(3-trifluoromethylbenzyl)imidazolidin-2-ylidene]sulfamoyl}-phenylthio]acetate (**16**).

Reaction mixture was heated for 7 h. The title compound was obtained. Yield 9.790 g (89%); m.p. 126–127 °C. IR (KBr): 3382 (N-H), 3074 (C-H_{Ar}), 2984, 2905 (C-H), 1715 (C=O), 1577, 1534, 1488 (C=N, C=C_{Ar}), 1380, 1173 (SO_2) cm^{-1}; ^{1}H NMR (500 MHz, DMSO-*d_6*) δ: 1.14 (t, *J=7.05 Hz*, 3H, CH_2**CH_3**), 2.32 (s, 3H, CH_3), 3.34-3.37 (m, 2H, imidazolidin), 3.46-3.50 (m, 2H, imidazolidin), 4.01 (s, 2H, S-**CH_2**), 4.09 (quartet, *J=7.2 Hz*, 2H, **CH_2**CH_3), 4.50 (s, 2H, CH_2(4-$CF_3C_6H_4$)), 7.43-7.69 (m, 4H, H_{Ar} and 1H, NH, imidazolidin, 1H, H-3), 7.88 (s, 1H, H-6) ppm.

Ethyl 2-{5-chloro-2-[N-{1-[3,5-bis(trifluoromethyl)benzyl]imidazolidin-2-ylidene}sulfamoyl]-4-methylphenylthio}acetate (**17**).

Reaction mixture was heated for 24 h. The title compound was obtained. Yield 10.135 g (82%); m.p. 128–129 °C. IR (KBr): 3382 (N-H), 3074 (C-H_{Ar}), 2984, 2905 (C-H), 1715 (C=O), 1577, 1534, 1488 (C=N, C=C_{Ar}), 1380, 1173 (SO_2) cm^{-1}; ^{1}H NMR (500 MHz, DMSO-*d_6*) δ: 1.13 (t, *J=7.05 Hz*, 3H, CH_2**CH_3**), 2.32 (s, 3H, CH_3), 3.38-3.41 (m, 2H, imidazolidin), 3.49-3.52 (m, 2H, imidazolidin), 3.91 (s, 2H, S-**CH_2**), 4.06 (quartet, *J=7.2 Hz*, 2H, **CH_2**CH_3), 4.50 (s, 2H, CH_2(4-$CF_3C_6H_4$)), 7.43-7.69 (m, 4H, H_{Ar} and 1H, NH, imidazolidin and 1H, H-3), 7.88 (s, 1H, H-6) ppm.

Ethyl 2-[5-chloro-2-{N-[1-(naphthalen-1-ylmethyl)imidazolidin-2-ylidene]sulfamoyl}-4-methylphenylthio]acetate (**18**).

Reaction mixture was heated for 7 h. The title compound was obtained. Yield 9.152 g (86%); m.p. 183–185 °C. IR (KBr): 3384 (N-H), 2983, 2940, 2854 (C-H), 1729 (C=O), 1530, 1468 (C=N, $C=C_{Ar}$), 1263, 1132 (SO_2) cm^{-1}; ^1H NMR (500 MHz, DMSO-d_6) δ: 1.10 (t, *J=7.05 Hz*, 3H, CH_2CH_3), 2.34 (s, 3H, CH_3), 3.25 (t, *J=8.75 Hz*, 2H, imidazolidin), 3.43 (t, *J=8.8 Hz*, 2H, imidazolidin), 3.96 (s, 2H, S-CH_2), 4.04 (quartet, *J=7.2 Hz*, 2H, CH_2CH_3), 4.86 (s, 2H, CH_2-(1-methylnaphthyl)), 7.34-8.07 (m, 7H, H_{Ar} naphthyl, 1H, NH, imidazolidin and 1H, H-3 and 1H, H-6) ppm; Anal. calcd. for $C_{25}H_{26}ClN_3O_4S_2$ (532.07); C, 56.43; H, 4.93; N, 7.90. Found: C, 56.31; H, 4.85; N, 7.87.

Ethyl 2-[5-chloro-4-methyl-N-{1-[4-(trifluoromethyl)benzyl]tetrahydropyrimidin-2(1H)-ylidene}sulfamoyl]phenylthio}phenylthio]acetate (**19**).

To the solution of 4-chloro-2-mercapto-5-methyl-*N*-{1-[4-(trifluoromethyl)benzyl]tetrahydropyrimidin-2(1*H*)-ylidene}benzenesulfonamide **10** (9.559 g, 0.020 mol) in dichloromethane, triethylamine 2.79 mL (2.024 g, 0.020 mol) and ethyl 2-bromoacetate 26.72 mL (40.080 g, 0.024 mol) were added sequentially. Then, the reaction mixture was stirred under reflux for 24 h and evaporate to dryness. Next to the obtained oil dichloromethane (40 mL) and water (150 mL) was added and stirred for 3 h. Organic layer was separated and washed with water (3 × 50 mL). Organic layer was dried over anhydrous magnesium sulfate and evaporated to dryness to give the pure title compound. The title compound was obtained. Yield 8.799 g (78%); m.p. 92–93 °C. IR (KBr): 3353 (N-H), 2983, 2965, 2920, 2902 (C-H), 1745 (C=O), 1573, 1560 (C=N, $C=C_{Ar}$), 1263, 1126 (SO_2) cm^{-1}; ^1H NMR (500 MHz, DMSO-d_6) δ: 1.15 (t, *J=7.05 Hz*, 3H, $CH_2\mathbf{CH_3}$), 1.81 (quintet, *J=5.63 Hz*, 2H, CH_2-heksahydropirimidin), 2.30 (s, 3H, CH_3), 3.23 (t, *J=5.85 Hz*, 2H, heksahydropyrimidin), 3.27-3.30 (m, 2H, heksahydropirimidin), 3.91 (s, 2H, S-$\mathbf{CH_2}$), 4.09 (quartet, *J=7.1 Hz*, 2H, $\mathbf{CH_2}CH_3$), 4.70 (s, 2H, CH_2(4-CF_3-C_6H_4-$\mathbf{CH_2}$), 7.36-7.83 (m, 4H, H_{Ar} and 1H, NH, heksahydropirimidin and 1H, H-3 and 1H, H-6) ppm; Anal. calcd. for $C_{23}H_{25}ClF_3N_3O_4S_2$ (564.04); C, 48.98; H, 4.47; N, 7.45. Found: C, 49.03; H, 4.54; N, 7.50.

S.3.2.3. General procedure for the preparation of 6-Substituted 2-[(4-Amino-1,3,5-triazin-2-yl)methylthio]-*N*-(imidazolidin-2-ylidene)-4-chloro-5-methylbenzenesulfonamide **20–157**

To the solution of sodium methoxide prepared from sodium (0.0368 g, 1.60 mmol) and anhydrous methanol (7.5 mL), ethyl 2-{2-[*N*-(imidazolidin-2-ylidene)sulfamoyl]-5-chloro-4-methylphenylthio}acetate **11–18** (0.80 mmol) and the next appropriate biguanide hydrochloride (1.60 mmol) was added. The reaction mixture was stirred under reflux for 45 h. After cooling the precipitate was filtered off and dried, then stirred vigorously with water (25 mL) for 25 min. The crude product was purified by crystallization from the appropriate solvent or by refluxing with methanol , ethanol, acetonitrile or diethyl ether in an ultrasonic bath for 10 minutes.

2-{[4-Amino-6-(dimethylamino)-1,3,5-triazin-2-yl]methylthio}-4-chloro-N-(imidazolidin-2-ylidene)-5-methylbenzenesulfonamide (**20**).

Starting from 1,1-dimethylbiguanide hydrochloride (0.265 g, 1.60 mmol). The title compound was obtained. Yield 0.183 g (50%); m.p. 268–269 °C; IR (KBr): 3334, 3229, 3167 (N-H), 2973, 2921, 2855 (C-H), 1608, 1585, 1568, 1513 (C=N, $C=C_{Ar}$), 1285, 1159 (SO_2) cm^{-1}; ^1H NMR (500 MHz, DMSO-d_6) δ: 2.30 (s, 3H, CH_3Ph), 3.02 (s, 3H, N-CH_3), 3.08 (s, 3H, N-CH_3), 3.39-3.44 (m, 2H, imidazolidin), 3.88 (s, 2H, S-CH_2), 6.83-6.92 (m, 2H, NH_2), 7.44-7.51 (m, 2H, NH, imidazolidin), 7.83 (s, 1H, H-3), 7.93 (s, 1H, H-6); ^{13}C NMR (125 MHz, DMSO-d_6 in 35 ℃) δ: 19.59, 36.31, 36.41, 42.32, 128.27, 128.34, 131.00, 132.10, 136.97, 139.94, 161.18, 165.75, 167.46, 174.18 ppm; Anal. calcd. for $C_{16}H_{21}ClN_8O_2S_2$ (456.97); C, 42.05; H, 4.63; N, 24.52. Found: C, 42.00; H, 4.57; N, 24.48.

2-[(4-Amino-6-morpholino-1,3,5-triazin-2-yl)methylthio]-4-chloro-N-(imidazolidin-2-ylidene)-5-methylbenzenesulfonamide (**21**).

Starting from *N*-carbamimidoylmorpholine-4-carboximidamide hydrochloride (0.332 g, 1.60 mmol). The title compound was obtained. Yield 0.156 g (39%); m.p. 242–243 °C. IR (KBr): 3435, 3351 (N-H), 2914, 2859 (C-H), 1607, 1562, 1519 (C=N, C=C_{Ar}), 1281, 1155 (SO_2) cm^{-1}; ^1H NMR (500 MHz, DMSO-*d*$_6$) δ: 2.30 (s, 3H, CH_3Ph), 3.37-3.43 (m, 4H, imidazolidin), 3.54-3.60 (m, 4H, morpholine), 3.61-3.75 (m, 4H, morpholine), 3.89 (s, 2H, S-CH_2), 6.95-7.02 (m, 2H, NH_2), 7.45-7.50 (m, 2H, NH, imidazolidin), 7.83 (s, 1H, H-3), 7.90 (s, 1H, H-6) ppm; ^{13}C NMR (125 MHz, DMSO-d$_6$) δ: 19.58, 42.29, 43.73, 66.58, 128.24, 131.02, 132.30, 136.60, 137.05, 139.64, 161.08, 164.91, 167.51, 174.65 ppm; Anal. calcd. for $C_{18}H_{23}ClN_8O_3S_2$ (499.01); C, 43.32; H, 6.45; N, 22.46. Found: C, 43.30; H, 6.59; N, 22.42. IR and ^1H NMR data were in accordance with those reported previously to the authentic sample of **21** [31].

2-{[4-Amino-6-(3,5,5-trimethyl-4,5-dihydro-1H-pyrazol-1-yl)-1,3,5-triazin-2-yl]methylthio}-4-chloro-N-(imidazolidin-2-ylidene)-5-methylbenzenesulfonamide (**22**).

Starting from *N*-carbamimidoyl-3,5,5-trimethyl-4,5-dihydro-1*H*-pyrazole-1-carboximidamide hydrochloride (0.372 g, 1.60 mmol). The title compound was obtained after refluxing with acetonitrile (1:50) in an ultrasonic bath for 10 minutes. Yield 0.194 g (46%); m.p. 273–274 °C; IR (KBr): 3323, 3207 (N-H), 2978, 2938, 2909 (C-H), 1600, 1539, 1524 (C=N, C=C_{Ar}), 1284, 1179 (SO_2) cm^{-1}; ^1H NMR (500 MHz, DMSO-*d*$_6$) δ: 1.48-1.53 (m, 6H, CH_3, pyrazol), 1.94 (br.s, 3H, CH_3, pyrazol), 2.29 (s, 3H, CH_3Ph), 2.78 (s, 2H, CH_2, pyrazol), 3.39-3.42 (m, 4H, imidazolidin), 3.80-4.01 (m, 2H, S-CH_2), 6.93-7.84 (m, 2H, NH_2 and 2H, NH, imidazolidin and 1H, H-3 and 1H, H-6) ppm; ^{13}C NMR (125 MHz, DMSO-d$_6$) δ: 16.47, 19.64, 26.41, 26.81, 42.29, 53.39, 64.23, 79.77, 128.11, 131.09, 132.39, 136.64, 137.19, 139.99, 155.50, 161.13, 162.94, 167.46, 173.90 ppm; Anal. calcd. for $C_{20}H_{26}ClN_9O_2S_2$ (524.06); C, 45.84; H, 5.00; N, 24.05. Found: C, 45.81; H, 5.06; N, 24.01. IR and ^1H NMR data were in accordance with those reported previously to the authentic sample of **22** [31].

2-{[4-Amino-6-(indolin-1-yl)-1,3,5-triazin-2-yl]methylthio}-4-chloro-N-(imidazolidin-2-ylidene)-5-methylbenzenesulfonamide (**23**).

Starting from *N*-carbamimidoylindoline-1-carboximidamide hydrochloride (0.384 g, 1.60 mmol). The title compound was obtained. Yield 0.231 g (54%); m.p. 276–277 °C; IR (KBr): 3388, 3304, 3147 (N-H), 2969, 2951, 2929, 2853 (C-H), 1607, 1582, 1558, 1531 (C=N, C=C_{Ar}), 1277, 1156 (SO_2) cm^{-1}; ^1H NMR (500 MHz, DMSO-*d*$_6$) δ: 2.29 (s, 3H, CH_3), 3.10 (t, *J=8.6 Hz,* 2H, hydroindoline), 3.40-3.41 (m, 4H, imidazolidin), 3.98-4.11 (m, 2H, S-CH_2 and 2H, hydroindoline), 6.92-8.41 (m, 4H, H_{Ar} and 2H, NH_2 and 2H, NH, imidazolidin and 1H, H-3 and 1H, H-6) ppm; ^{13}C NMR (125 MHz, DMSO-d$_6$) δ: 19.64, 27.13, 42.30, 48.48, 53.08, 117.36, 122.85, 125.38, 127.61, 128.34, 131.11, 131.22, 132.31, 133.31, 136.63, 137.03, 143.18, 161.13, 163.41, 167.26, 170.30 ppm; Anal. calcd. for $C_{22}H_{23}ClN_8O_2S_2$ (531.05); C, 49.76; H, 4.37; N, 21.10. Found: C, 49.70; H, 4.34; N, 21.05.

2-{[4-Amino-6-(3,4-dihydroquinolin-1(2H)-yl)-1,3,5-triazin-2-yl]methylthio}-4-chloro-N-(imidazolidin-2-ylidene)-5-methylbenzenesulfonamide (**24**).

Starting from *N*-carbamimidoyl-3,4-dihydroquinoline-1(2*H*)-carboximidamide hydrochloride (0.406 g, 1.60 mmol). The title compound was obtained. Yield 0.127 g (31%); m.p. 253–254 °C; IR (KBr): 3393, 3305, 3174 (N-H), 2957, 2925, 2854 (C-H), 1603, 1583, 1560, 1523 (C=N, C=C_{Ar}), 1278, 1154 (SO_2) cm^{-1}; ^1H NMR (500 MHz, DMSO-*d*$_6$) δ: 1.85 (quintet, *J=6.5 Hz,* 2H, 1,2,3,4-tetrahydroquinoline), 2.31 (s, 3H, CH_3), 2.72 (t, *J=6.7 Hz,* 2H, 1,2,3,4-tetrahydroquinoline), 3.31-3.32 (m, 4H, imidazolidin), 3.87-3.98 (m, 2H, S-CH_2 and 2H, 1,2,3,4-tetrahydroquinoline), 6.96-7.71 (m, 4H, H_{Ar} and 2H, NH_2 and 2H, NH, imidazolidin), 7.74 (s, 1H, H-3), 7.84 (s, 1H, H-6) ppm; ^{13}C NMR (125 MHz, DMSO-d$_6$) δ: 19.64, 24.04, 27.22, 42.29, 44.51, 124.06, 125.72, 126.23, 128.29, 128.99, 131.06, 131.59, 132.25, 136.69, 137.05, 139.27, 140.01, 161.14, 165.10, 167.48, 174.67 ppm; Anal. calcd. for $C_{23}H_{25}ClN_8O_2S_2$ (545.08); C, 50.68; H, 4.62; N, 20.56. Found: C, 50.60; H, 4.59; N, 20.52.

2-{[4-Amino-6-(phenylamino)-1,3,5-triazin-2-yl]methylthio}-4-chloro-N-(imidazolidin-2-ylidene)-5-methylbenzenesulfonamide (**25**).

Starting from 1-phenylbiguanide hydrochloride (0.342 g, 1.60 mmol). The title compound was obtained after refluxing with acetonitrile (1:23) in an ultrasonic bath for 10 minutes. Yield 0.189 g (46%); m.p. 238–239 °C; IR (KBr): 3407, 3341, 3230 (N-H), 2983, 2917, 2895, 2832 (C-H), 1576, 1469 (C=N, C=C_{Ar}), 1280, 1153 (SO_2) cm^{-1}; ^1H NMR (500 MHz, DMSO-d_6) δ: 2.31 (s, 3H, CH_3Ph), 3.38-3.44 (m, 4H, imidazolidin), 3.98-4.02 (m, 2H, S-CH_2), 6.96-7.87 (m, 5H, H_{Ar} and 2H, NH_2 and 1H, H-3 and 1H, H-6 and 2H, NH, imidazolidin), 9.55-9.60 (m, 1H, NH) ppm; Anal. calcd. for $C_{20}H_{21}ClN_8O_2S_2$ (505.02); C, 47.57; H, 4.19; N, 22.19. Found: C, 47.56; H, 4.20; N, 22.15. HRMS (ESI-TOF) (504.0917) calcd for $C_{20}H_{21}ClN_8O_2S_2$ $[M+H]^+$ (505.0995) found 505.1001.

2-[{4-Amino-6-[(4-fluorophenyl)amino]-1,3,5-triazin-2-yl}methylthio]-4-chloro-N-(imidazolidin-2-ylidene)-5-methylbenzenesulfonamide (**26**).

Starting from 1-(4-fluorophenyl)biguanide hydrochloride (0.371 g, 1.60 mmol). The title compound was obtained after refluxing with ethanol (1:28) in an ultrasonic bath for 10 minutes. Yield 0.169 g (40%); m.p. 208–209 °C; IR (KBr): 3370, 3340 (N-H), 2983, 2915, 2833 (C-H), 1615, 1530 (C=N, C=C_{Ar}), 1285, 1154 (SO_2) cm^{-1}; ^1H NMR (500 MHz, DMSO-d_6) δ: 2.31 (s, 3H, CH_3Ph), 3.39-3.42 (m, 4H, imimidazolidin), 3.99 (s, 2H, S-CH_2), 7.08-7.76 (m, 4H, H_{Ar} and 2H, NH_2 and 2H, NH, imidazolidin and 1H, H-3), 7.87 (s, 1H, H-6), 9.49-9.71 (m, 1H, NH) ppm; Anal. calcd. for $C_{20}H_{20}ClFN_8O_2S_2$ (523.01); C, 45.93; H, 3.85; N, 21.42. Found: C, 45.94; H, 3.85; N, 21.42. HRMS (ESI-TOF) (522.0823) calcd for $C_{20}H_{20}ClFN_8O_2S_2$ $[M+H]^+$ (523.0901) found 523.0923.

2-{[4-Amino-6-(benzylamino)-1,3,5-triazin-2-yl]methylthio}-4-chloro-N-(imidazolidin-2-ylidene)-5-methylbenzenesulfonamide (**27**).

Starting from 2-benzyl-1-(diaminomethylidene)guanidine hydrochloride (0.364 g, 1.60 mmol). The title compound was obtained after refluxing with acetonitrile (1:21) in an ultrasonic bath for 10 minutes and next solid washed with 1 mL hot ethanol. Yield 0.281 g (68%); m.p. 242–243 °C; IR (KBr): 3316, 3179 (N-H), 2970, 2942, 2923, 2874 (C-H), 1573, 1564, 1542, 1521 (C=C and C=N), 1278, 1153 (SO_2) cm^{-1}; ^1H NMR (500 MHz, DMSO-d_6 in T=100°C) δ: 2.33 (s, 3H, CH_3), 3.45 (s, 4H, imidazolidin), 3.89 (s, 2H, S-CH_2), 4.51 (dd, 2H, $\mathbf{CH_2}Ph$), 6.48-6.52 (m, 2H, NH_2), 7.20-7.40 (m, 5H, H_{Ar} and 2H, NH, imidazolidin and 1H, NH), 7.70 (s, 1H, H-3), 7.84 (s, 1H, H-6) ppm; Anal. calcd. for $C_{38}H_{39}ClFN_9O_2S_2$ (519.04); C, 48.59; H, 4.47; N, 21.59. Found: C, 48.36; H, 4.40; N, 21.58. HRMS (ESI-TOF) (518.1074) calcd for $C_{21}H_{23}ClN_8O_2S_2$ $[M+H]^+$ (519.1152) found 519.1150.

2-[{4-Amino-6-[methyl(phenyl)amino]-1,3,5-triazin-2-yl}methylthio]-4-chloro-N-(imidazolidin-2-ylidene)-5-methylbenzenesulfonamide (**28**).

Starting from 1-methyl-1-phenylbiguanide hydrochloride (0.364 g, 1.60 mmol). The title compound was obtained. Yield 0.196 g (47%); m.p. 231–232 °C; IR (KBr): 3386, 3301, 3138 (N-H), 2966, 2953, 2923, 2855 (C-H), 1605, 1585, 1528, 1517 (C=N, C=C_{Ar}), 1278, 1155 (SO_2) cm^{-1}; ^1H NMR (500 MHz, DMSO-d_6) δ: 2.30 (s, 3H, CH_3Ph), 3.39-3.40 (m, 3H, N-$\mathbf{CH_3}$), 3.40-3.42 (m, 4H, imidazolidin), 3.89 (s, 2H, S-CH_2), 6.92-7.05 (m, 2H, NH_2), 7.19-7.47 (m, 5H, H_{Ar} and 2H, NH, imidazolidin), 7.77 (s, 1H, H-3), 7.84 (s, 1H, H-6); ^{13}C NMR (125 MHz, DMSO-d_6) δ: 24.40, 43.29, 44.82, 47.06, 131.21, 132.20, 132.90, 134.24, 135.80, 136.94, 141.57, 141.85, 144.59, 149.66, 165.89, 170.61, 172.11, 179.26 ppm; Anal. calcd. for $C_{21}H_{23}ClN_8O_2S_2$ (519.04); C, 48.59; H, 4.47; N, 21.59. Found: C, 48.50; H, 4.42; N, 21.53.

2-[{4-Amino-6-[(4-chlorophenyl)(methyl)amino]-1,3,5-triazin-2-yl}methylthio]-4-chloro-N-(imidazolidin-2-ylidene)-5-methylbenzenesulfonamide (**29**).

Starting from 1-(4-chlorophenyl)-1-methylbiguanide hydrochloride (0.419 g, 1.60 mmol). The title compound was obtained after refluxing with acetonitrile (1:50) in an ultrasonic bath for 10 minutes.

Yield 0.112 g (25%); m.p. 237–238 °C; IR (KBr): 3390, 3335, 3211 (N-H), 2942, 2924, 2894, 2855 (C-H), 1562, 1541, 1523, 1490 (C=N, C=C_{Ar}), 1285, 1150 (SO_2) cm^{-1}; ^1H NMR (500 MHz, DMSO-d_6) δ: 2.30 (s, 3H, CH_3Ph), 3.36-3.39 (m, 3H, N-**CH_3**), 3.40-3.44 (m, 4H, imidazolidin), 3.89 (s, 2H, S-CH_2), 7.02-7.06 (m, 2H, NH_2), 7.32-7.47 (m, 4H, H_{Ar} and 2H, NH, imidazolidin), 7.74 (s, 1H, H-3), 7.84 (s, 1H, H-6) ppm; ^{13}C NMR (125 MHz, DMSO-d_6) δ: 19.65, 38.16, 42.31, 128.12, 129.19, 129.27, 130.47, 131.01, 132.17, 136.72, 137.07, 139.79, 143.68, 161.13, 165.78, 167.35, 174.65 ppm; Anal. calcd. for $C_{21}H_{22}Cl_2N_8O_2S_2$ (553.49); C, 45.57; H, 4.01; N, 20.24. Found: C, 45.55; H, 3.96; N, 20.20.

2-{[4-Amino-6-(4-methylopiperazyn-1-yl)-1,3,5-triazyn-2-yl]methylthio}-4-chloro-N-(imidazolidin-2-ylidene)-5-methylobenzenosulfonamid (**30**).

Starting from *N*-carbamimidoyl-4-methylpiperazine-1-carboximidamide hydrochloride (0.353 g, 1.60 mmol). The title compound was obtained. Yield 0.080 g (20%); m.p. 138–139 °C. IR (KBr): 3400, 3324, 3215 (N-H), 2978, 2918, 2873, 2815 (C-H), 1577, 1462 (C=N, C=C_{Ar}), 1279, 1150 (SO_2) cm^{-1}; ^1H NMR (500 MHz, DMSO-d_6) δ: 2.19 (s, 3H, **CH_3**-piperazine), 2.29-2.31 (m, 3H, CH_3Ph and 4H, piperazine), 3.33-3.34 (m, 4H, piperazine), 3.70-7.72 (m, 4H, imimidazolidin), 3.89 (s, 2H, S-CH_2), 6.91-6.98 (m, 2H, NH_2), 7.47-7.49 (m, 2H, NH, imidazolidin), 7.84 (s, 1H, H-3), 7.93 (s, 1H, H-6) ppm; Anal. calcd. for $C_{19}H_{26}ClN_9O_2S_2$ (512.08); C, 44.57; H, 5.12; N, 24.62. Found: C, 44.45; H, 5.20; N, 24.43. HRMS (ESI-TOF) (511.1339) calcd for $C_{19}H_{26}ClN_9O_2S_2$ $[M+H]^+$ (512.1417) found 512.1436.

2-{[4-Amino-6-(4-phenylpiperazin-1-yl)-1,3,5-triazin-2-yl]methylthio}-4-chloro-N-(imidazolidin-2-ylidene)-5-methylbenzenesulfonamide (**31**).

Starting from *N*-carbamimidoyl-4-phenylpiperazine-1-carboximidamide hydrochloride (0.452 g, 1.60 mmol). The title compound was obtained after refluxing with acetonitrile (1:25) in an ultrasonic bath for 10 minutes and next all was washed 1.5 mL ethanol. Yield 0.215 g (53%); m.p. 221–223 °C; IR (KBr): 3401, 3362, 3320 (N-H), 2935, 2893, 2863, 2815 (C-H), 1562, 1522 (C=N, C=C_{Ar}), 1289, 1149 (SO_2) cm^{-1}; ^1H NMR (500 MHz, DMSO-d_6) δ: 2.30 (s, 3H, CH_3), 3.05-3.21 (m, 4H, piperazine), 3.39-3.42 (m, 4H, imidazolidin), 3.77-3.90 (m, 4H, piperazine), 3.91 (s, 2H, S-CH_2), 6.78-7.48 (m, 5H, H_{Ar} and 2H, NH_2 and 2H, NH, imidazolidin), 7.83 (s, 1H, H-3), 7.92 (s, 1H, H-6) ppm; ^{13}C NMR (125 MHz, DMSO-d_6) δ: 19.59, 39.98, 40.15, 40.31, 40.48, 42.30, 49.03, 116.66, 120.14, 128.29, 129.71, 131.04, 132.30, 136.62, 137.09, 139.67, 151.58, 161.10, 164.81, 167.55, 174.68 ppm; Anal. calcd. for $C_{24}H_{28}ClN_9O_2S_2$ (574.12); C, 50.21; H, 4.92; N, 21.96. Found: C, 50.16; H, 5.00; N, 21.87.

2-[{4-Amino-6-[4-(4-fluorophenyl)piperazin-1-yl]-1,3,5-triazin-2-yl}methylthio]-4-chloro-N-(imidazolidin-2-ylidene)-5-methylbenzenesulfonamide (**32**).

Starting from *N*-carbamimidoyl-4-(4-fluorophenyl)piperazine-1-carboximidamide hydrochloride (0.481 g, 1.60 mmol). The title compound was obtained Yield 0.121 g (25.5%); m.p. 236–237 °C; IR (KBr): 3407, 3367, 3306 (N-H), 2986, 2897, 2862, 2812 (C-H), 1561, 1488 (C=N, C=C_{Ar}), 1288, 1149 (SO_2) cm^{-1}; ^1H NMR (500 MHz, DMSO-d_6) δ: 2.31 (s, 3H, CH_3Ph), 3.06-3.10 (m, 4H, imidazolidin), 3.41-3.43 (m, 4H, piperazine), 3.80-3.90 (m, 4H, piperazine), 3.92 (s, 2H, S-CH_2), 6.98-7.09 (m, 4H, H_{Ar} and 2H, NH_2), 7.47-7.49 (m, 2H, NH, imidazolidin), 7.85 (s, 1H, H-3), 7.93 (s, 1H, H-6) ppm; Anal. calcd. for $C_{24}H_{27}ClFN_9O_2S_2$ (592.11); C, 48.68; H, 4.60; N, 21.29. Found: C, 48.53; H, 4.50; N, 21.09. HRMS (ESI-TOF) (591.1402) calcd for $C_{24}H_{27}ClFN_9O_2S_2$ $[M+H]^+$ (592.1480) found. 592.1440.

2-[{4-Amino-6-[4-(2-fluorophenyl)piperazin-1-yl]-1,3,5-triazin-2-yl}methylthio]-4-chloro-N-(imidazolidin-2-ylidene)-5-methylbenzenesulfonamide (**33**).

Starting from *N*-carbamimidoyl-4-(2-fluorophenyl)piperazine-1-carboximidamide hydrochloride (0.481 g, 1.60 mmol). The title compound was obtained by crystallization from ethanol (1:33), remained part by crystallization from filtrate. Yield 0.097 g (20%); m.p. 222–224 °C with; IR (KBr): 3415, 3334, 3238 (N-H), 2919, 2857 (C-H), 1557, 1449 (C=N, C=C_{Ar}), 1238, 1152 (SO_2) cm^{-1}; ^1H NMR (500 MHz, DMSO-d_6) δ: 2.31 (s, 3H, CH_3Ph), 2.97-3.04 (m, 4H, imidazolidin), 3.40-3.43 (m, 4H, piperazine), 3.55-3.92 (m, 4H piperazine and 2H, S-CH_2), 7.01-7.16 (m, 4H, H_{Ar} and 2H, NH_2), 7.47-7.50 (m, 2H, NH, imidazolidin),

7.85 (s, 1H, H-3), 7.92 (s, 1H, H-6) ppm; Anal. calcd. for $C_{24}H_{27}ClFN_9O_2S_2$ (592.11); C, 48.68; H, 4.60; N, 21.29. Found: C, 48.66; H, 4.53; N, 21.23. HRMS (ESI-TOF) (591.1402) calcd for $C_{24}H_{27}ClFN_9O_2S_2$ $[M+H]^+$ (592.1480) found 592.1462.

2-{[4-Amino-6-{4-[4-(trifluoromethyl)phenyl]piperazin-1-yl}-1,3,5-triazin-2-yl]methylthio}-4-chloro-N-(imidazolidin-2-ylidene)-5-methylbenzenesulfonamide (**34**).

Starting from *N*-carbamimidoyl-4-[4-(trifluoromethyl)phenyl]piperazine-1-carboximidamide hydrochloride (0.561 g, 1.60 mmol). The title compound was obtained after refluxing with acetonitrile (1:21) in an ultrasonic bath for 10 minutes. Yield 0.224 g (44%); m.p. 221–222 °C; IR (KBr): 3412, 3307, 3183 (N-H), 2985, 2895, 2856 (C-H), 1569, 1445 (C=N, $C=C_{Ar}$), 1386, 1157 (SO_2) cm^{-1}; ^1H NMR (500 MHz, DMSO-d_6) δ: 2.31 (s, 3H, CH_3Ph), 3.33-3.35 (m, 4H, piperazine), 3.41-3.43 (m, 4H, imidazolidin), 3.81-3.90 (m, 4H, piperazine), 3.92 (s, 2H, S-CH_2), 6.96-7.04 (m, 2H, NH_2), 7.10-7.12 and 7.52-7.54 (m, 4H, H_{Ar}), 7.47-7.50 (m, 2H, NH, imidazolidin), 7.88 (s, 1H, H-3), 7.93 (s, 1H, H-6) ppm; Anal. calcd. for $C_{25}H_{27}ClF_3N_9O_2S_2$ (642.12); C, 46.76; H, 4.24; N, 19.63. Found: C, 46.62; H, 4.16; N, 19.58. HRMS (ESI-TOF) (641.1370) calcd for $C_{25}H_{27}ClF_3N_9O_2S_2$ $[M+H]^+$ 642.1448 found 642.1466.

2-{[4-Amino-6-{4-[3-(trifluoromethyl)phenyl]piperazin-1-yl}-1,3,5-triazin-2-yl]methylthio}-4-chloro-N-(imidazolidin-2-ylidene)-5-methylbenzenesulfonamide (**35**).

Starting from *N*-carbamimidoyl-4-[3-(trifluoromethyl)phenyl]piperazine-1-carboximidamide hydrochloride (0.561 g, 1.60 mmol). The title compound was obtained after refluxing with ethanol (1:21) in an ultrasonic bath for 10 minutes. Yield 0.278 g (54%); m.p. 243–244 °C; IR (KBr): 3318, 3227 (N-H), 2986, 2917, 2894, 2851 (C-H), 1572, 1546, 1508, 1475 (C=N, $C=C_{Ar}$), 1286, 1159 (SO_2) cm^{-1}; ^1H NMR (500 MHz, DMSO-d_6) δ: 2.31 (s, 3H, CH_3Ph), 3.26-3.28 (m, 4H, piperazine), 3.41-3.43 (m, 4H, imidazolidin), 3.81-3.91 (m, 4H, piperazine), 3.92 (s, 2H, S-CH_2), 7.00-7.49 (m, 4H, H_{Ar} and 2H, NH_2 and 2H, NH, imidazolidin), 7.85 (s, 1H, H-3), 7.93 (s, 1H, H-6) ppm; Anal. calcd. for $C_{25}H_{27}ClF_3N_9O_2S_2$ (642.12); C, 46.76; H, 4.24; N, 19.63. Found: C, 46.51; H, 4.14; N, 19.38; MALDI-TOF/TOF (641.1370) calcd for $C_{25}H_{27}ClF_3N_9O_2S_2$ $[M+H]^+$ 642.1448 found 642.1610.

2-[{4-Amino-6-[4-(4-chlorophenyl)piperazin-1-yl]-1,3,5-triazin-2-yl}methylthio]-4-chloro-N-(imidazolidin-2-ylidene)-5-methylbenzenesulfonamide (**36**).

Starting from *N*-carbamimidoyl-4-(4-chlorophenyl)piperazine-1-carboximidamide hydrochloride (0.508 g, 1.60 mmol). The title compound was obtained after refluxing with acetonitrile (1:50) in an ultrasonic bath for 10 minutes. Yield 0.163 g (33%); m.p. 217–218 °C; IR (KBr): 3403, 3305, 3175 (N-H), 2983, 2921, 2853 (C-H), 1565, 1543, 1519 (C=N, $C=C_{Ar}$), 1283, 1154 (SO_2) cm^{-1}; ^1H NMR (500 MHz, DMSO-d_6) δ: 2.30 (s, 3H, CH_3), 3.11-3.17 (m, 4H, piperazine), 3.37-3.43 (m, 4H, imidazolidin), 3.77-3.93 (m, 4H, piperazine), 3.91 (s, 2H, S-CH_2), 6.97-7.48 (m, 4H, H_{Ar} and 2H, NH_2 and 2H, NH, imidazolidin), 7.84 (s, 1H, H-3), 7.92 (s, 1H, H-6) ppm; ^{13}C NMR (125 MHz, DMSO-d_6) δ: 19.63, 42.31, 43.37, 48.79, 118.02, 123.46, 128.20, 129.36, 131.06, 132.13, 136.81, 137.02, 139.75, 150.42, 161.11, 164.89, 167.61, 174.68 ppm; Anal. calcd. for $C_{24}H_{27}Cl_2N_9O_4S_2$ (608.57); C, 47.37; H, 4.47; N, 20.71. Found: C, 47.30; H, 4.44; N, 20.62.

2-[{4-Amino-6-[4-(3-chlorophenyl)piperazin-1-yl]-1,3,5-triazin-2-yl}methylthio]-4-chloro-N-(imidazolidin-2-ylidene)-5-methylbenzenesulfonamide (**37**).

Starting from *N*-carbamimidoyl-4-(3-chlorophenyl)piperazine-1-carboximidamide hydrochloride (0.508 g, 1.60 mmol). The title compound was obtained after refluxing with ethanol (1:24) in an ultrasonic bath for 10 minutes. Yield 0.310 g (64%); m.p. 248–249 °C; IR (KBr): 3318, 3207, 3182 (N-H), 2981, 2921, 2894, 2846 (C-H), 1598, 1571, 1545, 1508 (C=N, $C=C_{Ar}$), 1289, 1158 (SO_2) cm^{-1}; ^1H NMR (500 MHz, DMSO-d_6) δ: 2.31 (s, 3H, CH_3Ph), 3.19-3.23 (m, 4H, piperazine), 3.41-3.43 (m, 4H, imidazolidin), 3.77-3.90 (m, 4H, piperazine), 3.92 (s, 2H, S-CH_2), 6.81-7.48 (m, 4H, H_{Ar} and 2H, NH_2 and 2H, NH, imidazolidin), 7.85 (s, 1H, H-3), 7.93 (s, 1H, H-6) ppm; Anal. calcd. for $C_{24}H_{27}Cl_2N_9O_2S_2$ (608.57); C, 47.37;

H, 4.47; N, 20.71. Found: C, 46.98; H, 4.24; N, 20.49; MALDI-TOF/TOF (607.1106) calcd for $C_{24}H_{27}Cl_2N_9O_2S_2$ $[M+H]^+$ (608.1184) found 608.0501.

2-[{4-Amino-6-[4-(3,4-dichlorophenyl)piperazin-1-yl]-1,3,5-triazin-2-yl}methylthio]-4-chloro-N-(1-imidazolidin-2-ylidene)-5-methylbenzenesulfonamide (**38**).

Starting from *N*-carbamimidoyl-4-(3,4-dichlorophenyl)piperazine-1-carboximidamide hydrochloride (0.563 g, 1.60 mmol). The title compound was obtained after refluxing with acetonitrile (1:20) in an ultrasonic bath for 10 minutes. Yield 0.150 g (29%); m.p. 245–246 °C (dec.); IR (KBr): 3392, 3362, 3221 (N-H), 2989, 2903, 2863, 2829 (C-H), 1519, 1487 (C=N, $C=C_{Ar}$), 1272, 1145 (SO_2) cm^{-1}; ^1H NMR (500 MHz, DMSO-d_6) δ: 2.31 (s, 3H, CH_3Ph), 3.21-3.24 (m, 4H, imidazolidin), 3.41-3.43 (m, 4H, piperazine), 3.78-3.90 (m, 4H, piperazine), 3.92 (s, 2H, S-CH_2), 6.97-7.43 (m, 3H, H_{Ar} and 2H, NH_2), 7.45-7.50 (m, 2H, NH, imidazolidin), 7.85 (s, 1H, H-3), 7.93 (s, 1H, H-6) ppm; Anal. calcd. for $C_{24}H_{26}Cl_3N_9O_2S_2$ (643.01); C, 44.83; H, 4.08; N, 19.60. Found: C, 44.63; H, 4.00; N, 19.10. HRMS (ESI-TOF) (641.0716) calcd for $C_{24}H_{26}Cl_3N_9O_2S_2$ $[M+H]^+$ (642.0794) found 642.0800.

2-[{4-Amino-6-[4-(3-chloro-4-fluorophenyl)piperazin-1-yl]-1,3,5-triazin-2-yl}methylthio]-4-chloro-N-(imidazolidin-2-ylidene)-5-methylbenzenesulfonamide (**39**).

Starting from *N*-carbamimidoyl-4-(3-chloro-4-fluorophenyl)piperazine-1-carboximidamide hydrochloride (0.536 g, 1.60 mmol). The title compound was obtained after refluxing with ethanol (1:33) in an ultrasonic bath for 10 minutes. Yield 0.265 g (53%); m.p. 229–231 °C; IR (KBr): 3317, 3204 (N-H), 2973, 2921, 2896, 2845 (C-H), 1569, 1541, 1504, 1474 (C=N, $C=C_{Ar}$), 1281, 1158 (SO_2) cm^{-1}; ^1H NMR (500 MHz, DMSO-d_6) δ: 2.31 (s, 3H, CH_3Ph), 3.14-3.16 (m, 4H, piperazine), 3.41-3.43 (m, 4H, imidazolidin), 3.78-3.90 (m, 4H, piperazine), 3.92 (s, 2H, S-CH_2), 6.97-7.49 (m, 3H, H_{Ar} and 2H, NH_2 and 2H, NH, imidazolidin), 7.85 (s, 1H, H-3), 7.93 (s, 1H, H-6) ppm; Anal. calcd. for $C_{24}H_{26}Cl_2FN_9O_2S_2$ (626.56); C, 46.01; H, 4.18; N, 20.12. Found: C, 45.85; H, 4.07; N, 19.94; MALDI-TOF/TOF (625.1012) calcd for $C_{24}H_{26}Cl_2FN_9O_2S_2$ $[M+H]^+$ (626.1090) found 626.0480.

2-[{4-amino-6-[4-(4-methoxyphenyl)piperazin-1-yl]-1,3,5-triazin-2-yl}methylthio]-4-chloro-N-(imidazolidin-2-ylidene)-5-methylbenzenesulfonamide (**40**).

Starting from *N*-carbamimidoyl-4-(4-methoxyphenyl)piperazine-1-carboximidamide hydrochloride (0.500 g, 1.60 mmol). The title compound was obtained after refluxing with mixture 1:1 ethanol/acetonitrile (1:28) in an ultrasonic bath for 10 minutes. Yield 0.252 g (52%); m.p. 185–186 °C; IR (KBr): 3323, 3222, 3152 (N-H), 2963, 2916, 2829, 2820 (C-H), 1608, 1569, 1511, 1473 (C=N, $C=C_{Ar}$), 1249, 1152 (SO_2) cm^{-1}; ^1H NMR (500 MHz, DMSO-d_6) δ: 2.31 (s, 3H, CH_3Ph), 2.99-3.01 (m, 4H, piperazine), 3.41-3.43 (m, 4H, imidazolidin), 3.69 (s, 3H, O-**C**$\mathbf{H_3}$), 3.79-3.90 (m, 4H, piperazine), 3.92 (s, 2H, S-CH_2), 6.83-7.48 (m, 4H, H_{Ar} and 2H, NH_2 and 2H, NH, imidazolidin), 7.85 (s, 1H, H-3), 7.93 (s, 1H, H-6) ppm; Anal. calcd. for $C_{25}H_{30}ClN_9O_3S_2$ (604.15); C, 49.70; H, 5.01; N, 20.87. Found: C, 49.19; H, 4.94; N, 20.32; MALDI-TOF/TOF (603.1602) calcd for $C_{25}H_{30}ClN_9O_3S_2$ $[M+H]^+$ (604.1680) found 604.1605.

2-[{4-Amino-6-[4-(2-methoxyphenyl)piperazin-1-yl]-1,3,5-triazin-2-yl}methylthio]-4-chloro-N-(imidazolidin-2-ylidene)-5-methylbenzenesulfonamide (**41**).

Starting from *N*-carbamimidoyl-4-(2-methoxyphenyl)piperazine-1-carboximidamide hydrochloride (0.500 g, 1.60 mmol). The title compound was obtained after refluxing with acetonitrile (1:22) in an ultrasonic bath for 10 minutes. Yield 0.241 g (50%); m.p. 135–136 °C; IR (KBr): 3378, 3220 (N-H), 2944, 2904, 2811 (C-H), 1557, 1445 (C=N, $C=C_{Ar}$), 1287, 1153 (SO_2) cm^{-1}; ^1H NMR (500 MHz, DMSO-d_6) δ: 2.31 (s, 3H, CH_3Ph), 2.94-2.96 (m, 4H, piperazine), 3.41-3.43 (m, 4H, imidazolidin), 3.80 (s, 3H, O-**C**$\mathbf{H_3}$), 3.83-3.87 (m, 4H, piperazine), 3.92 (s, 2H, S-**C**$\mathbf{H_2}$), 6.86-7.00 (m, 4H, H_{Ar} and 2H, NH_2), 7.47-7.48 (m, 2H, NH, imidazolidin), 7.85 (s, 1H, H-3), 7.92 (s, 1H, H-6) ppm; Anal. calcd. for $C_{25}H_{30}ClN_9O_3S_2$ (604.15); C, 49.70; H, 5.01; N, 20.87. Found: C, 49.56; H, 4.96; N, 20.78. HRMS (ESI-TOF) (603.1602) calcd for $C_{25}H_{30}ClN_9O_3S_2$ $[M+H]^+$ (604.1680) found 604.1674.

2-{[4-Amino-6-(4-benzylpiperazin-1-yl)-1,3,5-triazin-2-yl]methylthio}-4-chloro-N-(imidazolidin-2-ylidene)-5-methylbenzenesulfonamide (**42**).

Starting from 4-benzyl-*N*-carbamimidoylpiperazine-1-carboximidamide hydrochloride (0.475 g, 1.60 mmol). The title compound was obtained after refluxing with mixture 1:1 ethanol/acetonitrile (1:20) in an ultrasonic bath for 10 minutes. Yield 0.248 g (53%); m.p. 204–205 °C; IR (KBr): 3320, 3233, 3149 (N-H), 2981, 2941, 2888, 2831 (C-H), 1576, 1506, 1472 (C=N, C=C_{Ar}), 1284, 1122 (SO_2) cm^{-1}; ^{1}H NMR (500 MHz, DMSO-d_6) δ: 2.30 (s, 3H, CH_3Ph), 2.35-2.37 (m, 4H, piperazine), 3.40-3.43 (m, 4H, imidazolidin), 3.49 (s, 2H, CH_2Ph), 3.69-3.74 (m, 4H, piperazine), 3.89 (s, 2H, S-CH_2), 6.93-7.48 (m, 5H, H_{Ar} and 2H, NH_2 and 2H, NH, imidazolidin), 7.84 (s, 1H, H-3), 7.91 (s, 1H, H-6) ppm; Anal. calcd. for $C_{25}H_{30}ClN_9O_2S_2$ (588.15); C, 51.05; H, 5.14; N, 21.43. Found: C, 50.90; H, 4.96; N, 21.33; MALDI-TOF/TOF (587.1652) calcd for $C_{25}H_{30}ClN_9O_2S_2$ $[M+H]^+$ (588.1730) found 588.0512.

2-{[4-Amino-6-(4-benzylpiperidin-1-yl)-1,3,5-triazin-2-yl]methylthio}-4-chloro-N-(imidazolidin-2-ylidene)-5-methylbenzenesulfonamide (**43**).

Starting from 4-benzyl-*N*-carbamimidoylpiperidine-1-carboximidamide hydrochlorid (0.473 g, 1.60 mmol). The title compound was obtained after refluxing with ethanol (1:35) in an ultrasonic bath for 10 minutes. Yield 0.269 g (57%); m.p. 221–222 °C; IR (KBr): 3319, 3227 (N-H), 2929, 2911, 2859, 2843 (C-H), 1604, 1542, 1474 (C=N, C=C_{Ar}), 1267, 1153 (SO_2) cm^{-1}; ^{1}H NMR (500 MHz, DMSO-d_6) δ: 1.00-1.07 (m, 2H, piperidine C3 and C5), 1.58-1.60 (m, 2H, piperidine C3 and C5), 1.77-1.80 (m, 1H, piperidine C4), 2.30 (s, 3H, CH_3Ph), 2.50-2.53 (m, 2H, piperidine-C**H**$_2$Ph), 2.72-2.77 (m, 2H, piperidine C2 and C6), 3.41-3.43 (m, 4H, imidazolidin), 3.88 (s, 2H, S-CH_2), 4.59-4.66 (m, 2H, piperidine C2 and C6), 6.88-7.48 (m, 5H, H_{Ar} and 2H, NH, imidazolidin and 2H, NH_2), 7.84 (s, 1H, H-3), 7.92 (s, 1H, H-6) ppm; Anal. calcd. for $C_{26}H_{31}ClN_8O_2S_2$ (587.16); C, 53.18; H, 5.32; N, 19.08. Found: C, 52.92; H, 5.23; N, 18.79; MALDI-TOF/TOF (586.1700) calcd for $C_{26}H_{31}ClN_8O_2S_2$ $[M+H]^+$ (587.1778) found 587.0540.

2-[{4-Amino-6-[4-(4-fluorophenyl)-5,6-dihydropyridin-1(2H)-yl]-1,3,5-triazin-2-yl}methylthio]-4-chloro-N-(imidazolidin-2-ylidene)-5-methylbenzenesulfonamide (**44**).

Starting from *N*-carbamimidoyl-4-(4-fluorophenyl)-5,6-dihydropyridine-1(2*H*)-carboximidamide hydrochloride (0.476 g, 1.60 mmol). The title compound was obtained after refluxing with mixture 1:1 ethanol/acetonitrile (1:21) in an ultrasonic bath for 10 minutes. Yield 0.241 g (51%); m.p. 237–238 °C; IR (KBr): 3370, 3221 (N-H), 2967, 2904 (C-H), 1594, 1574, 1553, 1490 (C=N, C=C_{Ar}), 1273, 1151 (SO_2) cm^{-1}; ^{1}H NMR (500 MHz, DMSO-d_6 in 70°C) δ: 2.33 (s, 3H, CH_3Ph), 3.44-3.46 (m, 4H, imidazolidin and 2H, $CH2_{C5}$), 3.45 (t, *J=8.80 Hz*, 2H, imidazolidin), 3.93 (s, 2H, S-CH_2), 3.95-3.97 (m, 2H, $CH2_{C2}$), 4.31-4.35 (m, 2H, $CH2_{C6}$), 6.17-6.20 (m, 1H, CH_{C3}), 6.73-6.77 (m, 2H, NH_2), 7.14-7.17 (m, 2H, NH, imidazolidin), 7.34-7.49 (m, 4H, H_{Ar}), 7.84 (s, 1H, H-3), 7.90 (s, 1H, H-6) ppm; Anal. calcd. for $C_{25}H_{26}ClFN_8O_2S_2$ (589.11); C, 50.97; H, 4.45; N, 19.02. Found: C, 50.67; H, 4.31; N, 18.75; MALDI-TOF/TOF (588.1293) calcd for $C_{25}H_{26}ClFN_8O_2S_2$ $[M+H]^+$ (589.1371) found 589.0808.

2-{[4-Amino-6-(dimethylamino)-1,3,5-triazin-2-yl]methylthio}-N-(1-benzylimidazolidin-2-ylidene)-4-chloro-5-methylbenzenesulfonamide (**45**).

Starting from 1,1-dimethylbiguanide hydrochloride (0.265 g, 1.60 mmol). The title compound was obtained. Yield 0.110 g (25%); m.p. 201–202 °C; IR (KBr): 3393, 3169 (N-H), 2977, 2920, 2866 (C-H), 1585, 1567, 1518 (C=N, C=C_{Ar}), 1266, 1129 (SO_2) cm^{-1}; ^{1}H NMR (500 MHz, DMSO-d_6) δ: 2.29 (s, 3H, CH_3Ph), 2.99 (s, 3H, N-C**H**$_3$), 3.02 (s, 3H, N-C**H**$_3$), 3.26 (t, *J=8.8 Hz*, 2H, imidazolidin), 3.41 (t, *J=8.7 Hz*, 2H, imidazolidin), 3.89 (s, 2H, S-CH_2), 4.34 (s, 2H, C**H**$_2$-C_6H_5), 6.83-6.90 (m, 2H, NH_2), 7.18-7.39 (m, 5H, H_{Ar} and 1H, NH, imidazolidin), 7.83 (s, 1H, H-3), 7.95 (s, 1H, H-6) ppm; ^{13}C NMR (125 MHz, DMSO-d_6) δ: 19.63, 36.24, 36.37, 41.48, 44.93, 47.77, 128.10, 128.30, 128.52, 129.17, 130.97, 132.05, 136.92, 137.07, 137.19, 139.47, 158.81, 165.66, 167.41, 174.11 ppm; Anal. calcd. for $C_{23}H_{27}ClN_8O_2S_2$ (547.10); C, 50.49; H, 4.97; N, 20.48. Found: C, 50.40; H, 4.95; N, 20.23.

2-[(4-Amino-6-morpholino-1,3,5-triazin-2-yl)methylthio]-N-(1-benzylimidazolidin-2-ylidene)-4-chloro-5-methylbenzenesulfonamide (**46**).

Starting from *N*-carbamimidoylmorpholine-4-carboximidamide hydrochloride (0.332 g, 1.60 mmol). The title compound was obtained. Yield 0.081 g (17%); m.p. 200–201 °C. IR (KBr): 3346, 3162 (N-H), 2960, 2920, 2898, 2853 (C-H), 1598, 1584, 1569, 1528 (C=N, C=C$_{Ar}$), 1287, 1132 (SO_2) cm^{-1}; ^{1}H NMR (500 MHz, DMSO-*d*$_6$) δ: 2.30 (s, 3H, CH_3), 3.27 (t, *J=8.55 Hz,* 2H, imidazolidin), 3.42 (t, *J=8.8 Hz,* 2H, imidazolidin), 3.49-3.57 (m, 4H, morpholine), 3.62-3.65 (m, 4H, morpholine), 3.92 (s, 2H, S-CH_2), 4.35 (s, 2H, C**H**$_2$-C_6H_5), 6.95-7.02 (m, 2H, NH_2), 7.19-7.40 (m, 5H, H$_{Ar}$ and 1H, NH, imidazolidin), 7.84 (s, 1H, H-3), 7.93 (s, 1H, H-6) ppm; ^{13}C NMR (125 MHz, DMSO-d$_6$) δ: 18.98, 40.82, 44.28, 47.10, 65.90, 127.48, 127.76, 127.91, 128.54, 130.33, 131.51, 136.21, 136.27, 136.50, 138.90, 158.14, 164.35, 166.91, 173.88 ppm; Anal. calcd. for $C_{25}H_{29}ClN_8O_3S_2$ (589.13); C, 50.97; H, 4.96; N, 19.02. Found: C, 50.89; H, 4.78; N, 18.95.

2-{[4-Amino-6-(3,5,5-trimethyl-4,5-dihydro-1H-pyrazol-1-yl)-1,3,5-triazin-2-yl]methylthio}-N-(1-benzylimidazolidin-2-ylidene)-4-chloro-5-methylbenzenesulfonamide (**47**).

Starting from *N*-carbamimidoyl-3,5,5-trimethyl-4,5-dihydro-1*H*-pyrazole-1-carboximidamide hydrochloride (0.372 g, 1.60 mmol). After evaporation of the reaction mixture to dryness, the compound was dissolved in 8 mL mixture of benzene and ethanol (8:1). Separation on a chromatographic column was carried out using as the stationary phase silica gel (FLUKA 0.035-0.070 mm, 220-240 mesh) and as the mobile phase benzene ethanol (8:1). The title compound was obtained by mixing solid with water (25 mL). Yield 0.109 g (22%); m.p. 235–237 °C; IR (KBr): 3395, 3208 (N-H), 2962, 2919, 2859 (C-H), 1589, 1581, 1558, 1526 (C=N, C=C$_{Ar}$), 1271, 1165 (SO_2) cm^{-1}; ^{1}H NMR (500 MHz, DMSO-*d*$_6$) δ: 1.33-1.62 (m, 6H, 2 × CH_3, pyrazol), 1.93 (s, 3H, CH_3, pyrazol), 2.29 (s, 3H, CH_3Ph), 2.74 (s, 2H, CH_2, pyrazol), 3.25 (t, *J=8.9 Hz*, 2H, imidazolidin), 3.41 (t, *J=8.7 Hz*, 2H, imidazolidin), 3.80-4.10 (m, 2H, S-CH_2), 4.32 (s, 2H, C**H**$_2$-C_6H_5), 6.90-7.37 (m, 5H, H$_{Ar}$ and 2H, NH_2 and 1H, NH, imidazolidin), 7.64 (s, 1H, H-3), 7.84 (s, 1H, H-6) ppm; ^{13}C NMR (125 MHz, DMSO-d$_6$) δ: 16.50, 19.67, 26.51, 26.71, 41.46, 44.92, 47.72, 53.41, 64.16, 128.08, 128.48, 129.01, 129.17, 129.48, 131.03, 132.25, 136.94, 137.32, 139.84, 154.98, 158.83, 163.04, 167.57, 173.83 ppm; Anal. calcd. for $C_{27}H_{32}ClN_9O_2S_2$ (614.19); C, 52.86; H, 5.30; N, 20.47. Found: C, 52.99; H, 5.36; N, 20.41.

2-{[4-Amino-6-(indolin-1-yl)-1,3,5-triazin-2-yl]methylthio}-N-(1-benzylimidazolidin-2-ylidene)-4-chloro-5-methylbenzenesulfonamide (**48**).

Starting from *N*-carbamimidoylindoline-1-carboximidamide hydrochloride (0.384 g, 1.60 mmol). The title compound was obtained. Yield 0.195 g (39%); m.p. 185–186 °C; IR (KBr): 3342, 3217 (N-H), 2955, 2923, 2888 (C-H), 1598, 1589, 1518 (C=N, C=C$_{Ar}$), 1285, 1130 (SO_2) cm^{-1}; ^{1}H NMR (500 MHz, DMSO-*d*$_6$) δ: 2.29 (s, 3H, CH_3), 3.06 (t, *J=8.6 Hz,* 2H, hydroindoline), 3.13-3.27 (m, 2H, imidazolidin), 3.34-3.41 (m, 2H, imidazolidin), 4.02-4.06 (m, 2H, hydroindoline and 2H, S-CH_2), 4.25-4.42 (m, 2H, C**H**$_2$-C_6H_5), 6.90-8.42 (m, 9H, H$_{Ar}$ and 1H, NH, imidazolidin and 1H, H-3 and 1H, H-6 and 2H, NH_2) ppm; ^{13}C NMR (125 MHz, DMSO-d$_6$) δ: 19.66, 27.09, 41.44, 44.90, 47.76, 48.43, 117.31, 122.79, 122.80, 125.35, 125.71, 127.30, 127.57, 128.05, 128.46, 129.12, 131.09, 132.31, 133.25, 133.96, 136.90, 137.24, 139.84, 143.15, 158.82, 167.24 ppm; Anal. calcd. for $C_{29}H_{29}ClN_8O_2S_2$ (621.18); C, 56.07; H, 4.71; N, 18.04. Found: C, 56.00; H, 4.68; N, 18.05.

2-{[4-Amino-6-(3,4-dihydroquinolin-1(2H)-yl)-1,3,5-triazin-2-yl]methylthio}-N-(1-benzylimidazolidin-2-ylidene)-4-chloro-5-methylbenzenesulfonamide (**49**).

Starting from *N*-carbamimidoyl-3,4-dihydroquinoline-1(2*H*)-carboximidamide hydrochloride (0.406 g, 1.60 mmol). The title compound was obtained. Yield 0.092 g (18%); m.p. 191–192 °C; IR (KBr): 3401, 3340, 3231 (N-H), 2940, 2910, 2868 (C-H), 1586, 1561, 1518 (C=N, C=C$_{Ar}$), 1268, 1167 (SO_2) cm^{-1}; ^{1}H NMR (500 MHz, DMSO-*d*$_6$) δ: 1.80 (quintet, *J=6.5 Hz*, 2H, 1,2,3,4-tetrahydroquinoline), 2.30 (s, 3H, CH_3), 2.69 (t, *J=6.7 Hz*, 2H, 1,2,3,4-tetrahydroquinoline), 3.24 (t, *J=8.75 Hz*, 2H, imidazolidin), 3.40 (t, *J=8.55 Hz*,

2H, imidazolidin), 3.87 (t, 2H, *J=6.1 Hz*, 2H, 1,2,3,4-tetrahydroquinoline), 3.97 (s, 2H, S-CH_2), 4.32 (s, 2H, **C****H**$_2$-C_6H_5), 6.93-7.68 (m, 9H, H_{Ar} and 2H, NH_2, 1H, NH, imidazolidin), 7.79 (s, 1H, H-3), 7.85 (s, 1H, H-6) ppm; ^{13}C NMR (125 MHz, DMSO-d_6) δ: 19.67, 23.98, 27.21, 41.46, 44.47, 44.93, 47.74, 124.02, 125.68, 126.16, 128.08, 128.50, 128.53, 128.97, 129.15, 131.03, 131.50, 132.28, 136.74, 136.92, 137.25, 139.23, 139.76, 158.84, 165.09, 167.47, 174.66 ppm; Anal. calcd. for $C_{30}H_{31}ClN_8O_2S_2$ (635.20); C, 56.73; H, 4.92; N, 17.64. Found: C, 56.68; H, 4.86; N, 17.59.

2-{[4-Amino-6-(phenylamino)-1,3,5-triazin-2-yl]methylthio}-N-(1-benzylimidazolidin-2-ylidene)-4-chloro-5-methylbenzenesulfonamide (**50**).

Starting from 1-phenylbiguanide hydrochloride (0.342 g, 1.60 mmol). The title compound was obtained after refluxing with mixture 15:1 ethanol/acetonitrile (1:37) in an ultrasonic bath for 10 minutes. Yield 0.186 g (39%); m.p. 200–201 °C; IR (KBr): 3406, 3317, 3219 (N-H), 2920 (C-H), 1595, 1497 (C=N, C=C_{Ar}), 1276, 1168 (SO_2) cm^{-1}; ^1H NMR (500 MHz, DMSO-d_6) δ: 2.31 (s, 3H, CH_3Ph), 3.24 (t, *J=8.55 Hz*, 2H, imidazolidin), 3.41 (t, *J=8.55 Hz*, 2H, imidazolidin), 4.01 (s, 2H, S-CH_2), 4.34 (s, 2H, C**H**$_2$-C_6H_5), 6.93-7.88 (m, 10H, H_{Ar} and 2H, NH_2 and 1H, NH, imidazolidin and 1H, H-3 and 1H, H-6), 9.50-9.60 (m, 1H, NH) ppm; Anal. calcd. for $C_{27}H_{27}ClN_8O_2S_2$ (595.14); C, 54.49; H, 4.57; N, 18.83. Found: C, 54.40; H, 4.63; N, 18.73. HRMS (ESI-TOF) (594.1387) calcd for $C_{27}H_{27}ClN_8O_2S_2$ $[M+H]^+$ (595.1465) found 595.1480.

2-{[4-Amino-6-{[4-(trifluoromethyl)phenyl]amino}-1,3,5-triazin-2-yl]methylthio}-N-[1-benzylimidazolidin-2-ylidene]-4-chloro-5-methylbenzenesulfonamide (**51**).

Starting from 1-[4-(trifluoromethyl)phenyl]biguanide hydrochloride (0.451 g, 1.60 mmol). The title compound was obtained after refluxing with ethanol (1:36) in an ultrasonic bath for 10 minutes. Yield 0.178 g (34%); m.p. 195–196 °C; IR (KBr): 3379, 3315, 3203 (N-H), 2987, 2960, 2922, 2854 (C-H), 1583, 1552, 1523, 1489 (C=N, C=C_{Ar}), 1276, 1163 (SO_2) cm^{-1}; ^1H NMR (500 MHz, DMSO-d_6) δ: 2.31 (s, 3H, CH_3Ph), 3.24 (t, *J=8.55 Hz*, 2H, imidazolidin), 3.40 (t, *J=8.55 Hz*, 2H, imidazolidin), 4.07 (s, 2H, S-CH_2), 4.33 (s, 2H, C**H**$_2$-C_6H_5), 7.16-7.94 (m, 9H, H_{Ar} and 2H, NH_2 and 1H, NH, imidazolidin and 1H, H-3 and 1H, H-6), 9.90-10.00 (m, 1H, N**H**-C_6H_4-4-CF_3) ppm; Anal. calcd. for $C_{28}H_{26}ClF_3N_8O_2S_2$ (663.14); C, 50.71; H, 3.95; N, 16.90. Found: C, 50.65; H, 3.85; N, 16.63; MALDI-TOF/TOF (662.1261) calcd for $C_{28}H_{26}ClF_3N_8O_2S_2$ $[M+H]^+$ (663.1339) found 663.1154.

2-[{4-Amino-6-[(4-methoxyphenyl)amino]-1,3,5-triazin-2-yl}methylthio]-N-(1-benzylimidazolidin-2-ylidene)-4-chloro-5-methylbenzenesulfonamide (**52**).

Starting from 1-(4-methoxyphenyl)biguanide hydrochloride (0.390 g, 1.60 mmol). The title compound was obtained. Yield 0.104 g (21%); m.p. 214–215 °C; IR (KBr): 3404, 3317, 3221 (N-H), 2956, 2925, 2855, 2834 (C-H), 1588, 1560, 1534, 1508 (C=N, C=C_{Ar}), 1275, 1168 (SO_2) cm^{-1}; ^1H NMR (500 MHz, DMSO-d_6) δ: 2.31 (s, 3H, CH_3Ph), 3.21-3.28 (m, 2H, imidazolidin), 3.38-3.45 (m, 2H, imidazolidin), 3.70 (s, 3H, O-C**H**$_3$), 3.97-4.01 (m, 2H, S-CH_2), 4.31-4.37 (m, 2H, CH_2-C_6H_5), 6.80-7.88 (m, 9H, H_{Ar} and 2H, NH_2 and 1H, H-3 and 1H, H-6), 9.29-9.48 (m, 1H, N**H**-C_6H_4-4-OMe) ppm; Anal. calcd. for $C_{28}H_{29}ClN_8O_3S_2$ (625.16); C, 53.79; H, 4.68; N, 17.92. Found: C, 53.47; H, 4.57; N, 17.62. HRMS (ESI-TOF) (624.1493) calcd for $C_{28}H_{29}ClN_8O_3S_2$ $[M+H]^+$ (625.1571) found 625.1590.

2-{[4-Amino-6-(benzylamino)-1,3,5-triazin-2-yl]methylthio}-N-(1-benzylimidazolidin-2-ylidene)-4-chloro-5-methylbenzenesulfonamide (**53**).

Starting from 2-benzyl-1-(diaminomethylidene)guanidine hydrochloride (0.364 g, 1.60 mmol). The title compound was obtained after refluxing with acetonitrile (1:22) in an ultrasonic bath for 10 minutes and next solid washed with 1 mL hot ethanol. Yield 0.288 g (59%); m.p. 220–221 °C; IR (KBr): 3398, 3380, 3347 (N-H), 2986, 2955, 2883, 2843 (C-H), 1595, 1580, 1556, 1526 (C=N, C=C_{Ar}), 1264, 1172 (SO_2) cm^{-1}; ^1H NMR (500 MHz, DMSO-d_6 in T=100 °C) δ: 2.32 (s, 3H, CH_3), 3.32 (t, *J=8.8 Hz*, 2H, imidazolidin), 3.47 (t, *J=8.4 Hz*, 2H, imidazolidin), 3.90 (s, 2H, S-CH_2), 4.39 (s, 2H, **CH**$_2$-C_6H_5), 4.49 (dd, *J=6.4 Hz*, 2H, **CH**$_2$Ph), 6.74-6.86 (m, 2H, NH_2), 7.18-7.36 (m, 10H, H_{Ar} and 1H, NH, imidazolidin), 7.74 (s, 1H, H-3), 7.85 (s, 1H,

H-6) ppm; Anal. calcd. for $C_{28}H_{29}ClN_8O_2S_2$ (609.17); C, 55.21; H, 4.80; N, 18.39. Found: C, 55.16; H, 4.74; N, 18.32. HRMS (ESI-TOF) (608.1543) calcd for $C_{38}H_{39}ClFN_9O_2S_2$ $[M+H]^+$ (609.1621) found 609.1642.

2-[{4-Amino-6-[methyl(phenyl)amino]-1,3,5-triazin-2-yl}methylthio]-N-(1-benzylimidazolidin-2-ylidene)-4-chloro-5-methylbenzenesulfonamide (**54**).

Starting from 1-methyl-1-phenylbiguanide hydrochloride **(**0.364 g, 1.60 mmol**)**. The title compound was obtained. Yield 0.108 g (22%); m.p. 191–192 °C; IR (KBr): 3353, 3142 (N-H), 2971, 2947, 2919, 2880 (C-H), 1595, 1582, 1565, 1525 (C=N, C=C_{Ar}), 1268, 1129 (SO_2) cm^{-1}; ^1H NMR (500 MHz, DMSO-d_6) δ: 2.30 (s, 3H, CH_3Ph), 3.27 (t, *J=8.8 Hz*, 2H, imidazolidin), 3.36 (s, 3H, N-C**H**$_3$), 3.42 (t, *J=8.7 Hz*, 2H, imidazolidin), 3.91 (s, 2H, S-CH_2), 4.35 (s, 2H, C**H**$_2$-C_6H_5), 6.83-7.03 (m, 2H, NH_2), 7.18-7.39 (m, 9H, H_{Ar} and 1H, NH, imidazolidin), 7.81 (s, 1H, H-3), 7.84 (s, 1H, H-6) ppm; ^{13}C NMR (125 MHz, DMSO) δ: 19.66, 38.46, 41.48, 44.95, 47.76, 126.38, 127.36, 128.10, 128.35, 128.52, 129.17, 129.43, 130.99, 132.19, 136.89, 136.93, 137.27, 139.59, 144.86, 158.83, 165.84, 167.35, 174.49 ppm; Anal. calcd. for $C_{28}H_{29}ClN_8O_2S_2$ (609.17); C, 55.21; H, 4.80; N, 18.39. Found: C, 55.18; H, 4.76; N, 18.34.

2-[{4-Amino-6-[(4-chlorophenyl)(methyl)amino]-1,3,5-triazin-2-yl}methylthio]-N-(1-benzylimidazolidin-2-ylidene)-4-chloro-5-methylbenzenesulfonamide (**55**).

Starting from 1-(4-chlorophenyl)-1-methylbiguanide hydrochloride (0.419 g, 1.60 mmol)**.** The title compound was obtained. Yield 0.141 g (27%); m.p. 217–218 °C; IR (KBr): 3397, 3354, 3124 (N-H), 2970, 2928, 2890, 2856 (C-H), 1591, 1580, 1564, 1526 (C=N, C=C_{Ar}), 1265, 1128 (SO_2) cm^{-1}; ^1H NMR (500 MHz, DMSO-d_6) δ: 2.30 (s, 3H, CH_3), 3.26 (t, *J=7.2 Hz*, 2H, imidazolidin), 3.34 (s, 3H, CH_3-N), 3.42 (t, *J=7.3 Hz*, 2H, imidazolidin), 3.91 (s, 2H, S-CH_2), 4.34 (s, 2H, C**H**$_2$-C_6H_5), 7.00-7.05 (m, 2H, NH_2), 7.18-7.40 (m, 9H, H_{Ar} and 1H, NH, imidazolidin), 7.77 (s, 1H, H-3), 7.84 (s, 1H, H-6) ppm; ^{13}C NMR (125 MHz, DMSO-d_6) δ: 19.67, 38.08, 41.48, 44.95, 47.75, 128.10, 128.33, 128.51, 129.07, 129.17, 129.21, 130.40, 130.97, 132.16, 136.79, 136.92, 137.25, 139.55, 143.63, 158.82, 165.77, 167.36, 174.64 ppm; Anal. calcd. for $C_{28}H_{28}Cl_2N_8O_2S_2$ (643.61); C, 52.25; H, 4.39; N, 17.41. Found: C, 52.16; H, 4.34; N, 17.36.

2-{[4-Amino-6-(4-methylpiperazin-1-yl)-1,3,5-triazin-2-yl]methylthio}-N-(1-benzylimidazolidin-2-ylidene)-4-chloro-5-methylbenzenesulfonamide (**56**).

Starting from *N*-carbamimidoyl-4-methylpiperazine-1-carboximidamide hydrochloride (0.353 g, 1.60 mmol). The title compound was obtained after refluxing with ethanol (1:24) in an ultrasonic bath for 10 minutes. Yield 0.220 g (46%); m.p. 203–204 °C. IR (KBr): 3393, 3334, 3155 (N-H), 2931, 2909, 2864, 2853, (C-H), 1594, 1557, 1523 (C=N, C=C_{Ar}), 1342, 1168 (SO_2) cm^{-1}; ^1H NMR (500 MHz, DMSO-d_6) δ: 2.15 (s, 3H, C**H**$_3$-piperazine), 2.19-2.27 (m, 4H, piperazine), 2.31 (s, 3H, CH_3Ph), 3.27 (t, *J=8.8 Hz*, 2H, imidazolidin), 3.43 (t, *J=8.8 Hz*, 2H, imidazolidin), 3.62-3.67 (m, 4H piperazine), 3.91 (s, 2H, S-CH_2), 4.35 (s, 2H, C**H**$_2$-C_6H_5), 6.90-7.40 (m, 5H, H_{Ar} and 2H, NH_2 and 1H, NH, imidazolidin), 7.85 (s, 1H, H-3), 7.95 (s, 1H, H-6) ppm; Anal. calcd. for $C_{26}H_{32}ClN_9O_2S_2$ (602.17); C, 51.86; H, 5.36; N, 20.93. Found: C, 51.81; H, 5.25; N, 20.90. HRMS (ESI-TOF) (601.1809) calcd for $C_{26}H_{32}ClN_9O_2S_2$ $[M+H]^+$ (602.1887) found 602.1893.

2-{[4-Amino-6-(4-phenylpiperazin-1-yl)-1,3,5-triazin-2-yl]methylthio}-N-(1-benzylimidazolidin-2-ylidene)-4-chloro-5-methylbenzenesulfonamide (**57**).

Starting from *N*-carbamimidoyl-4-phenylpiperazine-1-carboximidamide hydrochloride (0.452 g, 1.60 mmol). The title compound was obtained. Yield 0.086 g (16%); m.p. 108–110 °C; IR (KBr): 3388, 3211 (N-H), 2919, 2854, 2817 (C-H), 1593, 1577, 1520 (C=N, C=C_{Ar}), 1278, 1128 (SO_2) cm^{-1}; ^1H NMR (500 MHz, DMSO-d_6) δ: 2.30 (s, 3H, CH_3), 3.05-3.10 (m, 4H, piperazine), 3.26 (t, *J=8.8 Hz*, 2H, imidazolidin), 3.42 (t, *J=8.55 Hz*, 2H, imidazolidin), 3.80-3.82 (m, 4H, piperazine), 3.94 (s, 2H, S-CH_2), 4.36 (s, 2H, C**H**$_2$-C_6H_5), 6.78-7.40 (m, 10H, H_{Ar} and 2H, NH_2 and 1H, NH, imidazolidin), 7.85 (s, 1H, H-3), 7.94 (s, 1H, H-6) ppm; ^{13}C NMR (125 MHz, DMSO-d_6) δ: 18.99, 40.82, 42.54, 44.28, 47.12, 48.35, 115.86, 115.96, 119.38, 127.49, 127.93, 128.54, 129.01, 130.26, 130.43, 131.50, 136.27, 136.55, 138.91, 150.95, 158.14, 164.23, 166.95, 173.90

ppm; Anal. calcd. for $C_{31}H_{34}ClN_9O_2S_2$ (664.24); C, 56.05; H, 5.16; N, 18.98. Found: C, 56.00; H, 5.23; N, 19.03.

2-[{4-Amino-6-[4-(4-fluorophenyl)piperazin-1-yl]-1,3,5-triazin-2-yl}methylthio]-N-(1-benzylimidazolidin-2-ylidene)-4-chloro-5-methylbenzenesulfonamide (**58**).

Starting from *N*-carbamimidoyl-4-(4-fluorophenyl)piperazine-1-carboximidamide hydrochloride (0.481 g, 1.60 mmol). The title compound was obtained Yield 0.237 g (43%); m.p. 204–205 °C; IR (KBr): 3361, 3234 (N-H), 2956, 2935, 2903, 2861 (C-H), 1590, 1554, 1515, 1477 (C=N, C=C_{Ar}), 1265, 1139 (SO_2) cm^{-1}; ^1H NMR (500 MHz, DMSO-d_6) δ: 2.30 (s, 3H, CH_3Ph), 3.00-3.03 (m, 4H, piperazine), 3.27 (t, *J=8.8 Hz,* 2H, imidazolidin), 3.42 (t, *J=8.8 Hz,* 2H, imidazolidin), 3.79-3.83 (m, 4H, piperazine), 3.94 (s, 2H, S-CH_2), 4.36 (s, 2H, **CH_2**-C_6H_5), 6.94-7.40 (m, 9H, H_{Ar} and 2H, NH_2 and 1H, NH, imidazolidin), 7.85 (s, 1H, H-3), 7.94 (s, 1H, H-6) ppm; Anal. calcd. for $C_{31}H_{33}ClFN_9O_2S_2$ (682.23); C, 54.58; H, 4.88; N, 18.48. Found: C, 54.65; H, 4.94; N, 18.48. HRMS (ESI-TOF) (681.1871) calcd for $C_{31}H_{33}ClFN_9O_2S_2$ $[M+H]^+$ (682.1949) found 682.1981.

2-[{4-Amino-6-[4-(2-fluorophenyl)piperazin-1-yl]-1,3,5-triazin-2-yl}methylthio]-N-(1-benzylimidazolidin-2-ylidene)-4-chloro-5-methylbenzenesulfonamide (**59**).

Starting from *N*-carbamimidoyl-4-(2-fluorophenyl)piperazine-1-carboximidamide hydrochloride (0.481 g, 1.60 mmol). The title compound was obtained by crystallization from methanol (1:3), remained part by crystallization from filtrate. Yield 0.224 g (41%); m.p. 194–195 °C; IR (KBr): IR (cm^{-1}): 3430, 3351, 3247 (N-H), 2970, 2918, 2858, 2828 (C-H), 1592, 1570, 1520, 1499 (C=N, C=C_{Ar}), 1256, 1163 (SO_2) cm^{-1}; ^1H NMR (500 MHz, DMSO-d_6) δ: 2.30 (s, 3H, CH_3Ph), 2.90-2.98 (m, 4H, piperazine), 3.27 (t, *J=8.8 Hz*, 2H, imidazolidin), 3.42 (t, *J=8.8 Hz*, 2H, imidazolidin), 3.80-3.85 (m, 4H, piperazine), 3.94 (s, 2H, S-CH_2), 4.36 (s, 2H, **CH_2**-C_6H_5), 6.99-7.40 (m, 9H, H_{Ar} and 2H, NH_2), 7.85 (s, 1H, H-3), 7.94 (s, 1H, H-6) ppm; Anal. calcd. for $C_{31}H_{33}ClFN_9O_2S_2$ (682.23); C, 54.58; H, 4.88; N, 18.48. Found: C, 54.57; H, 4.85; N, 15.41. HRMS (ESI-TOF) (681.1871) calcd for $C_{31}H_{33}ClFN_9O_2S_2$ $[M+H]^+$ (682.1949) found 682.1969.

2-{[4-Amino-6-{4-[4-(trifluoromethyl)phenyl]piperazin-1-yl}-1,3,5-triazin-2-yl]methylthio}-N-(1-benzylimidazolidin-2-ylidene)-4-chloro-5-methylbenzenesulfonamide (**60**).

Starting from *N*-carbamimidoyl-4-[4-(trifluoromethyl)phenyl]piperazine-1-carboximidamide hydrochloride (0.561 g, 1.60 mmol). The title compound was obtained by crystallization from the mixture 1:1 ethanol/acetonitrile (1:15), remained part by crystallization from filtrate. Yield 0.217 g (37%); m.p. 214–215 °C; IR (KBr): 3384, 3332, 3223 (N-H), 2979, 2921, 2869 (C-H), 1554, 1516, 1482 (C=N, C=C_{Ar}), 1275, 1163 (SO_2) cm^{-1}; ^1H NMR (500 MHz, DMSO-d_6) δ: 2.32 (s, 3H, CH_3Ph), 3.26-3.32 (m, 4H piperazine and 2H, imidazolidin), 3.43 (t, *J=8.8 Hz*, 2H, imidazolidin), 3.80-3.85 (m, 4H, piperazine), 3.95 (s, 2H, S-CH_2), 4.37 (s, 2H, **CH_2**-C_6H_5), 7.00-7.54 (m, 9H, H_{Ar} and 1H, NH, imidazolidin and 2H, NH_2), 7.86 (s, 1H, H-3), 7.95 (s, 1H, H-6) ppm; Anal. calcd. for $C_{32}H_{33}ClF_3N_9O_2S_2$ (732.24); C, 52.49; H, 4.54; N, 17.22. Found: C, 52.41; H, 4.45; N, 17.00. HRMS (ESI-TOF) (731.1839) calcd for $C_{32}H_{33}ClF_3N_9O_2S_2$ $[M+H]^+$ (732.1917) found 732.1901.

2-{[4-Amino-6-{4-[3-(trifluoromethyl)phenyl]piperazin-1-yl}-1,3,5-triazin-2-yl]methylthio}-N-(1-benzylimidazolidin-2-ylidene)-4-chloro-5-methylbenzenesulfonamide (**61**).

Starting from *N*-carbamimidoyl-4-[3-(trifluoromethyl)phenyl]piperazine-1-carboximidamide hydrochloride (0.561 g, 1.60 mmol). The title compound was obtained by crystallization from mixture 1:1 ethanol/acetonitrile (1:8). Yield 0.310 g (53%); m.p. 195–196 °C; IR (KBr): 3339, 3230, 3079 (N-H), 2955, 2914, 2876 (C-H), 1600, 1561, 1526 (C=N, C=C_{Ar}), 1287, 1164 (SO_2) cm^{-1}; ^1H NMR (500 MHz, DMSO-d_6) δ: 2.31 (s, 3H, CH_3Ph), 3.18-3.30 (m, 4H, piperazine and 2H, imidazolidin), 3.43 (t, *J=8.8 Hz,* 2H, imidazolidin), 3.83 (s, 4H, piperazine), 3.95 (s, 2H, S-CH_2), 4.37 (s, 2H, **CH_2**-C_6H_5), 7.00-7.46 (m, 9H, H_{Ar} and 2H, NH_2 and 1H, NH, imidazolidin), 7.86 (s, 1H, H-3), 7.95 (s, 1H, H-6) ppm; Anal. calcd. for

$C_{32}H_{33}ClF_3N_9O_2S_2$ (732.24); C, 52.49; H, 4.54; N, 17.22. Found: C, 52.28; H, 4.40; N, 17.09; MALDI-TOF/TOF (731.1839) calcd for $C_{32}H_{33}ClF_3N_9O_2S_2$ $[M+H]^+$ (732.1917) found 732.1992.

2-[{4-Amino-6-[4-(4-chlorophenyl)piperazin-1-yl]-1,3,5-triazin-2-yl}methylthio]-N-(1-benzylimidazolidin-2-ylidene)-4-chloro-5-methylbenzenesulfonamide (**62**).

Starting from *N*-carbamimidoyl-4-(4-chlorophenyl)piperazine-1-carboximidamide hydrochloride (0.508 g, 1.60 mmol). The title compound was obtained. Yield 0.118 g (21%); m.p. 181–182 °C; IR (KBr): 3366, 3324, 3226 (N-H), 2973, 2902, 2858, 2827 (C-H), 1594, 1577, 1557, 1528 (C=N, $C=C_{Ar}$), 1291, 1167 (SO_2) cm^{-1}; ^1H NMR (500 MHz, DMSO-d_6) δ: 2.29 (s, 3H, CH_3), 3.05-3.10 (m, 4H, piperazine), 3.26 (t, *J=8.8 Hz*, 2H, imidazolidin), 3.41 (t, *J=8.55 Hz*, 2H, imidazolidin), 3.69-3.87 (m, 4H, piperazine), 3.93 (s, 2H, S-CH_2), 4.35 (s, 2H, $\mathbf{CH_2}$-C_6H_5), 6.93-7.39 (9H, H_{Ar} and 1H, NH, imidazolidin and 1H, NH_2), 7.84 (s, 1H, H-3), 7.93 (s, 1H, H-6) ppm; ^{13}C NMR (125 MHz, DMSO-d_6) δ: 19.65, 41.47, 42.91, 44.93, 47.78, 48.68, 117.99, 123.46, 128.14, 128.42, 128.60, 129.19, 129.35, 131.00, 132.13, 136.91, 136.93, 137.20, 139.55, 150.38, 158.78, 164.90, 167.60, 174.57 ppm; Anal. calcd. for $C_{31}H_{33}Cl_2N_9O_2S_2$ (698.69); C, 53.29; H, 4.76; N, 18.04. Found: C, 53.20; H, 4.74; N, 17.99.

2-[{4-Amino-6-[4-(3-chlorophenyl)piperazin-1-yl]-1,3,5-triazin-2-yl}methylthio]-N-(1-benzylimidazolidin-2-ylidene)-4-chloro-5-methylbenzenesulfonamide (**63**).

Starting from *N*-carbamimidoyl-4-(3-chlorophenyl)piperazine-1-carboximidamide hydrochloride (0.508 g, 1.60 mmol). The title compound was obtained after refluxing with ethanol (1:36) in an ultrasonic bath for 10 minutes. Yield 0.275 g (49%); m.p. 198–199 °C; IR (KBr): 3350, 3245 (N-H), 2973, 2914, 2851, 2814 (C-H), 1593, 1569, 1523, 1486 (C=N, $C=C_{Ar}$), 1263, 1162 (SO_2) cm^{-1}; ^1H NMR (500 MHz, DMSO-d_6) δ: 2.32 (s, 3H, CH_3Ph), 3.12-3.17 (m, 4H, piperazine), 3.28 (t, *J=5.9 Hz*, 2H, imidazolidin), 3.43 (t, *J=5.9 Hz*, 2H, imidazolidin), 3.78-3.83 (m, 4H, piperazine), 3.95 (s, 2H, S-CH_2), 4.37 (s, 2H, $\mathbf{CH_2}$-C_6H_5), 6.81-7.41 (m, 9H, H_{Ar} and 2H, NH_2 and 1H, NH, imidazolidin), 7.86 (s, 1H, H-3), 7.95 (s, 1H, H-6) ppm; Anal. calcd. for $C_{31}H_{33}Cl_2N_9O_2S_2$ (698.69); C, 53.29; H, 4.76; N, 18.04. Found: C, 53.10; H, 4.68; N, 17.77; MALDI-TOF/TOF (697.1576) calcd for $C_{31}H_{33}Cl_2N_9O_2S_2$ $[M+H]^+$ (698.1654) found 698.1655.

2-[{4-Amino-6-[4-(3,4-dichlorophenyl)piperazin-1-yl]-1,3,5-triazin-2-yl}methylthio]-N-(1-benzylimidazolidin-2-ylidene)-4-chloro-5-methylbenzenesulfonamide (**64**).

Starting from *N*-carbamimidoyl-4-(3,4-dichlorophenyl)piperazine-1-carboximidamide hydrochloride (0.563 g, 1.60 mmol). The title compound was obtained by crystallization from 2:1:1 acetonitrile/cyclopentanone/ethanol (1:15), remained part by crystallization from filtrate. Next all was refluxing with ethanol (1:22) in an ultrasonic bath for 10 minutes. Next all was crystallization from chloroform (1:29). Yield 0.177 g (30%); m.p. 223–225 °C; IR (KBr): 3395, 3340, 3223 (N-H), 2980, 2942, 2916, 2873 (C-H), 1595, 1557, 1520, 1485 (C=N, $C=C_{Ar}$), 1282, 1166 (SO_2) cm^{-1}; ^1H NMR (500 MHz, DMSO-d_6) δ: 2.31 (s, 3H, CH_3Ph), 3.13-3.17 (m, 4H, piperazine), 3.27 (t, *J=9.0 Hz*, 2H, imidazolidin), 3.42 (t, *J=8.8 Hz*, 2H, imidazolidin), 3.79 (s, 4H, piperazine), 3.94 (s, 2H, S-CH_2), 4.36 (s, 2H, $\mathbf{CH_2}$-C_6H_5), 6.92-7.42 (m, 8H, H_{Ar} and 2H, NH_2 and 1H, NH, imidazolidin), 7.85 (s, 1H, H-3), 7.94 (s, 1H, H-6) ppm; Anal. calcd. for $C_{31}H_{32}Cl_3N_9O_2S_2$ (733.13); C, 50.79; H, 4.40; N, 17.19. Found: C, 50.80; H, 4.41; N, 17.10. HRMS (ESI-TOF) (731.1186) calcd for $C_{31}H_{32}Cl_3N_9O_2S_2$ $[M+H]^+$ (732.1264) found 732.1268.

2-[{4-Amino-6-[4-(3-chloro-4-fluorophenyl)piperazin-1-yl]-1,3,5-triazin-2-yl}methylthio]-N-(1-benzylimidazolidin-2-ylidene)-4-chloro-5-methylbenzenesulfonamide (**65**).

Starting from *N*-carbamimidoyl-4-(3-chloro-4-fluorophenyl)piperazine-1-carboximidamide hydrochloride (0.536 g, 1.60 mmol). Next all was refluxing with ethanol (1:38) in an ultrasonic bath for 10 minutes and next obtain solid was washed 1 mL benzene and 2 mL acetonitrile. Yield 0.291 g (51%); m.p. 201–202 °C; IR (KBr): 3391, 3331, 3223 (N-H), 2983, 2945, 2918, 2878 (C-H), 1603, 1558, 1504, 1474 (C=N, $C=C_{Ar}$), 1271, 1166 (SO_2)cm^{-1}; ^1H NMR (500 MHz, DMSO-d_6) δ: 2.31 (s, 3H, CH_3Ph), 3.06-3.10 (m, 4H, piperazine), 3.28 (t, *J=8.8 Hz*, 2H, imidazolidin), 3.43 (t, *J=8.8 Hz*, 2H, imidazolidin), 3.78-3.83 (m, 4H,

piperazine), 3.95 (s, 2H, S-CH_2), 4.37 (s, 2H, C**H**$_2$-C_6H_5), 6.93-7.41 (m, 8H, H_{Ar} and 2H, NH_2 and 1H, NH, imidazolidin), 7.86 (s, 1H, H-3), 7.95 (s, 1H, H-6) ppm; Anal. calcd. for $C_{31}H_{32}Cl_2FN_9O_2S_2$ (716.68); C, 51.95; H, 4.50; N, 17.59. Found: C, 51.88; H, 4.41; N, 17.27; MALDI-TOF/TOF (715.1481) calcd for $C_{31}H_{32}Cl_2FN_9O_2S_2$ $[M+H]^+$ (716.1559) found 716.0973.

2-[{4-Amino-6-[4-(2-methoxyphenyl)piperazin-1-yl]-1,3,5-triazin-2-yl}methylthio]-N-(1-benzylimidazolidin-2-ylidene)-4-chloro-5-methylbenzenesulfonamide (**66**).

Starting from *N*-carbamimidoyl-4-(2-methoxyphenyl)piperazine-1-carboximidamide hydrochloride (0.500 g, 1.60 mmol). The title compound was obtained by crystallization from the mixture 4:1 ethanol/acetone (1:12), remained part by crystallization from filtrate. Yield 0.201 g (36%); m.p. 178–179 °C; IR (KBr): 3378, 3320, 3225 (N-H), 2973, 2953, 2902, 2893 (C-H), 1569, 1527, 1494, 1478 (C=N, C=C_{Ar}), 1384, 1165 (SO_2) cm^{-1}; ^1H NMR (500 MHz, DMSO-d_6) δ: 2.30 (s, 3H, CH_3Ph), 2.82-2.93 (m, 4H, piperazine), 3.27 (t, *J=8.8 Hz*, 2H, imidazolidin), 3.42 (t, *J=8.8 Hz*, 2H, imidazolidin), 3.78-3.81 (m, 4H, piperazine and 3H, O-C**H**$_3$), 3.94 (s, 2H, S-CH_2), 4.35 (s, 2H, C**H**$_2$-C_6H_5), 6.84-7.40 (m, 9H, H_{Ar} and 2H, NH_2 and 1H, NH, imidazolidin), 7.85 (s, 1H, H-3), 7.94 (s, 1H, H-6) ppm; Anal. calcd. for $C_{32}H_{36}ClN_9O_3S_2$ (694.27); C, 55.36; H, 5.23; N, 18.16. Found: C, 55.28; H, 5.19; N, 17.99. HRMS (ESI-TOF) (693.2071) calcd for $C_{32}H_{36}ClN_9O_3S_2$ $[M+H]^+$ (694.2149) found 694.2140.

2-[{4-Amino-6-[4-(4-nitrophenyl)piperazin-1-yl]-1,3,5-triazin-2-yl}methylthio]-N-(1-benzylimidazolidin-2-ylidene)-4-chloro-5-methylbenzenesulfonamide (**67**).

Starting from *N*-carbamimidoyl-4-(4-nitrophenyl)piperazine-1-carboximidamide hydrochloride (0.524 g, 1.60 mmol). The title compound was obtained by crystallization from mixture 2:1 cyclohexanol/ EtOH (1:18). Yield 0.264 g (47%); m.p. 249–250 °C (dec.); IR (KBr): 3420, 3390, 3325, 3221 (N-H), 2991, 2915, 2869 (C-H), 1596, 1547, 1482 (C=N, C=C_{Ar}), 1515, 1344 (N-O), 1272, 1166 (SO_2) cm^{-1}; ^1H NMR (500 MHz, DMSO-d_6) δ: 2.31 (s, 3H, CH_3Ph), 3.27 (t, *J=8.55 Hz*, 2H, imidazolidin), 3.43 (t, *J=8.75 Hz*, 2H, imidazolidin), 3.47-3.52 (m, 4H, piperazine), 3.79-3.86 (m, 4H, piperazine), 3.94 (s, 2H, S-CH_2), 4.37 (s, 2H, C**H**$_2$-C_6H_5), 7.00-7.95 (m, 9 H_{Ar} and 2H, NH_2 and 1H, NH, imidazolidin), 8.07 (s, 1H, H-3), 8.09 (s, 1H, H-6) ppm; Anal. calcd. for $C_{38}H_{40}ClN_9O_2S_2$ (709.24); C, 52.50; H, 4.69; N, 19.75. Found: C, 52.66; H, 4.80; N, 19.43. HRMS (ESI-TOF) (708.1816) calcd for $C_{31}H_{33}ClN_{10}O_4S_2$ $[M+H]^+$ (709.1894) found 709.1932.

2-{[4-Amino-6-(4-benzylpiperazin-1-yl)-1,3,5-triazin-2-yl]methylthio}-N-(1-benzylimidazolidin-2-ylidene)-4-chloro-5-methylbenzenesulfonamide (**68**).

Starting from 4-benzyl-*N*-carbamimidoylpiperazine-1-carboximidamide hydrochloride (0.475 g, 1.60 mmol). The title compound was obtained crystallization from mixture 2:1:1 EtOH/cyclopentanone/MeCN (1:13). Yield 0.286 g (53%); m.p. 112–114 °C; IR (KBr): 3395, 3334, 3239 (N-H), 2947, 2916, 2897, 2861 (C-H), 1592, 1581, 1556, 1519 (C=N, C=C_{Ar}), 1284, 1167 (SO_2) cm^{-1}; ^1H NMR (500 MHz, DMSO-d_6) δ: 2.29 (s, 3H, CH_3Ph and 4H, piperazine), 3.27 (t, *J=8.5 Hz*, 2H, imidazolidin), 3.42 (t, *J=9.0 Hz*, 4H, imidazolidin and 2H, Ph-CH_2), 3.62-3.69 (m, 4H, piperazine), 3.91 (s, 2H, S-CH_2), 4.34 (s, 2H, C**H**$_2$-C_6H_5), 6.92-7.40 (m, 9H, H_{Ar} and 2H, NH_2 and 1H, NH, imidazolidin), 7.84 (s, 1H, H-3), 7.92 (s, 1H, H-6) ppm; Anal. calcd. for $C_{32}H_{36}ClN_9O_2S_2$ (678.27); C, 56.67; H, 5.35; N, 18.59. Found: C, 56.43; H, 5.34; N, 18.80. HRMS (ESI-TOF) (677.2122) calcd for $C_{32}H_{36}ClN_9O_2S_2$ $[M+H]^+$ (678.2200) found 678.2185.

2-[{4-Amino-6-[4-(4-benzhydrylphenyl)piperazin-1-yl]-1,3,5-triazin-2-yl}methylthio]-N-(1-benzylimidazolidin-2-ylidene)-4-chloro-5-methylbenzenesulfonamide (**69**).

Starting from 4-benzhydryl-*N*-carbamimidoylpiperazine-1-carboximidamide hydrochloride (0.597 g, 1.60 mmol). The title compound was obtained by crystallization from mixture 2:1:1 EtOH/ cyclopentanone/MeCN (1:21). Yield 0.263 g (40%); m.p. 225–226 °C; IR (KBr): 3383, 3338, 3241 (N-H), 2951, 2915, 2893, 2853 (C-H), 1603, 1557, 1519, 1489 (C=N, C=C_{Ar}), 1273, 1165 (SO_2) cm^{-1}; ^1H NMR (500 MHz, DMSO-d_6) δ: 2.16-2.28 (m, 4H, piperazine and 3H, CH_3Ph), 3.24 (t, *J=8.8 Hz*, 2H, imidazolidin), 3.41 (t, *J=8.75 Hz*, 2H, imidazolidin), 3.61-3.67 (m, 4H, piperazine), 3.90 (s, 2H, S-CH_2), 4.27 (s, 1H,

C**H**Ph$_2$), 4.31 (s, 2H, C**H**$_2$-C$_6$H$_5$), 6.95-7.42 (m, 15H, H$_{Ar}$ and 2H, NH$_2$), 7.81 (s, 1H, H-3), 7.86 (s, 1H, H-6) ppm; Anal. calcd. for $C_{38}H_{40}ClN_9O_2S_2$ (754.37); C, 60.50; H, 5.34; N, 16.71. Found: C, 60.34; H, 5.34; N, 16.58. HRMS (ESI-TOF) (753.2435) calcd for $C_{38}H_{40}ClN_9O_2S_2$ [M+H]$^+$ (754.2513) found 754.2514.

2-[{4-Amino-6-[4-(phenylsulfonyl)piperazin-1-yl]-1,3,5-triazin-2-yl}methylthio]-N-(1-benzylimidazolidin-2-ylidene)-4-chloro-5-methylbenzenesulfonamide (**70**).

Starting from *N*-carbamimidoyl-4-(phenylsulfonyl)piperazine-1-carboximidamide hydrochloride (0.555 g, 1.60 mmol). The title compound was obtained after refluxing with mixture 4:1 ethanol/acetonitrile (1:27) in an ultrasonic bath for 10 minutes. Yield 0.319 g (55%); m.p. 208–209 °C. IR (KBr): 3305, 3196, 3125 (N-H), 2916, 2897, 2862 (C-H), 1595, 1566, 1520 (C=N, C=C$_{Ar}$), 1276, 1170 (SO$_2$) cm^{-1}; ^{1}H NMR (500 MHz, DMSO-*d*$_6$) δ: 2.31 (s, 3H, CH$_3$), 2.79-2.87 (m, 4H, piperazine), 3.25 (t, *J=8.8 Hz*, 2H, imidazolidin), 3.41 (t, *J=9.0 Hz*, 2H, imidazolidin), 3.75 (s, 4H, piperazine), 3.90 (s, 2H, S-CH$_2$), 4.31 (s, 2H, CH$_2$Ph), 6.98-7.73 (m, 10H, H$_{Ar}$ and 1H, NH, imidazolidin and 2H, NH$_2$), 7.82 (s, 1H, H-3), 7.84 (s, 1H, H-6) ppm; Anal. calcd. for $C_{31}H_{34}ClN_9O_4S_3$ (728.31); C, 51.12; H, 4.71; N, 17.31. Found: C, 51.01; H, 4.66; N, 17.22. HRMS (ESI-TOF) (727.1584) calcd for $C_{31}H_{34}ClN_9O_4S_3$ [M+H]$^+$ (728.1662) found 728.1860.

2-{[4-Amino-6-(4-tosylpiperazin-1-yl)-1,3,5-triazin-2-yl]methylthio}-N-(1-benzylimidazolidin-2-ylidene)-4-chloro-5-methylbenzenesulfonamide (**71**).

Starting from *N*-carbamimidoyl-4-[(4-methylphenyl)sulfonyl]piperazine-1-carboximidamide hydrochloride (0.577 g, 1.60 mmol). The title compound was obtained after refluxing with mixture 8:3 ethanol/acetonitrile (1:26) in an ultrasonic bath for 10 minutes and next all was mixted of DCM (1:30) for 20h. Yield 0.275 g (46%); m.p. 237–239 °C; IR (KBr): 3307, 3191 (N-H), 2958, 2919, 2898, 2862 (C-H), 1595, 1579, 1566, 1520 (C=N, C=C$_{Ar}$), 1265, 1166 (SO$_2$) cm^{-1}; ^{1}H NMR (500 MHz, DMSO-*d*$_6$) δ: 2.31 (s, 3H, CH$_3$), 2.38 (s, 3H, CH$_3$), 2.73-2.84 (m, 4H, piperazine), 3.25 (t, *J=8.8 Hz*, 2H, imidazolidin), 3.41 (t, *J=8.8 Hz*, 2H, imidazolidin), 3.75 (s, 4H, piperazine), 3.90 (s, 2H, S-CH$_2$), 4.30 (s, 2H, CH$_2$Ph), 6.97-7.58 (m, 9H, H$_{Ar}$ and 1H, NH, imidazolidin and 2H, NH$_2$), 7.81 (s, 1H, H-3), 7.85 (s, 1H, H-6) ppm; Anal. calcd. for $C_{32}H_{36}ClN_9O_4S_3$ (742.33); C, 51.77; H, 4.89; N, 16.98. Found: C, 51.82; H, 4.91; N, 16.99. HRMS (ESI-TOF) (741.1741) calcd for $C_{32}H_{36}ClN_9O_4S_3$ [M+H]$^+$ (742.1819) found 742.2122.

2-{[4-Amino-6-(4-benzylpiperidin-1-yl)-1,3,5-triazin-2-yl]methylthio}-N-(1-benzylimidazolidin-2-ylidene)-4-chloro-5-methylbenzenesulfonamide (**72**).

Starting from 4-benzyl-*N*-carbamimidoylpiperidine-1-carboximidamide hydrochlorid (0.473 g, 1.60 mmol). The title compound was obtained after refluxing with ethanol (1:35) in an ultrasonic bath for 10 minutes. Yield 0.246 g (40%); m.p. 180–181 °C; IR (KBr): 3338, 3208, 3144 (N-H), 2925, 2847 (C-H), 1581, 1549, 1519 (C=N, C=C$_{Ar}$), 1287, 1130 (SO$_2$) cm^{-1}; ^{1}H NMR (500 MHz, DMSO-*d*$_6$) δ: 0.90-1.04 (m, 2H, piperidine C3 and C5), 1.51-1.55 (m, 2H, piperidine C3 and C5), 1.72-1.77 (m, 1H, piperidine C4), 2.29 (s, 3H, CH$_3$Ph), 2.46-2.47 (m, 2H, piperidine-C**H**$_2$Ph), 2.67-2.72 (m, 2H, piperidine C2 and C6), 3.27 (t, *J=8.8 Hz*, 2H, imidazolidin), 3.43 (t, *J=8.8 Hz*, 2H, imidazolidin), 3.91 (s, 2H, S-CH$_2$), 4.34-4.35 (s, 2H, C**H**$_2$-C$_6$H$_5$), 4.53-4.61 (m, 2H, piperidine C2 and C6), 6.89-7.40 (m, 10H, H$_{Ar}$ and 1H, NH, imidazolidin and 2H, NH$_2$), 7.85 (s, 1H, H-3), 7.94 (s, 1H, H-6) ppm; Anal. calcd. for $C_{33}H_{37}ClN_8O_2S_2$ (677.28); C, 58.52; H, 5.51; N, 16.54. Found: C, 58.45; H, 5.50; N, 16.59; MALDI-TOF/TOF (676.2169) calcd for $C_{33}H_{37}ClN_8O_2S_2$ [M+H]$^+$ (677.2247) found 677.0613.

2-[{4-Amino-6-[4-(4-fluorophenyl)-5,6-dihydropyridin-1(2H)-yl]-1,3,5-triazin-2-yl}methylthio]-N-(1-benzylimidazolidin-2-ylidene)-4-chloro-5-methylbenzenesulfonamide (**73**).

Starting from *N*-carbamimidoyl-4-(4-fluorophenyl)-5,6-dihydropyridine-1(*2H*)-carboximidamide hydrochloride (0.476 g, 1.60 mmol). The title compound was obtained after refluxing with mixture 1:1 ethanol/acetonitrile (1:26) in an ultrasonic bath for 10 minutes. Yield 0.265 g (40%); m.p. 195–196 °C; IR (KBr): 3361, 3230 (N-H), 2963, 2925, 2883, 2861 (C-H), 1589, 1557, 1515, 1475 (C=N, C=C$_{Ar}$), 1264, 1124 (SO$_2$) cm^{-1}; ^{1}H NMR (500 MHz, DMSO-*d*$_6$ with TFA) δ: 2.31 (s, 3H, CH$_3$Ph), 2.39-2.43 (m, 2H, CH2$_{C5}$), 3.29

(t, *J=8.55 Hz*, 2H, imidazolidin), 3.45 (t, *J=8.80 Hz*, 2H, imidazolidin), 3.92-3.93 (m, 2H, $CH2_{C2}$), 4.17 (s, 2H, S-CH_2), 4.25-4.34 (m, 2H, $CH2_{C6}$), 4.37 (s, 2H, C**H**$_2$-C_6H_5), 6.03-6.09 (m, 1H, CH_{C3}), 7.07-7.41 (m, 9H, H_{Ar} and 1H, imidazolidin and 2H, NH_2), 7.83-7.88 (m, 1H, H-3 and 1H, H-6) ppm; Anal. calcd. for $C_{32}H_{32}ClFN_8O_2S_2$ (679.23); C, 56.58; H, 4.75; N, 16.50. Found: C, 56.33; H, 4.61; N, 16.32; MALDI-TOF/TOF (678.1762) calcd for $C_{32}H_{32}ClFN_8O_2S_2$ $[M+H]^+$ (679.1840) found 679.0156.

2-{[4-Amino-6-(indolin-1-yl)-1,3,5-triazin-2-yl]methylthio}-4-chloro-5-methyl-N-[1-(naphthalen-1-ylmethyl)imidazolidin-2-ylidene]benzenesulfonamide (**74**).

Starting from *N*-carbamimidoylindoline-1-carboximidamide hydrochloride (0.384 g, 1.60 mmol). The title compound was obtained by crystallization from ethanol (1:35). The precipitate was washed with 2 mL MeCN and 2 mL benzene. Yield 0.314 g (59%); m.p. 198–199 °C; IR (KBr): 3332, 3239, 3116 (N-H), 2982, 2911, 2881, 2854 (C-H), 1573, 1554, 1523, 1472 (C=N, C=C_{Ar}), 1277, 1131 (SO_2) cm^{-1}; ^1H NMR (500 MHz, DMSO-d_6 in 50°C) δ: 2.33 (s, 3H, CH_3), 3.01 (t, *J=8.05 Hz*, 2H, hydroindoline), 3.17 (t, *J=8.05 Hz*, 2H, imidazolidin), 3.38 (t, *J=8.55 Hz*, 2H, imidazolidin), 3.98 (t, *J=8.55 Hz*, 2H, hydroindoline), 4.04 (s, 2H, S-CH_2), 4.76-4.79 (m, 2H, C**H**$_2$-naphthyl), 6.90-8.40 (m, 7H, H_{Ar} (naphthyl), 4H, hydroindoline and 2H, NH_2 and 1H, NH, imidazolidin and 1H, H-3 and 1H, H-6) ppm; Anal. calcd. for $C_{33}H_{31}ClN_8O_2S_2$ (671.23); C, 59.05; H, 4.66; N, 16.69. Found: C, 58.98; H, 4.58; N, 16.58; MALDI-TOF/TOF (670.1700) calcd for $C_{33}H_{31}ClN_8O_2S_2$ $[M+H]^+$ (671.1778) found 671.1603.

2-{[4-Amino-6-(phenylamino)-1,3,5-triazin-2-yl]methylthio}-4-chloro-5-methyl-N-[1-(naphthalen-1-ylmethyl)imidazolidin-2-ylidene]benzenesulfonamide (**75**).

Starting from 1-phenylbiguanide hydrochloride (0.342 g, 1.60 mmol). The title compound was obtained after refluxing with ethanol (1:37) in an ultrasonic bath for 10 minutes. Yield 0.225 g (44%); m.p. 197–198 °C; IR (KBr): 3323, 3219 (N-H), 2961, 2918, 2859 (C-H), 1577, 1588, 1534, 1497 (C=N, C=C_{Ar}), 1278, 1157 (SO_2) cm^{-1}; ^1H NMR (500 MHz, DMSO-d_6 in 80°C) δ: 2.34 (s, 3H, CH_3Ph), 3.24 (t, *J=8.55 Hz*, 2H, imidazolidin), 3.43 (t, *J=8.55 Hz*, 2H, imidazolidin), 3.99 (s, 2H, S-CH_2), 4.82-4.84 (m, 2H, C**H**$_2$-naphthyl), 6.85-8.06 (m, 7H, H_{Ar} naphthyl and 5H, H_{Ar}, phenyl and 2H, NH_2 and 1H, NH, imidazolidin and 1H, H-3 and 1H, H-6), 9.24-9.27 (m, 1H, NH) ppm; Anal. calcd. for $C_{31}H_{29}ClN_8O_2S_2$ (645.20); C, 57.71; H, 4.53; N, 17.37. Found: C, 57.69; H, 4.50; N, 17.35; MALDI-TOF/TOF (644.1543) calcd for $C_{31}H_{29}ClN_8O_2S_2$ $[M+H]^+$ (645.1621) found 645.1182.

2-[{4-Amino-6-[(4-chlorophenyl)(methyl)amino]-1,3,5-triazin-2-yl}methylthio]-4-chloro-5-methyl-N-[1-(naphthalen-1-ylmethyl)imidazolidin-2-ylidene]benzenesulfonamide (**76**).

Starting from 1-(4-chlorophenyl)-1-methylbiguanide hydrochloride (0.419 g, 1.60 mmol). The title compound was obtained after refluxing with mixture 1:1 ethanol/dichloromethane (1:50) in an ultrasonic bath for 10 minutes. Yield 0.210 g (38%); m.p. 154–155 °C; IR (KBr): 3340, 3235 (N-H), 2947, 2922, 2855 (C-H), 1575, 1557, 1531, 1491 (C=N, C=C_{Ar}), 1273, 1130 (SO_2) cm^{-1}; ^1H NMR (500 MHz, DMSO-d_6) δ: 2.33 (s, 3H, CH_3Ph), 3.22 (t, *J=5.80 Hz*, 2H, imidazolidin), 3.32 (s, 3H, N-C**H**$_3$), 3.41 (t, *J=5.80 Hz*, 2H, imidazolidin), 3.91 (s, 2H, C**H**$_2$-naphthyl), 4.82 (s, 2H, S-CH_2), 7.00-7.05 (m, 2H, NH_2), 7.24-8.04 (m, 7H, naphthyl and 4H, C_6**H**$_4$-4-Cl and 1H, NH, imidazolidin and 1H, H-3 and 1H, H-6) ppm; Anal. calcd. for $C_{32}H_{30}Cl_2N_8O_2S_2$ (693.67); C, 55.41; H, 4.36; N, 16.15. Found: C, 55.18; H, 4.23; N, 15.84; MALDI-TOF/TOF (692.1310) calcd for $C_{32}H_{30}Cl_2N_8O_2S_2$ $[M+H]^+$ (693.1388) found 693.1048.

2-{[4-Amino-6-(4-phenylpiperazin-1-yl)-1,3,5-triazin-2-yl]methylthio}-4-chloro-5-methyl-N-[1-(naphthalen-1-ylmethyl)imidazolidin-2-ylidene]benzenesulfonamide (**77**).

Starting from *N*-carbamimidoyl-4-phenylpiperazine-1-carboximidamide hydrochloride (0.452 g, 1.60 mmol). The title compound was obtained after refluxing with ethanol (1:33) in an ultrasonic bath for 10 minutes. Next all solid was crystallization from the mixture 2:1 ethanol/ *N,N*-dimethylformamide (1:34), remained part by crystallization from filtrate. Yield 0.243 g (42.56%); m.p. 230–231 °C; IR (KBr): 3328, 3230 (N-H), 2957, 2914, 2890, 2856 (C-H), 1584, 1562, 1553, 1523 (C=N, C=C_{Ar}), 1275, 1129 (SO_2) cm-

1; ^{1}H NMR (500 MHz, DMSO-d_6) δ: 2.33 (s, 3H, CH_3), 3.08-3.12 (m, 4H, piperazine), 3.20 (t, *J=8.55 Hz*, 2H, imidazolidin), 3.36-3.40 (m, 2H, imidazolidin), 3.71-3.79 (m, 4H, piperazine), 3.95 (s, 2H, S-CH_2), 4.83 (s, 2H, C**H**$_2$-naphthyl), 6.78-8.07 (m, 12H, H_{Ar} and 2H, NH_2 and 1H, NH, imidazolidin and 1H, H-3 and 1H, H-6) ppm; Anal. calcd. for $C_{35}H_{36}ClN_9O_2S_2$ (714.30); C, 58.85; H, 5.08; N, 17.65. Found: C, 58.51; H, 5.06; N, 17.36; MALDI-TOF/TOF (713.2122) calcd for $C_{35}H_{36}ClN_9O_2S_2$ $[M+H]^+$ (714.2200) found 714.1625.

2-{[4-Amino-6-{4-[4-(trifluoromethyl)phenyl]piperazin-1-yl}-1,3,5-triazin-2-yl]methylthio}-4-chloro-5-methyl-N-[1-(naphthalen-1-ylmethyl)imidazolidin-2-ylidene]-benzenesulfonamide (**78**).

Starting from *N*-carbamimidoyl-4-[4-(trifluoromethyl)phenyl]piperazine-1-carboximidamide hydrochloride (0.561 g, 1.60 mmol). The title compound was obtained after refluxing with mixture 3:1 ethanol/acetonitrile (1:47) in an ultrasonic bath for 10 minutes. Next all solid was crystallization from the mixture 3:1 ethanol/ *N,N*-dimethylformamide (1:37), remained part by crystallization from filtrate. Yield 0.334 g (53%); m.p. 231–232 °C; IR (KBr): 3336, 3234 (N-H), 2991, 2960, 2918, 2857 (C-H), 1586, 1555, 1526, 1487 (C=N, $C=C_{Ar}$), 1272, 1127 (SO_2) cm^{-1}; ^{1}H NMR (500 MHz, DMSO-d_6) δ: 2.33 (s, 3H, CH_3Ph), 3.09-3.31 (m, 2H, imidazolidin and 4H, piperazine), 3.39 (t, *J=8.8 Hz*, 2H, imidazolidin), 3.72-3.78 (m, 4H, piperazine), 3.95 (s, 2H, S-CH_2), 4.83 (s, 2H, C**H**$_2$-naphthyl), 6.98-8.07 (m, 7H, naphthyl and 4H, C_6**H**$_4$-4-CF_3 and 2H, NH_2 and 1H, NH, imidazolidin and 1H, H-3 and 1H, H-6) ppm; Anal. calcd. for $C_{36}H_{35}ClF_3N_9O_2S_2$ (782.30); C, 55.27; H, 4.51; N, 16.11. Found: C, 55.31; H, 4.54; N, 16.11; MALDI-TOF/TOF (781.1996) calcd for $C_{36}H_{35}ClF_3N_9O_2S_2$ $[M+H]^+$ (782.2074) found 782.2306.

2-[{4-Amino-6-[4-(4-chlorophenyl)piperazin-1-yl]-1,3,5-triazin-2-yl}methylthio]-4-chloro-5-methyl-N-[1-(naphthalen-1-ylmethyl)imidazolidin-2-ylidene]benzenesulfonamide (**79**).

Starting from *N*-carbamimidoyl-4-(4-chlorophenyl)piperazine-1-carboximidamide hydrochloride (0.508 g, 1.60 mmol). The title compound was obtained after refluxing with mixture 1:1 ethanol/acetonitrile (1:41) in an ultrasonic bath for 10 minutes. Yield 0.342 g (57%); m.p. 222–223 °C; IR (KBr): 3333, 3232 (N-H), 2957, 2916, 2884, 2854 (C-H), 1587, 1556, 1526, 1497 (C=N, $C=C_{Ar}$), 1272, 1127 (SO_2) cm^{-1}; ^{1}H NMR (500 MHz, DMSO-d_6) δ: 2.33 (s, 2H, CH_3Ph), 2.91-3.09 (m, 4H, piperazine), 3.20 (t, *J=8.8 Hz*, 2H, imidazolidin), 3.39 (t, *J=8.8 Hz*, 2H, imidazolidin), 3.71-3.78 (m, 4H, piperazine), 3.95 (s, 2H, S-CH_2), 4.83 (s, 2H, C**H**$_2$-naphthyl), 6.86-8.07 (m, 7H, H_{Ar} naphthyl and 4H, H_{Ar} phenyl and 2H, NH_2 and 1H, NH, imidazolidin and 1H, H-3 and 1H, H-6) ppm; Anal. calcd. for $C_{35}H_{35}Cl_2N_9O_2S_2$ (748.75); C, 56.14; H, 4.71; N, 16.84. Found: C, 55.81; H, 4.58; N, 16.59; MALDI-TOF/TOF (747.1732) calcd for $C_{35}H_{35}Cl_2N_9O_2S_2$ $[M+H]^+$ (748.1810) found 747.9744.

2-[{4-Amino-6-[4-(3,4-dichlorophenyl)piperazin-1-yl]-1,3,5-triazin-2-yl}methylthio]-4-chloro-5-methyl-N-[1-(naphthalen-1-ylmethyl)imidazolidin-2-ylidene]benzenesulfonamide (**80**).

Starting from *N*-carbamimidoyl-4-(3,4-dichlorophenyl)piperazine-1-carboximidamide hydrochloride (0.563 g, 1.60 mmol). The title compound was obtained after refluxing with ethanol (1:35) in an ultrasonic bath for 10 minutes and next all was washed 2 mL acetonitrile and 2 mL diethyl ether. Yield 0.398 g (64%); m.p. 179–181 °C; IR (KBr): 3397, 3334, 3227 (N-H), 2947, 1918, 2880, 2855 (C-H), 1591, 1557, 1515, 1484 (C=N, $C=C_{Ar}$), 1267, 1143 (SO_2) cm^{-1}; ^{1}H NMR (500 MHz, DMSO-d_6 in 80 °C) δ: 2.35 (s, 2H, CH_3Ph), 3.07-3.12 (m, 4H, piperazine), 3.24 (t, *J=8.8 Hz*, 2H, imidazolidin), 3.43 (t, *J=8.8 Hz*, 2H, imidazolidin), 3.74-3.76 (m, 4H, piperazine), 3.93 (s, 2H, S-CH_2), 4.85 (s, 2H, C**H**$_2$-naphthyl), 6.71-8.10 (m, 7H, H_{Ar} naphthyl and 3H, H_{Ar} and 2H, NH_2 and 1H, NH, imidazolidin and 1H, H-3 and 1H, H-6) ppm; Anal. calcd. for $C_{35}H_{34}Cl_3N_9O_2S_2$ (783.19); C, 53.67; H, 4.38; N, 16.10. Found: C, 53.72; H, 4.41; N, 16.13; MALDI-TOF/TOF (781.1342) calcd for $C_{35}H_{34}Cl_3N_9O_2S_2$ $[M+H]^+$ (782.1420) found 782.0742.

2-{[4-Amino-6-(4-benzylpiperazin-1-yl)-1,3,5-triazin-2-yl]methylthio}-4-chloro-5-methyl-N-[1-(naphthalen-1-ylmethyl)imidazolidin-2-ylidene]benzenesulfonamide (**81**).

Starting from 4-benzyl-*N*-carbamimidoylpiperazine-1-carboximidamide hydrochloride (0.475 g, 1.60 mmol). The title compound was obtained after refluxing with ethanol (1:40) in an ultrasonic bath

for 10 minutes. Yield 0.276 g (47%); m.p. 136–137 °C; IR (KBr): 3301, 3214, 3139 (N-H), 2917, 2892, 2861, 2810 (C-H), 1588, 1562, 1517, 1455 (C=N, C=C_{Ar}), 1274, 1130 (SO_2) cm^{-1}; ^1H NMR (500 MHz, DMSO-d_6 in 70 °C) δ: 2.24-2.30 (m, 4H, piperazine), 2.32 (s, 3H, CH_3Ph), 3.24 (t, *J=8.8 Hz*, 2H, imidazolidin), 3.41-3.43 (m, 2H, imidazolidin and 2H, CH_2-Ph), 3.60-3.65 (m, 4H, piperazine), 3.90 (s, 2H, S-CH_2), 4.82 (s, 2H, C**H**$_2$-naphthyl), 6.99-8.08 (m, 7H, H_{Ar} naphthyl and 5H, H_{Ar} phenyl, 2H, NH_2 and 1H, NH, imidazolidin and 1H, H-3 and 1H, H-6) ppm; Anal. calcd. for $C_{36}H_{38}ClN_9O_2S_2$ (728.3290); C, 59.37; H, 5.26; N, 17.31. Found: C, 59.24; H, 5.15; N, 17.00; MALDI-TOF/TOF (727.2278) calcd for $C_{36}H_{38}ClN_9O_2S_2$ $[M+H]^+$ (728.2356) found 728.0475.

2-[(4-Amino-6-morpholino-1,3,5-triazin-2-yl)methylthio]-4-chloro-N-[1-(4-fluorobenzyl)imidazolidin-2-ylidene]-5-methylbenzenesulfonamide (**82**).

Starting from *N*-carbamimidoylmorpholine-4-carboximidamide hydrochloride (0.332 g, 1.60 mmol). The title compound was obtained after refluxing with ethanol (1:36) in an ultrasonic bath for 10 minutes. Yield 0.259 g (53%); m.p. 202–204 °C; IR (KBr): 3391, 3322, 3226 (N-H), 2961, 2941, 2918, 2855 (C-H), 1584, 1546, 1530, 1482 (C=N, C=C_{Ar}), 1264, 1131 (SO_2) cm^{-1}; ^1H NMR (500 MHz, DMSO-d_6 in T=70 °C) δ: 2.33 (s, 3H, CH_3), 3.31 (t, *J=8.7 Hz*, 2H, imidazolidin), 3.46 (t, *J=8.7 Hz*, 2H, imidazolidin), 3.56 (t, *J=4.6 Hz*, 4H, morpholine), 3.66 (t, *J=4.75 Hz*, 4H, morpholine), 3.93 (s, 2H, S-CH_2), 4.36 (s, 2H, C**H**$_2$-C_6H_4-4-F), 6.75-7.30 (m, 4H, H_{Ar} and 2H, NH_2 and 1H, NH, imidazolidin), 7.85 (s, 1H, H-3), 7.89 (s, 1H, H-6) ppm; Anal. calcd. for $C_{25}H_{28}ClFN_8O_3S_2$ (607.12); C, 49.46; H, 4.65; N, 18.46. Found: C, 49.32; H, 4.55; N, 18.15; MALDI-TOF/TOF (606.1398) calcd for $C_{25}H_{28}ClFN_8O_3S_2$ $[M+H]^+$ (607.1476) found 607.1006.

2-{[4-Amino-6-(indolin-1-yl)-1,3,5-triazin-2-yl]methylthio}-4-chloro-N-[1-(4-fluorobenzyl)imidazolidin-2-ylidene]-5-methylbenzenesulfonamide (**83)**.

Starting from *N*-carbamimidoylindoline-1-carboximidamide hydrochloride (0.384 g, 1.60 mmol). The title compound was obtained by crystallization from ethanol (1:23). Yield 0.316 g (62%); m.p. 188–189 °C; IR (KBr): 3369, 3317, 3116 (N-H), 2975, 2889, 2859 (C-H), 1589, 1517 (C=N, C=C_{Ar}), 1258, 1129 (SO_2) cm^{-1}; ^1H NMR (500 MHz, DMSO-d_6) δ: 2.31 (s, 3H, CH_3), 3.08 (t, *J=8.3 Hz*, 2H, hydroindoline), 3.18-3.26 (m, 2H, imidazolidin), 3.36-3.42 (m, 2H, imidazolidin), 4.06 (t, *J=8.5 Hz*, 2H, hydroindoline and 2H, S-CH_2), 4.26-4.35 (m, 2H, C**H**$_2$-C_6H_4-4-F), 6.92-8.60 (m, 8H, H_{Ar} and 2H, NH_2 and 1H, NH, imidazolidin and 1H, H-3 and 1H, H-6) ppm; Anal. calcd. for $C_{29}H_{28}ClFN_8O_2S_2$ (639.17); C, 54.49; H, 4.42; N, 17.53. Found: C, 54.45; H, 4.40; N, 17.53; MALDI-TOF/TOF (638.1449) calcd for $C_{29}H_{28}ClFN_8O_2S_2$ $[M+H]^+$ (639.1527) found 638.9605.

2-{[4-Amino-6-(3,4-dihydroquinolin-1(2H)-yl)-1,3,5-triazin-2-yl]methylthio}-4-chloro-N-[1-(4-fluorobenzyl)imidazolidin-2-ylidene]-5-methylbenzenesulfonamide (**84**).

Starting from *N*-carbamimidoyl-3,4-dihydroquinoline-1(2*H*)-carboximidamide hydrochloride (0.406 g, 1.60 mmol). The title compound was obtained after refluxing with ethanol (1:38) in an ultrasonic bath for 10 minutes. Yield 0.160 g (31%); m.p. 109–110 °C; IR (KBr): 3409, 3178 (N-H), 2968, 2952, 2926, 2896 (C-H), 1581, 1564, 1510, 1489 (C=N, C=C_{Ar}), 1274, 1130 (SO_2)cm^{-1}; ^1H NMR (500 MHz, DMSO-d_6 in 70 °C) δ: 1.85 (quintet, *J=6.0 Hz*, 2H, 1,2,3,4-tetrahydroquinoline), 2.33 (s, 3H, CH_3), 2.71 (t, *J=6.7 Hz*, 2H, 1,2,3,4-tetrahydroquinoline), 3.29 (t, *J=8.8 Hz*, 2H, imidazolidin), 3.43 (t, *J=8.75 Hz*, 2H, imidazolidin), 3.90 (t, *J=6.1 Hz*, 2H, 1,2,3,4-tetrahydroquinoline), 3.98 (s, 2H, S-CH_2), 4.34 (s, 2H, C**H**$_2$-C_6H_4-4-F), 6.86-7.25 (m, 8H, H_{Ar} and 2H, NH_2 and 1H, NH, imidazolidin), 7.77 (s, 1H, H-3), 7.86 (s, 1H, H-6) ppm; Anal. calcd. for $C_{30}H_{30}ClFN_8O_2S_2$ (653.19); C, 55.16; H, 4.63; N, 17.15. Found: C, 55.12; H, 4.60; N, 17.15; MALDI-TOF/TOF (652.1606) calcd for $C_{30}H_{30}ClFN_8O_2S_2$ $[M+H]^+$ (653.1684) found 653.1461.

{[4-Amino-6-(phenylamino)-1,3,5-triazin-2-yl]methylthio}-4-chloro-N-[1-(4-fluorobenzyl)imidazolidin-2-ylidene]-5-methylbenzenesulfonamide (**85**).

Starting from 1-phenylbiguanide hydrochloride (0.342 g, 1.60 mmol). The title compound was obtained after refluxing with ethanol (1:23) in an ultrasonic bath for 10 minutes and next refluxing with

acetonitrile (1:25) in an ultrasonic bath for 10 minutes. Yield 0.284 g (58%); m.p. 209–210 °C; IR (KBr): 3318, 3214 (N-H), 2958, 2928, 2892, 2856 (C-H), 1576, 1535, 1508, 1496 (C=N, C=C_{Ar}), 1273, 1129 (SO_2) cm^{-1}; ^1H NMR (500 MHz, DMSO-d_6) δ: 2.31 (s, 3H, CH_3Ph), 3.23-3.26 (m, 2H, imidazolidin), 3.41 (t, *J=8.5 Hz*, 2H, imidazolidin), 4.04 (s, 2H, S-CH_2), 4.31-4.34 (m, 2H, C**H**2-C_6H_4-4-F), 6.94-7.74 (m, 9H, H_{Ar} and 2H, NH_2 and 1H, NH, imidazolidin and 1H, H-3), 7.88 (s, 1H, H-6), 9.49-9.62 (m, 1H, NH) ppm; Anal. calcd. for $C_{27}H_{26}ClFN_8O_2S_2$ (613.13); C, 52.89; H, 4.27; N, 18.28. Found: C, 52.50; H, 4.20; N, 18.17; MALDI-TOF/TOF (612.1293) calcd for $C_{27}H_{26}ClFN_8O_2S_2$ $[M+H]^+$ (613.1371) found 612.9694.

2-[{4-Amino-6-[(4-fluorophenyl)amino]-1,3,5-triazin-2-yl}methylthio}-4-chloro-N-[1-(4-fluorobenzyl)imidazolidin-2-ylidene]-5-methylbenzenesulfonamide (**86**).

Starting from 1-(4-fluorophenyl)biguanide hydrochloride (0.371 g, 1.60 mmol). The title compound was obtained after refluxing with ethanol (1:35) in an ultrasonic bath for 10 minutes. Yield 0.265 g (51%); m.p. 220–221 °C; IR (KBr): 3320, 3206 (N-H), 2967, 2928, 2898, 2866 (C-H), 1592, 1538, 1506 (C=N, C=C_{Ar}), 1275, 1156 (SO_2) cm^{-1}; ^1H NMR (500 MHz, DMSO-d_6) δ: 2.31 (s, 3H, CH_3Ph), 3.25-3.26 (m, 2H, imimidazolidin), 3.39-3.41 (m, 2H, imimidazolidin), 4.03 (s, 2H, S-CH_2), 4.33 (s, 2H, C**H**2-C_6H_4-4-F), 7.06-7.73 (m, 8H, H_{Ar} and 2H, NH_2 and 1H, H-3), 7.73 (s, 1H, H-6), 9.51-9.66 (m, 1H, NH) ppm; Anal. calcd. for $C_{27}H_{25}ClF_2N_8O_2S_2$ (631.12); C, 51.38; H, 3.99; N, 17.75. Found: C, 50.98; H, 3.80; N, 17.43; MALDI-TOF/TOF (630.1198) calcd for $C_{27}H_{25}ClF_2N_8O_2S_2$ $[M+H]^+$ (631.1276) found 631.1318.

2-{[4-Amino-6-{[4-(trifluoromethyl)phenyl]amino}-1,3,5-triazin-2-yl]methylthio}-4-chloro-N-[1-(4-fluorobenzyl)imidazolidin-2-ylidene]-5-methylbenzenesulfonamide (**87**).

Starting from 1-[4-(trifluoromethyl)phenyl]biguanide hydrochloride (0.451 g, 1.60 mmol). The title compound was obtained after mixing with ethanol (1:30) in an ultrasonic bath for 10 minutes in r.t. Yield 0.189 g (35%); m.p. 216–217 °C; IR (KBr): 3319, 3210 (N-H), 2962, 2929, 2900, 2859 (C-H), 1595, 1552, 1526 (C=N, C=C_{Ar}), 1269, 1138 (SO_2) cm^{-1}; ^1H NMR (500 MHz, DMSO-d_6) δ: 2.31 (s, 3H, CH_3Ph), 3.23 (t, *J=8.8 Hz*, 2H, imidazolidin), 3.40 (t, *J=8.55 Hz*, 2H, imidazolidin), 4.09 (s, 2H, S-CH_2), 4.31 (s, 2H, C**H**2-C_6H_4-4-F), 7.05-7.94 (m, 8H, H_{Ar} and 2H, NH_2 and 1H, NH, imidazolidin and 1H, H-3 and 1H, H-6), 9.90-9.98 (m, 1H, N**H**-C_6H_4-4-CF_3) ppm; Anal. calcd. for $C_{28}H_{25}ClF_4N_8O_2S_2$ (681.13); C, 49.37; H, 3.70; N, 16.45. Found: C, 49.22; H, 3.59; N, 16.10; MALDI-TOF/TOF (680.1167) calcd for $C_{28}H_{25}ClF_4N_8O_2S_2$ $[M+H]^+$ (681.1245) found 681.1454.

2-[{4-Amino-6-[(4-methoxyphenyl)amino]-1,3,5-triazin-2-yl}methylthio]-4-chloro-N-[1-(4-fluorobenzyl)imidazolidin-2-ylidene]-5-methylbenzenesulfonamide (**88**).

Starting from 1-(4-methoxyphenyl)biguanide hydrochloride (0.390 g, 1.60 mmol). The title compound was obtained after refluxing with ethanol (1:33) in an ultrasonic bath for 10 minutes. Yield 0.287 g (57%); m.p. 231–232 °C; IR (KBr): 3319, 3213 (N-H), 2957, 2933, 2836 (C-H), 1596, 1537, 1508 (C=N, C=C_{Ar}), 1239, 1164 (SO_2) cm^{-1}; ^1H NMR (500 MHz, DMSO-d_6) δ: 2.32 (s, 3H, CH_3Ph), 3.21-3.28 (m, 2H, imimidazolidin), 3.37-3.44 (m, 2H, imimidazolidin), 3.70 (s, 3H, O-C**H**3), 4.01 (br.s, 2H, S-CH_2), 4.28-4.37 (m, 2H, C**H**2-C_6H_4-4-F), 6.80-7.67 (m, 8H, H_{Ar} and 1H, NH_2 and 1H, NH, imidazolidin and 1H, H-3), 7.88 (s, 1H, H-6), 9.25-9.55 (m, 1H, N**H**-C_6H_4-4-OMe) ppm; Anal. calcd. for $C_{28}H_{28}ClFN_8O_3S_2$ (643.16); C, 52.29; H, 4.39; N, 17.42. Found: C, 51.97; H, 4.32; N, 17.21; MALDI-TOF/TOF (642.1398) calcd for $C_{28}H_{28}ClFN_8O_3S_2$ $[M+H]^+$ (643.1476) found 643.0997.

2-[{4-Amino-6-[methyl(phenyl)amino]-1,3,5-triazin-2-yl}methylthio]-4-chloro-N-[1-(4-fluorobenzyl)imidazolidin-2-ylidene]-5-methylbenzenesulfonamide (**89**).

Starting from 1-methyl-1-phenylbiguanide hydrochloride (0.364 g, 1.60 mmol). The title compound was obtained after refluxing with ethanol (1:34) in an ultrasonic bath for 10 minutes. Yield 0.309 g (62%); m.p. 193–194 °C; IR (KBr): 3379, 3120 (N-H), 2936, 2919, 2894, 2855 (C-H), 1589, 1565, 1526, 1493 (C=N, C=C_{Ar}), 1264, 1129 (SO_2) cm^{-1}; ^1H NMR (500 MHz, DMSO-d_6) δ: 2.32 (s, 3H, CH_3Ph), 3.26-3.30 (m, 2H, imidazolidin), 3.37 (s, 3H, N-C**H**3), 3.41-3.45 (m, 2H, imidazolidin), 3.94 (s, 2H, S-CH_2), 4.35 (s, 2H, C**H**2-

C_6H_4-4-F), 6.98-7.42 (m, 9H, H_{Ar} and 2H, NH_2 and 1H, NH, imidazolidin), 7.82 (s, 1H, H-3), 7.86 (s, 1H, H-6) ppm; Anal. calcd. for $C_{28}H_{28}ClFN_8O_2S_2$ (627.16); C, 53.62; H, 4.50; N, 17.87. Found: C, 53.22; H, 4.23; N, 17.62; MALDI-TOF/TOF (626.1449) calcd for $C_{28}H_{28}ClFN_8O_2S_2$ [M+H]$^+$ (627.1527) found 627.1519.

2-[{4-Amino-6-[(4-chlorophenyl)(methyl)amino]-1,3,5-triazin-2-yl}methylthio]-4-chloro-N-[1-(4-fluorobenzyl)imidazolidin-2-ylidene]-5-methylbenzenesulfonamide (**90**).

Starting from 1-(4-chlorophenyl)-1-methylbiguanide hydrochloride (0.419 g, 1.60 mmol). The title compound was obtained after refluxing with ethanol (1:30) in an ultrasonic bath for 10 minutes. Yield 0.349 g (66%); m.p. 212–213 °C; IR (KBr): 3382, 3330, 3161 (N-H), 2981, 2945, 2891, 2854 (C-H), 1591, 1561, 1523, 1491 (C=N, C=C_{Ar}), 1270, 1155 (SO_2) cm^{-1}; ^{1}H NMR (500 MHz, DMSO-d_6) δ: 2.32 (s, 3H, CH_3Ph), 3.28 (t, *J=8.55 Hz*, 2H, imidazolidin), 3.36 (s, 3H, N-C**H**$_3$), 3.43 (t, *J=8.55 Hz*, 2H, imidazolidin), 3.95 (s, 2H, S-CH_2), 4.34-4.35 (m, 2H, C**H**$_2$-C_6H_4-4-F), 7.02-7.42 (m, 8H, H_{Ar} and 2H, NH_2 and 1H, NH, imidazolidin), 7.79 (s, 1H, H-3), 7.86 (s, 1H, H-6) ppm; Anal. calcd. for $C_{28}H_{27}Cl_2FN_8O_2S_2$ (661.60); C, 50.83; H, 4.11; N, 16.94. Found: C, 50.80; H, 4.10; N, 16.91; MALDI-TOF/TOF (660.1059) calcd for $C_{28}H_{27}Cl_2FN_8O_2S_2$ [M+H]$^+$ (661.1137) found 661.0535.

2-{[4-Amino-6-(4-methylpiperazin-1-yl)-1,3,5-triazin-2-yl]methylthio}-4-chloro-N-[1-(4-fluorobenzyl)imidazolidin-2-ylidene]-5-methylbenzenesulfonamide (**91**).

Starting from *N*-carbamimidoyl-4-methylpiperazine-1-carboximidamide hydrochloride (0.353 g, 1.60 mmol). The title compound was obtained by evaporation post-reaction mixture and next add 4 mL ethanol and after dissolved solid, add 50 mL water. All was mixture 30 min and filtered. Yield 0.303 g (69%); m.p. 168–169 °C. IR (KBr): 3396, 3301, 3137 (N-H), 2978, 2941, 2860, 2799 (C-H), 1584, 1515, 1451 (C=N, C=C_{Ar}), 1259, 1144 (SO_2) cm^{-1}; ^{1}H NMR (500 MHz, DMSO-d_6 in 70 °C) δ: 2.18 (s, 3H, C**H**$_3$-piperazine), 2.24-2.28 (m, 4H, piperazine), 2.33 (s, 3H, CH_3Ph), 3.31 (t, *J=8.7 Hz*, 2H, imidazolidin), 3.46 (t, *J=8.65 Hz*, 2H, imidazolidin), 3.65-3.67 (m, 4H, piperazine), 3.92 (s, 2H, S-CH_2), 4.36 (s, 2H, C**H**$_2$-C_6H_4-4-F), 6.70-7.30 (m, 4H, H_{Ar} and 2H, NH_2 and 1H, NH, imidazolidin), 7.85 (s, 1H, H-3), 7.90 (s, 1H, H-6) ppm; Anal. calcd. for $C_{26}H_{31}ClFN_9O_2S_2$ (620.16); C, 50.35; H, 5.04; N, 20.33. Found: C, 49.97; H, 4.81; N, 19.93; MALDI-TOF/TOF (619.1715) calcd for $C_{26}H_{31}ClFN_9O_2S_2$ [M+H]$^+$ (620.1793) found 620.0363.

2-{[4-Amino-6-(4-phenylpiperazin-1-yl)-1,3,5-triazin-2-yl]methylthio}-4-chloro-N-[1-(4-fluorobenzyl)imidazolidin-2-ylidene]-5-methylbenzenesulfonamide (**92**).

Starting from *N*-carbamimidoyl-4-phenylpiperazine-1-carboximidamide hydrochloride (0.452 g, 1.60 mmol). The title compound was obtained by crystallization from ethanol (1:31). Yield 0.363 g (67%); m.p. 208–209 °C; IR (KBr): 3348, 3244 (N-H), 2972, 2910, 2858, 2815 (C-H), 1595, 1565, 1546, 1519 (C=N, C=C_{Ar}), 1263, 1157 (SO_2) cm^{-1}; ^{1}H NMR (500 MHz, DMSO-d_6) δ: 2.32 (s, 3H, CH_3Ph), 3.07-3.10 (m, 4H, piperazine), 3.27 (t, *J=8.8 Hz*, 2H, imidazolidin), 3.42 (t, *J=8.75 Hz*, 2H, imidazolidin), 3.80-3.84 (m, 4H, piperazine), 3.97 (s, 2H, S-CH_2), 4.35 (s, 2H, C**H**$_2$-C_6H_4-4-F), 6.80-7.42 (m, 9H, H_{Ar} and 2H, NH_2 and 1H, NH, imidazolidin), 7.86 (s, 1H, H-3), 7.95 (s, 1H, H-6) ppm; Anal. calcd. for $C_{31}H_{33}ClFN_9O_2S_2$ (682.23); C, 54.58; H, 4.88; N, 18.48. Found: C, 54.52; H, 4.85; N, 18.48; MALDI-TOF/TOF (681.1871) calcd for $C_{31}H_{33}ClFN_9O_2S_2$ [M+H]$^+$ (682.1949) found 681.9681.

2-[{4-Amino-6-[4-(4-fluorophenyl)piperazin-1-yl]-1,3,5-triazin-2-yl}methylthio]-4-chloro-N-[1-(4-fluorobenzyl)imidazolidin-2-ylidene]-5-methylbenzenesulfonamide (**93**).

Starting from *N*-carbamimidoyl-4-(4-fluorophenyl)piperazine-1-carboximidamide hydrochloride (0.481 g, 1.60 mmol). The title compound was obtained after refluxing with ethanol (1:32) in an ultrasonic bath for 10 minutes. Yield 0.358 g (64%); m.p. 189–190 °C; IR (KBr): 3354, 3246 (N-H), 2922, 2910, 2877 (C-H), 1595, 1561, 1508 (C=N, C=C_{Ar}), 1238, 1158 (SO_2) cm^{-1}; ^{1}H NMR (500 MHz, DMSO-d_6) δ: 2.31 (s, 3H, CH_3Ph), 3.01-3.04 (m, 4H, piperazine), 3.27 (t, *J=8.8 Hz*, 2H, imidazolidin), 3.42 (t, *J=8.75 Hz*, 2H, imidazolidin), 3.79-3.85 (m, 4H, piperazine), 3.97 (s, 2H, S-CH_2), 4.35 (s, 2H, C**H**$_2$-C_6H_4-4-F), 6.95-7.42 (m, 8H, H_{Ar} and 2H, NH_2 and 1H, NH, imidazolidin), 7.86 (s, 1H, H-3), 7.95 (s, 1H, H-6) ppm; Anal.

calcd. for $C_{31}H_{32}ClF_2N_9O_2S_2$ (700.22); C, 53.17; H, 4.61; N, 18.00. Found: C, 53.15; H, 4.59; N, 18.00; MALDI-TOF/TOF (699.1777) calcd for $C_{31}H_{32}ClF_2N_9O_2S_2$ $[M+H]^+$ (700.1855) found 700.1724.

2-[{4-Amino-6-[4-(2-fluorophenyl)piperazin-1-yl]-1,3,5-triazin-2-yl}methylthio]-4-chloro-N-[1-(4-fluorobenzyl)imidazolidin-2-ylidene]-5-methylbenzenesulfonamide (**94**).

Starting from *N*-carbamimidoyl-4-(2-fluorophenyl)piperazine-1-carboximidamide hydrochloride (0.481 g, 1.60 mmol). The title compound was obtained after refluxing with ethanol (1:31) in an ultrasonic bath for 10 minutes. Yield 0.361 g (65%); m.p. 203–204 °C; IR (KBr): IR (cm^{-1}): 3347, 3245 (N-H), 2967, 2903, 2920, 2865 (C-H), 1594, 1569, 1544, 1499 (C=N, $C=C_{Ar}$), 1257, 1162 (SO_2) cm^{-1}; ^1H NMR (500 MHz, DMSO-d_6) δ: 2.31 (s, 3H, CH_3Ph), 2.92-2.98 (m, 4H, piperazine), 3.27 (t, *J=8.8 Hz*, 2H, imidazolidin), 3.42 (t, *J=8.75 Hz,* 2H, imidazolidin), 3.81-3.85 (m, 4H, piperazine), 3.97 (s, 2H, S-CH_2), 4.35 (s, 2H, C**H**$_2$-C_6H_4-4-F), 6.98-7.42 (m, 8H, H_{Ar} and 2H, NH_2 and 1H, NH, imidazolidin), 7.86 (s, 1H, H-3), 7.94 (s, 1H, H-6) ppm; Anal. calcd. for $C_{31}H_{32}ClF_2N_9O_2S_2$ (700.22); C, 53.17; H, 4.61; N, 18.00. Found: C, 53.14; H, 4.60; N, 18.00; MALDI-TOF/TOF (699.1777) calcd for $C_{31}H_{32}ClF_2N_9O_2S_2$ $[M+H]^+$ (700.1855) found 700.0157.

2-{[4-Amino-6-{4-[4-(trifluoromethyl)phenyl]piperazin-1-yl}-1,3,5-triazin-2-yl]methylthio}-4-chloro-N-[1-(4-fluorobenzyl)imidazolidin-2-ylidene]-5-methylbenzenesulfonamide (**95**).

Starting from *N*-carbamimidoyl-4-[3-(trifluoromethyl)phenyl]piperazine-1-carboximidamide hydrochloride (0.561 g, 1.60 mmol). The title compound was obtained after refluxing with ethanol (1:31) in an ultrasonic bath for 10 minutes. Yield 0.422 g (73%); m.p. 236–237 °C; IR (KBr): 3341, 3221 (N-H), 2946, 2920, 2881, 2833 (C-H), 1598, 1523 (C=N, $C=C_{Ar}$), 1268, 1128 (SO_2) cm^{-1}; ^1H NMR (500 MHz, DMSO-d_6) δ: 2.32 (s, 3H, CH_3Ph), 3.26-3.29 (m, 4H, piperazine and 2H, imidazolidin), 3.42 (t, *J=8.75 Hz*, 2H, imidazolidin), 3.80-3.85 (m, 4H, piperazine), 3.97 (s, 2H, S-CH_2), 4.36 (s, 2H, C**H**$_2$-C_6H_4-4-F), 7.00-7.54 (m, 8H, H_{Ar} and 2H, NH_2 and 1H, NH, imidazolidin), 7.86 (s, 1H, H-3), 7.95 (s, 1H, H-6) ppm; Anal. calcd. for $C_{32}H_{32}ClF_4N_9O_2S_2$ (750.23); C, 51.23; H, 4.30; N, 16.80. Found: C, 51.17; H, 4.20; N, 16.71; MALDI-TOF/TOF (749.1745) calcd for $C_{32}H_{32}ClF_4N_9O_2S_2$ $[M+H]^+$ (750.1823) found 750.0207.

2-[{4-Amino-6-[4-(4-chlorophenyl)piperazin-1-yl]-1,3,5-triazin-2-yl}methylthio]-4-chloro-N-[1-(4-fluorobenzyl)imidazolidin-2-ylidene]-5-methylbenzenesulfonamide (**96**).

Starting from *N*-carbamimidoyl-4-(4-chlorophenyl)piperazine-1-carboximidamide hydrochloride (0.508 g, 1.60 mmol). The title compound was obtained after refluxing with ethanol (1:33) in an ultrasonic bath for 10 minutes. Yield 0.358 g (63%); m.p. 194–195 °C; IR (KBr): 3386, 3344, 3221 (N-H), 2919, 2878, 2829 (C-H), 1598, 1557, 1519, 1512 (C=N, $C=C_{Ar}$), 1270, 1157 cm^{-1}; ^1H NMR (500 MHz, DMSO-d_6) δ: 2.31 (s, 3H, CH_3Ph), 3.07-3.12 (m, 4H, piperazine), 3.27 (t, *J=5.7 Hz,* 2H, imidazolidin), 3.42 (t, *J=5.9 Hz*, 2H, imidazolidin), 3.79-3.84 (m, 4H, piperazine), 3.96 (s, 2H, S-CH_2), 4.35 (s, 2H, C**H**$_2$-C_6H_4-4-F), 6.95-7.42 (m, 8H, H_{Ar} and 2H, NH_2 and 1H, NH, imidazolidin), 7.86 (s, 1H, H-3), 7.95 (s, 1H, H-6) ppm; ^{13}C NMR (125 MHz, DMSO-d_6) δ: 19.41, 41.27, 42.68, 44.67, 46.83, 48.45, 115.79, 117.76, 123.26, 128.19, 129.13, 130.53, 131.92, 132.97, 136.64, 137.00, 139.33, 150.17, 158.49, 161.02, 162.96, 164.70, 167.39, 174.35 ppm; Anal. calcd. for $C_{31}H_{32}Cl_2FN_9O_2S_2$ (716.68); C, 51.95; H, 4.50; N, 17.59. Found: C, 52.01; H, 4.55; N, 17.62; MALDI-TOF/TOF (715.1481) calcd for $C_{31}H_{32}Cl_2FN_9O_2S_2$ $[M+H]^+$ (716.1559) found 716.1799.

2-[{4-Amino-6-[4-(3-chlorophenyl)piperazin-1-yl]-1,3,5-triazin-2-yl}methylthio]-4-chloro-N-{1-[4-(fluorbenzyl)]imidazolidin-2-ylidene}-5-methylbenzenesulfonamide (**97**).

Starting from *N*-carbamimidoyl-4-(3-chlorophenyl)piperazine-1-carboximidamide hydrochloride (0.508 g, 1.60 mmol). The title compound was obtained after refluxing with ethanol (1:28) in an ultrasonic bath for 10 minutes. Yield 0.338 g (60%); m.p. 213–214 °C; IR (KBr): 3347, 3245 (N-H), 2977, 2914, 2855, 2820 (C-H), 1594, 1569, 1521 (C=N, $C=C_{Ar}$), 1265, 1161 (SO_2) cm^{-1}; ^1H NMR (500 MHz, DMSO-d_6) δ: 2.32 (s, 3H, CH_3Ph), 3.14-3.16 (m, 4H, piperazine), 3.27 (t, *J=5.8 Hz*, 2H, imidazolidin), 3.43 (t, *J=6.0 Hz*, 2H, imidazolidin), 3.78-3.84 (m, 4H, piperazine), 3.97 (s, 2H, S-CH_2), 4.35 (s, 2H, C**H**$_2$-C_6H_4-4-F), 6.81-7.42 (m, 8H, H_{Ar} and 2H, NH_2 and 1H, NH, imidazolidin), 7.86 (s, 1H, H-3), 7.95 (s, 1H, H-6) ppm; Anal.

calcd. for $C_{31}H_{32}Cl_2FN_9O_2S_2$ (716.68); C, 51.95; H, 4.50; N, 17.59. Found: C, 51.90; H, 4.49; N, 17.58; MALDI-TOF/TOF (715.1481) calcd for $C_{31}H_{32}Cl_2FN_9O_2S_2$ $[M+H]^+$ (716.1559) found 715.9756.

2-[{4-Amino-6-[4-(3,4-dichlorophenyl)piperazin-1-yl]-1,3,5-triazin-2-yl}methylthio]-4-chloro-N-[1-(4-fluorobenzyl)imidazolidin-2-ylidene]-5-methylbenzenesulfonamide (**98**).

Starting from *N*-carbamimidoyl-4-(3,4-dichlorophenyl)piperazine-1-carboximidamide hydrochloride (0.563 g, 1.60 mmol). The title compound was obtained after refluxing with ethanol (1:32) in an ultrasonic bath for 10 minutes. Yield 0.369 g (61%); m.p. 239–241 °C; IR (KBr): 3223, 3095 (N-H), 2946, 2917, 2880, 2831 (C-H), 1598, 1557, 1522, 1512 (C=N, $C=C_{Ar}$), 1268, 1129 (SO_2) cm^{-1}; ^1H NMR (500 MHz, DMSO-d_6) δ: 2.32 (s, 3H, CH_3Ph), 3.15-3.17 (m, 4H, piperazine), 3.27 (t, *J=8.75 Hz,* 2H, imidazolidin), 3.43 (t, *J=8.75 Hz,* 2H, imidazolidin), 3.77-3.83 (m, 4H, piperazine), 3.96 (s, 2H, S-CH_2), 4.35 (s, 2H, **CH**$_2$-C_6H_4-4-F), 6.94-7.43 (m, 7H, H_{Ar} and 2H, NH_2 and 1H, NH, imidazolidin), 7.86 (s, 1H, H-3), 7.95 (s, 1H, H-6) ppm; Anal. calcd. for $C_{31}H_{31}Cl_3FN_9O_2S_2$ (751.12); C, 49.57; H, 4.16; N, 16.78. Found: C, 49.38; H, 3.93; N, 16.59; MALDI-TOF/TOF (749.1092) calcd for $C_{31}H_{31}Cl_3FN_9O_2S_2$ $[M+H]^+$ (750.1170) found 749.9188.

2-[{4-Amino-6-[4-(3-chloro-4-fluorophenyl)piperazin-1-yl]-1,3,5-triazin-2-yl}methylthio]-4-chloro-N-[1-(4-fluorobenzyl)imidazolidin-2-ylidene]-5-methylbenzenesulfonamide (**99**).

Starting from *N*-carbamimidoyl-4-(3-chloro-4-fluorophenyl)piperazine-1-carboximidamide hydrochloride (0.536 g, 1.60 mmol). The title compound was obtained after refluxing with ethanol (1:28) in an ultrasonic bath for 10 minutes. Yield 0.403 g (62%); m.p. 215–216 °C; IR (KBr): 3392, 3339, 3225 (N-H), 2949, 2920, 2882, 2853 (C-H), 1597, 1558, 1512, 1476 (C=N, $C=C_{Ar}$), 1269, 1156 (SO_2) cm^{-1}; ^1H NMR (500 MHz, DMSO-d_6) δ: 2.32 (s, 3H, CH_3Ph), 3.04-3.14 (m, 4H, piperazine), 3.27 (t, *J=8.75 Hz*, 2H, imidazolidin), 3.43 (t, *J=8.75 Hz*, 2H, imidazolidin), 3.78-3.83 (m, 4H, piperazine), 3.96 (s, 2H, S-CH_2), 4.35 (s, 2H, **CH**$_2$-C_6H_4-4-F), 6.94-7.42 (m, 7H, H_{Ar} and 2H, NH_2 and 1H, NH, imidazolidin), 7.86 (s, 1H, H-3), 7.95 (s, 1H, H-6) ppm; Anal. calcd. for $C_{31}H_{31}Cl_2F_2N_9O_2S_2$ (734.67); C, 50.68; H, 4.25; N, 17.16. Found: C, 50.88; H, 4.06; N, 17.10; MALDI-TOF/TOF (733.1387) calcd for $C_{31}H_{31}Cl_2F_2N_9O_2S_2$ $[M+H]^+$ (734.1465) found 733.9447.

2-[{4-Amino-6-[4-(4-methoxyphenyl)piperazin-1-yl]-1,3,5-triazin-2-yl}methylthio]-4-chloro-N-[1-(4-fluorobenzyl)imidazolidin-2-ylidene]-5-methylbenzenesulfonamide (**100**).

Starting from *N*-carbamimidoyl-4-(4-methoxyphenyl)piperazine-1-carboximidamide hydrochloride (0.500 g, 1.60 mmol). The title compound was obtained after refluxing with ethanol (1:32) in an ultrasonic bath for 10 minutes. Yield 0.399 g (70%); m.p. 214–216 °C; IR (KBr): 3331, 3229 (N-H), 2960, 2936, 2900, 2839 (C-H), 1592, 1557, 1542, 1514 (C=N, $C=C_{Ar}$), 1246, 1142 (SO_2) cm^{-1}; ^1H NMR (500 MHz, DMSO-d_6) δ: 2.31 (s, 3H, CH_3Ph), 2.92-2.97 (m, 4H, piperazine), 3.27 (t, *J=8.8 Hz*, 2H, imidazolidin), 3.42 (t, *J=8.8 Hz*, 2H, imidazolidin), 3.69 (s, 3H, O-**CH**$_3$), 3.79-3.83 (m, 4H, piperazine), 3.96 (s, 2H, S-CH_2), 4.35 (s, 2H, **CH**$_2$-C_6H_4-4-F), 6.82-7.42 (m, 8H, H_{Ar} and 2H, NH_2 and 1H, NH, imidazolidin), 7.86 (s, 1H, H-3), 7.95 (s, 1H, H-6) ppm; Anal. calcd. for $C_{32}H_{35}ClFN_9O_3S_2$ (712.26); C, 53.96; H, 4.95; N, 17.70. Found: C, 53.93; H, 4.94; N, 17.70; MALDI-TOF/TOF (711.1977) calcd for $C_{32}H_{35}ClFN_9O_3S_2$ $[M+H]^+$ (712.2055) found 712.0672.

2-[{4-Amino-6-[4-(4-benzyl)piperazin-1-yl]-1,3,5-triazin-2-yl}methylthio]-4-chloro-N-[1-(4-fluorobenzyl)imidazolidin-2-ylidene]-5-methylbenzenesulfonamide (**101**).

Starting from 4-benzyl-*N*-carbamimidoylpiperazine-1-carboximidamide hydrochloride (0.475 g, 1.60 mmol). The title compound was obtained after refluxing with ethanol (1:33) in an ultrasonic bath for 10 minutes. Yield 0.319 g (52%); m.p. 172–173 °C; IR (KBr): 3330, 3234 (N-H), 2947, 2907, 2858, 2816 (C-H), 1588, 1557, 1519 (C=N, $C=C_{Ar}$), 1265, 1129 (SO_2)cm^{-1}; ^1H NMR (500 MHz, DMSO-d_6) δ: 2.29 (s, 3H, CH_3Ph and 4H, piperazine), 3.27 (t, *J=8.75 Hz*, 2H, imidazolidin), 3.37-3.46 (m, 2H, imidazolidin and 2H, Ph-CH_2), 3.62-3.70 (m, 4H, piperazine), 3.94 (s, 2H, S-CH_2), 4.33 (s, 2H, **CH**$_2$$C_6H_4$-4-F), 6.93-7.41 (m, 9H,

H_{Ar} 2H, NH_2 and 1H, NH, imidzolidin), 7.85 (s, 1H, H-3), 7.93 (s, 1H, H-6) ppm; Anal. calcd. for $C_{32}H_{35}ClFN_9O_2S_2$ (696.26); C, 55.20; H, 5.07; N, 18.11. Found: C, 55.17; H, 5.05; N, 18.11; MALDI-TOF/TOF (695.2028) calcd for $C_{32}H_{35}ClFN_9O_2S_2$ $[M+H]^+$ (696.2106) found 696.1479.

2-{[4-Amino-6-(indolin-1-yl)-1,3,5-triazin-2-yl]methylthio}-N-[1-(4-bromobenzyl)imidazolidin-2-ylidene]-4-chloro-5-methylbenzenesulfonamide (**102**).

Starting from *N*-carbamimidoylindoline-1-carboximidamide hydrochloride (0.384 g, 1.60 mmol). The title compound was obtained by crystallization from ethanol (1:20). Yield 0.287 g (51%); m.p. 209–210 °C; IR (KBr): 3345, 3239 (N-H), 2953, 2894, 2853 (C-H), 1584, 1557, 1519, 1488 (C=N, $C=C_{Ar}$), 1271, 1125 (SO_2) cm^{-1}; ^1H NMR (500 MHz, DMSO-d_6) δ: 2.31 (s, 3H, CH_3), 3.08 (t, *J=8.55 Hz*, 2H, hydroindoline), 3.17-3.28 (m, 2H, imidazolidin), 3.36-3.43 (m, 2H, imidazolidin), 4.04-4.20 (m, 2H, hydroindoline and 2H, S-CH_2), 4.23-4.34 (m, 2H, C**H**$_2$-C_6H_4-4-Br), 6.91-8.44 (m, 8H, H_{Ar} and 2H, NH_2 and 1H, NH, imidazolidin and 1H, H-3 and 1H, H-6) ppm; Anal. calcd. for $C_{29}H_{28}BrClN_8O_2S_2$ (700.07); C, 49.75; H, 4.03; N, 16.01. Found: C, 49.46; H, 3.99; N, 15.76; MALDI-TOF/TOF (698.0649) calcd for $C_{29}H_{28}BrClN_8O_2S_2$ $[M+H]^+$ (699.0727) found 699.0720.

2-{[4-Amino-6-(3,4-dihydroquinolin-1(2H)-yl)-1,3,5-triazin-2-yl]methylthio}-N-[1-(4-bromobenzyl)imidazolidin-2-ylidene]-4-chloro-5-methylbenzenesulfonamide (**103**).

Starting from *N*-carbamimidoyl-3,4-dihydroquinoline-1(2*H*)-carboximidamide hydrochloride (0.406 g, 1.60 mmol). The title compound was obtained after refluxing with ethanol (1:32) in an ultrasonic bath for 10 minutes. Yield 0.249 g (44%); m.p. 142–144 °C; IR (KBr): 3334, 3230 (N-H), 2947, 2916, 2890, 2837 (C-H), 1588, 1572, 1556, 1528 (C=N, $C=C_{Ar}$), 1278, 1171 (SO_2) cm^{-1}; ^1H NMR (500 MHz, DMSO-d_6) δ: 1.81 (quintet, *J=5.6 Hz*, 2H, 1,2,3,4-tetrahydroquinoline), 2.31 (s, 3H, CH_3), 2.70 (t, *J=6.6 Hz*, 2H, 1,2,3,4-tetrahydroquinoline), 3.26 (t, *J=8.55 Hz*, 2H, imidazolidin), 3.42 (t, *J=8.75 Hz*, 2H, imidazolidin), 3.89 (t, *J=6.1 Hz*, 2H, 1,2,3,4-tetrahydroquinoline), 4.00 (s, 2H, S-CH_2), 4.31 (s, 2H, C**H**$_2$-C_6H_4-4-Br), 6.95-7.69 (m, 8H, H_{Ar} and 2H, NH_2 and 1H, NH, imidazolidin), 7.79 (s, 1H, H-3), 7.86 (s, 1H, H-6) ppm; Anal. calcd. for $C_{30}H_{30}BrClN_8O_2S_2$ (714.10); C, 50.46; H, 4.23; N, 15.69. Found: C, 50.09; H, 4.16; N, 15.96; MALDI-TOF/TOF (712.0805) calcd for $C_{30}H_{30}BrClN_8O_2S_2$ $[M+H]^+$ (713.0883) found 713.1172.

2-{[4-Amino-6-(phenylamino)-1,3,5-triazin-2-yl]methylthio}-N-[1-(4-bromobenzyl)imidazolidin-2-ylidene]-4-chloro-5-methylbenzenesulfonamide (**104**).

Starting from 1-phenylbiguanide hydrochloride (0.342 g, 1.60 mmol). The title compound was obtained after refluxing with ethanol (1:21) in an ultrasonic bath for 10 minutes. Yield 0.225 g (42%); m.p. 212–213 °C; IR (KBr): 3321, 3212 (N-H), 2965, 2923, 2894, 2853 (C-H), 1596, 1574, 1533, 1488 (C=N, $C=C_{Ar}$), 1264, 1130 (SO_2) cm^{-1}; ^1H NMR (500 MHz, DMSO-d_6) δ: 2.31 (s, 3H, CH_3Ph), 3.26 (t, *J=8.55 Hz*, 2H, imidazolidin), 3.42 (t, *J=8.55 Hz*, 2H, imidazolidin), 4.03 (s, 2H, S-CH_2), 4.28-4.34 (m, 2H, C**H**$_2$-C_6H_4-4-Br), 6.94-7.74 (m, 9H, H_{Ar} and 1H, NH, imidazolidin and 1H, H-3 and 2H, NH_2), 7.87 (s, 1H, H-6), 9.51-9.60 (m, 1H, NH) ppm; Anal. calcd. for $C_{27}H_{26}BrClN_8O_2S_2$ (674.03); C, 48.11; H, 3.89; N, 16.62. Found: C, 48.09; H, 3.88; N, 16.61; MALDI-TOF/TOF (672.0492) calcd for $C_{27}H_{26}BrClN_8O_2S_2$ $[M+H]^+$ (673.0570) found 672.9877.

2-[{4-Amino-6-[(4-chlorophenyl)(methyl)amino]-1,3,5-triazin-2-yl]methylthio}-N-[1-(4-bromobenzyl)imidazolidin-2-ylidene]-4-chloro-5-methylbenzenesulfonamide (**105**).

Starting from 1-(4-chlorophenyl)-1-methylbiguanide hydrochloride (0.419 g, 1.60 mmol). The title compound was obtained after refluxing with ethanol (1:21) in an ultrasonic bath for 10 minutes. Yield 0.304 g (53%); m.p. 219–220 °C; IR (KBr): 3325, 3163 (N-H), 2977, 2949, 2918, 2889 (C-H), 1592, 1561, 1526, 1490 (C=N, $C=C_{Ar}$), 1276, 1168 (SO_2) cm^{-1}; ^1H NMR (500 MHz, DMSO-d_6) δ: 2.31 (s, 3H, CH_3Ph), 3.29 (t, *J=8.8 Hz*, 2H, imidazolidin), 3.37 (s, 3H, N-C**H**$_3$), 3.44 (t, *J=9.05 Hz*, 2H, imidazolidin), 3.94 (s, 2H, S-CH_2), 4.31-4.34 (m, 2H, C**H**$_2$-C_6H_4-4-Br), 7.02-7.46 (m, 8H, H_{Ar} and 2H, NH_2 and 1H, NH, imidazolidin), 7.77 (s, 1H, H-3), 7.85 (s, 1H, H-6) ppm; Anal. calcd. for $C_{28}H_{27}BrCl_2N_8O_2S_2$ (722.51); C, 46.55; H, 3.77; N, 15.51.

Found: C, 46.48; H, 3.72; N, 15.39; MALDI-TOF/TOF (720.0259) calcd for $C_{28}H_{27}BrCl_2N_8O_2S_2$ $[M+H]^+$ (721.0337) found 720.9861.

2-{[4-Amino-6-(4-phenylpiperazin-1-yl)-1,3,5-triazin-2-yl]methylthio}-N-[1-(4-bromobenzyl)imidazolidin-2-ylidene]-4-chloro-5-methylbenzenesulfonamide (**106**).

Starting from *N*-carbamimidoyl-4-phenylpiperazine-1-carboximidamide hydrochloride (0.452 g, 1.60 mmol). The title compound was obtained by crystallization from ethanol (1:20). Yield 0.300 g (51%); m.p. 192–193 °C; IR (KBr): 3292, 3206, 3137 (N-H), 2958, 2911, 2858, 2824 (C-H), 1592, 1553, 1519, 1493 (C=N, $C=C_{Ar}$), 1287, 1172 (SO_2) cm^{-1}; ^1H NMR (500 MHz, DMSO-d_6) δ: 2.31 (s, 3H, CH_3Ph), 3.00-3.16 (m, 4H, piperazine), 3.28 (t, *J=8.75 Hz*, 2H, imidazolidin), 3.42 (t, *J=8.8 Hz*, 2H, imidazolidin), 3.79-3.86 (m, 4H, piperazine), 3.97 (s, 2H, S-CH_2), 4.34 (s, 2H, C**H**$_2$-C_6H_4-4-Br), 6.80-7.48 (m, 9H, H_{Ar} and 2H, NH_2 and 1H, NH, imidazolidin), 7.85 (s, 1H, H-3), 7.93 (s, 1H, H-6) ppm; Anal. calcd. for $C_{31}H_{33}BrClN_9O_2S_2$ (743.14); C, 50.10; H, 4.48; N, 16.96. Found: C, 50.07; H, 4.47; N, 16.96; MALDI-TOF/TOF (741.1071) calcd for $C_{31}H_{33}BrClN_9O_2S_2$ $[M+H]^+$ (742.1149) found 742.0496.

2-[{4-Amino-6-[4-(4-fluorophenyl)piperazin-1-yl]-1,3,5-triazin-2-yl}methylthio}-N-[1-(4-bromobenzyl)imidazolidin-2-ylidene]-4-chloro-5-methylbenzenesulfonamide (**107**).

Starting from *N*-carbamimidoyl-4-(4-fluorophenyl)piperazine-1-carboximidamide hydrochloride (0.481 g, 1.60 mmol). The title compound was obtained after refluxing with ethanol (1:21) in an ultrasonic bath for 10 minutes. Yield 0.319 g (52%); m.p. 199–200 °C; IR (KBr): 3338, 3231, 3182 (N-H), 2945, 2886, 2864, 2825 (C-H), 1592, 1558, 1511, 1488 (C=N, $C=C_{Ar}$), 1270, 1125 (SO_2) cm^{-1}; ^1H NMR (500 MHz, DMSO-d_6) δ: 2.31 (s, 3H, CH_3), 3.00-3.05 (m, 4H, piperazine), 3.28 (t, *J=8.75 Hz*, 2H, imidazolidin), 3.43 (t, *J=8.8 Hz*, 2H, imidazolidin), 3.79-3.85 (m, 4H, piperazine), 3.96 (s, 2H, S-CH_2), 4.34 (s, 2H, C**H**$_2$-C_6H_4-4-Br), 6.95-7.48 (m, 8H, H_{Ar} and 2H, NH_2 and 1H, NH, imidazolidin), 7.85 (s, 1H, H-3), 7.93 (s, 1H, H-6) ppm; Anal. calcd. for $C_{31}H_{32}BrClFN_9O_2S_2$ (761.13); C, 48.92; H, 4.24; N, 16.56. Found: C, 48.93; H, 4.15; N, 16.57; MALDI-TOF/TOF (759.0976) calcd for $C_{31}H_{32}BrClFN_9O_2S_2$ $[M+H]^+$ (760.1054) found 760.0043.

2-{[4-Amino-6-{4-[3-(trifluoromethyl)phenyl]piperazin-1-yl}-1,3,5-triazin-2-yl]methylthio}-N-[1-(4-bromobenzyl)imidazolidin-2-ylidene]-4-chloro-5-methylbenzenesulfonamide (**108**).

Starting from *N*-carbamimidoyl-4-[3-(trifluoromethyl)phenyl]piperazine-1-carboximidamide hydrochloride (0.561 g, 1.60 mmol). The title compound was obtained after refluxing with ethanol (1:21) in an ultrasonic bath for 10 minutes. Yield 0.330 g (51%); m.p. 190–191 °C; IR (KBr): 3343, 3228 (N-H), 2951, 2914, 2890, 2857 (C-H), 1600, 1553, 1526, 1488 (C=N, $C=C_{Ar}$), 1290, 1162 (SO_2) cm^{-1}; ^1H NMR (500 MHz, DMSO-d_6) δ: 2.31 (s, 3H, CH_3Ph), 3.20-3.23 (m, 4H, piperazine), 3.28 (t, *J=8.8 Hz*, 2H, imidazolidin), 3.43 (t, *J=8.8 Hz*, 2H, imidazolidin), 3.83 (s, 4H, piperazine), 3.97 (s, 2H, S-CH_2), 4.34 (s, 2H, C**H**$_2$-C_6H_4-4-Br), 7.01-7.47 (m, 8H, H_{Ar} and 2H, NH_2 and 1H, NH, imidazolidin), 7.85 (s, 1H, H-3), 7.93 (s, 1H, H-6) ppm; Anal. calcd. for $C_{32}H_{32}BrClF_3N_9O_2S_2$ (811.14); C, 47.38; H, 3.98; N, 15.54. Found: C, 46.94; H, 3.87; N, 15.51; MALDI-TOF/TOF (809.0944) calcd for $C_{32}H_{32}BrClF_3N_9O_2S_2$ $[M+H]^+$ (810.1022) found 810.0150.

2-[{4-Amino-6-[4-(4-chlorophenyl)piperazin-1-yl]-1,3,5-triazin-2-yl}methylthio]-N-[1-(4-bromobenzyl)imidazolidin-2-ylidene]-4-chloro-5-methylbenzenesulfonamide (**109**).

Starting from *N*-carbamimidoyl-4-(4-chlorophenyl)piperazine-1-carboximidamide hydrochloride (0.508 g, 1.60 mmol). The title compound was obtained after refluxing with ethanol (1:21) in an ultrasonic bath for 10 minutes. Yield 0.365 g (59%); m.p. 199–200 °C; IR (KBr): 3370, 3321, 3228 (N-H), 2973, 2945, 2893, 2861 (C-H), 1589, 1553, 1519, 1501 (C=N, $C=C_{Ar}$), 1271, 1154 (SO_2) cm^{-1}; ^1H NMR (500 MHz, DMSO-d_6) δ: 2.31 (s, 3H, CH_3Ph), 3.06-3.11 (m, 4H, piperazine), 3.28 (t, *J=8.75 Hz*, 2H, imidazolidin), 3.43 (t, *J=8.8 Hz*, 2H, imidazolidin), 3.78-3.84 (m, 4H, piperazine), 3.96 (s, 2H, S-CH_2), 4.34 (s, 2H, C**H**$_2$-C_6H_4-4-Br), 6.95-7.47 (m, 8H, H_{Ar} and 2H, NH_2 and 1H, NH, imidazolidin), 7.85 (s, 1H, H-3), 7.92 (s, 1H, H-6) ppm; ^{13}C NMR (125 MHz, DMSO-d_6) δ: 19.41, 41.27, 42.68, 44.67, 46.83, 48.45, 115.79,

117.76, 123.26, 128.19, 129.13, 130.53, 131.92, 132.97, 136.64, 137.00, 139.33, 150.17, 158.49, 161.02, 162.96, 164.70, 167.39, 174.35 ppm; Anal. calcd. for $C_{31}H_{32}BrCl_2N_9O_2S_2$ (777.58); C, 47.88; H, 4.15; N, 16.21. Found: C, 47.85; H, 4.08; N, 16.24; MALDI-TOF/TOF (775.0681) calcd for $C_{31}H_{32}BrCl_2N_9O_2S_2$ $[M+H]^+$ (776.0759) found 775.9246.

2-[{4-Amino-6-[4-(3,4-dichlorophenyl)piperazin-1-yl]-1,3,5-triazin-2-yl}methylthio]-N-[1-(4-bromobenzyl)imidazolidin-2-ylidene]-4-chloro-5-methylbenzenesulfonamide (**110**).

Starting from *N*-carbamimidoyl-4-(3,4-dichlorophenyl)piperazine-1-carboximidamide hydrochloride (0.563 g, 1.60 mmol). The title compound was obtained after refluxing with ethanol (1:20) in an ultrasonic bath for 10 minutes. Yield 0.393 g (61%); m.p. 230–231 °C; IR (KBr): 3381, 3331, 3223 (N-H), 2944, 2916, 2877, 2835 (C-H), 1599, 1557, 1519, 1483 (C=N, C=C_{Ar}), 1282, 1165 (SO_2) cm^{-1}; ^1H NMR (500 MHz, DMSO-d_6) δ: 2.31 (s, 3H, CH_3), 3.16-3.18 (m, 4H, piperazine), 3.28 (t, *J=8.8 Hz,* 2H, imidazolidin), 3.43 (t, *J=8.75 Hz,* 2H, imidazolidin), 3.78-3.84 (m, 4H, piperazine), 3.96 (s, 2H, S-CH_2), 4.34 (s, 2H, **C**H_2-C_6H_4-4-Br), 6.93-7.47 (m, 7H, H_{Ar} and 2H, NH_2 and 1H, NH, imidazolidin), 7.85 (s, 1H, H-3), 7.93 (s, 1H, H-6) ppm; Anal. calcd. for $C_{31}H_{31}BrCl_3N_9O_2S_2$ (812.03); C, 45.85; H, 3.85; N, 15.52. Found: C, 45.80; H, 3.76; N, 15.15; MALDI-TOF/TOF (809.0291) calcd for $C_{31}H_{31}BrCl_3N_9O_2S_2$ $[M+H]^+$ (810.0369) found 809.8684.

2-{[4-Amino-6-(4-benzylpiperazin-1-yl)-1,3,5-triazin-2-yl]methylthio}-4-chloro-N-[1-(4-fluorobenzyl)imidazolidin-2-ylidene]-5-methylbenzenesulfonamide (**111**).

Starting from 4-benzyl-*N*-carbamimidoylpiperazine-1-carboximidamide hydrochloride (0.475 g, 1.60 mmol). The title compound was obtained after refluxing with ethanol (1:22) in an ultrasonic bath for 10 minutes. Yield 0.329 g (55%); m.p. 175–176 °C; IR (KBr): 3378, 3224, 3176 (N-H), 2966, 2920, 2896, 2861 (C-H), 1580, 1569, 1524, 1487 (C=N, C=C_{Ar}), 1284, 1162 (SO_2) cm^{-1}; ^1H NMR (500 MHz, DMSO-d_6) δ: 2.29 (s, 3H, CH_3Ph), 2.31-2.35 (m, 4H, piperazine), 3.28 (t, *J=8.8 Hz,* 2H, imidazolidin), 3.41-3.46 (m, 2H, imidazolidin and 2H, Ph-CH_2), 3.62-3.70 (m, 4H, piperazine), 3.93 (s, 2H, S-CH_2), 4.32 (s, 2H, **C**H_2-C_6H_4-4-Br), 6.93-7.46 (m, 9H, H_{Ar} and 2H, NH_2 and 1H, NH, imidzolidin), 7.83 (s, 1H, H-3), 7.91 (s, 1H, H-6) ppm; Anal. calcd. for $C_{32}H_{35}BrClN_9O_2S_2$ (757.17); C, 50.76; H, 4.66; N, 16.65. Found: C, 50.59; H, 4.53; N, 16.48; MALDI-TOF/TOF (755.1227) calcd for $C_{32}H_{35}BrClN_9O_2S_2$ $[M+H]^+$ (756.1305) found 755.9094.

2-{[4-Amino-6-(3,4-dihydroquinolin-1(2H)-yl)-1,3,5-triazin-2-yl]methylthio}-4-chloro-5-methyl-N-{1-[3-(trifluoromethyl)benzyl]imidazolidin-2-ylidene}benzenesulfonamide (**112**).

Starting from *N*-carbamimidoyl-3,4-dihydroquinoline-1(*2H*)-carboximidamide hydrochloride (0.406 g, 1.60 mmol). The title compound was obtained by evaporate methanol. 50 ml water was added to the resulting oil and mixed for 20 min. The oil was decanted and crystallized from 1.5 mL MeCN to give (0.185 g, 40%). All was crystallized from (1:22) MeCN. Yield 0.100 g (22%); m.p. 112–113 °C; IR (KBr): 3315, 3185 (N-H), 2969, 2925, 2851 (C-H), 1585, 1561, 1522 (C=N, C=C_{Ar}), 1271, 1162 (SO_2) cm^{-1}; ^1H NMR (500 MHz, DMSO-d_6) δ: 1.80 (quintet, *J=8.4 Hz,* 2H, 1,2,3,4-tetrahydroquinoline), 2.31 (s, 3H, CH_3Ph), 2.70 (t, *J=6.55 Hz,* 2H, tetrahydroquinoline), 3.29 (t, *J=8.55 Hz,* 2H, imidazolidin), 3.44 (t, *J=8.55 Hz,* 2H, imidazolidin), 3.88 (t, *J=6.10 Hz,* 2H, tetrahydroquinoline), 3.97 (s, 2H, S-CH_2), 4.44 (s, 2H, **C**H_2-C_6H_4-3-CF_3), 6.94-7.68 (m, 8H, H_{Ar} and 2H, NH_2 and 1H, NH, imidazolidin), 7.78 (s, 1H, H-3), 7.87 (s, 1H, H-6) ppm; ^{13}C NMR (125 MHz, DMSO-d_6) δ: 19.42, 23.74, 26.98, 41.34, 44.23, 44.92, 46.30, 47.14, 123.77, 124.64, 124.81, 125.44, 125.92, 127.50, 128.43, 128.73, 130.03, 130.78, 131.25, 132.33, 136.35, 137.06, 138.46, 139.03, 139.58, 158.66, 161.73, 164.87, 167.26, 170.15, 174.36 ppm; Anal. calcd. for $C_{31}H_{30}ClF_3N_8O_2S_2$ (703.20); C, 52.95; H, 4.30; N, 15.93. Found: C, 52.19; H, 4.41; N, 15.06; MALDI-TOF/TOF (702.1574) calcd for $C_{31}H_{30}ClF_3N_8O_2S_2$ $[M+H]^+$ (703.1652) found 703.0936.

2-{[4-Amino-6-(4-phenylpiperazin-1-yl)-1,3,5-triazin-2-yl]methylthio}-4-chloro-5-methyl-N-{1-[3-(trifluoromethyl)benzyl]imidazolidin-2-ylidene}-4-benzenesulfonamide (**113**).

Starting from *N*-carbamimidoyl-4-phenylpiperazine-1-carboximidamide hydrochloride (0.452 g, 1.60 mmol). The title compound was obtained by crystallization from ethanol (1:23). Yield 0.330 g (56%); m.p. 148–149 °C; IR (KBr): 3315, 3208, 3163 (N-H), 2968, 2933, 2902, 2850 (C-H), 1585, 1562, 1541, 1514 (C=N, $C=C_{Ar}$), 1277, 1163 (SO_2) cm^{-1}; ^1H NMR (500 MHz, DMSO-d_6) δ: 2.31 (s, 3H, CH_3Ph), 3.07-3.10 (m, 4H, piperazine), 3.30-3.33 (m, 2H, imidazolidin), 3.43-3.47 (m, 2H, imidazolidin), 3.79-3.84 (m, 4H, piperazine), 3.93 (s, 2H, S-CH_2), 4.48 (s, 2H, $\mathbf{CH_2}$-C_6H_4-3-CF_3), 6.80-7.64 (m, 9H, H_{Ar} and 2H, NH_2 and 1H, NH, imidazolidin), 7.86 (s, 1H, H-3), 7.93 (s, 1H, H-6) ppm; Anal. calcd. for $C_{32}H_{33}ClF_3N_9O_2S_2$ (732.24); C, 52.49; H, 4.54; N, 17.22. Found: C, 52.28; H, 4.48; N, 17.01; MALDI-TOF/TOF (731.1839) calcd for $C_{32}H_{33}ClF_3N_9O_2S_2$ $[M+H]^+$ (732.1917) found 732.2485.

2-{[4-Amino-6-{4-[4-(trifluoromethyl)phenyl]piperazin-1-yl}-1,3,5-triazin-2-yl]methylthio}-4-chloro-5-methyl-N-{1-[3-(trifluoromethyl)benzyl]imidazolidin-2-ylidene}benzenesulfonamide (**114**).

Starting from *N*-carbamimidoyl-4-[4-(trifluoromethyl)phenyl]piperazine-1-carboximidamide hydrochloride (0.561 g, 1.60 mmol). The title compound was obtained after refluxing with ethanol (1:20) in an ultrasonic bath for 10 minutes. Yield 0.282 g (47%); m.p. 218–220 °C; IR (KBr): 3396, 3219 (N-H), 2949, 2921, 2886, 2861 (C-H), 1576, 1554, 1523 (C=N, $C=C_{Ar}$), 1272, 1165 (SO_2) cm^{-1}; ^1H NMR (500 MHz, DMSO-d_6) δ: 2.31 (s, 3H, CH_3Ph), 3.07-3.33 (m, 4H, piperazine and 2H, imidazolidin), 3.44-3.47 (m, 2H, imidazolidin), 3.79-3.85 (m, 4H, piperazine), 3.93 (s, 2H, S-CH_2), 4.48 (s, 2H, $\mathbf{CH_2}$-C_6H_4-3-CF_3), 6.99-7.63 (m, 8H, H_{Ar} and 2H, NH_2 and 1H, NH, imidazolidin), 7.86 (s, 1H, H-3), 7.93 (s, 1H, H-6) ppm; Anal. calcd. for $C_{33}H_{32}ClF_6N_9O_2S_2$ (800.24); C, 49.53; H, 4.03; N, 15.75. Found: C, 49.45; H, 3.94; N, 15.69; MALDI-TOF/TOF (799.1713) calcd for $C_{33}H_{32}ClF_6N_9O_2S_2$ $[M+H]^+$ (800.1791) found 800.1279.

[{4-Amino-6-[4-(3,4-dichlorophenyl)piperazin-1-yl]-1,3,5-triazin-2-yl}methylthio]-4-chloro-5-methyl-N-{1-[3-(trifluoromethyl)benzyl]imidazolidin-2-ylidene}benzenesulfonamide (**115**).

Starting from *N*-carbamimidoyl-4-(3,4-dichlorophenyl)piperazine-1-carboximidamide hydrochloride (0.563 g, 1.60 mmol). The title compound was obtained after refluxing with ethanol (1:20) in an ultrasonic bath for 10 minutes. Yield 0.343 g (52%); m.p. 215–216 °C; IR (KBr): 3394, 3333, 3219 (N-H), 2921, 2834, 2879 (C-H), 1595, 1557, 1523, 1485 (C=N, $C=C_{Ar}$), 1271, 1162 (SO_2) cm^{-1}; ^1H NMR (500 MHz, DMSO-d_6) δ: 2.31 (s, 3H, CH_3Ph), 3.10-3.22 (m, 4H, piperazine), 3.30-3.33 (m, 2H, imidazolidin), 3.40-3.47 (m, 2H, imidazolidin), 3.76-3.83 (m, 4H, piperazine), 3.92 (s, 2H, S-CH_2), 4.48 (s, 2H, $\mathbf{CH_2}$-C_6H_4-3-CF_3), 6.93-7.62 (m, 7H, H_{Ar} and 2H, NH_2 and 1H, NH, imidazolidin), 7.85 (s, 1H, H-3), 7.93 (s, 1H, H-6) ppm; Anal. calcd. for $C_{32}H_{31}Cl_3F_3N_9O_2S_2$ (801.13); C, 47.97; H, 3.90; N, 15.74. Found: C, 47.93; H, 3.79; N, 15.65; MALDI-TOF/TOF (799.1060) calcd for $C_{32}H_{31}Cl_3F_3N_9O_2S_2$ $[M+H]^+$ (800.1138) found 799.9224.

2-[{4-Amino-6-[4-(4-methoxyphenyl)piperazin-1-yl]-1,3,5-triazin-2-yl}methylthio]-4-chloro-5-methyl-N-{1-[3-(trifluoromethyl)benzyl]imidazolidin-2-ylidene}-benzenesulfonamide (**116**).

Starting from *N*-carbamimidoyl-4-(4-methoxyphenyl)piperazine-1-carboximidamide hydrochloride (0.500 g, 1.60 mmol). The title compound was obtained after refluxing with ethanol (1:20) in an ultrasonic bath for 10 minutes. Yield 0.321 g (53%); m.p. 191–192 °C; IR (KBr): 3322, 3222, 3159 (N-H), 2977, 2923, 2904, 2857 (C-H), 1585, 1559, 1546, 1511 (C=N, $C=C_{Ar}$), 1274, 1163 (SO_2) cm^{-1}; ^1H NMR (500 MHz, DMSO-d_6) δ: 2.31 (s, 3H, CH_3Ph), 2.92-2.96 (m, 4H, piperazine), 3.30-3.33 (m, 2H, imidazolidin), 3.43-3.47 (m, 2H, imidazolidin), 3.69 (s, 3H, O-$\mathbf{CH_3}$), 3.78-3.83 (m, 4H, piperazine), 3.93 (s, 2H, S-CH_2), 4.48 (s, 2H, $\mathbf{CH_2}$-C_6H_4-3-CF_3), 6.82-7.63 (m, 8H, H_{Ar} and 2H, NH_2 and 1H, NH, imidazolidin), 7.86 (s, 1H, H-3), 7.93 (s, 1H, H-6) ppm; Anal. calcd. for $C_{33}H_{35}ClF_3N_9O_3S_2$ (762.27); C, 52.00; H, 4.63; N, 16.54. Found: C, 51.76; H, 4.44; N, 16.28; MALDI-TOF/TOF (761.1945) calcd for $C_{33}H_{35}ClF_3N_9O_3S_2$ $[M+H]^+$ (762.2023) found 762.0063.

2-{[4-Amino-6-(4-benzylpiperazin-1-yl)-1,3,5-triazin-2-yl]methylthio}-4-chloro-5-methyl-N-{1-[3-(trifluoromethyl)benzyl]imidazolidin-2-ylidene}benzenesulfonamide (**117**).

Starting from 4-benzyl-*N*-carbamimidoylpiperazine-1-carboximidamide hydrochloride (0.475 g, 1.60 mmol). The title compound was obtained after refluxing with ethanol (1:22) in an ultrasonic bath for 10 minutes. Yield 0.251 g (42%); m.p. 169–170 °C; IR (KBr): 3316, 3208, 3155 (N-H), 2979, 2935, 2916, 2859 (C-H), 1587, 1561, 1530, 1512 (C=N, C=C_{Ar}), 1275, 1163 (SO_2) cm^{-1}; ^{1}H NMR (500 MHz, DMSO-d_6) δ: 2.23-2.35 (m, 3H, CH_3Ph and 4H, piperazine), 3.29-3.33 (m, 2H, imidazolidin), 3.43-3.47 (m, 2H, imidazolidin and 2H, CH_2Ph), 3.61-3.69 (m, 4H, piperazine), 3.90 (s, 2H, S-CH_2), 4.45 (s, 2H, C**H**$_2$-C_6H_4-3-CF_3), 6.92-7.62 (m, 9H, H_{Ar} and 2H, NH_2 and 1H, NH, imidazolidin), 7.84 (s, 1H, H-3), 7.91 (s, 1H, H-6) ppm; Anal. calcd. for $C_{33}H_{35}ClF_3N_9O_2S_2$ (746.27); C, 53.11; H, 4.73; N, 16.89. Found: C, 53.01; H, 4.58; N, 16.88; MALDI-TOF/TOF (745.1996) calcd for $C_{33}H_{35}ClF_3N_9O_2S_2$ [M+H]$^+$ (746.2074) found 746.0577.

2-{[4-Amino-6-(dimethylamino)-1,3,5-triazin-2-yl]methylthio}-4-chloro-5-methyl-N-{1-[4-(trifluoromethyl)benzyl]imidazolidin-2-ylidene}benzenesulfonamide (**118**).

Starting from 1,1-dimethylbiguanide hydrochloride (0.265 g, 1.60 mmol). The title compound was obtained after refluxing with ethanol (1:26) in an ultrasonic bath for 10 minutes. Yield 0.280 g (60%); m.p. 208–209 °C; IR (KBr): 3325, 3234, 3136 (N-H), 2977, 2936, 2922, 2863 (C-H), 1569, 1510, 1484 (C=N, C=C_{Ar}), 1284, 1131 (SO_2) cm^{-1}; ^{1}H NMR (500 MHz, DMSO-d_6) δ: 2.30 (s, 3H, CH_3Ph), 3.00 (s, 3H, N-C**H**$_3$), 3.03 (s, 3H, N-C**H**$_3$), 3.31-3.35 (m, 2H, imidazolidin), 3.46 (t, *J=8.75 Hz*, 2H, imidazolidin), 3.92 (s, 2H, S-CH_2), 4.46 (s, 2H, C**H**$_2$-C_6H_4-4-CF_3), 6.85-6.90 (m, 2H, NH_2), 7.41-7.63 (m, 4H, H_{Ar} and 1H, NH, imidazolidin), 7.83 (s, 1H, H-3), 7.94 (s, 1H, H-6) ppm; Anal. calcd. for $C_{24}H_{26}ClF_3N_8O_2S_2$ (615.09); C, 46.86; H, 4.26; N, 18.22. Found: C, 46.85; H, 4.24; N, 18.22; MALDI-TOF/TOF (614.1261) calcd for $C_{24}H_{26}ClF_3N_8O_2S_2$ [M+H]$^+$ (615.1339) found 615.0067.

2-{[(4-Amino-6-morpholino-1,3,5-triazin-2-yl)methylthio]-4-chloro-5-methyl-N-{1-[4-(trifluoromethyl)benzyl]imidazolidin-2-ylidene}benzenesulfonamide (**119**).

Starting from *N*-carbamimidoylmorpholine-4-carboximidamide hydrochloride (0.332 g, 1.60 mmol). The title compound was obtained after refluxing with ethanol (1:25) in an ultrasonic bath for 10 minutes.Yield 0.293 g (56%); m.p. 198–199 °C; IR (KBr): 3396, 3350, 3233 (N-H), 2954, 2916, 2894, 2849 (C-H), 1580, 1519, 1465 (C=N, C=C_{Ar}), 1276, 1163 (SO_2) cm^{-1}; ^{1}H NMR (500 MHz, DMSO-d_6) δ: 2.30 (s, 3H, CH_3Ph), 3.31-3.33 (m, 2H, imidazolidin), 3.46 (t, *J=8.8 Hz*, 2H, imidazolidin), 3.51-3.58 (m, 4H, morpholine), 3.61-3.67 (m, 4H, morpholine), 3.93 (s, 2H, S-CH_2), 4.46 (s, 2H, C**H**$_2$-C_6H_4-4-CF_3), 6.95-7.65 (m, 4H, H_{Ar} and 2H, NH_2 and 1H, NH, imidazolidin), 7.84 (s, 1H, H-3), 7.91 (s, 1H, H-6) ppm; Anal. calcd. for $C_{26}H_{28}ClF_3N_8O_3S_2$ (657.13); C, 47.52; H, 4.29; N, 17.05. Found: C, 47.12; H, 4.23; N, 16.77; MALDI-TOF/TOF (656.1366) calcd for $C_{26}H_{28}ClF_3N_8O_3S_2$ [M+H]$^+$ (657.1444) found 656.9855.

2-{[4-Amino-6-(indolin-1-yl)-1,3,5-triazin-2-yl]methylthio}-4-chloro-5-methyl-N-{1-[4-(trifluoromethyl)benzyl]imidazolidin-2-ylidene}benzenesulfonamide (**120**).

Starting from *N*-carbamimidoylindoline-1-carboximidamide hydrochloride (0.384 g, 1.60 mmol). The title compound was obtained by crystallization from ethanol (1:95). Yield 0.230 g (41.74%); m.p. 175–176 °C; IR (KBr): 3341, 3175, 3230 (N-H), 2951, 2922, 2908, 2856 (C-H), 1584, 1558, 1519 (C=N, C=C_{Ar}), 1282, 1166 (SO_2) cm^{-1}; ^{1}H NMR (500 MHz, DMSO-d_6) δ: 2.30 (s, 3H, CH_3), 3.03-3.11 (m, 2H, hydroindoline), 3.21-2.26 (m, 2H, imidazolidin), 3.38-3.46 (m, 2H, imidazolidin), 4.05-4.06 (m, 2H, hydroindoline and 2H, S-CH_2), 4.36-4.45 (m, 2H, C**H**$_2$-C_6H_4-4-CF_3), 6.91-8.44 (m, 8H, H_{Ar} and 1H, NH, imidazolidin and 2H, NH_2 and 1H, H-3 and 1H, H-6) ppm; Anal. calcd. for $C_{30}H_{28}ClF_3N_8O_2S_2$ (689.17); C, 52.28; H, 4.10; N, 16.26. Found: C, 51.84; H, 4.15; N, 16.14. HRMS (ESI-TOF) (688.1417) calcd for $C_{30}H_{28}ClF_3N_8O_2S_2$ [M+H]$^+$ (689.1495) found 689.1504.

2-{[4-Amino-6-(3,4-dihydroquinolin-1(2H)-yl)-1,3,5-triazin-2-yl]methylthio}-4-chloro-5-methyl-N-{1-[4-(trifluoromethyl)benzyl]imidazolidin-2-ylidene}benzenesulfonamide (**121**).

Starting from *N*-carbamimidoyl-3,4-dihydroquinoline-1(2*H*)-carboximidamide hydrochloride (0.406 g, 1.60 mmol). The title compound was obtained after refluxing with ethanol (1:29) in an

ultrasonic bath for 10 minutes. Yield 0.178 g (32%); m.p. 192–193 °C; IR (KBr): 3331, 3229 (N-H), 2954, 2922, 2896, 2845 (C-H), 1592, 1581, 1557, 1528 (C=N, $C=C_{Ar}$), 1270, 1168 (SO_2) cm^{-1}; ^1H NMR (500 MHz, DMSO-*d*$_6$) δ: 1.80 (quintet, *J=6.2 Hz*, 2H, 1,2,3,4-tetrahydroquinolin), 2.31 (s, 3H, CH_3Ph), 2.70 (t, *J=6.6 Hz*, 2H, 1,2,3,4-tetrahydroquinoline), 3.29-3.31 (m, 2H, imidazolidin), 3.44 (t, *J=8.55 Hz*, 2H, imidazolidin), 3.88 (t, *J=5.85 Hz*, 2H, 1,2,3,4-tetrahydroquinolin), 3.99 (s, 2H, S-CH_2), 4.43 (s, 2H, C**H**$_2$-C_6H_4-4-CF_3), 6.95-7.67 (m, 8H, H_{Ar} and 2H, NH_2 and 1H, NH, imidazolidin), 7.78 (s, 1H, H-3), 7.86 (s, 1H, H-6) ppm; Anal. calcd. for $C_{31}H_{30}ClF_3N_8O_2S_2$ (703.20); C, 52.95; H, 4.30; N, 15.93. Found: C, 52.65; H, 4.20; N, 15.63. HRMS (ESI-TOF) (702.1574) calcd for $C_{31}H_{30}ClF_3N_8O_2S_2$ $[M+H]^+$ (703.1652) found 703.1650.

2-{[4-Amino-6-(phenylamino)-1,3,5-triazin-2-yl]methylthio}-4-chloro-5-methyl-N-{1-[4-(trifluoromethyl)benzyl]imidazolidin-2-ylidene}benzenesulfonamide (**122**).

Starting from 1-phenylbiguanide hydrochloride (0.342 g, 1.60 mmol). The title compound was obtained after refluxing with ethanol (1:32) in an ultrasonic bath for 10 minutes. Yield 0.259 g (46%); m.p. 223–224 °C (dec.); IR (KBr): 3319, 3220 (N-H), 2993, 2956, 2922, 2853 (C-H), 1597, 1575, 1553, 1530 (C=N, $C=C_{Ar}$), 1273, 1167 (SO_2) cm^{-1}; ^1H NMR (500 MHz, DMSO-*d*$_6$) δ: 2.30 (s, 3H, CH_3Ph), 3.28-3.34 (m, 2H, imidazolidin), 3.42-3.46 (m, 2H, imidazolidin), 4.03 (s, 2H, S-CH_2), 4.42-4.46 (m, 2H, C**H**$_2$-C_6H_4-4-CF_3), 6.94-7.73 (m, 9H, H_{Ar} and 1H, NH and 2H, NH_2 and imidazolidin and 1H, H-3), 7.86 (s, 1H, H-6), 9.49-9.61 (m, 1H, NH) ppm; Anal. calcd. for $C_{28}H_{26}ClF_3N_8O_2S_2$ (663.14); C, 50.71; H, 3.95; N, 16.90. Found: C, 50.71; H, 3.85; N, 16.86. HRMS (ESI-TOF) (662.1261) calcd for $C_{28}H_{26}ClF_3N_8O_2S_2$ $[M+H]^+$ (663.1339) found 663.1334.

2-[{4-Amino-6-[(4-fluorophenyl)amino]-1,3,5-triazin-2-yl}methylthio]-4-chloro-5-methyl-N-{1-[4-(trifluoromethyl)benzyl]imidazolidin-2-ylidene}benzenesulfonamide (**123**).

Starting from 1-(4-fluorophenyl)biguanide hydrochloride (0.371 g, 1.60 mmol). The title compound was obtained. Yield 0.264 g (48%); m.p. 218–220 °C; IR (KBr): 3323, 3208 (N-H), 2962, 2934, 2898, 2861 (C-H), 1595, 1538, 1506 (C=N, $C=C_{Ar}$), 1282, 1165 (SO_2) cm^{-1}; ^1H NMR (500 MHz, DMSO-*d*$_6$ in 60 °C) δ: 2.31 (s, 3H, CH_3), 3.32-3.35 (m, 2H, imimidazolidin), 3.46-3.49 (m, 2H, imimidazolidin), 4.01 (s, 2H, S-CH_2), 4.46 (s, 2H, C**H**$_2$-C_6H_4-4-CF_3), 6.97-7.72 (m, 8H, H_{Ar} and 2H, NH_2 and 1H, NH, imidazolidin and 1H, H-3), 7.86 (s, 1H, H-6), 9.38-9.45 (m, 1H, N-H) ppm; ^{13}C NMR (125 MHz, DMSO-d$_6$) δ: 19.40, 40.26, 41.34, 44.98, 47.25, 115.19, 115.37, 122.04, 125.74, 128.28, 128.63, 128.86, 130.87, 132.19, 136.41, 137.12, 139.64, 141.74, 157.00, 158.72, 158.91, 164.57, 167.20, 174.47 ppm; Anal. calcd. for $C_{28}H_{25}ClF_4N_8O_2S_2$ (681.13); C, 49.37; H, 3.70; N, 16.45. Found: C, 48.99; H, 3.30; N, 16.07; MALDI-TOF/TOF (680.1167) calcd for $C_{28}H_{25}ClF_4N_8O_2S_2$ $[M+H]^+$ (681.1245) found 680.9702.

2-[{4-Amino-6-[(3-chlorophenyl)amino]-1,3,5-triazin-2-yl}methylthio]-4-chloro-5-methyl-N-{1-[4-(trifluoromethyl)benzyl]imidazolidin-2-ylidene}benzenesulfonamide (**124**).

Starting from 1-(3-chlorophenyl)biguanide hydrochloride (0.397 g, 1.60 mmol). The title compound was obtained after refluxing with ethanol (1:35) in an ultrasonic bath for 10 minutes. Yield 0.249 g (45%); m.p. 204–205 °C; IR (KBr): 3320, 3219 (N-H), 2990, 2951, 2922, 2855 (C-H), 1596, 1571, 1549, 1525 (C=N, $C=C_{Ar}$), 1287, 1129 (SO_2) cm^{-1}; ^1H NMR (500 MHz, DMSO-*d*$_6$) δ: 2.30 (s, 3H, CH_3Ph), 3.29-3.33 (m, 2H, imidazolidin), 3.43-3.47 (m, 2H, imidazolidin), 4.04 (s, 2H, S-CH_2), 4.45 (s, 2H, C**H**$_2$-C_6H_4-4-CF_3), 6.77-7.86 (m, 8H, H_{Ar} and 2H, NH_2 and 1H, NH, imidazolidin and 1H, H-3 and 1H, H-6), 9.72-9.80 (m, 1H, NH) ppm; Anal. calcd. for $C_{28}H_{25}Cl_2F_3N_8O_2S_2$ (697.58); C, 48.21; H, 3.61; N, 16.06. Found: C, 47.96; H, 3.56; N, 15.96; MALDI-TOF/TOF (696.0871) calcd for $C_{28}H_{25}Cl_2F_3N_8O_2S_2$ $[M+H]^+$ (697.0949) found. 696.9099.

2-{[4-Amino-6-{[4-(trifluoromethyl)phenyl]amino}-1,3,5-triazin-2-yl]methylthio}-4-chloro-5-methyl-N-{1-[4-(trifluoromethyl)benzyl]imidazolidin-2-ylidene}benzenesulfonamide (**125**).

Starting from 1-[4-(trifluoromethyl)phenyl]biguanide hydrochloride (0.451 g, 1.60 mmol). The title compound was obtained by crystallization with ethanol (1:11). Yield 0.197 g (34%); m.p. 136–137 °C; IR

(KBr): 3331, 3223 (N-H), 2979, 2945, 2920, 2902 (C-H), 1585, 1581, 1526, 1489 (C=N, $C=C_{Ar}$), 1273, 1168 (SO_2) cm^{-1}; ^1H NMR (500 MHz, DMSO-d_6) δ: 2.30 (s, 3H, CH_3), 3.28 (t, *J=8.55 Hz*, 2H, imidazolidin), 3.42 (t, *J=8.55 Hz*, 2H, imidazolidin), 4.08 (s, 2H, S-CH_2), 4.43 (s, 2H, C**H**$_2$-C_6H_4-4-CF_3), 7.24-7.94 (m, 8H, H_{Ar} and 2H, NH_2 and 1H, NH, imidazolidin and 1H, H-3 and 1H, H-6), 9.90-10.01 (m, 1H, N**H**-C_6H_4-4-CF_3) ppm; Anal. calcd. for $C_{29}H_{25}ClF_6N_8O_2S_2$ (731.13); C, 47.64; H, 3.45; N, 15.33. Found: C, 47.54; H, 3.40; N, 15.11; MALDI-TOF/TOF (730.1135) calcd for $C_{29}H_{25}ClF_6N_8O_2S_2$ $[M+H]^+$ (731.1213) found 731.0334.

2-[{4-Amino-6-[(4-methoxyphenyl)amino]-1,3,5-triazin-2-yl}methylthio]-4-chloro-5-methyl-N-{1-[4-(trifluoromethyl)benzyl]imidazolidin-2-ylidene}benzenesulfonamide (**126**).

Starting from 1-(4-methoxyphenyl)biguanide hydrochloride (0.390 g, 1.60 mmol). The title compound was obtained after refluxing with ethanol (1:30) in an ultrasonic bath for 10 minutes. Yield 0.295 g (53%); m.p. 216–217 °C; IR (KBr): 3315, 3254, 3216 (N-H), 2956, 2933, 2836 (C-H), 1596, 1561, 1534, 1509 (C=N, $C=C_{Ar}$), 1285, 1166 (SO_2) cm^{-1}; ^1H NMR (500 MHz, DMSO-d_6) δ: 2.31 (s, 3H, CH_3Ph), 3.30-3.34 (m, 2H, imimidazolidin), 3.40-3.48 (m, 2H, imimidazolidin), 3.70 (br.s, 3H, O-CH_3), 4.00 (s, 2H, S-CH_2), 4.44 (s, 2H, C**H**$_2$-C_6H_4-4-CF_3), 6.80-7.65 (m, 8H, H_{Ar} and 2H, NH_2 and 1H, NH, imidazolidin and 1H, H-3), 7.86 (s, 1H, H-6), 9.20-9.55 (m, 1H, N**H**-C_6H_4-4-OMe) ppm; Anal. calcd. for $C_{29}H_{28}ClF_3N_8O_3S_2$ (693.16); C, 50.25; H, 4.07; N, 16.17. Found: C, 50.26; H, 4.07; N, 16.17; MALDI-TOF/TOF (692.1366) calcd for $C_{29}H_{28}ClF_3N_8O_3S_2$ $[M+H]^+$ (693.1444) found 693.1486.

2-{[4-Amino-6-(benzylamino)-1,3,5-triazin-2-yl]methylthio}-4-chloro-5-methyl-N-{1-[4-(trifluoromethyl)benzyl]imidazolidin-2-ylidene}benzenesulfonamide (**127**).

Starting from 2-benzyl-1-(diaminomethylidene)guanidine hydrochloride (0.364 g, 1.60 mmol). The title compound was obtained after refluxing with ethanol (1:21) in an ultrasonic bath for 10 minutes. Yield 0.297 g (55%); m.p. 260.5–262.8 °C; IR (KBr): 3336, 3258, 3191 (N-H), 2952, 2932, 2901 (C-H), 1592, 1554, 1523 (C=N, $C=C_{Ar}$), 1264, 1167 (SO_2) cm^{-1}; ^1H NMR (500 MHz, DMSO-d_6 in 100 °C) δ: 2.31 (s, 3H, CH_3), 3.30 (t, *J=8.5 Hz,* 2H, imidazolidin), 3.50 (t, *J=8.5 Hz,* 2H, imidazolidin), 3.90 (s, 2H, S-CH_2), 4.47 (s, 2H, C**H**$_2$-C_6H_4-4-CF_3), 4.49 (s, 2H, CH_2Ph), 6.44-6.49 (m, 2H, NH_2), 7.18-7.62 (m, 9H, H_{Ar} and 1H, NH, imidazolidin), 7.72 (s, 1H, H-3), 7.83 (s, 1H, H-6) ppm; Anal. calcd. for $C_{29}H_{28}ClF_3N_8O_2S_2$ (677.16); C, 51.44; H, 4.17; N, 16.55. Found: C, 51.11; H, 4.06; N, 16.38; MALDI-TOF/TOF (676.1417) calcd for $C_{29}H_{28}ClF_3N_8O_2S_2$ $[M+H]^+$ (677.1495) found 677.1663.

2-[{4-Amino-6-[(phenyl)(methyl)amino]-1,3,5-triazin-2-yl}methylthio]-4-chloro-5-methyl-N-{1-[4-(trifluoromethyl)benzyl]imidazolidin-2-ylidene}benzenesulfonamide (**128**).

Starting from 1-methyl-1-phenylbiguanide hydrochloride (0.364 g, 1.60 mmol). The title compound was obtained after refluxing with ethanol (1:30) in an ultrasonic bath for 10 minutes and remained part by crystallization from filtrate. Yield 0.266 g (49%); m.p. 178–179 °C; IR (KBr): 3393, 3310, 3203 (N-H), 2970, 2932, 2912, 2855 (C-H), 1579, 1560, 1520 (C=N, $C=C_{Ar}$), 1278, 1165 (SO_2) cm^{-1}; ^1H NMR (500 MHz, DMSO-d_6) δ: 2.30 (s, 3H, CH_3Ph), 3.32-3.37 (m, 2H, imidazolidin and 3H, N-C**H**$_3$), 3.45-3.48 (m, 2H, imidazolidin), 3.93 (s, 2H, S-CH_2), 4.44-4.47 (m, 2H, C**H**$_2$-C_6H_4-4-CF_3), 6.93-7.01 (m, 2H, NH_2), 7.18-7.64 (m, 9H, H_{Ar} and 1H, NH, imidazolidin), 7.79 (s, 1H, H-3), 7.84 (s, 1H, H-6). Anal. calcd. for $C_{29}H_{28}ClF_3N_8O_2S_2$ (677.16); C, 51.44; H, 4.17; N, 16.55. Found: C, 51.16; H, 4.12; N, 16.23. HRMS (ESI-TOF) (677.1632) calcd for $C_{29}H_{28}ClF_3N_8O_2S_2$ $[M+H]^+$ (677.1495) found 677.1487.

2-[{4-Amino-6-[(4-chlorophenyl)(methyl)amino]-1,3,5-triazin-2-yl}methylthio]-4-chloro-5-methyl-N-{1-[4-(trifluoromethyl)benzyl]imidazolidin-2-ylidene}benzenesulfonamide (**129**).

Starting from 1-(4-chlorophenyl)-1-methylbiguanide hydrochloride (0.419 g, 1.60 mmol). The title compound was obtained after refluxing with ethanol (1:29) in an ultrasonic bath for 10 minutes. Yield 0.263 g (46%); m.p. 189–190 °C; IR (KBr): 3300, 3189, 3136 (N-H), 2970, 2946, 2915, 2857 (C-H), 1580, 1560, 1519, 1492 (C=N, $C=C_{Ar}$), 1279, 1166 (SO_2) cm^{-1}; ^1H NMR (500 MHz, DMSO-d_6) δ: 2.30 (s, 3H, CH_3Ph), 3.32-3.36 (m, 2H, imidazolidin and 3H, N-C**H**$_3$), 3.45-3.48 (m, 2H, imidazolidin), 3.93 (s, 2H, S-CH_2), 4.44-

4.47 (m, 2H, C**H**$_2$-C_6H_4-4-CF_3), 7.02-7.06 (m, 2H, NH_2), 7.28-7.76 (m, 8H, H_{Ar} and 1H, NH, imidazolidin and 1H, H-3), 7.84 (s, 1H, H-6) ppm; Anal. calcd. for $C_{29}H_{27}Cl_2F_3N_8O_2S_2$ (711.61); C, 48.95; H, 3.82; N, 15.75. Found: C, 48.98; H, 3.81; N, 15.73; MALDI-TOF/TOF (710.1028) calcd for $C_{29}H_{27}Cl_2F_3N_8O_2S_2$ $[M+H]^+$ (711.1106) found 711.1132.

2-{[4-Amino-6-(4-methylpiperazin-1-yl)-1,3,5-triazin-2-yl]methylthio}-4-chloro-5-methyl-N-{1-[4-(trifluoromethyl)benzyl]imidazolidin-2-ylidene}benzenesulfonamide (**130**).

Starting from *N*-carbamimidoyl-4-methylpiperazine-1-carboximidamide hydrochloride (0.353 g, 1.60 mmol). The title compound was obtained by crystallization with ethanol (1:10), remained part by crystallization from filtrate. Yield 0.233 g (44%); m.p. 134–135 °C. IR (KBr): 3364, 3222 (N-H), 2942, 2922, 2878, 2861 (C-H), 1589, 1561, 1522, 1504 (C=N, C=C_{Ar}), 1279, 1148 (SO_2) cm^{-1}; ^1H NMR (500 MHz, DMSO-d_6) δ: 2.15 (s, 3H, C**H**$_3$-piperazine), 2.18-2.26 (m, 4H, piperazine), 2.30 (s, 3H, CH_3Ph), 3.30-3.32 (m, 2H, imidazolidin), 3.46 (t, *J=8.55 Hz*, 2H, imidazolidin), 3.61-3.68 (m, 4H, piperazine), 3.93 (s, 2H, S-CH_2), 4.46 (s, 2H, C**H**$_2$-C_6H_4-4-CF_3), 6.91-7.65 (m, 4H, H_{Ar} and 2H, NH_2 and 1H, NH, imidazolidin), 7.84 (s, 1H, H-3), 7.92 (s, 1H, H-6) ppm; Anal. calcd. for $C_{27}H_{31}ClF_3N_9O_2S_2$ (670.17); C, 48.39; H, 4.66; N, 18.81. Found: C, 48.27; H, 4.68; N, 18.42; MALDI-TOF/TOF (669.1683) calcd for $C_{27}H_{31}ClF_3N_9O_2S_2$ $[M+H]^+$ (670.1761) found 670.0896.

2-{[4-Amino-6-(4-phenylpiperazin-1-yl)-1,3,5-triazin-2-yl]methylthio}-4-chloro-5-methyl-N-{1-[4-(trifluoromethyl)benzyl]imidazolidin-2-ylidene}benzenesulfonamide (**131**).

Starting from *N*-carbamimidoyl-4-phenylpiperazine-1-carboximidamide hydrochloride (0.452 g, 1.60 mmol). The title compound was obtained by crystallization from ethanol (1:24). Yield 0.341 g (58%); m.p. 160–161 °C; IR (KBr): 3353, 3227, 3188 (N-H), 2983, 2922, 2904, 2874 (C-H), 1595, 1565, 1549, 1526 (C=N, C=C_{Ar}), 1266, 1164 (SO_2) cm^{-1}; ^1H NMR (500 MHz, DMSO-d_6) δ: 2.31 (s, 3H, CH_3Ph), 3.07-3.09 (m, 4H, piperazine), 3.31-3.33 (m, 2H, imidazolidin), 3.44-3.47 (m, 2H, imidazolidin), 3.82 (s, 4H, piperazine), 3.96 (s, 2H, S-CH_2), 4.47 (s, 2H, C**H**$_2$-C_6H_4-4-CF_3), 6.80-7.65 (m, 9H, H_{Ar} and 2H, NH_2 and 1H, NH, imidazolidin), 7.85 (s, 1H, H-3), 7.93 (s, 1H, H-6) ppm; Anal. calcd. for $C_{32}H_{33}ClF_3N_9O_2S_2$ (732.24); C, 52.49; H, 4.54; N, 17.22. Found: C, 52.19; H, 4.53; N, 17.12. HRMS (ESI-TOF) (731.1839) calcd for $C_{32}H_{33}ClF_3N_9O_2S_2$ $[M+H]^+$ (732.1917) found 732.1902.

2-[{4-Amino-6-[4-(4-fluorophenyl)piperazin-1-yl]-1,3,5-triazin-2-yl}methylthio]-4-chloro-5-methyl-N-{1-[4-(trifluoromethyl)benzyl]imidazolidin-2-ylidene}-benzenesulfonamide (**132**).

Starting from *N*-carbamimidoyl-4-(4-fluorophenyl)piperazine-1-carboximidamide hydrochloride (0.481 g, 1.60 mmol). The title compound was obtained after refluxing with ethanol (1:24) in an ultrasonic bath for 10 minutes. Yield 0.378 g (58%); m.p. 205–206 °C; IR (KBr): 3311, 3196, 3131 (N-H), 2980, 2912, 2883, 2866, (C-H), 1576, 1535, 1515, 1488 (C=N, C=C_{Ar}), 1237, 1128 (SO_2) cm^{-1}; ^1H NMR (500 MHz, DMSO-d_6) δ: 2.31 (s, 3H, CH_3Ph), 2.95-3.09 (m, 4H, piperazine), 3.31-3.33 (m, 2H, imidazolidin), 3.46 (t, *J=8.8 Hz*, 2H, imidazolidin), 3.78-3.87 (m, 4H, piperazine), 3.96 (s, 2H, S-CH_2), 4.47 (s, 2H, C**H**$_2$-C_6H_4-4-CF_3), 6.95-7.65 (m, 8H, H_{Ar} and 2H, NH_2 and 1H, NH, imidazolidin), 7.85 (s, 1H, H-3), 7.93 (s, 1H, H-6) ppm; Anal. calcd. for $C_{32}H_{32}ClF_4N_9O_2S_2$ (750.23); C, 51.23; H, 4.30; N, 16.80. Found: C, 51.24; H, 4.32; N, 16.80. HRMS (ESI-TOF) (749.1745) calcd for $C_{32}H_{32}ClF_4N_9O_2S_2$ $[M+H]^+$ (750.1823) found 750.1850.

2-[{4-Amino-6-[4-(2-fluorophenyl)piperazin-1-yl]-1,3,5-triazin-2-yl}methylthio]-4-chloro-5-methyl-N-{1-[4-(trifluoromethyl)benzyl]imidazolidin-2-ylidene}benzenesulfonamide (**133**).

Starting from *N*-carbamimidoyl-4-(2-fluorophenyl)piperazine-1-carboximidamide hydrochloride (0.481 g, 1.60 mmol). The title compound was obtained by crystallization from ethanol (1:19), remained part by crystallization from filtrate. Yield 0.343 g (52%); m.p. 190–192 °C with; IR (KBr): 3373, 3214, 3153 (N-H), 2978, 2903, 2863 (C-H), 1598, 1571, 1547 1522 (C=N, C=C_{Ar}), 1272, 1126 (SO_2) cm^{-1}; ^1H NMR (500 MHz, DMSO-d_6) δ: 2.30 (s, 3H, CH_3Ph), 2.92-2.97 (m, 4H, piperazine), 3.31-3.33 (m, 2H, imidazolidin), 3.46 (t, *J=8.8 Hz*, 2H, imidazolidin), 3.83 (s, 4H, piperazine), 3.96 (s, 2H, S-CH_2), 4.47 (s, 2H, C**H**$_2$-C_6H_4-4-

CF_3), 6.98-7.65 (m, 8H, H_{Ar} and 2H, NH_2 and 1H, NH, imidazolidin), 7.85 (s, 1H, H-3), 7.92 (s, 1H, H-6) ppm; Anal. calcd. for $C_{32}H_{32}ClF_4N_9O_2S_2$ (750.23); C, 51.23; H, 4.30; N, 16.80. Found: C, 51.26; H, 4.34; N, 16.79. HRMS (ESI-TOF) (749.1745) calcd for $C_{32}H_{32}ClF_4N_9O_2S_2$ $[M+H]^+$ (750.1823) found 750.1836.

2-{[4-Amino-6-{4-[4-(trifluoromethyl)phenyl]piperazin-1-yl}-1,3,5-triazin-2-yl]methylthio}-4-chloro-5-methyl-N-{1-[4-(trifluoromethyl)benzyl]imidazolidin-2-ylidene}benzenesulfonamide (**134**).

Starting from *N*-carbamimidoyl-4-[4-(trifluoromethyl)phenyl]piperazine-1-carboximidamide hydrochloride (0.561 g, 1.60 mmol). The title compound was obtained after refluxing with ethanol (1:23) in an ultrasonic bath for 10 minutes. Yield 0.395 g (68%); m.p. 239–240 °C; IR (KBr): 3389, 3323, 3222 (N-H), 2948, 2918, 2884, 2851 (C-H), 1556, 1544, 1520 (C=N, $C=C_{Ar}$), 1286, 1166 (SO_2) cm^{-1}; ^1H NMR (500 MHz, DMSO-d_6) δ: 2.31 (s, 3H, CH_3Ph), 3.20-3.31 (m, 4H, piperazine and 2H, imidazolidin), 3.46 (t, *J=8.5 Hz*, 2H, imidazolidin), 3.82 (s, 4H, piperazine), 3.96 (s, 2H, S-CH_2), 4.47 (s, 2H, C**H**$_2$-C_6H_4-4-CF_3), 7.00-7.65 (m, 8H, H_{Ar} and 2H, NH_2 and 1H, NH, imidazolidin), 7.84 (s, 1H, H-3), 7.93 (s, 1H, H-6) ppm; Anal. calcd. for $C_{33}H_{32}ClF_6N_9O_2S_2$ (800.24); C, 49.53; H, 4.03; N, 15.75. Found: C, 49.49; H, 4.02; N, 15.65. HRMS (ESI-TOF) (799.1713) calcd for $C_{33}H_{32}ClF_6N_9O_2S_2$ $[M+H]^+$ (800.1791) found 800.1797.

2-{[4-Amino-6-{4-[3-(trifluoromethyl)phenyl]piperazin-1-yl}-1,3,5-triazin-2-yl]methylthio}-4-chloro-5-methyl-N-{1-[4-(trifluoromethyl)benzyl]imidazolidin-2-ylidene}benzenesulfonamide (**135**).

Starting from *N*-carbamimidoyl-4-[3-(trifluoromethyl)phenyl]piperazine-1-carboximidamide hydrochloride (0.561 g, 1.60 mmol). The title compound was obtained after refluxing with ethanol (1:23) in an ultrasonic bath for 10 minutes. Yield 0.355 g (56%); m.p. 168–169 °C; IR (KBr): 3388, 3302, 3187 (N-H), 2975, 2934, 2912, 2889, 2856 (C-H), 1589, 1557, 1534, 1518 (C=N, $C=C_{Ar}$), 1257, 1151 (SO_2) cm^{-1}; ^1H NMR (500 MHz, DMSO-d_6) δ: 2.30 (s, 3H, CH_3Ph), 3.18-3.21 (m, 4H, piperazine), 3.31-3.33 (m, 2H, imidazolidin), 3.46 (t, *J=8.8 Hz*, 2H, imidazolidin), 3.82 (s, 4H, piperazine), 3.96 (s, 2H, S-CH_2), 4.47 (s, 2H, C**H**$_2$-C_6H_4-4-CF_3), 7.00-7.64 (m, 8H, H_{Ar} and 2H, NH_2 and 1H, NH, imidazolidin), 7.84 (s, 1H, H-3), 7.93 (s, 1H, H-6) ppm; Anal. calcd. for $C_{33}H_{32}ClF_6N_9O_2S_2$ (800.24); C, 49.53; H, 4.03; N, 15.75. Found: C, 49.51; H, 3.96; N, 15.75; MALDI-TOF/TOF (799.1713) calcd for $C_{33}H_{32}ClF_6N_9O_2S_2$ $[M+H]^+$ (800.1791) found 800.1104.

2-[{4-Amino-6-[4-(4-chlorophenyl)piperazin-1-yl]-1,3,5-triazin-2-yl}methylthio]-4-chloro-5-methyl-N-{1-[4-(trifluoromethyl)benzyl]imidazolidin-2-ylidene}benzenesulfonamide (**136**).

Starting from *N*-carbamimidoyl-4-(4-chlorophenyl)piperazine-1-carboximidamide hydrochloride (0.508 g, 1.60 mmol). The title compound was obtained after refluxing with ethanol (1:33) in an ultrasonic bath for 10 minutes. Yield 0.401 g (65%); m.p. 191–192 °C; IR (KBr): 3308, 3210, 3112 (N-H), 2955, 2911, 2881, 2833 (C-H), 1576, 1516 (C=N, $C=C_{Ar}$), 1274, 1167 (SO_2) cm^{-1}; ^1H NMR (500 MHz, DMSO-d_6) δ: 2.30 (s, 3H, CH_3Ph), 3.04-3.15 (m, 4H, piperazine), 3.31-3.33 (m, 2H, imidazolidin), 3.46 (t, *J=8.8 Hz*, 2H, imidazolidin), 3.77-3.84 (m, 4H, piperazine), 3.95 (s, 2H, S-CH_2), 4.47 (s, 2H, C**H**$_2$-C_6H_4-4-CF_3), 6.95-7.65 (m, 8H, H_{Ar} and 2H, NH_2 and 1H, NH, imidazolidin), 7.84 (s, 1H, H-3), 7.93 (s, 1H, H-6) ppm; Anal. calcd. for $C_{32}H_{32}Cl_2F_3N_9O_2S_2$ (766.69); C, 50.13; H, 4.21; N, 16.44. Found: C, 50.10; H, 4.18; N, 16.44; MALDI-TOF/TOF (765.1450) calcd for $C_{32}H_{32}Cl_2F_3N_9O_2S_2$ $[M+H]^+$ (766.1528) found 766.1793.

2-[{4-Amino-6-[4-(3-chlorophenyl)piperazin-1-yl]-1,3,5-triazin-2-yl}methylthio]-4-chloro-5-methyl-N-{1-[4-(trifluoromethyl)benzyl]imidazolidin-2-ylidene}benzenesulfonamide (**137**).

Starting from *N*-carbamimidoyl-4-(3-chlorophenyl)piperazine-1-carboximidamide hydrochloride (0.508 g, 1.60 mmol). The title compound was obtained after refluxing with mixture 20:1 ethanol/benzene (1:50) in an ultrasonic bath for 10 minutes. Yield 0.339 g (55%); m.p. 190–191 °C; IR (KBr): 3391, 3305, 3128 (N-H), 2930, 2910, 2874, 2859 (C-H), 1576, 1536, 1518, 1472 (C=N, $C=C_{Ar}$), 1163, 1257 (SO_2) cm^{-1}; ^1H NMR (500 MHz, DMSO-d_6) δ: 2.31 (s, 3H, CH_3Ph), 3.09-3.21 (m, 4H, piperazine), 3.30-3.32 (m, 2H, imidazolidin), 3.46 (t, *J=5.9 Hz*, 2H, imidazolidin), 3.76-3.84 (m, 4H, piperazine), 3.96 (s, 2H, S-CH_2), 4.47 (s, 2H, C**H**$_2$-C_6H_4-4-CF_3), 6.81-7.65 (m, 8H, H_{Ar} and 2H, NH_2 and 1H, NH, imidazolidin), 7.84

(s, 1H, H-3), 7.93 (s, 1H, H-6) ppm; Anal. calcd. for $C_{32}H_{32}Cl_2F_3N_9O_2S_2$ (766.69); C, 50.13; H, 4.21; N, 16.44. Found: C, 49.86; H, 4.10; N, 16.09; MALDI-TOF/TOF (765.1450) calcd for $C_{32}H_{32}Cl_2F_3N_9O_2S_2$ $[M+H]^+$ (766.1528) found 766.1521.

2-[{4-Amino-6-[4-(3,4-dichlorophenyl)piperazin-1-yl]-1,3,5-triazin-2-yl}methylthio]-4-chloro-5-methyl-N-{1-[4-(trifluoromethyl)benzyl]imidazolidin-2-ylidene}benzenesulfonamide (**138**).

Starting from *N*-carbamimidoyl-4-(3,4-dichlorophenyl)piperazine-1-carboximidamide hydrochloride (0.563 g, 1.60 mmol). The title compound was obtained after refluxing with ethanol (1:35) in an ultrasonic bath for 10 minutes. Yield 0.460 g (64%); m.p. 221–222 °C; IR (KBr): 3394, 3327, 3227 (N-H), 2984, 2916, 2857, 2887 (C-H), 1555, 1544, 1519, 1482 (C=N, C=C_{Ar}), 1284, 1168 (SO_2) cm^{-1}; ^1H NMR (500 MHz, DMSO-d_6) δ: 2.31 (s, 3H, CH_3Ph), 3.11-3.22 (m, 4H, piperazine), 3.33-3.35 (m, 2H, imidazolidin), 3.44-3.48 (m, 2H, imidazolidin), 3.80 (s, 4H, piperazine), 3.95 (s, 2H, S-CH_2), 4.47 (s, 2H, C**H**$_2$-C_6H_4-4-CF_3), 6.93-7.64 (m, 7H, H_{Ar} and 2H, NH_2 and 1H, NH, imidazolidin), 7.84 (s, 1H, H-3), 7.93 (s, 1H, H-6) ppm; Anal. calcd. for $C_{32}H_{31}Cl_3F_3N_9O_2S_2$ (801.13); C, 47.97; H, 3.90; N, 15.74. Found: C, 47.69; H, 3.81; N, 15.56. HRMS (ESI-TOF) (799.1060) calcd for $C_{32}H_{31}Cl_3F_3N_9O_2S_2$ $[M+H]^+$ (800.1138) found 800.1206.

2-[{4-Amino-6-[4-(4-methoxyphenyl)piperazin-1-yl]-1,3,5-triazin-2-yl}methylthio]-4-chloro-5-methyl-N-{1-[4-(trifluoromethyl)benzyl]imidazolidin-2-ylidene}benzenesulfonamide (**139**).

Starting from *N*-carbamimidoyl-4-(4-methoxyphenyl)piperazine-1-carboximidamide hydrochloride (0.500 g, 1.60 mmol). The title compound was obtained by crystallization from ethanol (1:23), remained part by crystallization from filtrate. Yield 0.376 g (62%); m.p. 162–163 °C; IR (KBr): 3319, 3217, 3149 (N-H), 2971, 2925, 2894, 2859 (C-H), 1584, 1564, 1544, 1511 (C=N, C=C_{Ar}), 1277, 1166 (SO_2) cm^{-1}; ^1H NMR (500 MHz, DMSO-d_6) δ: 2.30 (s, 3H, CH_3Ph), 2.92-2.96 (m, 4H, piperazine), 3.31-3.33 (m, 2H, imidazolidin), 3.46 (t, *J=8.75 Hz*, 2H, imidazolidin), 3.69 (s, 3H, O-C**H**$_3$), 3.78-3.83 (m, 4H, piperazine), 3.95 (s, 2H, S-CH_2), 4.46 (s, 2H, C**H**$_2$-C_6H_4-4-CF_3), 6.82-7.65 (m, 8H, H_{Ar} and 2H, NH_2 and 1H, NH, imidazolidin), 7.84 (s, 1H, H-3), 7.93 (s, 1H, H-6) ppm; Anal. calcd. for $C_{33}H_{35}ClF_3N_9O_3S_2$ (762.2677); C, 52.00; H, 4.63; N, 16.54. Found: C, 52.04; H, 4.65; N, 16.54. HRMS (ESI-TOF) (761.1945) calcd for $C_{33}H_{35}ClF_3N_9O_3S_2$ $[M+H]^+$ (762.2023) found 762.2108.

2-{[4-Amino-6-(4-benzylpiperazin-1-yl)-1,3,5-triazin-2-yl]methylthio}-4-chloro-5-methyl-N-{1-[4-(trifluoromethyl)benzyl]imidazolidin-2-ylidene}benzenesulfonamide (**140**).

Starting from 4-benzyl-*N*-carbamimidoylpiperazine-1-carboximidamide hydrochloride (0.475 g, 1.60 mmol). The title compound was obtained by crystallization from EtOH (1:19). Yield 0.286 g (55%); m.p. 168–170 °C; IR (KBr): 3376, 3173 (N-H), 2967, 2922, 2869 (C-H), 1579, 1569, 1524, 1494 (C=N, C=C_{Ar}), 1278, 1166 (SO_2) cm^{-1}; ^1H NMR (500 MHz, DMSO-d_6) δ: 2.19-2.33 (m, 3H, CH_3Ph and 4H, piperazine), 3.30-3.33 (m, 2H, imidazolidin), 3.43-3.47 (m, 2H, imidazolidin and 2H, Ph-CH_2), 3.63-3.68 (m, 4H, piperazine), 3.92 (s, 2H, S-CH_2), 4.44 (s, 2H, C**H**$_2$$C_6H_4$-4-$CF_3$), 6.92-6.96 (m, 2H, NH_2), 7.30-7.64 (m, 9H, H_{Ar} and 1H, NH, imidazolidin), 7.83 (s, 1H, H-3), 7.90 (s, 1H, H-6) ppm; ^{13}C NMR (125 MHz, DMSO-d_6) δ: 19.39, 41.36, 42.85, 43.16, 44.93, 47.18, 52.59, 52.89, 62.45, 121.44, 123.60, 125.77, 125.80, 125.83, 125.86, 127.49, 128.21, 128.37, 128.69, 128.95, 129.32, 130.71, 131.86, 136.60, 136.94, 138.37, 139.25, 141.77, 158.62, 164.60, 167.36, 174.22 ppm; Anal. calcd. for $C_{33}H_{35}ClF_3N_9O_2S_2$ (746.27); C, 53.11; H, 4.73; N, 16.89. Found: C, 52.81; H, 4.60; N, 16.71. HRMS (ESI-TOF) (745.1996) calcd for $C_{33}H_{35}ClF_3N_9O_2S_2$ $[M+H]^+$ (746.2074) found 746.2096.

2-{[4-Amino-6-(4-benzhydrylpiperazin-1-yl)-1,3,5-triazin-2-yl]methylthio}-4-chloro-5-methyl-N-{1-[4-(trifluoromethyl)benzyl]imidazolidin-2-ylidene}benzenesulfonamide (**141**).

Starting from 4-benzhydryl-*N*-carbamimidoylpiperazine-1-carboximidamide hydrochloride (0.597 g, 1.60 mmol). The title compound was obtained after refluxing with ethanol (1:21) in an ultrasonic bath for 10 minutes. Yield 0.455 g (69%); m.p. 205–206 °C; IR (KBr): 3377, 3158 (N-H), 2965, 2924, 2894, 2862 (C-H), 1593, 1583, 1516, 1488 (C=N, C=C_{Ar}), 1270, 1163 (SO_2) cm^{-1}; ^1H NMR (500 MHz, DMSO-d_6) δ: 2.12-

2.30 (m, 4H, piperazine and 3H, CH_3Ph), 3.29 (t, *J=8.8 Hz*, 2H, imidazolidin), 3.44 (t, *J=8.75 Hz*, 2H, imidazolidin), 3.59-3.68 (m, 4H, piperazine), 3.92 (s, 2H, S-CH_2), 4.26 (s, 1H, **C**HPh_2), 4.41 (s, 2H, **C**H$_2$-C_6H_4-4-CF_3), 6.95-7.62 (m, 14H, H_{Ar} and 1H, NH, imidazolidin and 2H, NH_2), 7.80 (s, 1H, H-3), 7.85 (s, 1H, H-6) ppm; Anal. calcd. for $C_{39}H_{39}ClF_3N_9O_2S_2$ (822.36); C, 56.96; H, 4.78; N, 15.33. Found: C, 57.04; H, 4.81; N, 15.24; MALDI-TOF/TOF (821.2309) calcd for $C_{39}H_{39}ClF_3N_9O_2S_2$ $[M+H]^+$ (822.2387) found 822.0657.

2-{[4-Amino-6-(4-benzylpiperidin-1-yl)-1,3,5-triazin-2-yl]methylthio}-4-chloro-5-methyl-N-{1-[4-(trifluoromethyl)benzyl]imidazolidin-2-ylidene}benzenesulfonamide (**142**).

Starting from 4-benzyl-*N*-carbamimidoylpiperidine-1-carboximidamide hydrochlorid (0.473 g, 1.60 mmol). The title compound was obtained after refluxing with ethanol (1:25) in an ultrasonic bath for 10 minutes. Yield 0.374 g (63%); m.p. 200–201 °C; IR (KBr): 3382, 3155 (N-H), 2942, 2925, 2852 (C-H), 1599, 1562, 1549, 1522,(C=N, C=C_{Ar}), 1270, 1160 (SO_2) cm^{-1}; ^{1}H NMR (500 MHz, DMSO-d_6) δ: 0.93-0.96 (m, 2H, piperidine C3 and C4), 1.49-1.72 (m, 2H, piperidine C3 and C5), 1.73-1.76 (m, 1H, piperidine C4), 2.29 (s, 3H, CH_3Ph), 2.45-2.47 (m, 2H, piperidine-C**H$_2$**Ph), 2.66-2.70 (m, 2H, piperidine C2 and C6), 3.30-3.33 (m, 2H, imidazolidin), 3.46 (t, *J=8.75 Hz*, 2H, imidazolidin), 3.92 (s, 2H, S-CH_2), 4.44 (s, 2H, C**H$_2$**-C_6H_4-4-CF_3), 4.55-4.57 (m, 2H, piperidine C2 and C6), 6.88-7.64 (m, 9H, H_{Ar} and 1H, NH, imidazolidin and 2H, NH_2), 7.83 (s, 1H, H-3), 7.91 (s, 1H, H-6) ppm; Anal. calcd. for $C_{34}H_{36}ClF_3N_8O_2S_2$ (745.28); C, 54.79; H, 4.87; N, 15.04. Found: C, 54.66; H, 4.69; N, 14.82; MALDI-TOF/TOF (744.2043) calcd for $C_{34}H_{36}ClF_3N_8O_2S_2$ $[M+H]^+$ (745.2121) found 745.0301.

2-[{4-Amino-6-[4-(4-fluorophenyl)-5,6-dihydropyridin-1(2H)-yl]-1,3,5-triazin-2-yl}methylthio]-4-chloro-5-methyl-N-{1-[4-(trifluoromethyl)benzyl]imidazolidin-2-ylidene}benzenesulfonamide (**143**).

Starting from *N*-carbamimidoyl-4-(4-fluorophenyl)-5,6-dihydropyridine-1(2*H*)-carboximidamide hydrochloride (0.476 g, 1.60 mmol). The title compound was obtained after refluxing with mixture 1:1 ethanol/acetonitrile (1:21) in an ultrasonic bath for 10 minutes. Yield 0.321 g (54%); m.p. 237–238 °C; IR (KBr): 3309, 3224, 3194 (N-H), 2928, 2911, 2882, 2855 (C-H), 1573, 1536, 1513 (C=N, C=C_{Ar}), 1277, 1168 (SO_2) cm^{-1}; ^{1}H NMR (500 MHz, DMSO-d_6) δ: 2.31 (s, 3H, CH_3), 2.30-2.37 (m, 2H, $CH2_{C5}$), 3.30-3.31 (m, 2H, imidazolidin), 3.45 (t, *J=8.55 Hz*, 2H, imidazolidin), 3.86-3.92 (m, 2H, $CH2_{C2}$), 3.96 (s, 2H, S-CH_2), 4.24-4.31 (m, 2H, $CH2_{C6}$), 4.44 (s, 2H, C**H$_2$**-C_6H_4-4-CF_3), 6.11-6.18 (m, 1H, CH_{C3}), 6.94-7.02 (m, 2H, NH_2), 7.15-7.64 (m, 8H, H_{Ar} and 1H, NH, imidazolidin), 7.85 (s, 1H, H-3), 7.96 (s, 1H, H-6) ppm; Anal. calcd. for $C_{33}H_{31}ClF_4N_8O_2S_2$ (747.23); C, 53.04; H, 4.18; N, 15.00. Found: C, 52.73; H, 4.12; N, 14.78; MALDI-TOF/TOF (746.1636) calcd for $C_{33}H_{31}ClF_4N_8O_2S_2$ $[M+H]^+$ (747.1714) found 747.1020.

2-{[4-Amino-6-(indolin-1-yl)-1,3,5-triazin-2-yl]methylthio}-N-{1-[3,5-bis(trifluoromethyl)benzyl]imidazolidin-2-ylidene}-4-chloro-5-methylbenzenesulfonamide (**144)**.

Starting from *N*-carbamimidoylindoline-1-carboximidamide hydrochloride (0.384 g, 1.60 mmol). The title compound was obtained by crystallization from ethanol (1:22). Yield 0.324 g (54%); m.p. 210–211 °C; IR (KBr): 3388, 3317, 3144 (N-H), 2985, 2945, 2926, 2866 (C-H), 1584, 1577, 1523, 1481 (C=N, C=C_{Ar}), 1277, 1166 (SO_2) cm^{-1}; ^{1}H NMR (500 MHz, DMSO-d_6) δ: 2.30 (s, 3H, CH_3), 3.06 (t, *J=8.55 Hz*, 2H, hydroindoline), 3.28-3.33 (m, 2H, imidazolidin), 3.41-3.47 (m, 2H, imidazolidin), 3.85-4.05 (m, 2H, hydroindoline and 2H, S-CH_2), 4.49-4.58 (m, 2H, C**H$_2$**-C_6H_3-3,5-bis(CF_3)), 6.89-8.42 (m, 7H, H_{Ar} and 2H, NH_2 and 1H, NH, imidazolidin and 1H, H-3 and 1H, H-6) ppm; Anal. calcd. for $C_{31}H_{27}ClF_6N_8O_2S_2$ (757.19); C, 49.17; H, 3.59; N, 14.80. Found: C, 49.16; H, 3.54; N, 14.74; MALDI-TOF/TOF (756.1291) calcd for $C_{31}H_{27}ClF_6N_8O_2S_2$ $[M+H]^+$ (757.1369) found 757.0510.

2-{[4-Amino-6-(3,4-dihydroquinolin-1(2H)-yl)-1,3,5-triazin-2-yl]methylthio}-N-{1-[3,5-bis(trifluoromethyl)benzyl]imidazolidin-2-ylidene}-4-chloro-5-methylbenzenesulfonamide (**145**).

Starting from *N*-carbamimidoyl-3,4-dihydroquinoline-1(2*H*)-carboximidamide hydrochloride (0.406 g, 1.60 mmol). The title compound was obtained after refluxing with ethanol (1:35) in an

ultrasonic bath for 10 minutes. Yield 0.119 g (19%); m.p. 180–181 °C; IR (KBr): 3395, 3323, 3216 (N-H), 2946, 2926, 2896, 2853 (C-H), 1584, 1558, 1523, 1489 (C=N, C=C_{Ar}), 1279, 1133 (SO_2) cm^{-1}; ^1H NMR (500 MHz, DMSO-d_6) δ: 1.80 (quintet, *J=6.35 Hz*, 2H, 1,2,3,4-tetrahydroquinoline), 2.31 (s, 3H, CH_3), 2.69 (t, *J=6.85 Hz*, 2H, 1,2,3,4-tetrahydroquinoline), 3.33-3.37 (m, 2H, imidazolidin), 3.45 (t, *J=8.55 Hz*, 2H, imidazolidin), 3.87 (t, *J=5.85 Hz*, 2H, 1,2,3,4-tetrahydroquinoline), 3.93 (s, 2H, S-CH_2), 4.53 (s, 2H, C**H**$_2$-C_6H_3-3,5-bis(CF_3)), 6.94-8.00 (m, 8H, H_{Ar} and 2H, NH_2 and 1H, NH, imidazolidin and 1H, H-3 and 1H, H-6) ppm; Anal. calcd. for $C_{32}H_{29}ClF_6N_8O_2S_2$ (771.20); C, 49.84; H, 3.79; N, 14.53. Found: C, 49.27; H, 3.77; N, 13.97; MALDI-TOF/TOF (770.1448) calcd for $C_{32}H_{29}ClF_6N_8O_2S_2$ $[M+H]^+$ (771.1526) found 771.1052.

2-{[4-Amino-6-(phenylamino)-1,3,5-triazin-2-yl]methylthio}-N-{1-[3,5-bis(trifluoromethyl)benzyl]imidazolidin-2-ylidene}-4-chloro-5-methylbenzenesulfonamide (**146**).

Starting from 1-phenylbiguanide hydrochloride (0.342 g, 1.60 mmol). The title compound was obtained. Yield 0.346 g (59%); m.p. 199–201 °C; IR (KBr): 3346, 3321, 3219 (N-H), 2974, 2930, 2901 (C-H), 1588, 1557, 1533, 1486 (C=N, C=C_{Ar}), 1281, 1146 (SO_2) cm^{-1}; ^1H NMR (500 MHz, DMSO-d_6) δ: 2.31 (s, 3H, CH_3), 3.33-3.36 (m, 2H, imidazolidin), 3.46 (t, *J=8.55 Hz*, 2H, imidazolidin), 3.97 (s, 2H, S-CH_2), 4.55 (s, 2H, C**H**$_2$-C_6H_3-3,5-bis(CF_3)), 6.94-7.99 (m, 8H, H_{Ar} and 1H, NH, imidazolidin and 1H, H-3 and 1H, H-6 and 1H, NH_2), 9.48-9.57 (m, 1H, NH) ppm; Anal. calcd. for $C_{29}H_{25}ClF_6N_8O_2S_2$ (731.13); C, 47.64; H, 3.45; N, 15.33. Found: C, 47.31; H, 3.39; N, 14.83; MALDI-TOF/TOF (730.1135) calcd for $C_{29}H_{25}ClF_6N_8O_2S_2$ $[M+H]^+$ (731.1213) found 730.9371.

2-{[4-Amino-6-{[4-(trifluoromethyl)phenyl]amino}-1,3,5-triazin-2-yl]methylthio}-N-{1-[3,5-bis(trifluoromethyl)benzyl]imidazolidin-2-ylidene}-4-chloro-5-methylbenzenesulfonamide (**147**).

Starting from 1-[4-(trifluoromethyl)phenyl]biguanide hydrochloride (0.451 g, 1.60 mmol). The title compound was obtained by crystallization from ethanol (1:21). Yield 0.148 g (26%); m.p. 207–208 °C; IR (KBr): 3327, 3269, 3226 (N-H), 2989, 2965, 2934, 2898 (C-H), 1592, 1553, 1526 (C=N, C=C_{Ar}), 1281, 1170 (SO_2) cm^{-1}; ^1H NMR (500 MHz, DMSO-d_6) δ: 2.30 (s, 3H, CH_3), 3.34-3.35 (m, 2H, imidazolidin), 3.45 (t, *J=8.55 Hz*, 2H, imidazolidin), 4.02 (s, 2H, S-CH_2), 4.54 (s, 2H, C**H**$_2$-C_6H_4-3,5-bis(CF_3)), 7.21-7.97 (m, 7H, H_{Ar} and 2H, NH_2 and 1H, NH, imidazolidin and 1H, H-3 and 1H, H-6), 9.88-9.93 (m, 1H, N**H**-C_6H_4-4-CF_3) ppm; Anal. calcd. for $C_{30}H_{24}ClF_9N_8O_2S_2$ (799.13); C, 45.09; H, 3.03; N, 14.02. Found: C, 44.69; H, 2.89; N, 13.77; MALDI-TOF/TOF (798.1008) calcd for $C_{30}H_{24}ClF_9N_8O_2S_2$ $[M+H]^+$ (799.1086) found 799.1296.

2-{[4-Amino-6-(benzylamino)-1,3,5-triazin-2-yl]methylthio}-N-{1-[3,5-bis(trifluoromethyl)benzyl]imidazolidin-2-ylidene}-4-chloro-5-methylbenzenesulfonamide (**148**).

Starting from 2-benzyl-1-(diaminomethylidene)guanidine hydrochloride (0.364 g, 1.60 mmol). The title compound was obtained by add 3 mL ethanol to afterreaction mixture. Next all was filtred and mixed with 50 mL water during 20 min. Next obtain solid was refluxing with ethanol (1:20) in an ultrasonic bath for 10 minutes. Yield 0.207 g (35%); m.p. 183–184 °C; IR (KBr): 3388, 3342, 3269 (N-H), 2967, 2940, 2902, 2878 (C-H), 1604, 1550, 1512, 1492 (C=N, C=C_{Ar}), 1279, 1170 (SO_2) cm^{-1}; ^1H NMR (500 MHz, DMSO-d_6 in 100 °C) δ: 2.31 (s, 3H, CH_3), 3.41 (t, *J=8.5 Hz,* 2H, imidazolidin), 3.52 (t, *J=8.65 Hz,* 2H, imidazolidin), 3.86 (s, 2H, S-CH_2), 4.47 (d, 2H, C**H**$_2$-C_6H_4-4-CF_3), 4.58 (s, 2H, CH_2Ph), 6.43-6.47 (m, 2H, NH_2), 7.70-7.87 (m, 9H, H_{Ar} and 1H, NH, imidazolidin), 7.84 (s, 1H, H-3), 7.90 (s, 1H, H-6) ppm; Anal. calcd. for $C_{30}H_{27}ClF_6N_8O_2S_2$ (745.16); C, 48.35; H, 3.65; N, 15.04. Found: C, 48.33; H, 3.63; N, 15.04; MALDI-TOF/TOF (744.1291) calcd for $C_{30}H_{27}ClF_6N_8O_2S_2$ $[M+H]^+$ (745.1369) found 745.0521.

2-[{4-Amino-6-[(4-chlorophenyl)(methyl)amino]-1,3,5-triazin-2-yl]methylthio}-N-{1-[3,5-bis(trifluoromethyl)benzyl]imidazolidin-2-ylidene}-4-chloro-5-methylbenzenesulfonamide (**149**).

Starting from 1-(4-chlorophenyl)-1-methylbiguanide hydrochloride (0.419 g, 1.60 mmol)**.** The title compound was obtained after refluxing with ethanol (1:21) in an ultrasonic bath for 10 minutes. Yield 0.353 g (57%); m.p. 214–215 °C; IR (KBr): 3311, 3201, 3133 (N-H), 2947, 2918, 2900, 2855 (C-H), 1599, 1577, 1524, 1491 (C=N, C=C_{Ar}), 1278, 1172 (SO_2) cm^{-1}; ^1H NMR (500 MHz, DMSO-d_6) δ: 2.31 (s, 3H, CH_3Ph),

3.33-3.39 (m, 2H, imidazolidin and 3H, N-C**H**$_3$), 3.48 (t, *J=8.8 Hz*, 2H, imidazolidin), 3.87 (s, 2H, S-CH$_2$), 4.55-4.57 (m, 2H, C**H**$_2$-C$_6$H$_3$-3,5-bis(CF$_3$)), 6.99-7.02 (m, 2H, NH$_2$), 7.28-8.01 (m, 7H, H$_{Ar}$ and 1H, NH, imidazolidin and 1H, H-3 and 1H, H-6) ppm; Anal. calcd. for $C_{30}H_{26}Cl_2F_6N_8O_2S_2$ (779.61); C, 46.22; H, 3.36; N, 14.37. Found: C, 46.20; H, 3.28; N, 14.21; MALDI-TOF/TOF (778.0901) calcd for $C_{30}H_{26}Cl_2F_6N_8O_2S_2$ [M+H]$^+$ (779.0979) found 779.0104.

2-{[4-Amino-6-(4-phenylpiperazin-1-yl)-1,3,5-triazin-2-yl]methylthio}-N-{1-[3,5-bis(trifluoromethyl)benzyl]imidazolidin-2-ylidene}-4-chloro-5-methylbenzenesulfonamide (**150**).

Starting from *N*-carbamimidoyl-4-phenylpiperazine-1-carboximidamide hydrochloride (0.452 g, 1.60 mmol). The title compound was obtained by crystallization from ethanol (1:23). Yield 0.337 g (53%); m.p. 194–195 °C; IR (KBr): 3298, 3116 (N-H), 2945, 2922, 2896, 2853 (C-H), 1580, 1539, 1498 (C=N, C=C$_{Ar}$), 1278, 1139 (SO$_2$) cm^{-1}; ^{1}H NMR (500 MHz, DMSO-*d*$_6$) δ: 2.31 (s, 3H, CH$_3$), 3.07-3.11 (m, 4H, piperazine), 3.36-3.39 (m, 2H, imidazolidin), 3.46-3.49 (m, 2H, imidazolidin), 3.78-3.83 (m, 4H, piperazine), 3.89 (s, 2H, S-CH$_2$), 4.58 (s, 2H, C**H**$_2$-C$_6$H$_3$-3,5-bis(CF$_3$)), 6.80-8.02 (m, 8H, H$_{Ar}$ and 2H, NH$_2$ and 1H, NH, imidazolidin and 1H, H-3 and 1H, H-6) ppm; Anal. calcd. for $C_{33}H_{32}ClF_6N_9O_2S_2$ (800.24); C, 49.53; H, 4.03; N, 15.75. Found: C, 49.49; H, 3.94; N, 15.60; MALDI-TOF/TOF (799.1713) calcd for $C_{33}H_{32}ClF_6N_9O_2S_2$ [M+H]$^+$ (800.1791) found 799.9533.

2-[{4-Amino-6-[4-(4-fluorophenyl)piperazin-1-yl]-1,3,5-triazin-2-yl}methylthio]-N-{1-[3,5-bis(trifluoromethyl)benzyl]imidazolidin-2-ylidene}-4-chloro-5-methylbenzenesulfonamide (**151**).

Starting from *N*-carbamimidoyl-4-(4-fluorophenyl)piperazine-1-carboximidamide hydrochloride (0.481 g, 1.60 mmol). The title compound was obtained after refluxing with ethanol (1:20) in an ultrasonic bath for 10 minutes. Yield 0.289 g (44%); m.p. 180–181 °C; IR (KBr): 3309, 3196 (N-H), 2959, 2920, 2856 (C-H), 1581, 1564, 1542, 1511 (C=N, C=C$_{Ar}$), 1277, 1167 (SO$_2$) cm^{-1}; ^{1}H NMR (500 MHz, DMSO-*d*$_6$) δ: 2.30 (s, 3H, CH$_3$), 2.96-3.09 (m, 4H, piperazine), 3.38 (t, *J=8.8 Hz*, 2H, imidazolidin), 3.47 (t, *J=8.8 Hz*, 2H, imidazolidin), 3.77-3.83 (m, 4H, piperazine), 3.88 (s, 2H, S-CH$_2$), 4.58 (s, 2H, C**H**$_2$-C$_6$H$_3$-3,5-bis(CF$_3$)), 6.95-7.90 (m, 7H, H$_{Ar}$ and 2H, NH$_2$ and 1H, NH, imidazolidin and 1H, H-3), 8.02 (s, 1H, H-6) ppm; Anal. calcd. for $C_{33}H_{31}ClF_7N_9O_2S_2$ (818.23); C, 48.44; H, 3.82; N, 15.41. Found: C, 48.36; H, 3.76; N, 15.27; MALDI-TOF/TOF (817.1619) calcd for $C_{33}H_{31}ClF_7N_9O_2S_2$ [M+H]$^+$ (818.1697) found 817.9797.

2-[{4-Amino-6-[4-(2-fluorophenyl)piperazin-1-yl]-1,3,5-triazin-2-yl}methylthio]-N-{1-[3,5-bis(trifluoromethyl)benzyl]imidazolidin-2-ylidene}-4-chloro-5-methylbenzenesulfonamide (**152**).

Starting from *N*-carbamimidoyl-4-(2-fluorophenyl)piperazine-1-carboximidamide hydrochloride (0.481 g, 1.60 mmol). The title compound was obtained by crystallization from ethanol (1:21), remained part by crystallization from filtrate. Yield 0.326 g (50%); m.p. 166–167 °C with; IR (KBr): 3309, 3202, 3153 (N-H), 2945, 2923, 2862 (C-H), 1580, 1546, 1518, 1500 (C=N, C=C$_{Ar}$), 1277, 1173 (SO$_2$) cm^{-1}; ^{1}H NMR (500 MHz, DMSO-*d*$_6$) δ: 2.30 (s, 3H, CH$_3$), 2.91-2.97 (m, 4H, piperazine), 3.38 (t, *J=8.8 Hz*, 2H, imidazolidin), 3.47 (t, *J=8.55 Hz*, 2H, imidazolidin), 3.80-3.81 (m, 4H, piperazine), 3.89 (s, 2H, S-CH$_2$), 4.58 (s, 2H, C**H**$_2$-C$_6$H$_4$-4-F), 6.95-7.90 (m, 7H, H$_{Ar}$ and 2H, NH$_2$ and 1H, NH, imidazolidin and 1H, H-3), 8.01 (s, 1H, H-6) ppm; Anal. calcd. for $C_{33}H_{31}ClF_7N_9O_2S_2$ (818.23); C, 48.44; H, 3.82; N, 15.41. Found: C, 48.85; H, 4.00; N, 15.48; MALDI-TOF/TOF (817.1619) calcd for $C_{33}H_{31}ClF_7N_9O_2S_2$ [M+H]$^+$ (818.1697) found 818.1389.

2-{[4-Amino-6-{4-[3-(trifluoromethyl)phenyl]piperazin-1-yl}-1,3,5-triazin-2-yl]methylthio}-N-{1-[3,5-bis(trifluoromethyl)benzyl]imidazolidin-2-ylidene}-4-chloro-5-methylbenzenesulfonamide (**153**).

Starting from *N*-carbamimidoyl-4-[3-(trifluoromethyl)phenyl]piperazine-1-carboximidamide hydrochloride (0.561 g, 1.60 mmol). The title compound was obtained after refluxing with ethanol (1:22) in an ultrasonic bath for 10 minutes. Yield 0.338 g (49%); m.p. 196–197 °C; IR (KBr): 3372, 3346, 3232 (N-H), 2983, 2923, 2857 (C-H), 1591, 1577, 1568, 1525 (C=N, C=C$_{Ar}$), 1279, 1171 (SO$_2$) cm^{-1}; ^{1}H NMR (500 MHz, DMSO-*d*$_6$) δ: 2.31 (s, 3H, CH$_3$), 3.15-3.27 (m, 4H, piperazine), 3.38 (t, *J=8.8 Hz*, 2H, imidazolidin), 3.48 (t, *J=8.55 Hz*, 2H, imidazolidin), 3.79-3.84 (m, 4H, piperazine), 3.89 (s, 2H, S-CH$_2$), 4.58 (s, 2H, C**H**$_2$-

C_6H_3-3,5-bis(CF_3)), 6.98-7.90 (m, 7H, H_{Ar} and 2H, NH_2 and 1H, NH, imidazolidin and 1H, H-3), 8.01 (s, 1H, H-6) ppm; ^{13}C NMR (125 MHz, DMSO-d$_6$) δ: 19.36, 41.47, 42.70, 45.22, 46.98, 47.98, 111.82, 115.45, 119.55, 121.78, 122.60, 123.80, 124.77, 125.97, 128.48, 129.10, 130.46, 130.66, 130.97, 132.07, 136.29, 137.04, 139.42, 140.83, 151.59, 158.68, 164.72, 167.38, 174.21 ppm; Anal. calcd. for $C_{34}H_{31}ClF_9N_9O_2S_2$ (868.24); C, 47.03; H, 3.60; N, 14.52. Found: C, 47.08; H, 3.63; N, 14.53; MALDI-TOF/TOF (867.1587) calcd for $C_{34}H_{31}ClF_9N_9O_2S_2$ [M+H]$^+$ (868.1665) found 868.1077.

2-[{4-Amino-6-[4-(4-chlorophenyl)piperazin-1-yl]-1,3,5-triazin-2-yl}methylthio]-N-{1-[3,5-bis(trifluoromethyl)benzyl]imidazolidin-2-ylidene}-4-chloro-5-methylbenzenesulfonamide (**154**).

Starting from *N*-carbamimidoyl-4-(4-chlorophenyl)piperazine-1-carboximidamide hydrochloride (0.508 g, 1.60 mmol). The title compound was obtained after refluxing with ethanol (1:22) in an ultrasonic bath for 10 minutes. Yield 0.384 g (58%); m.p. 219–220 °C; IR (KBr): 3390, 3228 (N-H), 2953, 2923, 2904, 2862 (C-H), 1578, 1531, 1499 (C=N, C=C_{Ar}), 1278, 1166 (SO_2) cm^{-1}; ^{1}H NMR (500 MHz, DMSO-*d*$_6$) δ: 2.31 (s, 3H, CH_3), 2.99-3.16 (m, 4H, piperazine), 3.38 (t, *J=8.60 Hz*, 2H, imidazolidin), 3.47 (t, *J=8.55 Hz*, 2H, imidazolidin), 3.77-3.83 (m, 4H, piperazine), 3.88 (s, 2H, S-CH_2), 4.58 (s, 2H, **C**$\mathbf{H_2}$-C_6H_4-3,5-bis(CF_3)), 6.95-7.90 (m, 7H, H_{Ar} and 2H, NH_2 and 1H, NH, imidazolidin and 1H, H-3), 8.02 (s, 1H, H-6) ppm; ^{13}C NMR (125 MHz, DMSO-d$_6$) δ: 19.38, 41.48, 42.71, 45.23, 46.99, 48.47, 117.75, 121.78, 122.60, 123.27, 124.77, 128.50, 129.11, 130.67, 130.72, 130.98, 132.09, 136.29, 137.03, 139.42, 140.82, 150.17, 158.68, 164.68, 167.39, 174.22 ppm; Anal. calcd. for $C_{33}H_{31}Cl_2F_6N_9O_2S_2$ (834.68); C, 47.49; H, 3.74; N, 15.10. Found: C, 47.46; H, 3.66; N, 14.98; MALDI-TOF/TOF (833.1323) calcd for $C_{33}H_{31}Cl_2F_6N_9O_2S_2$ [M+H]$^+$ (834.1401) found 833.9153.

2-[{4-Amino-6-[4-(3,4-dichlorophenyl)piperazin-1-yl]-1,3,5-triazin-2-yl}methylthio]-N-{1-[3,5-bis(trifluoromethyl)benzyl]imidazolidin-2-ylidene}-4-chloro-5-methylbenzenesulfonamide (**155**).

Starting from *N*-carbamimidoyl-4-(3,4-dichlorophenyl)piperazine-1-carboximidamide hydrochloride (0.563 g, 1.60 mmol). The title compound was obtained after refluxing with ethanol (1:20) in an ultrasonic bath for 10 minutes. Yield 0.381 g (55%); m.p. 220–222 °C; IR (KBr): 3344, 3223 (N-H), 2949, 2922, 2882, 2855 (C-H), 1596, 1556, 1522, 1484 (C=N, C=C_{Ar}), 1277, 1166 (SO_2) cm^{-1}; ^{1}H NMR (500 MHz, DMSO-*d*$_6$) δ: 2.31 (s, 3H, CH_3), 3.16-3.18 (m, 4H, piperazine), 3.38 (t, *J=8.75 Hz*, 2H, imidazolidin), 3.48 (t, *J=8.55 Hz*, 2H, imidazolidin), 3.76-3.82 (m, 4H, piperazine), 3.88 (s, 2H, S-CH_2), 4.58 (s, 2H, **C**$\mathbf{H_2}$-C_6H_3-3,5-bis(CF_3)), 6.94-7.90 (m, 6H, H_{Ar} and 2H, NH_2 and 1H, NH, imidazolidin and 1H, H-3), 8.01 (s, 1H, H-6) ppm; Anal. calcd. for $C_{33}H_{30}Cl_3F_6N_9O_2S_2$ (869.13); C, 46.86; H, 4.26; N, 18.22. Found: C, 45.46; H, 3.41; N, 14.33; MALDI-TOF/TOF (867.0934) calcd for $C_{33}H_{30}Cl_3F_6N_9O_2S_2$ [M+H]$^+$ (868.1012) found 867.9495.

2-{[4-Amino-6-(4-benzylpiperazin-1-yl)-1,3,5-triazin-2-yl]methylthio}-N-{1-[3,5-bis(trifluoromethyl)benzyl]imidazolidin-2-ylidene}-4-chloro-5-methylbenzenesulfonamide (**156**).

Starting from 4-benzyl-*N*-carbamimidoylpiperazine-1-carboximidamide hydrochloride (0.475 g, 1.60 mmol). The title compound was obtained after refluxing with ethanol (1:20) in an ultrasonic bath for 10 minutes. Yield 0.248 g (42%); m.p. 175–176 °C; IR (KBr): 3365, 3322, 3142 (N-H), 2951, 2920, 2889, 2859 (C-H), 1583, 1567, 1519 (C=N, C=C_{Ar}), 1257, 1161 (SO_2) cm^{-1}; ^{1}H NMR (500 MHz, DMSO-*d*$_6$) δ: 2.29-2.31 (m, 3H, CH_3 and 4H, piperazine), 3.37 (t, *J=8.8 Hz*, 2H, imidazolidin), 3.45-3.49 (m, 2H, imidazolidin and 2H, Ph-**C**$\mathbf{H_2}$), 3.64-3.66 (m, 4H, piperazine), 3.85 (s, 2H, S-CH_2), 4.55 (s, 2H, **C**$\mathbf{H_2}$-C_6H_3-3,5-bis(CF_3)), 6.89-6.92 (m, 2H, NH_2), 7.24-7.34 (m, 5H, CH_2-$C_6\mathbf{H_5}$), 7.53-7.55 (m, 1H, NH, imidazolidin), 7.83 (s, 1H, H-3), 7-88-7.89 (m, 3H, CH_2-$C_6\mathbf{H_3}$-3,5-bis(CF_3)), 8.01 (s, 1H, H-6) ppm; Anal. calcd. for $C_{34}H_{34}ClF_6N_9O_2S_2$ (814.27); C, 50.15; H, 4.21; N, 15.48. Found: C, 50.01; H, 4.09; N, 15.24; MALDI-TOF/TOF (813.1870) calcd for $C_{34}H_{34}ClF_6N_9O_2S_2$ [M+H]$^+$ (814.1948) found 814.1517.

2-{[4-Amino-6-(4-benzylpiperidin-1-yl)-1,3,5-triazin-2-yl]methylthio}-N-{1-[3,5-bis(trifluoromethyl)benzyl]imidazolidin-2-ylidene}-4-chloro-5-methylbenzenesulfonamide (**157**).

Starting from 4-benzyl-*N*-carbamimidoylpiperidine-1-carboximidamide hydrochlorid (0.473 g, 1.60 mmol). The title compound was obtained after refluxing with ethanol (1:20) in an ultrasonic bath for 10 minutes. Yield 0.189 g (42%); m.p. 170–171 °C; IR (KBr): 3388, 3319, 3203 (N-H), 2969, 2938, 2920, 2846 (C-H), 1581, 1565, 1537, 1518 (C=N, $C=C_{Ar}$), 1255, 1160 (SO_2) cm^{-1}; ^1H NMR (500 MHz, DMSO-d_6) δ: 0.93-0.96 (m, 2H, piperidine C3 and C5), 1.50-1.54 (m, 2H, piperidine C3 and C5), 1.72-1.75 (m, 1H, piperidine C4), 2.29 (s, 3H, CH_3), 2.46 (d, *J=3.65 Hz*, 2H, piperidine-**C**$\mathbf{H_2}$Ph), 2.65-2.69 (m, 2H, piperidine C2 and C6), 3.37 (t, *J=8.8 Hz*, 2H, imidazolidin), 3.47 (t, *J=8.75 Hz*, 2H, imidazolidin), 3.85 (s, 2H, S-CH_2), 4.51-4.58 (m, 2H, **C**$\mathbf{H_2}$-C_6H_3-3,5-bis(CF_3) and 2H, piperidine C2 and C6), 6.85-7.89 (m, 8H, H_{Ar} and 1H, NH, imidazolidin and 2H, NH_2 and 1H, H-3), 8.01 (s, 1H, H-6) ppm; Anal. calcd. for $C_{35}H_{35}ClF_6N_8O_2S_2$ (813.28); C, 51.69; H, 4.34; N, 13.78. Found: C, 51.30; H, 4.24; N, 13.54; MALDI-TOF/TOF (812.1917) calcd for $C_{35}H_{35}ClF_6N_8O_2S_2$ $[M+H]^+$ (813.1995) found 812.9682.

S.3.2.4. General procedure for the preparation of 6-Substituted 2-[(4-Amino-1,3,5-triazin-2-yl)methylthio]-*N*-(tetrahydropyrimidin-2(1*H)*-ylidene)-4-chloro-5-methylbenzenesulfonamide **158-162**

To the solution of sodium methoxide prepared from sodium (0.0368 g, 1.60 mmol) and anhydrous methanol (7.5 mL), ethyl 2-{5-chloro-4-methyl-2-[*N*-{1-[4-(trifluoromethyl)benzyl]tetrahydropyrimidin-2(*1H*)-ylidene}sulfamoyl]phenylthio}acetate **19** (0.80 mmol) and the next appropriate biguanide hydrochloride (1.60 mmol) was added. The reaction mixture was stirred under reflux for 45 h. After cooling the precipitate was filtered off and dried, then stirred vigorously with water (25 mL) for 25 min. The crude product was purified by crystallization from the ethanol or by refluxing with ethanol in an ultrasonic bath for 10 minutes.

2-{[4-Amino-6-(3,4-dihydroquinolin-1(2H)-yl)-1,3,5-triazin-2-yl]methylthio}-4-chloro-5-methyl-N-{1-[4-(trifluoromethyl)benzyl]tetrahydropyrimidin-2(1H)-ylidene}benzenesulfonamide (**158**).

Starting from *N*-carbamimidoyl-3,4-dihydroquinoline-1(*2H*)-carboximidamide hydrochloride (0.406 g, 1.60 mmol). The title compound was obtained after refluxing with ethanol (1:20) in an ultrasonic bath for 10 minutes. Yield 0.133 g (34%); m.p. 181–182 °C; IR (KBr): 3356, 3232 (N-H), 2945, 2932, 2879, 2866 (C-H), 1581, 1557, 1525 (C=N, $C=C_{Ar}$), 1281, 1127 (SO_2) cm^{-1}; ^1H NMR (500 MHz, DMSO-d_6) δ: 1.74-1.78 (m, 2H, hexahydropyrimidine), 1.80-1.84 (m, 2H, 1,2,3,4-tetrahydroquinoline), 2.29 (s, 3H, CH_3), 2.70 (t, *J=6.55 Hz*, 2H, 1,2,3,4-tetrahydroquinoline), 3.18 (t, *J=5.6 Hz*, 2H, hexahydropyrimidine), 3.23-3.26 (m, 2H, hexahydropyrimidine), 3.88-3.90 (m, 2H, 1,2,3,4-tetrahydroquinoline), 3.87-3.92 (m, 2H, S-CH_2), 4.60 (s, 2H, **C**$\mathbf{H_2}$-C_6H_4-4-CF_3), 6.95-7.68 (m, 8H, H_{Ar} and 2H, NH_2 and 1H, NH, hexahydropyrimidine), 7.70 (s, 1H, H-3), 7.79 (s, 1H, H-6) ppm; Anal. calcd. for $C_{32}H_{32}ClF_3N_8O_2S_2$ (717.17); C, 53.59; H, 4.50; N, 15.62. Found: C, 53.80; H, 4.44; N, 15.49; MALDI-TOF/TOF (716.1730) calcd for $C_{32}H_{32}ClF_3N_8O_2S_2$ $[M+H]^+$ (717.1808) found 717.1829.

2-{[4-Amino-6-(4-phenylpiperazin-1-yl)-1,3,5-triazin-2-yl]methylthio}-4-chloro-5-methyl-N-{1-[4-(trifluoromethyl)benzyl]tetrahydropyrimidin-2(1H)-ylidene}benzenesulfonamide (**159**).

Starting from *N*-carbamimidoyl-4-phenylpiperazine-1-carboximidamide hydrochloride (0.452 g, 1.60 mmol). The title compound was obtained by crystallization from ethanol (1:20). Yield 0.325 g (54%); m.p. 213–214 °C; IR (KBr): 3337, 3200, 3110 (N-H), 2972, 2926, 2912, 2858 (C-H), 1577, 1540, 1518, 1505 (C=N, $C=C_{Ar}$), 1270, 1168 (SO_2) cm^{-1}; ^1H NMR (500 MHz, DMSO-d_6) δ: 1.78 (quintet, *J=5.6 Hz*, 2H, hexahydropyrimidine), 2.29 (s, 3H, CH_3), 3.07-3.10 (m, 4H, piperazine), 3.21 (t, *J=5.6 Hz*, 2H, hexahydropyrimidine), 3.43-3.47 (m, 2H, hexahydropyrimidine), 3.80-3.84 (m, 4H, piperazine), 3.87 (s, 2H, S-CH_2), 4.64 (s, 2H, **C**$\mathbf{H_2}$-C_6H_4-4-CF_3), 6.80-7.59 (m, 9H, H_{Ar} and 2H, NH_2 and 1H, hexahydropyrimidine), 7.78 (s, 1H, H-3), 7.85 (s, 1H, H-6) ppm; Anal. calcd. for $C_{33}H_{35}ClF_3N_9O_2S_2$ (746.27); C, 53.11; H, 4.73; N, 16.89. Found: C, 52.98; H, 4.67; N, 16.63; MALDI-TOF/TOF (745.1996) calcd for $C_{33}H_{35}ClF_3N_9O_2S_2$ $[M+H]^+$ (746.2074) found 746.0085.

2-[{4-Amino-6-[4-(3-(trifluoromethyl)phenyl]piperazin-1-yl}-1,3,5-triazin-2-yl]methylthio}-4-chloro-5-methyl-N-{1-[4-(trifluoromethyl)benzyl]tetrahydropyrimidin-2(1H)-ylidene}benzenesulfonamide (**160**).

Starting from *N*-carbamimidoyl-4-[3-(trifluoromethyl)phenyl]piperazine-1-carboximidamide hydrochloride (0.561 g, 1.60 mmol). The title compound was obtained after refluxing with ethanol (1:20) in an ultrasonic bath for 10 minutes. Yield 0.335 g (52%); m.p. 192–193 °C; IR (KBr): 3337, 3305, 3192 (N-H), 2975, 2956, 2929, 2884 (C-H), 1576, 1562, 1539, 1518 (C=N, $C=C_{Ar}$), 1272, 1148 (SO_2) cm^{-1}; ^1H NMR (500 MHz, DMSO-d_6) δ: 1.78 (quintet, *J=5.7 Hz*, 2H, hexahydropyrimidine), 2.29 (s, 3H, CH_3), 3.20-3.25 (m, 4H, hexahydropyrimidine), 3.24-3.28 (m, 4H, piperazine), 3.80-3.85 (m, 4H, piperazine), 3.87 (s, 2H, S-CH_2), 4.64 (s, 2H, C**H**$_2$-C_6H_4-4-CF_3), 7.02-7.58 (m, 8H, H_{Ar} and 2H, NH_2 and 1H, NH, hexahydropyrimidine), 7.78 (s, 1H, H-3), 7.85 (s, 1H, H-6) ppm; Anal. calcd. for $C_{34}H_{34}ClF_6N_9O_2S_2$ (814.27); C, 50.15; H, 4.21; N, 15.48. Found: C, 50.13; H, 4.15; N, 15.16.

2-[{4-Amino-6-[4-(4-chlorophenyl)piperazin-1-yl]-1,3,5-triazin-2-yl}methylthio]-4-chloro-5-methyl-N-{1-[4-(trifluoromethyl)benzyl]tetrahydropyrimidin-2(1H)-ylidene}benzenesulfonamide (**161**).

Starting from *N*-carbamimidoyl-4-(4-chlorophenyl)piperazine-1-carboximidamide hydrochloride (0.508 g, 1.60 mmol). Starting from *N*-carbamimidoyl-4-(4-chlorophenyl)piperazine-1-carboximidamide hydrochloride (0.508 g, 1.60 mmol). The title compound was obtained after refluxing with ethanol (1:20) in an ultrasonic bath for 10 minutes. Yield 0.320 g (51%); m.p. 207–208 °C; IR (KBr): 3343, 3222 (N-H), 2955, 2944, 2920, 2860 (C-H), 1595, 1557, 1520, 1495 (C=N, $C=C_{Ar}$), 1267, 1160 (SO_2) cm^{-1}; ^1H NMR (500 MHz, DMSO-d_6) δ: 1.78 (quintet, *J=5.6 Hz*, 2H, hexahydropyrimidine), 2.28 (s, 3H, CH_3), 3.08-3.12 (m, 4H, piperazine), 3.21 (t, *J=5.8 Hz*, 2H, hexahydropyrimidine), 3.24-3.29 (m, 2H, hexahydropyrimidine), 3.79-3.85 (m, 4H, piperazine), 3.87 (s, 2H, S-CH_2), 4.64 (s, 2H, C**H**$_2$-C_6H_4-4-CF_3), 6.96-7.58 (m, 8H, H_{Ar} and 2H, NH_2 and 1H, hexahydropyrimidine), 7.78 (s, 1H, H-3), 7.85 (s, 1H, H-6) ppm; Anal. calcd. for $C_{33}H_{34}Cl_2F_3N_9O_2S_2$ (780.71); C, 50.77; H, 4.39; N, 16.15. Found: C, 50.75; H, 4.40; N, 16.15.

2-{[4-Amino-6-(4-benzylpiperazin-1-yl)-1,3,5-triazin-2-yl]methylthio}-4-chloro-5-methyl-N-{1-[4-(trifluoromethyl)benzyl]tetrahydropyrimidin-2(1H)-ylidene}benzenesulfonamide (**162**).

Starting from 4-benzyl-*N*-carbamimidoylpiperazine-1-carboximidamide hydrochloride (0.475 g, 1.60 mmol). The title compound was obtained after refluxing with ethanol (1:17) in an ultrasonic bath for 10 minutes. Yield 0.272 g (65%); m.p. 217–218 °C; IR (KBr): 3387, 3323, 3226 (N-H), 2949, 2937, 2919, 2880 (C-H), 1585, 1557, 1515 (C=N, $C=C_{Ar}$), 1276, 1159 (SO_2) cm^{-1}; ^1H NMR (500 MHz, DMSO-d_6) δ: 1.78 (quintet, *J=5.70 Hz*, 2H, hexahydropyrimidine), 2.27 (s, 3H, CH_3), 2.28-2.34 (m, 4H, piperazine), 3.20 (t, *J=5.65 Hz*, 2H, hexahydropyrimidine), 3.25-3.28 (m, 2H, hexahydropyrimidine), 3.46 (s, 2H, CH_2Ph), 3.64-3.69 (m, 4H, piperazine), 3.84 (s, 2H, S-CH_2), 4.62 (s, 2H, C**H**$_2$-C_6H_4-4-CF_3), 6.92-7.58 (m, 9H, H_{Ar} and 2H, NH_2 and 1H, hexahydropyrimidine), 7.77 (s, 1H, H-3), 7.83 (s, 1H, H-6) ppm; Anal. calcd. for $C_{34}H_{37}ClF_3N_9O_2S_2$ (760.29); C, 53.71; H, 4.91; N, 16.58. Found: C, 53.65; H, 4.86; N, 16.48; MALDI-TOF/TOF (759.2152) calcd for $C_{34}H_{37}ClF_3N_9O_2S_2$ $[M+H]^+$ (760.2230) found 760.0721.

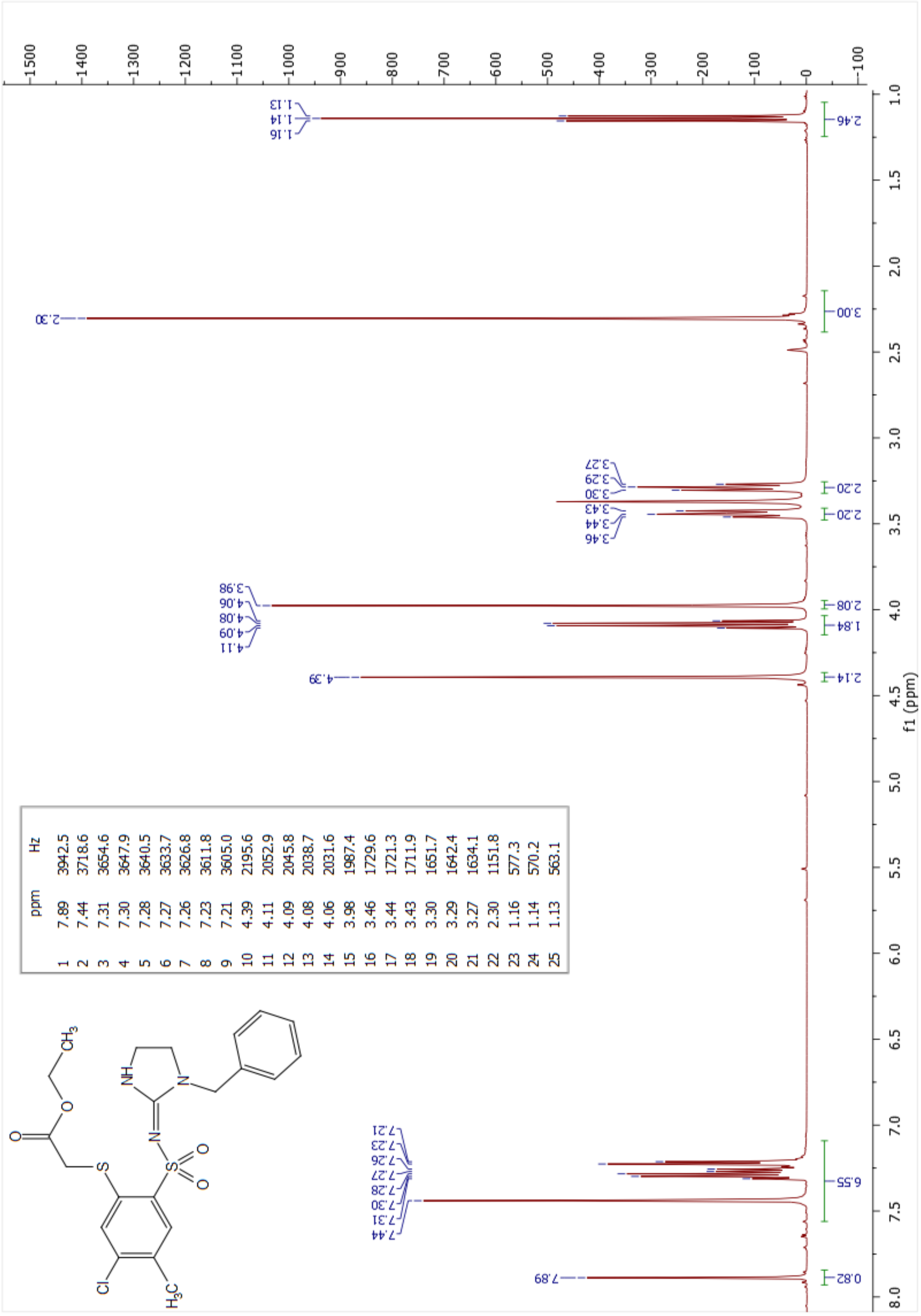

Spectrum 1 . ^{1}H NMR of compound **12** (500 MHz, DMSO-d_6)

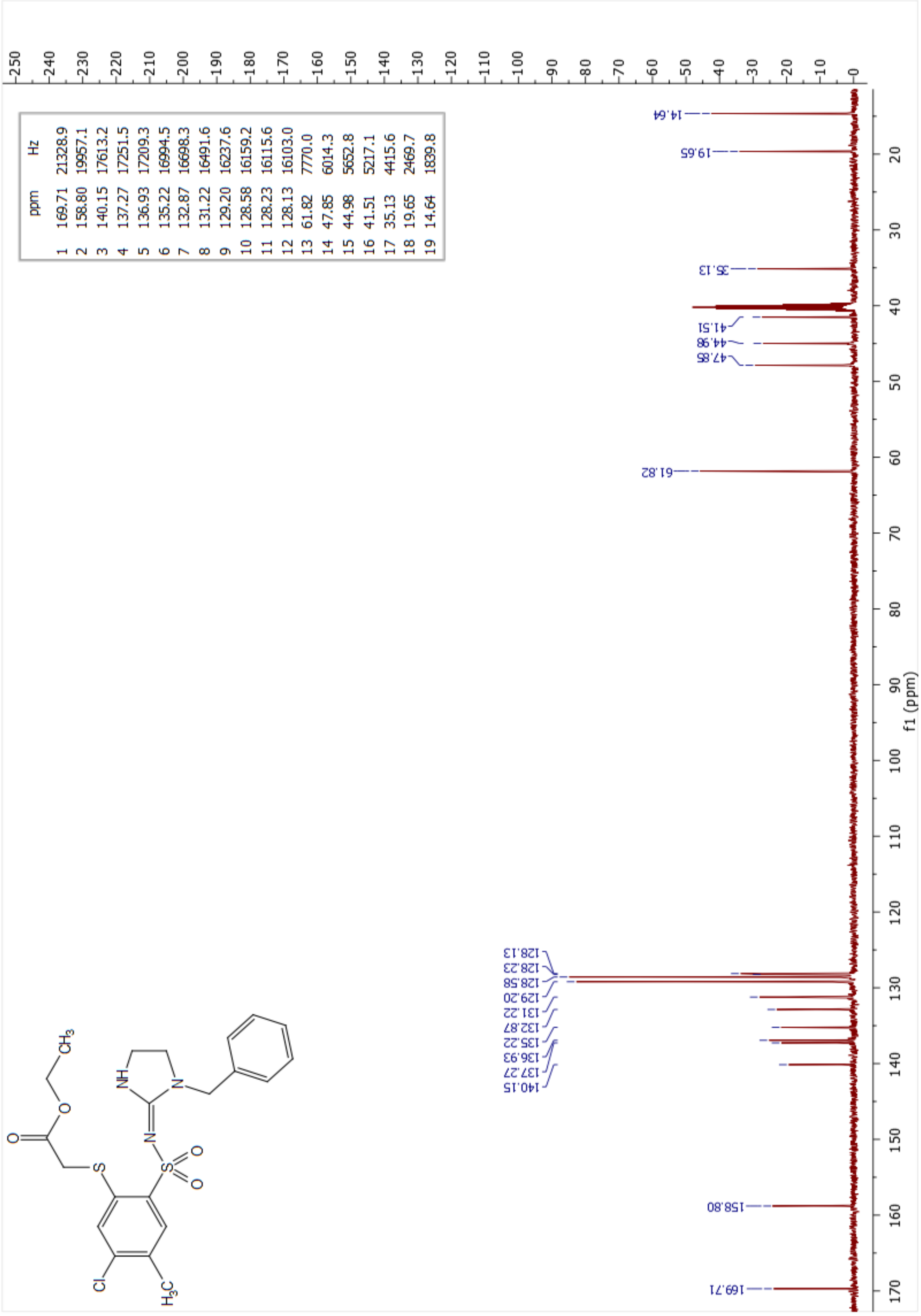

Spectrum 2. ^{13}C NMR of compound **12** (125 MHz, DMSO-d_6)

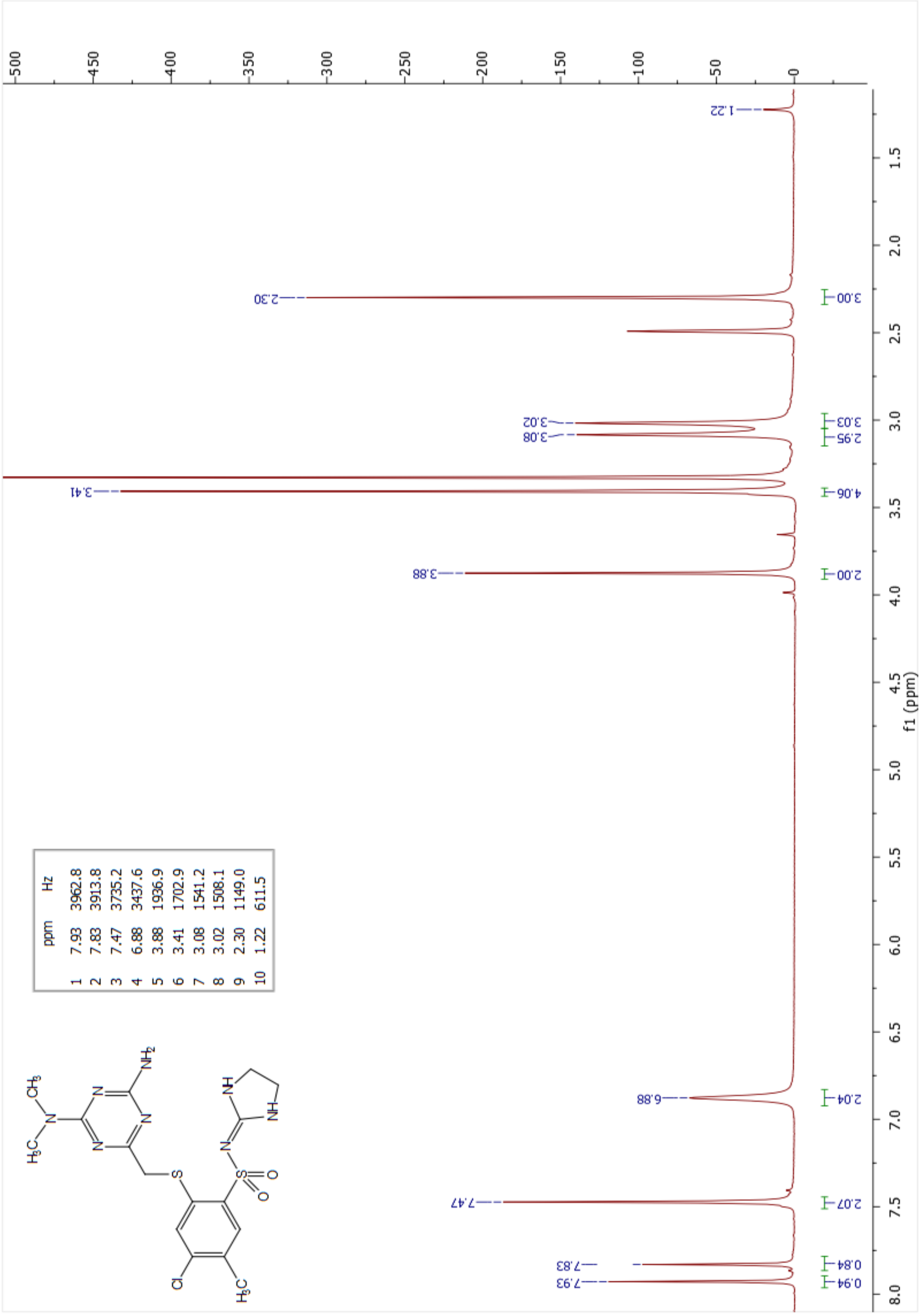

Spectrum 3. ^{1}H NMR of compound **20** (500 MHz, DMSO-d$_6$)

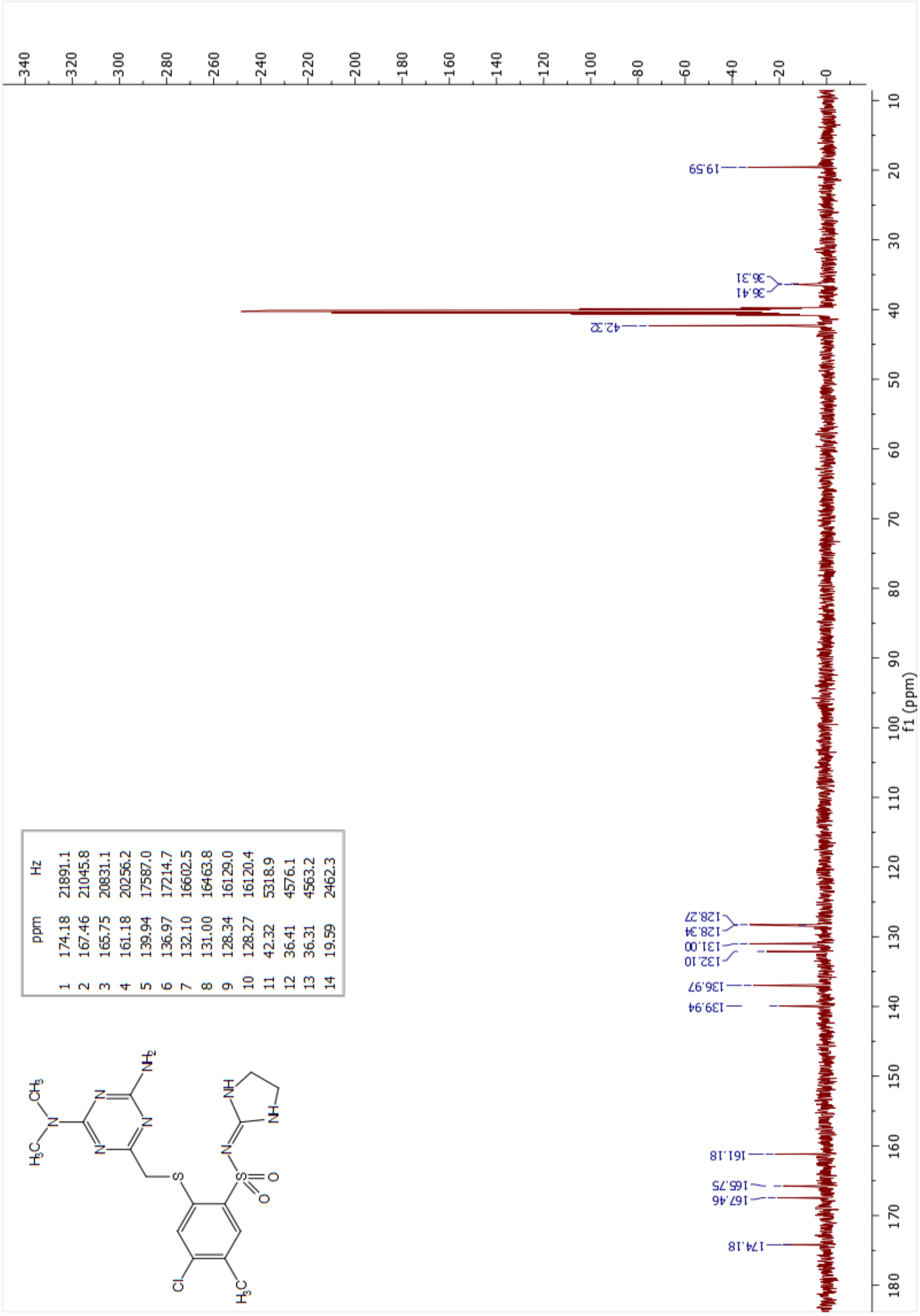

Spectrum 4. ^{13}C NMR of compound **20** (125 MHz, DMSO-d_6)

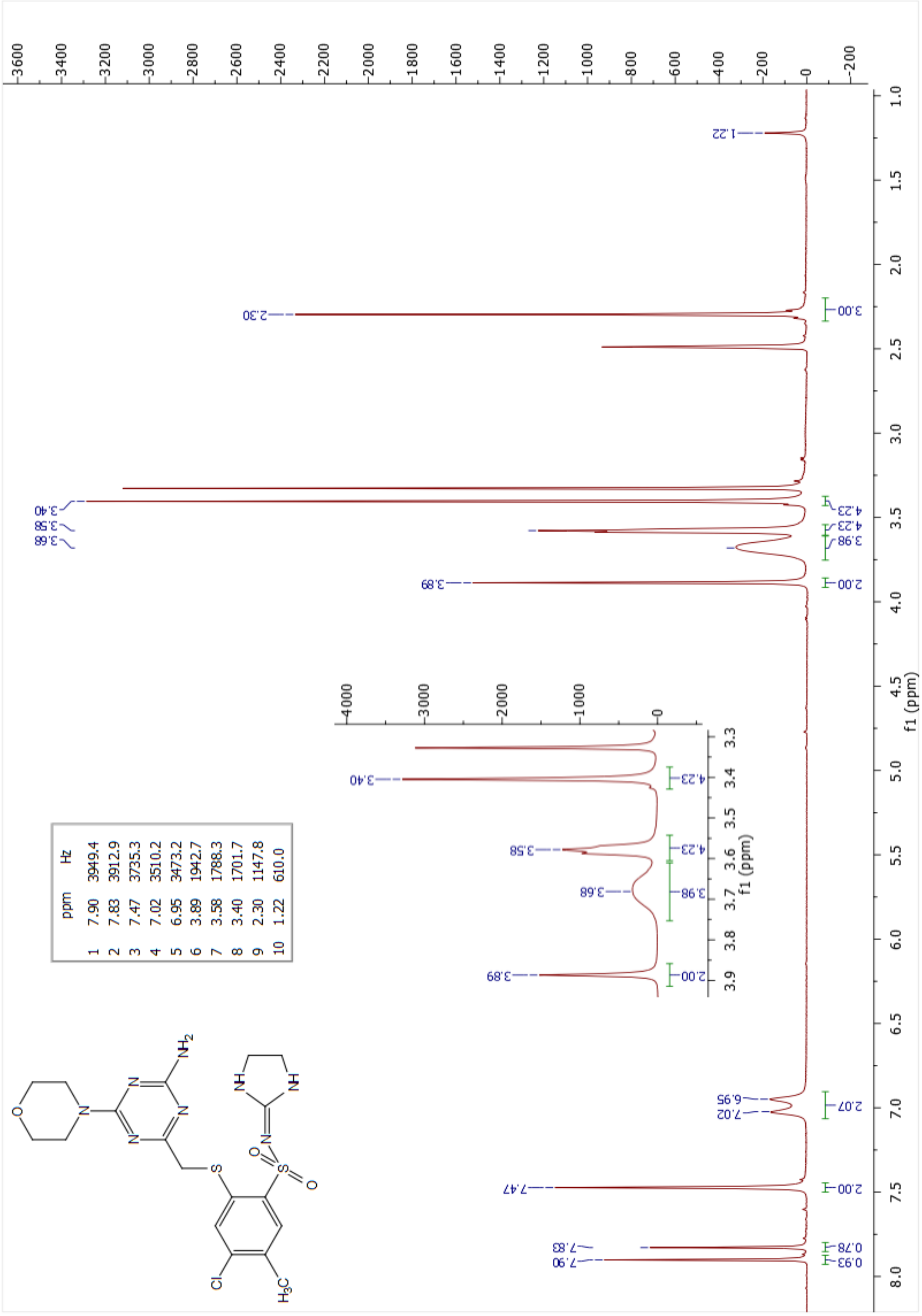

Spectrum 5. ^{1}H NMR of compound **21** (500 MHz, DMSO-d_6)

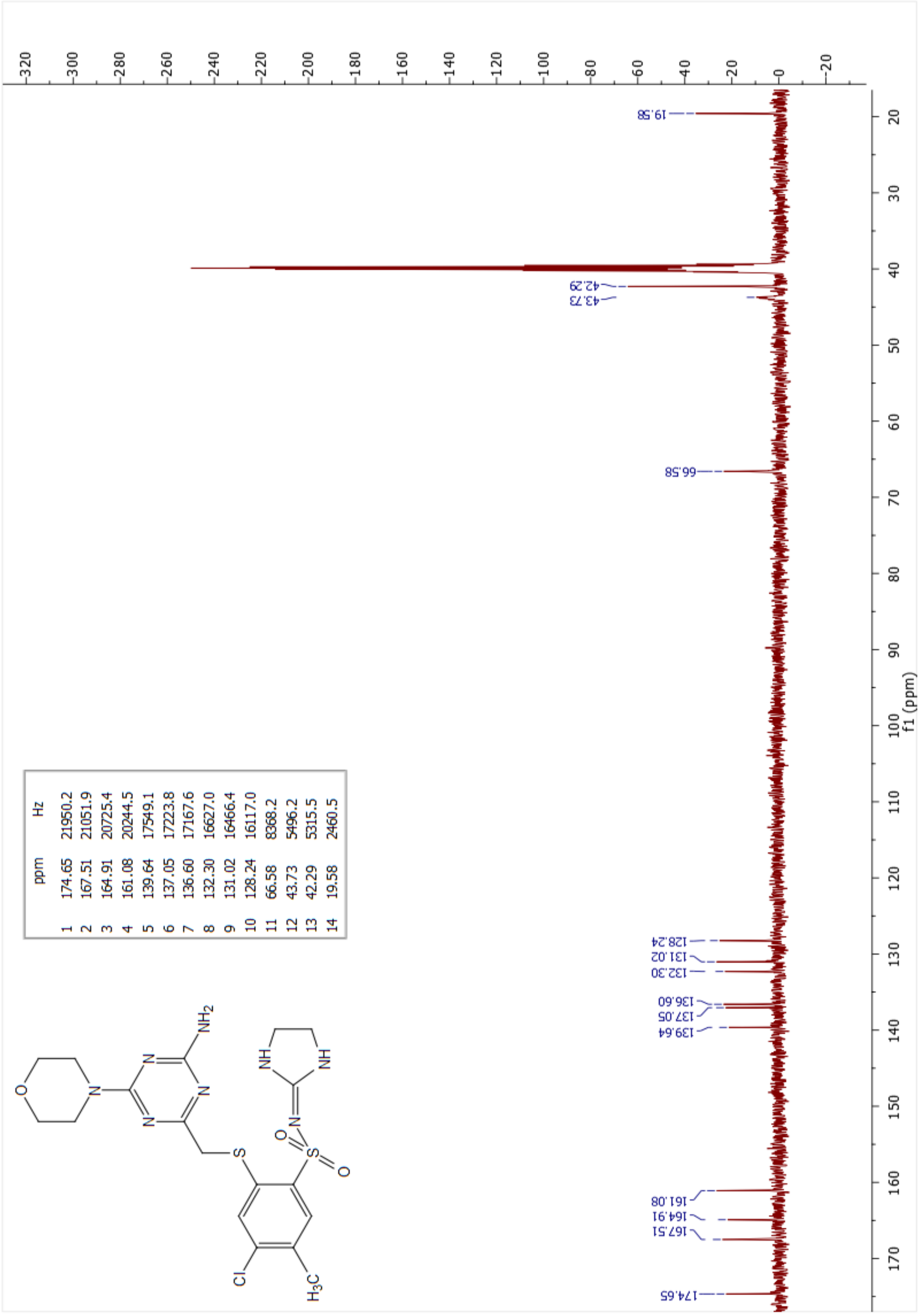

Spectrum 6. ^{13}C NMR of compound **21** (125 MHz, DMSO-d_6)

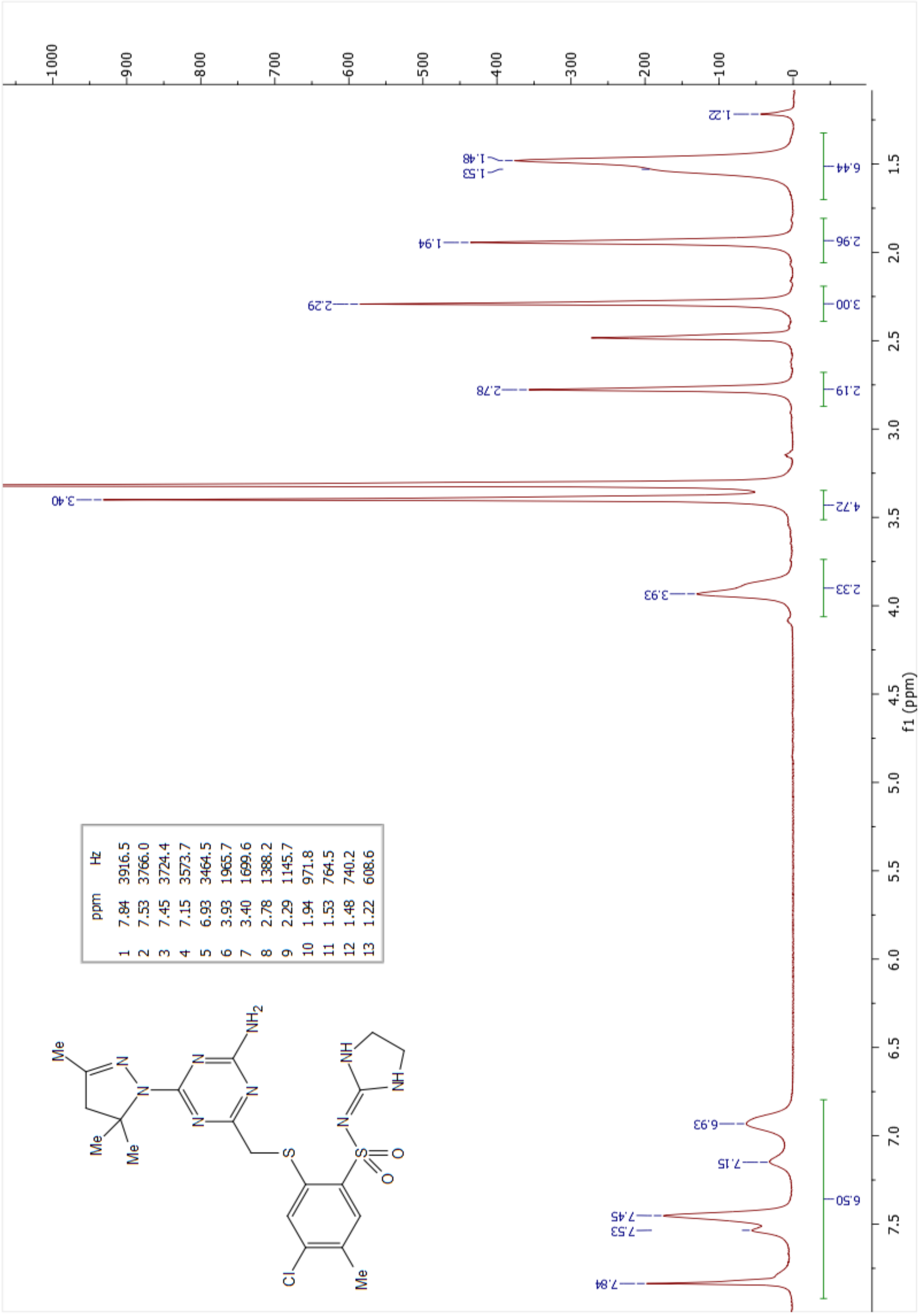

Spectrum 7. ^{1}H NMR of compound **22** (500 MHz, DMSO-d6)

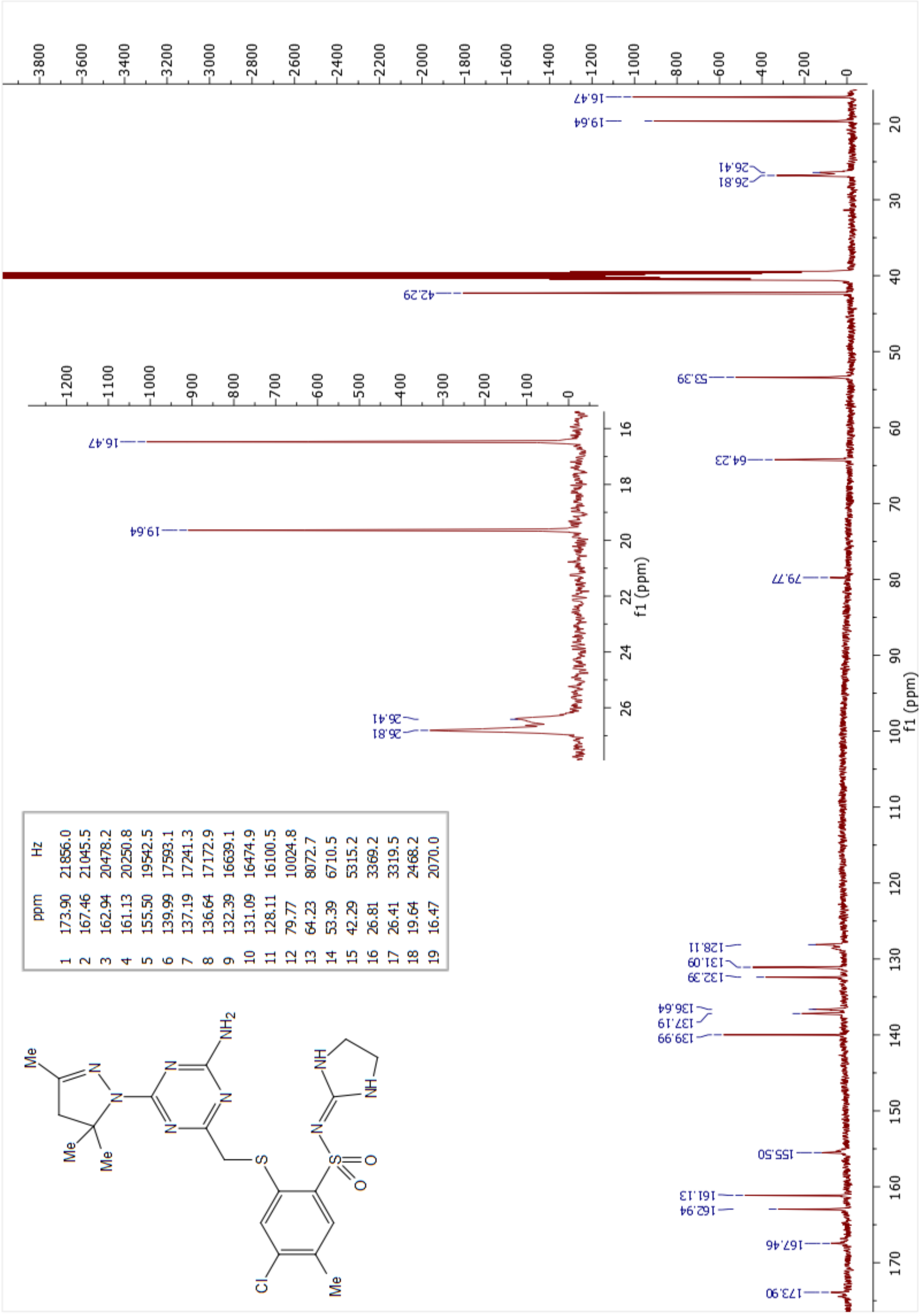

Spectrum 8. ^{13}C NMR of compound **22** (125 MHz, DMSO-d$_6$)

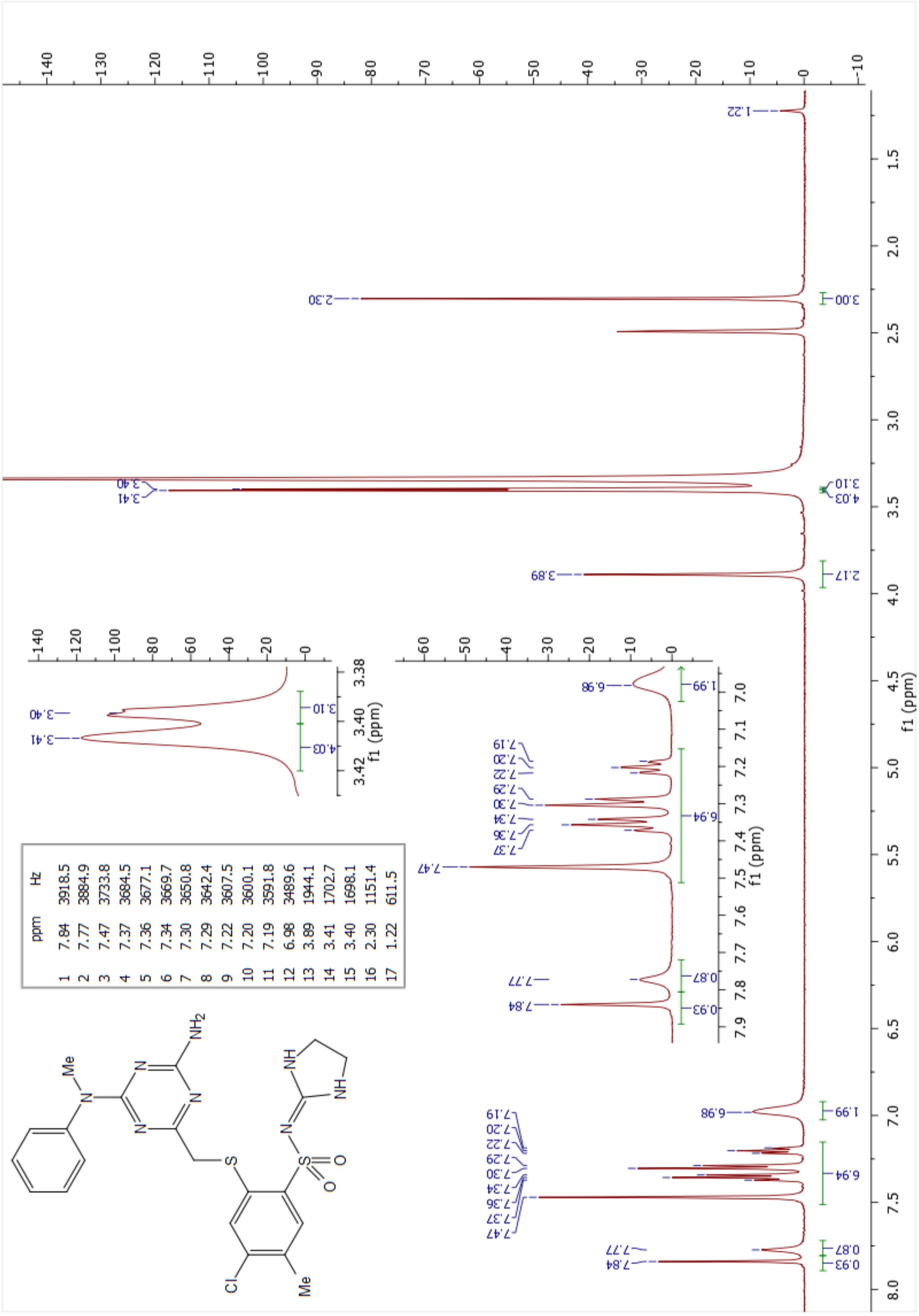

Spectrum 9. ^{1}H NMR of compound **28** (500 MHz, DMSO-d$_6$)

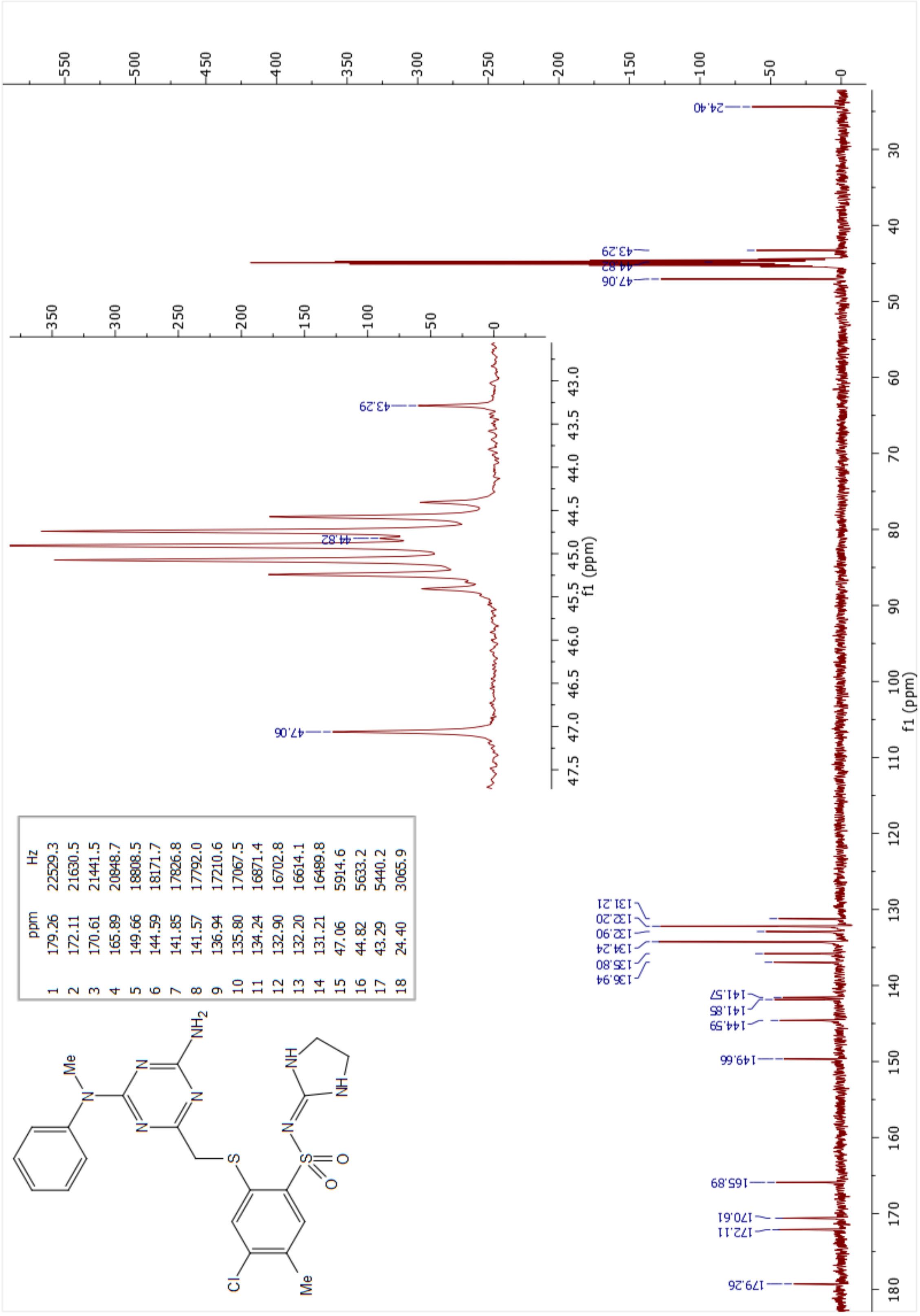

Spectrum 10. ^{13}C NMR of compound **28** (125 MHz, DMSO-d_6)

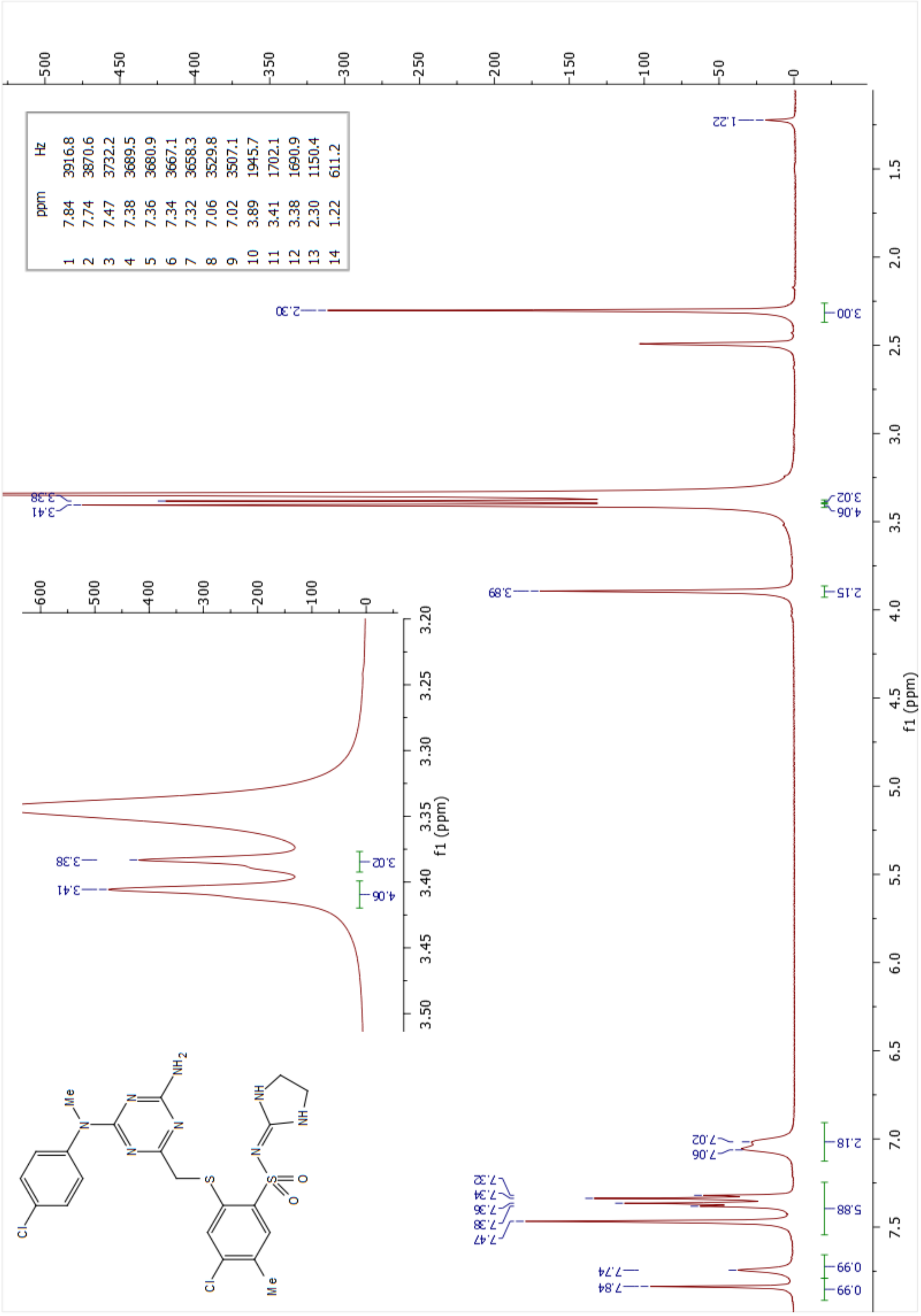

Spectrum 11. ^{1}H NMR of compound **29** (500 MHz, DMSO-d_6)

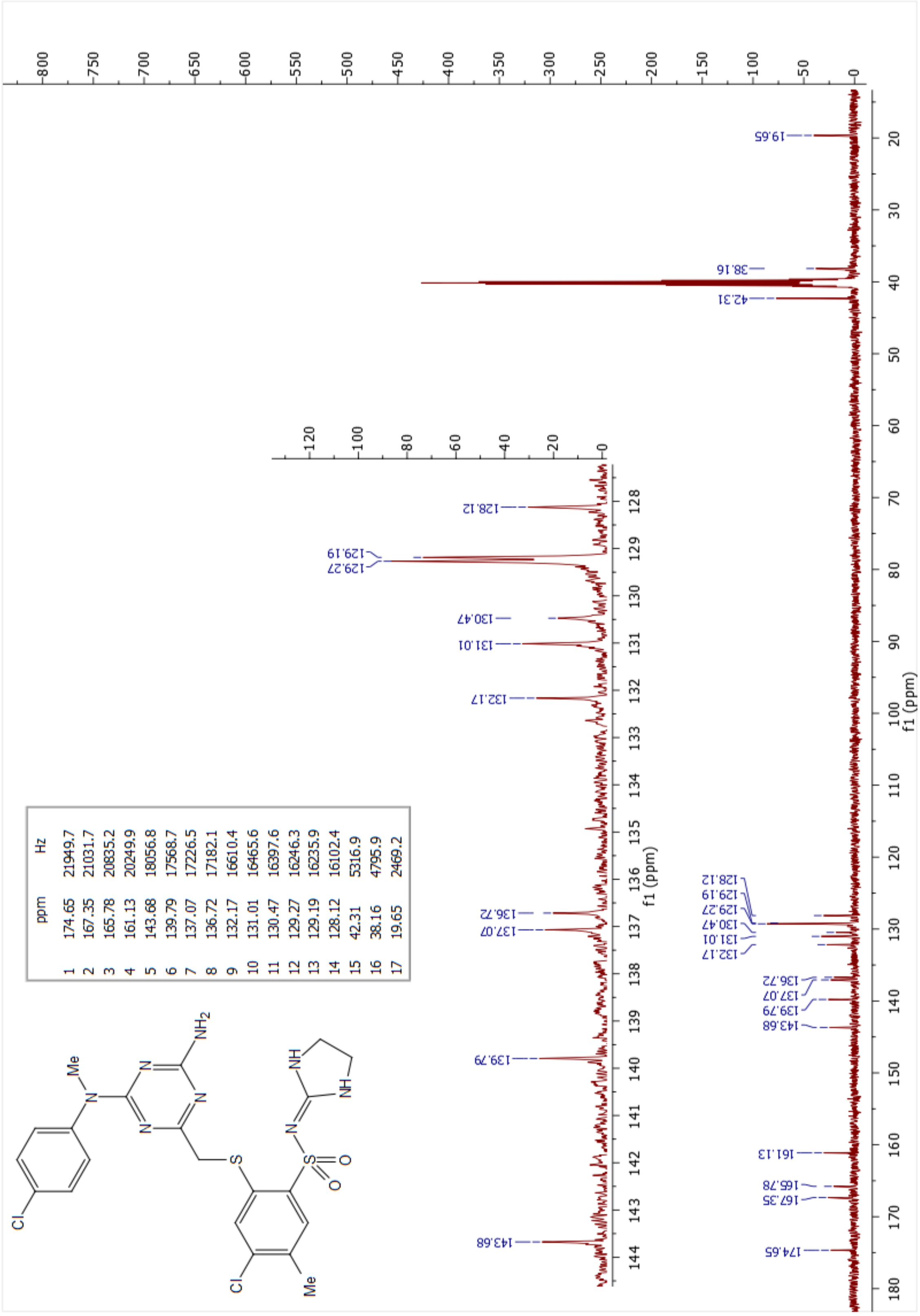

Spectrum 12. ^{13}C NMR of compound **29** (125 MHz, DMSO-d$_6$)

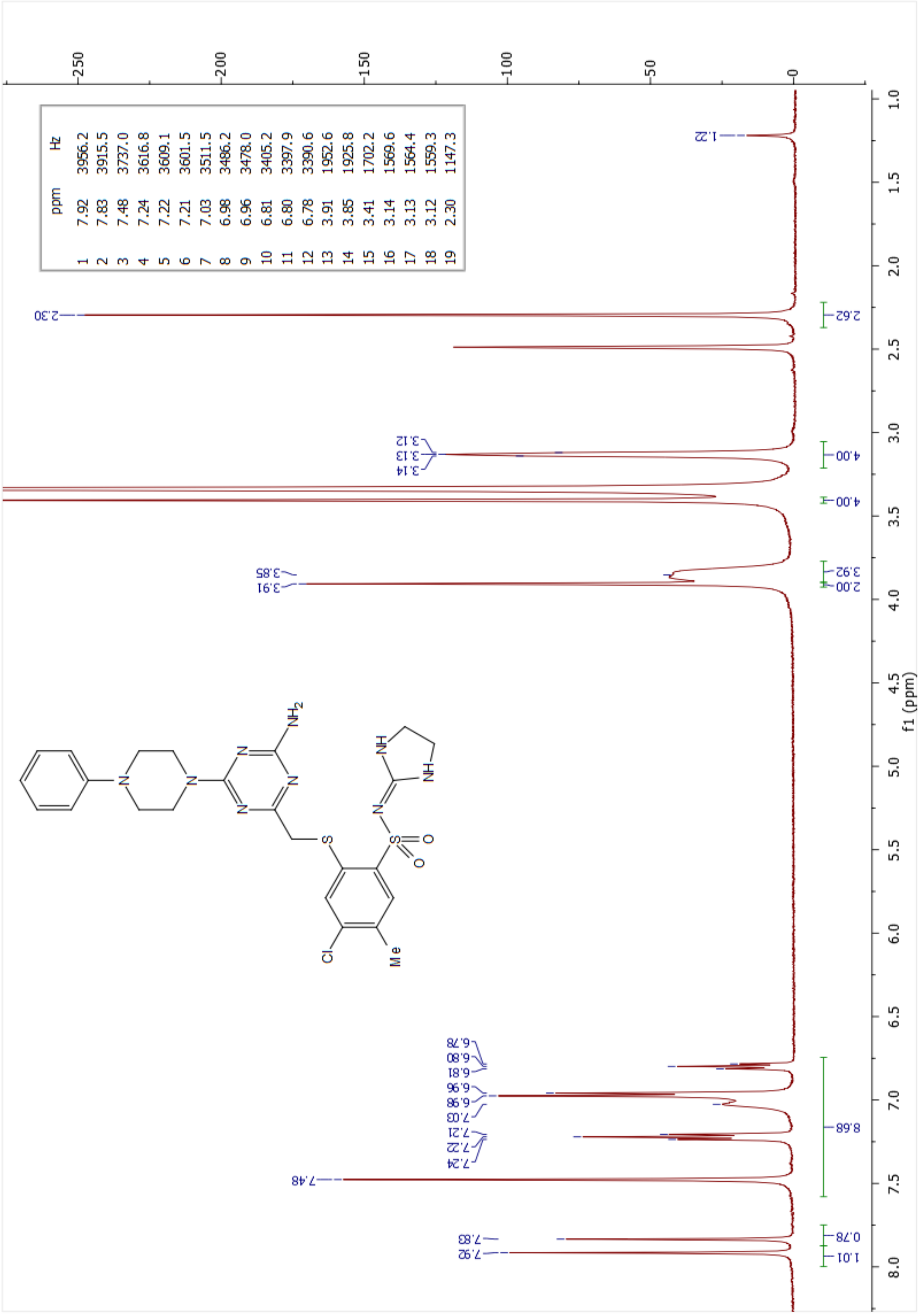

Spectrum 13. ^{1}H NMR of compound **31** (500 MHz, DMSO-d_6)

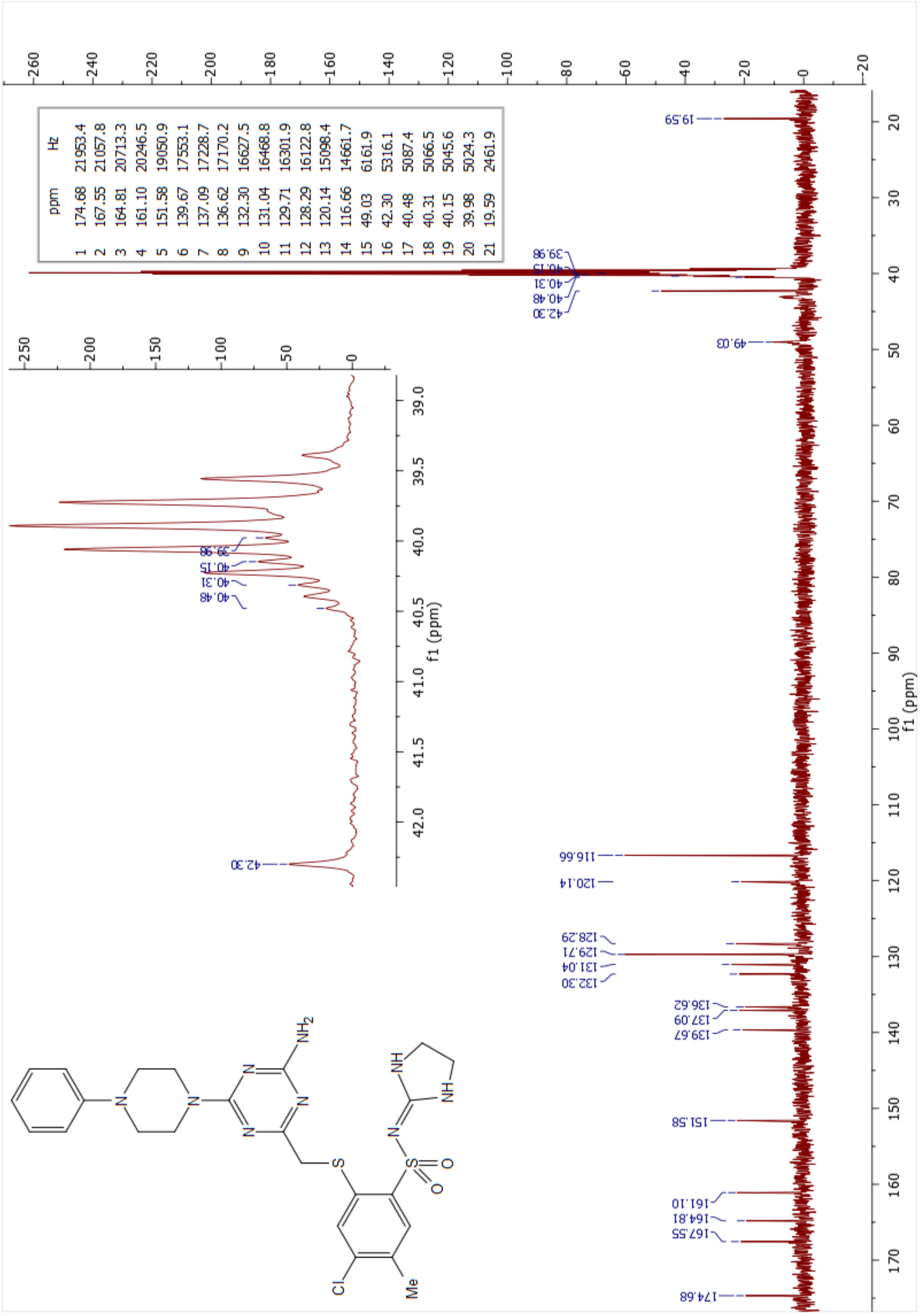

Spectrum 14. ^{13}C NMR of compound **31** (125 MHz, DMSO-d_6)

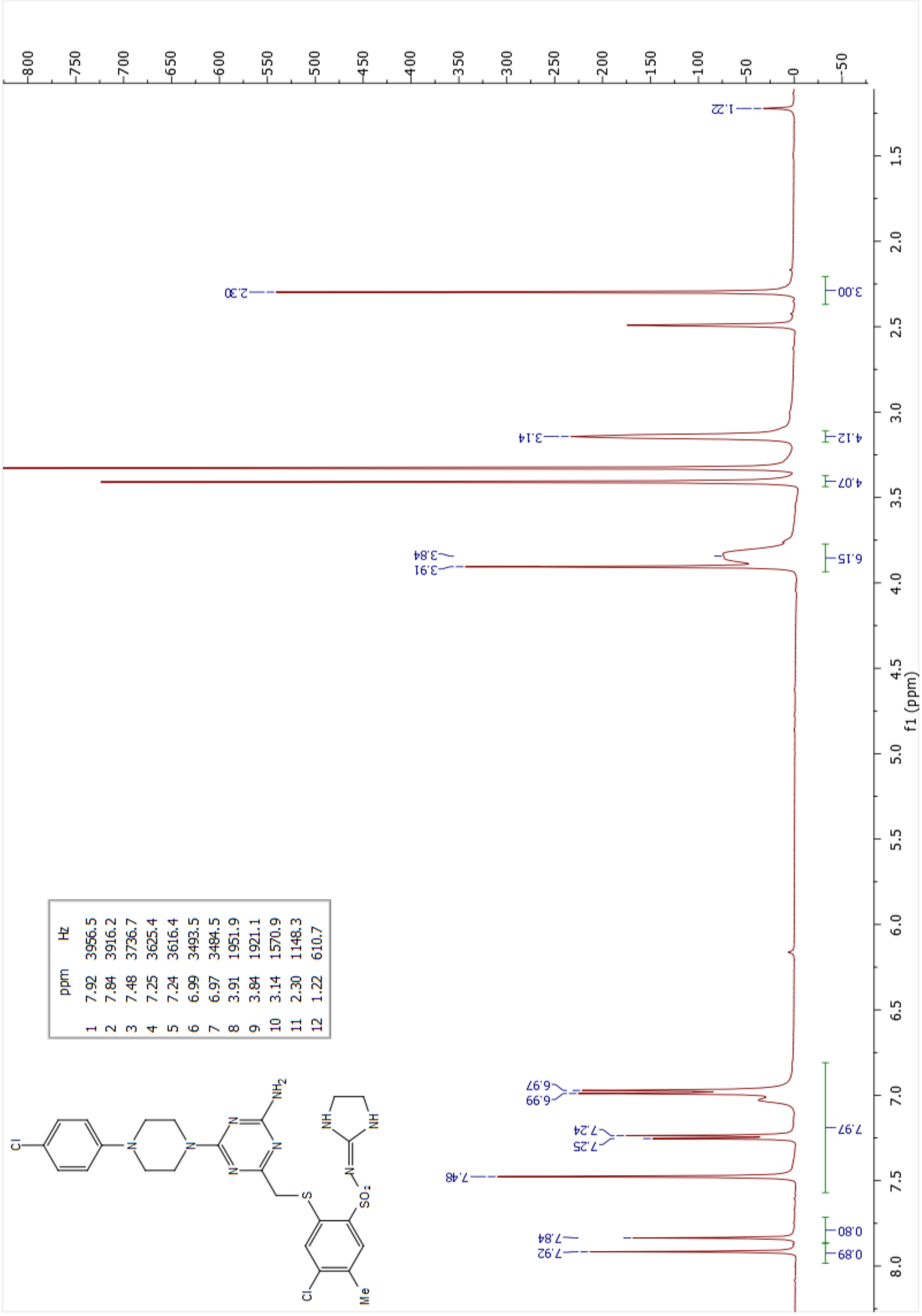

Spectrum 15. ^{1}H NMR of compound **36** (500 MHz, DMSO-d$_6$)

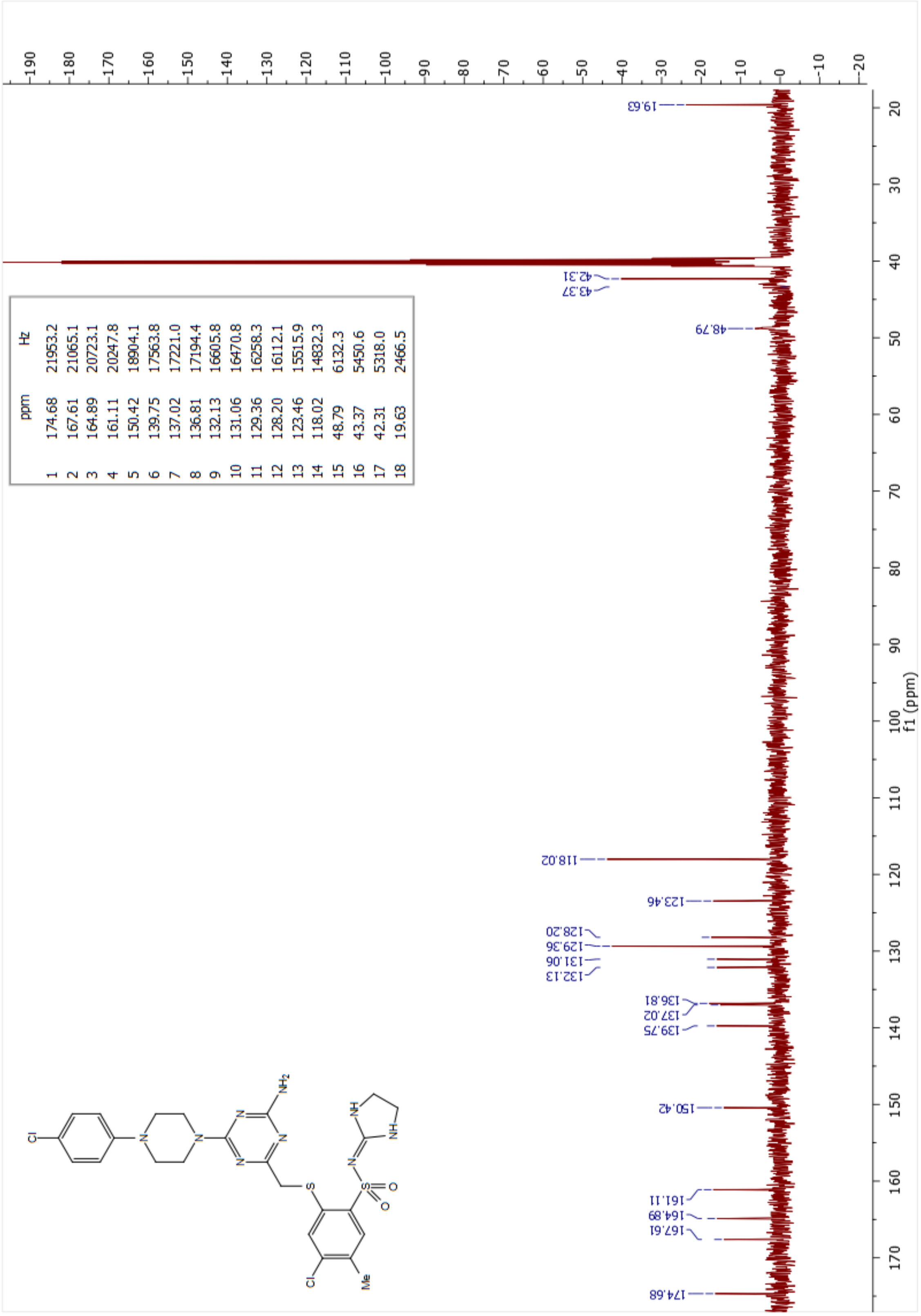

Spectrum 16. ^{13}C NMR of compound **36** (125 MHz, DMSO-d$_6$)

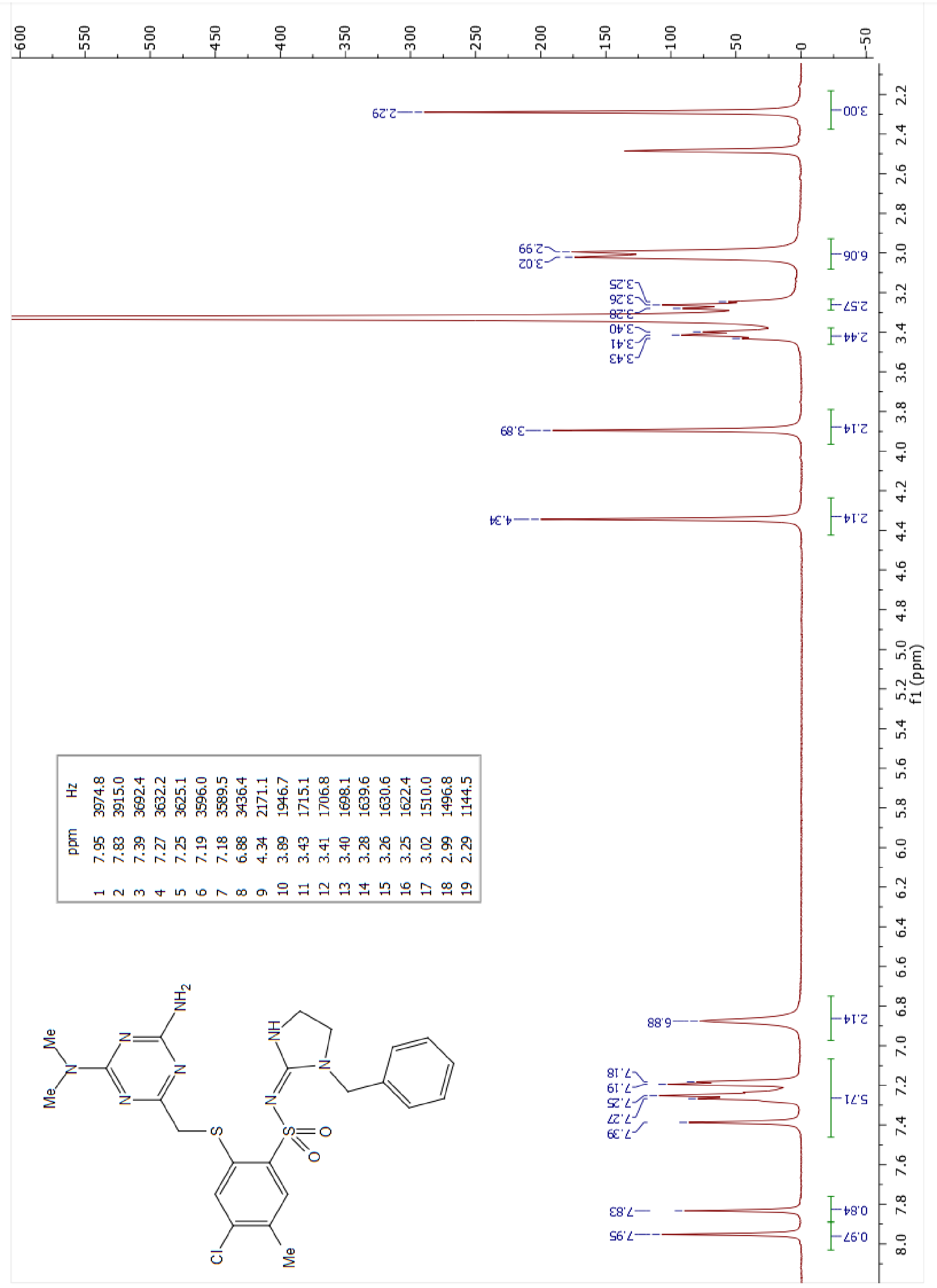

Spectrum 17. ^{1}H NMR of compound **45** (500 MHz, DMSO-d_6)

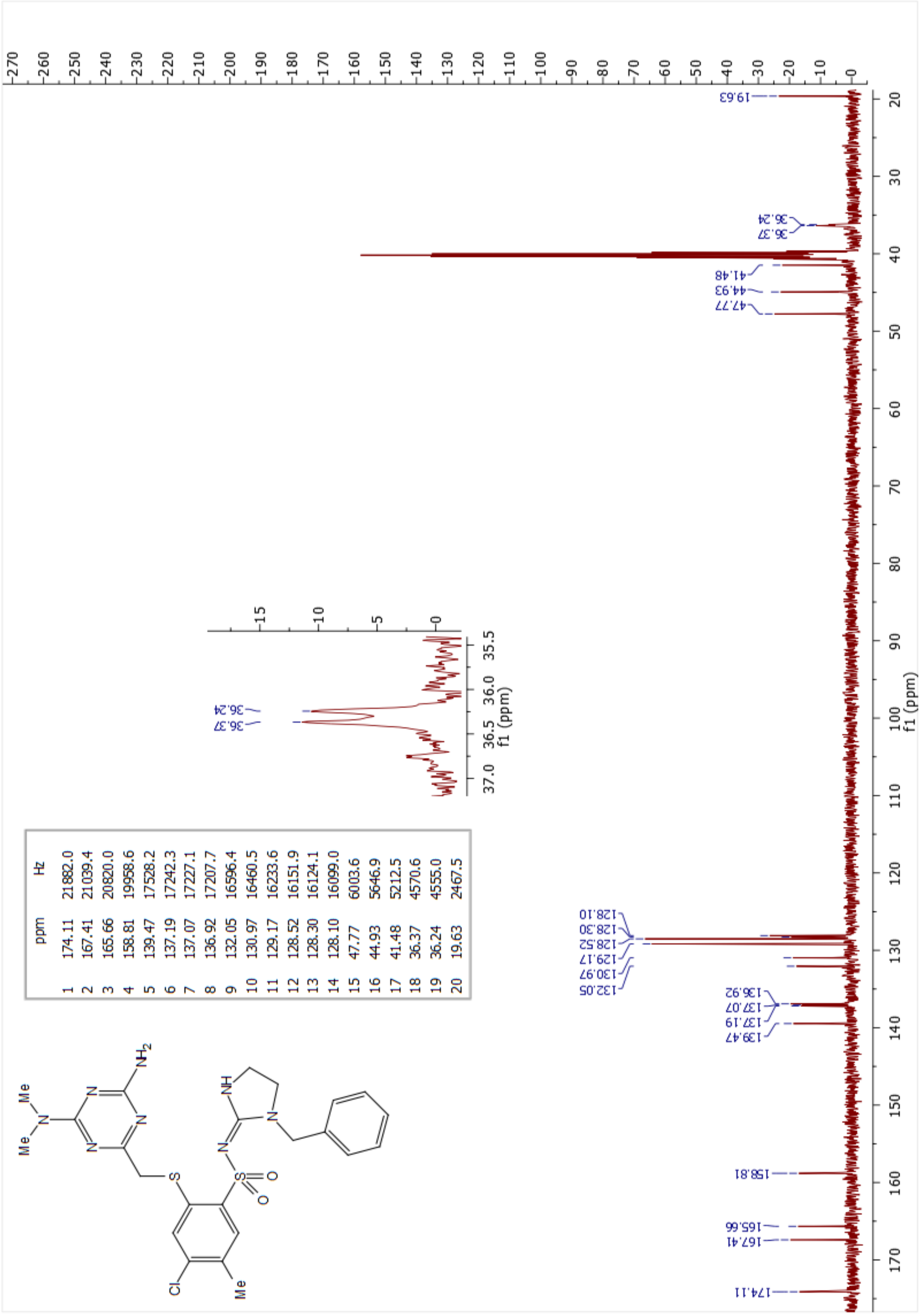

Spectrum 18. ^{13}C NMR of compound **45** (125 MHz, DMSO-d_6)

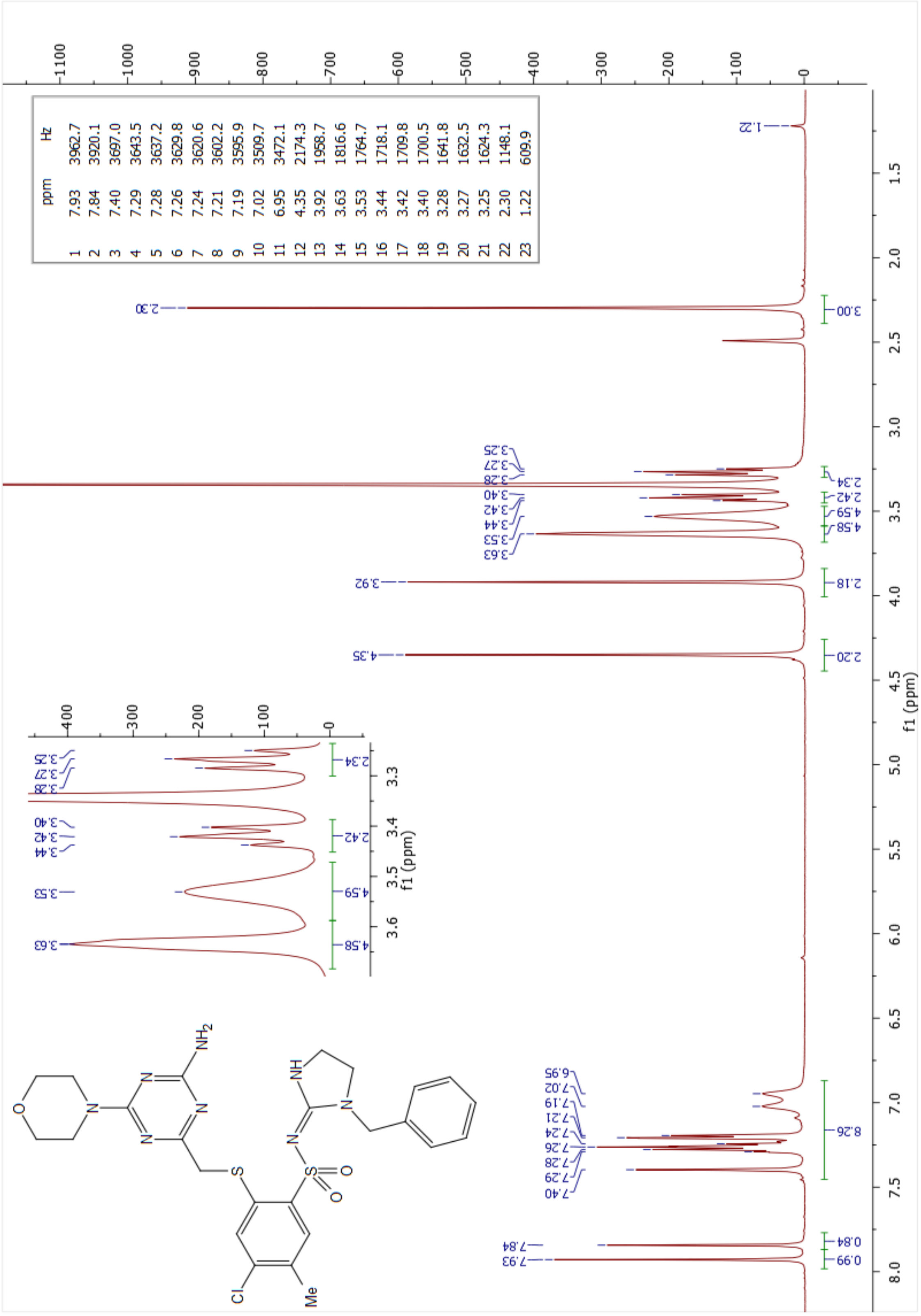

Spectrum 19. ^{1}H NMR of compound **46** (500 MHz, DMSO-d_6)

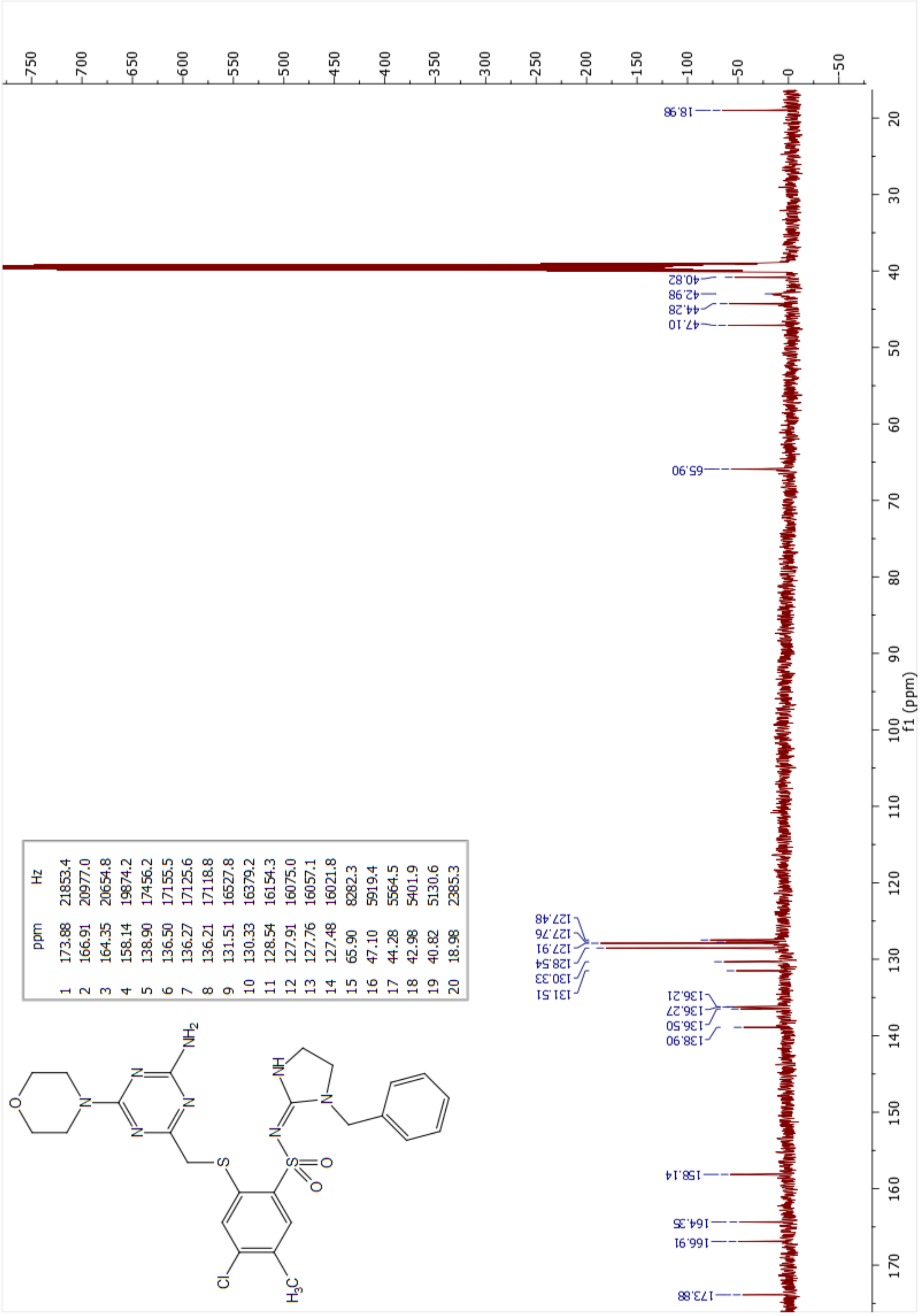

Spectrum 20. ^{13}C NMR of compound **46** (125 MHz, DMSO-d_6)

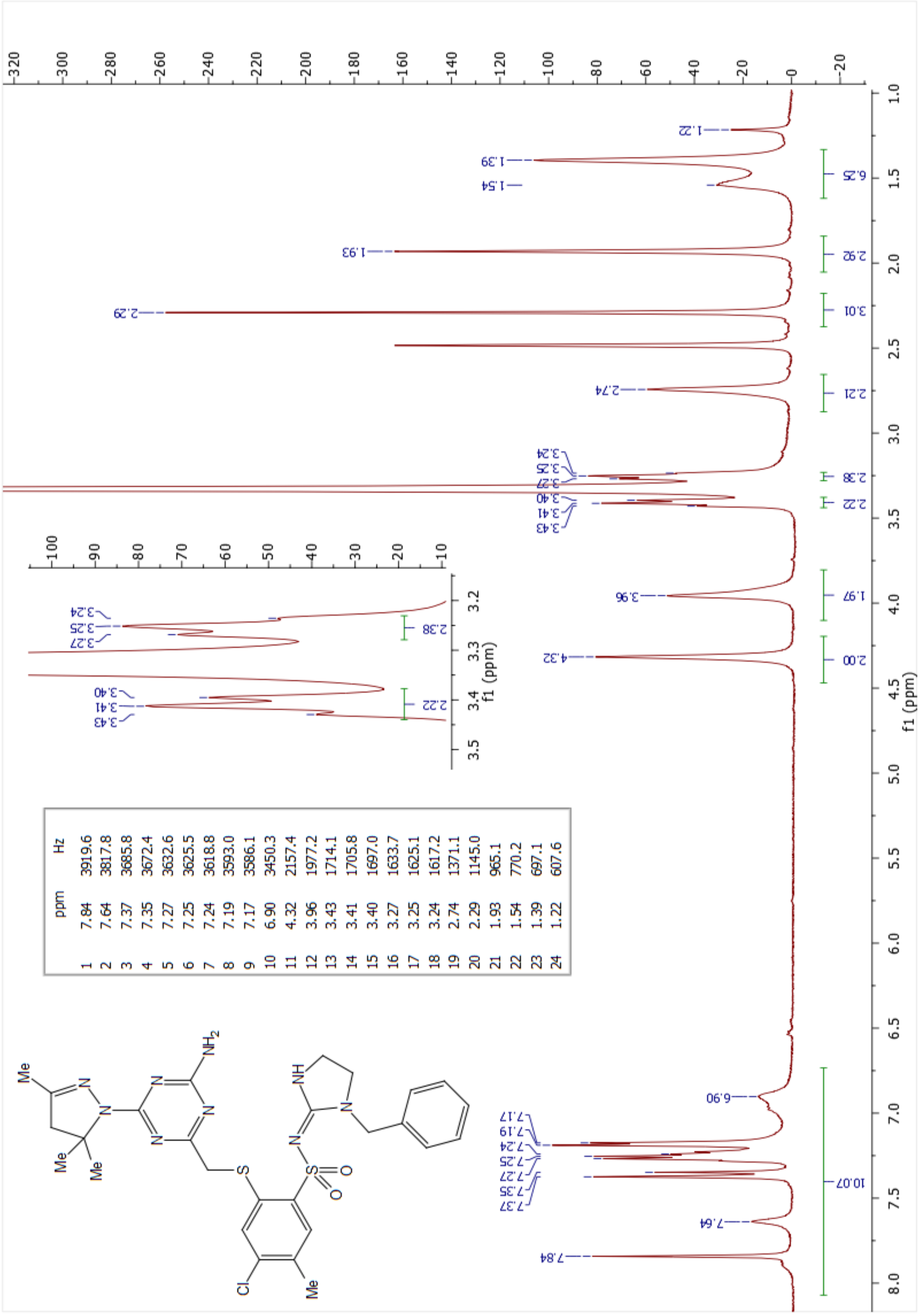

Spectrum 21. ^{1}H NMR of compound **47** (500 MHz, DMSO-d$_6$)

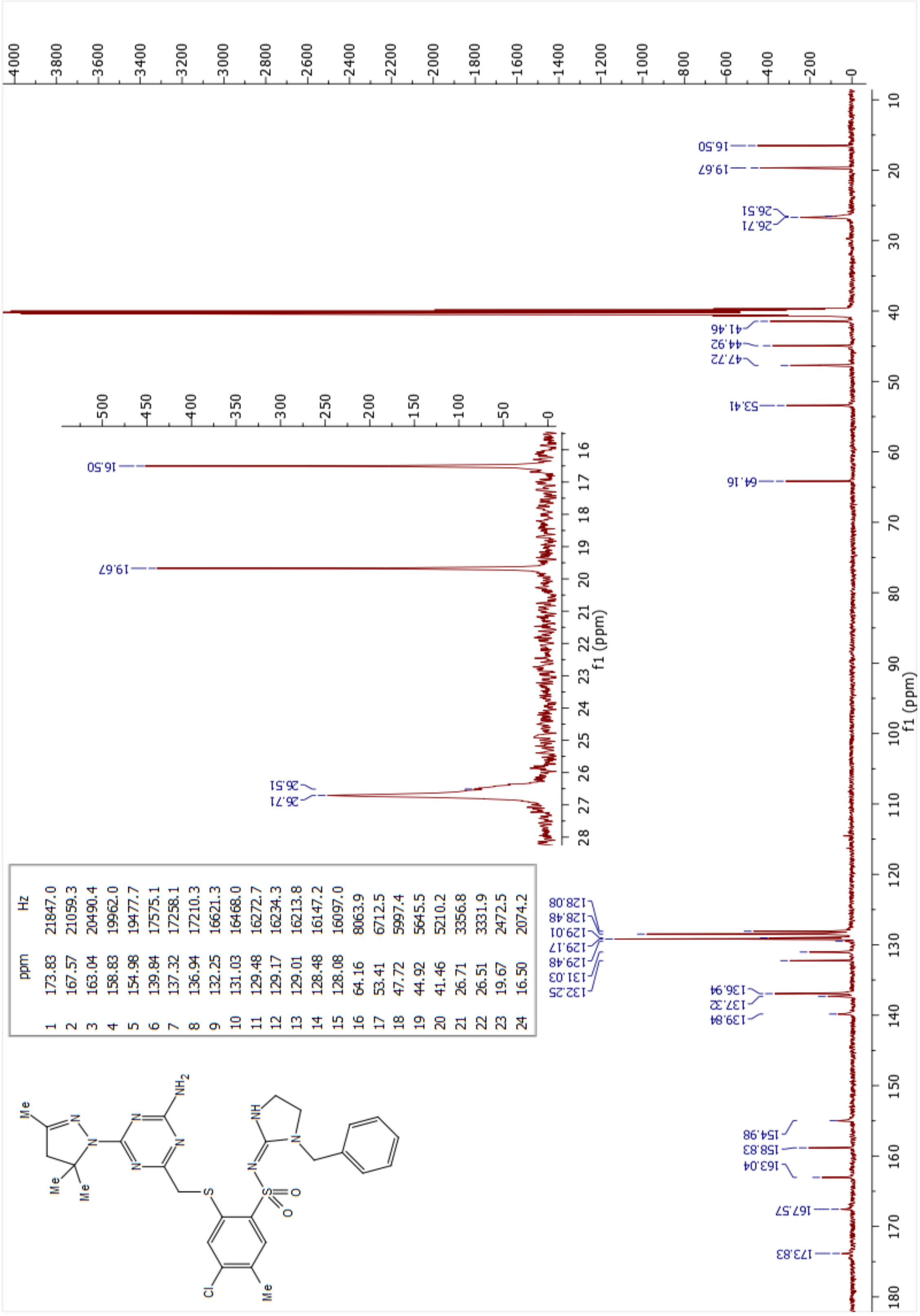

	ppm	Hz
1	173.83	21847.0
2	167.57	21059.3
3	163.04	20490.4
4	158.83	19962.0
5	154.98	19477.7
6	139.84	17575.1
7	137.32	17258.1
8	136.94	17210.3
9	132.25	16621.3
10	131.03	16468.0
11	129.48	16272.7
12	129.17	16234.3
13	129.01	16213.8
14	128.48	16147.2
15	128.08	16097.0
16	64.16	8063.9
17	53.41	6712.5
18	47.72	5997.4
19	44.92	5645.5
20	41.46	5210.2
21	26.71	3356.8
22	26.51	3331.9
23	19.67	2472.5
24	16.50	2074.2

Spectrum 22. ^{13}C NMR of compound **47** (125 MHz, DMSO-d_6)

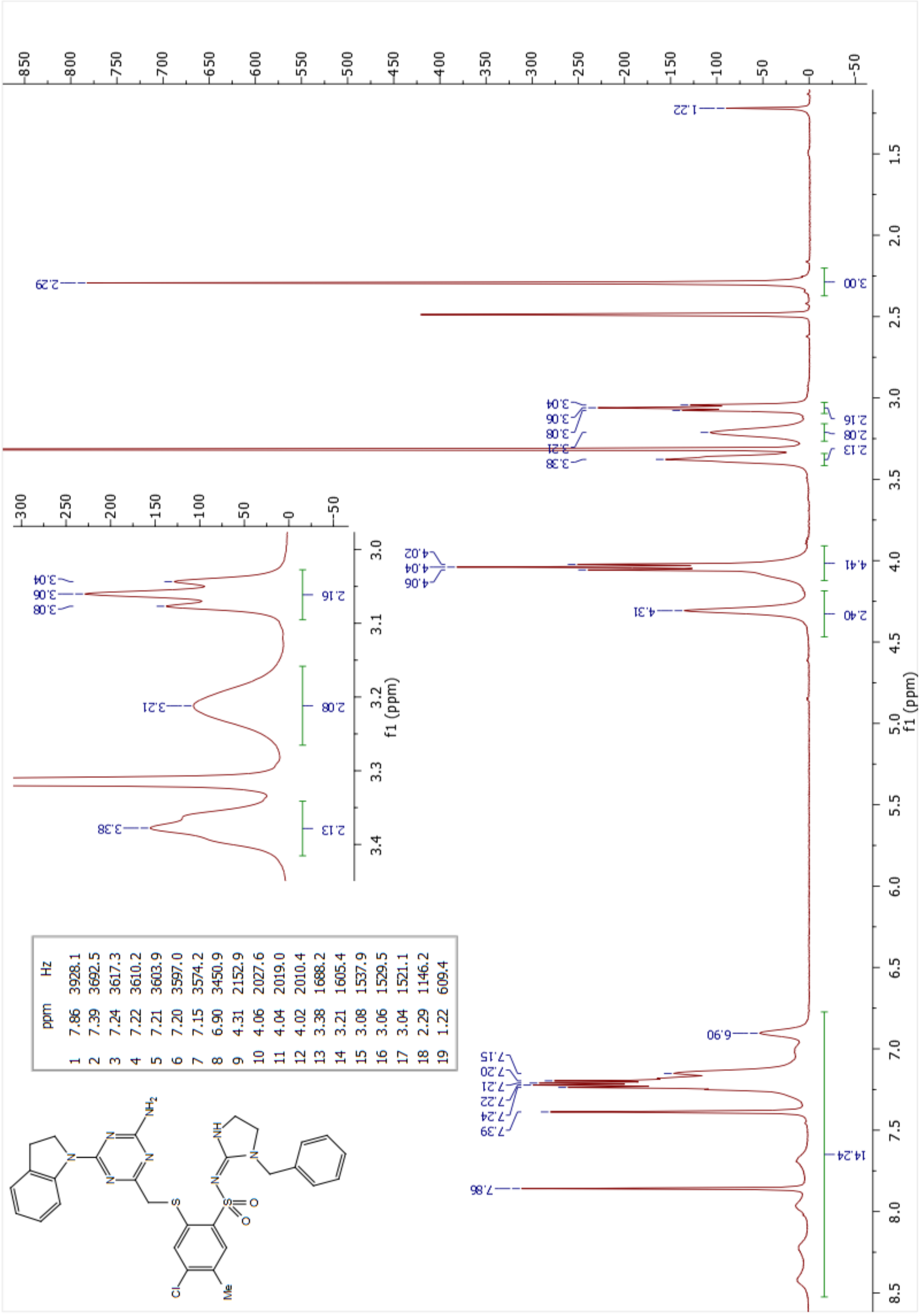

Spectrum 23. ^{1}H NMR of compound **48** (500 MHz, DMSO-d6)

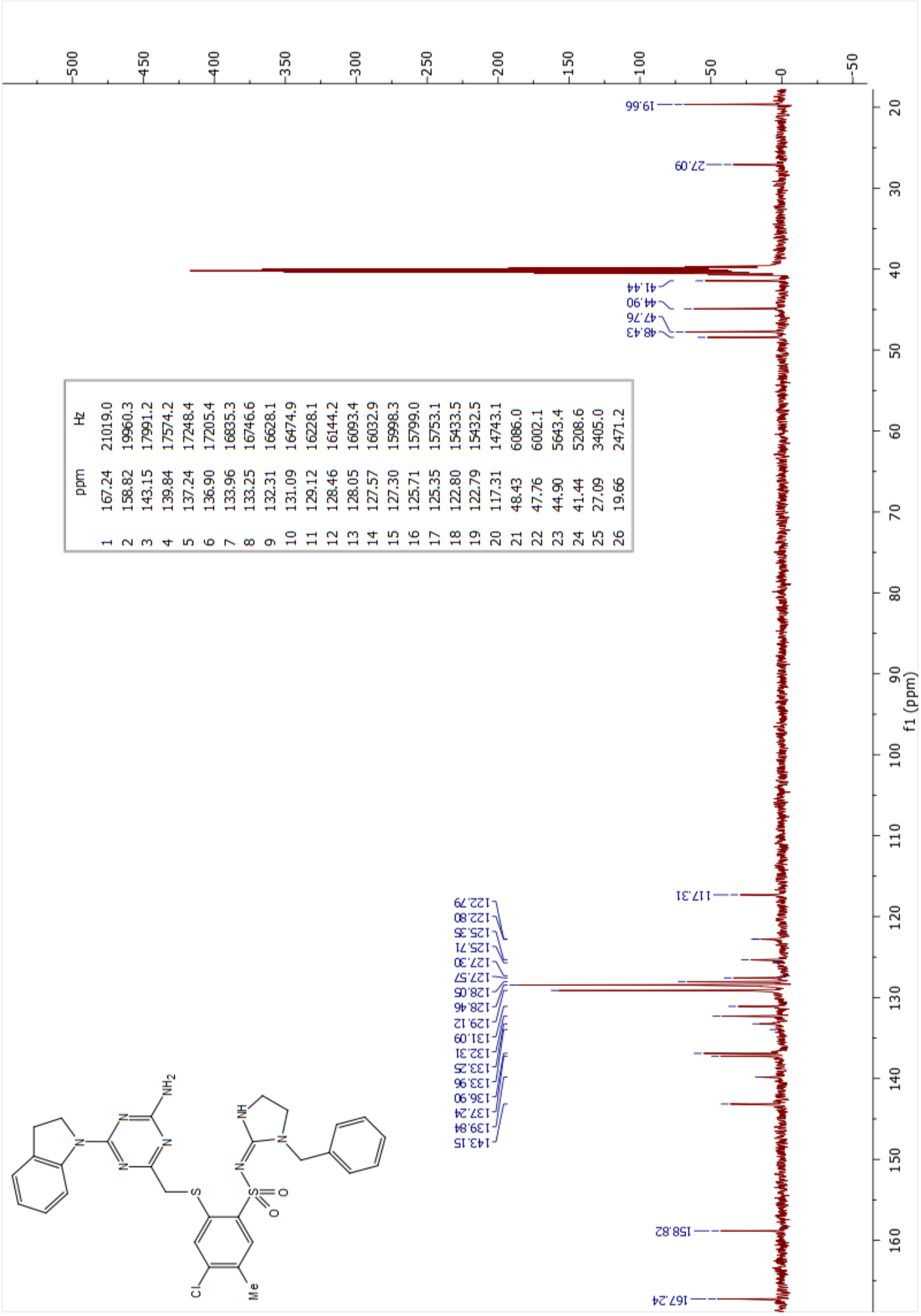

Spectrum 24. ^{13}C NMR of compound **48** (125 MHz, DMSO-d$_6$)

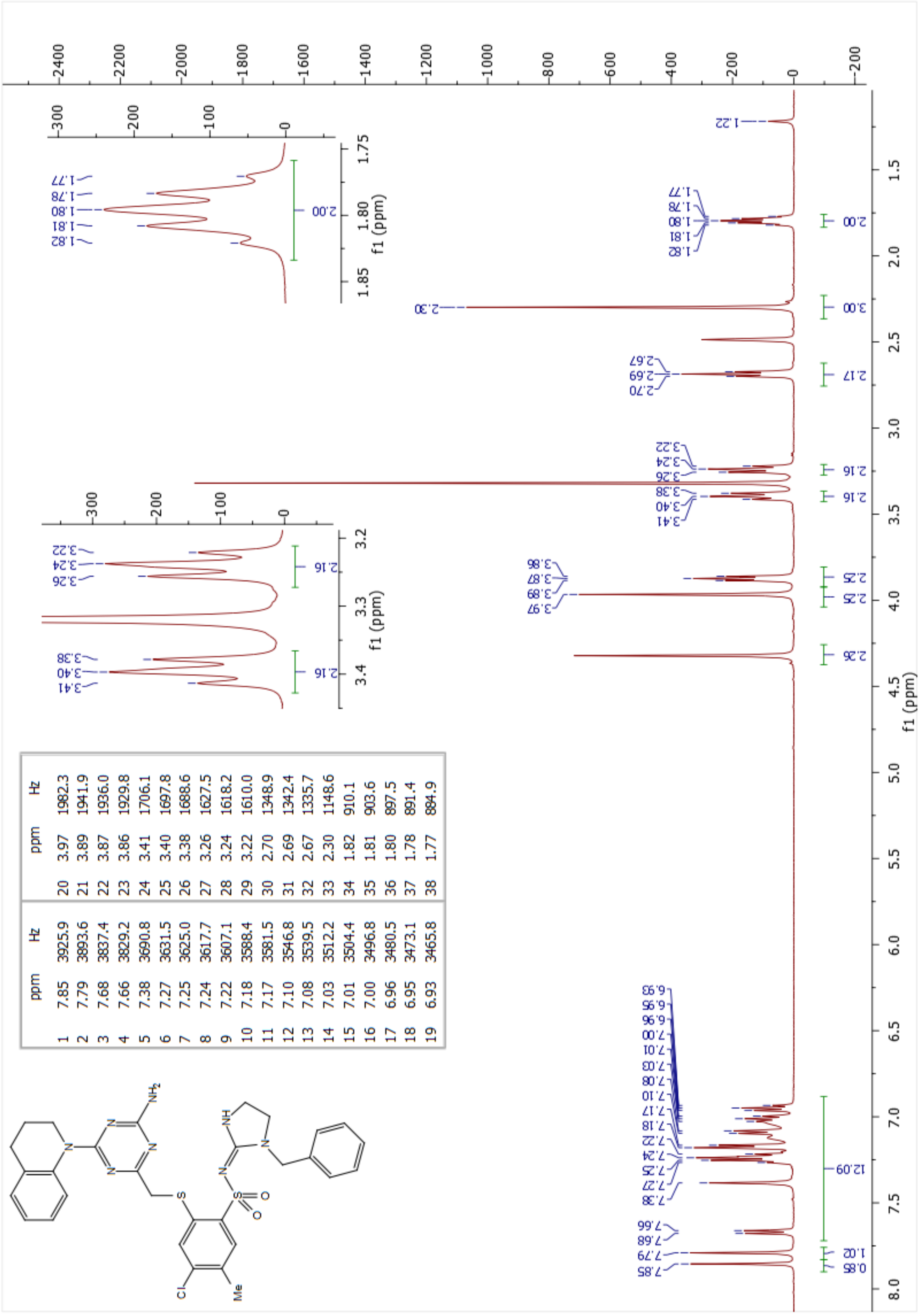

Spectrum 25. ^{1}H NMR of compound **49** (500 MHz, DMSO-d_6)

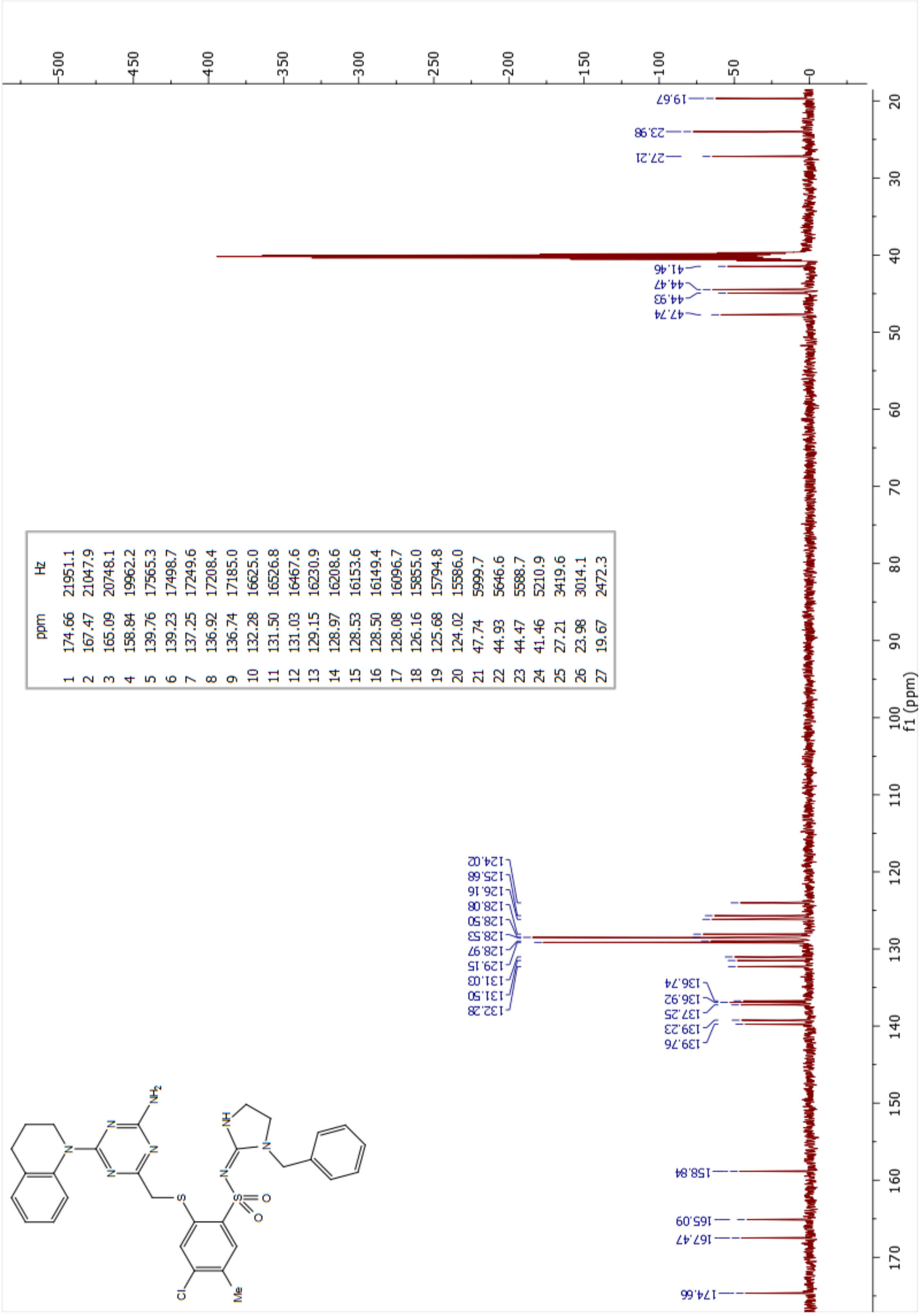

Spectrum 26. ^{13}C NMR of compound **49** (125 MHz, DMSO-d_6)

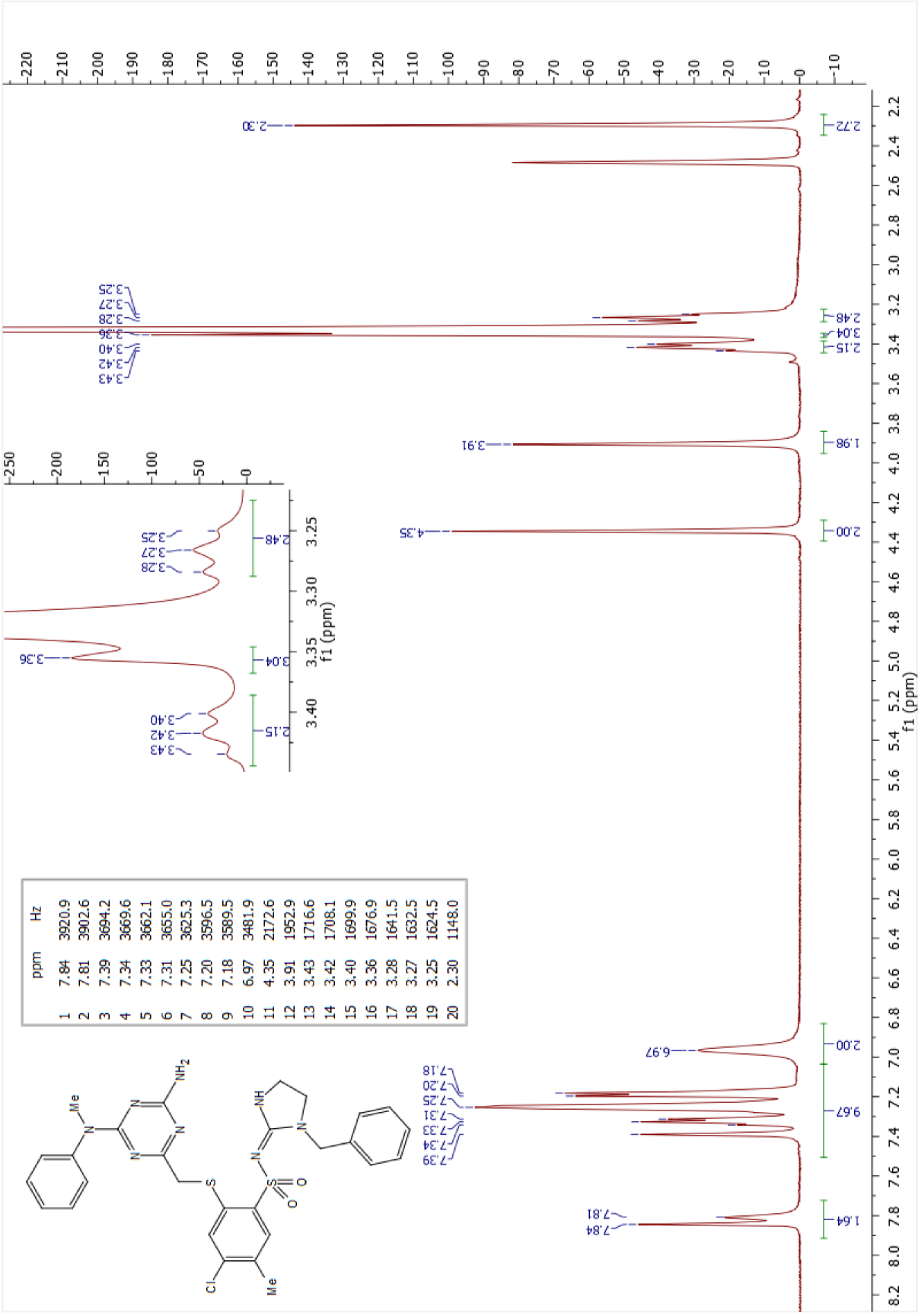

Spectrum 27. ^{1}H NMR of compound **54** (500 MHz, DMSO-d$_6$)

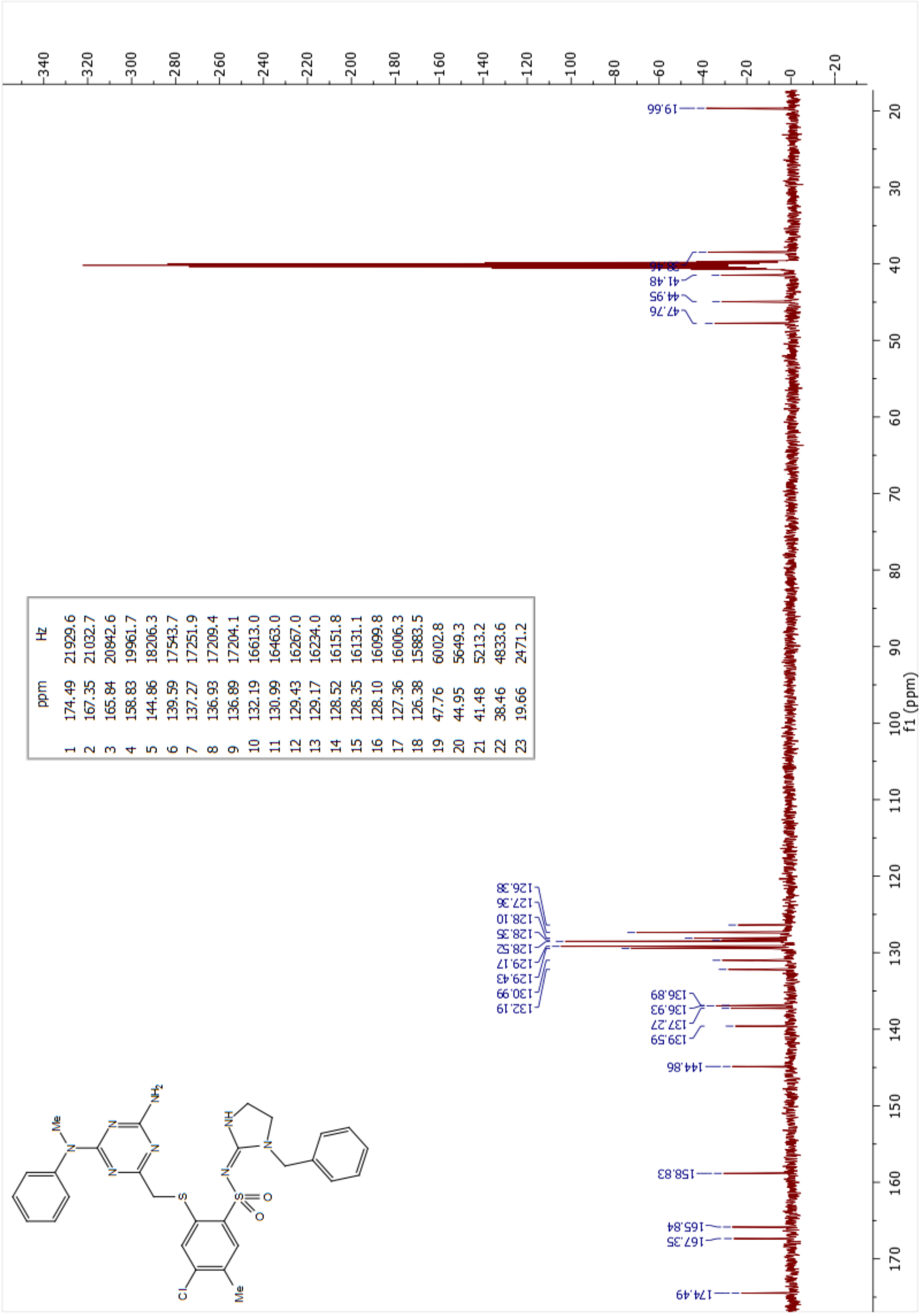

Spectrum 28. ^{13}C NMR of compound **54** (125 MHz, DMSO-d$_6$)

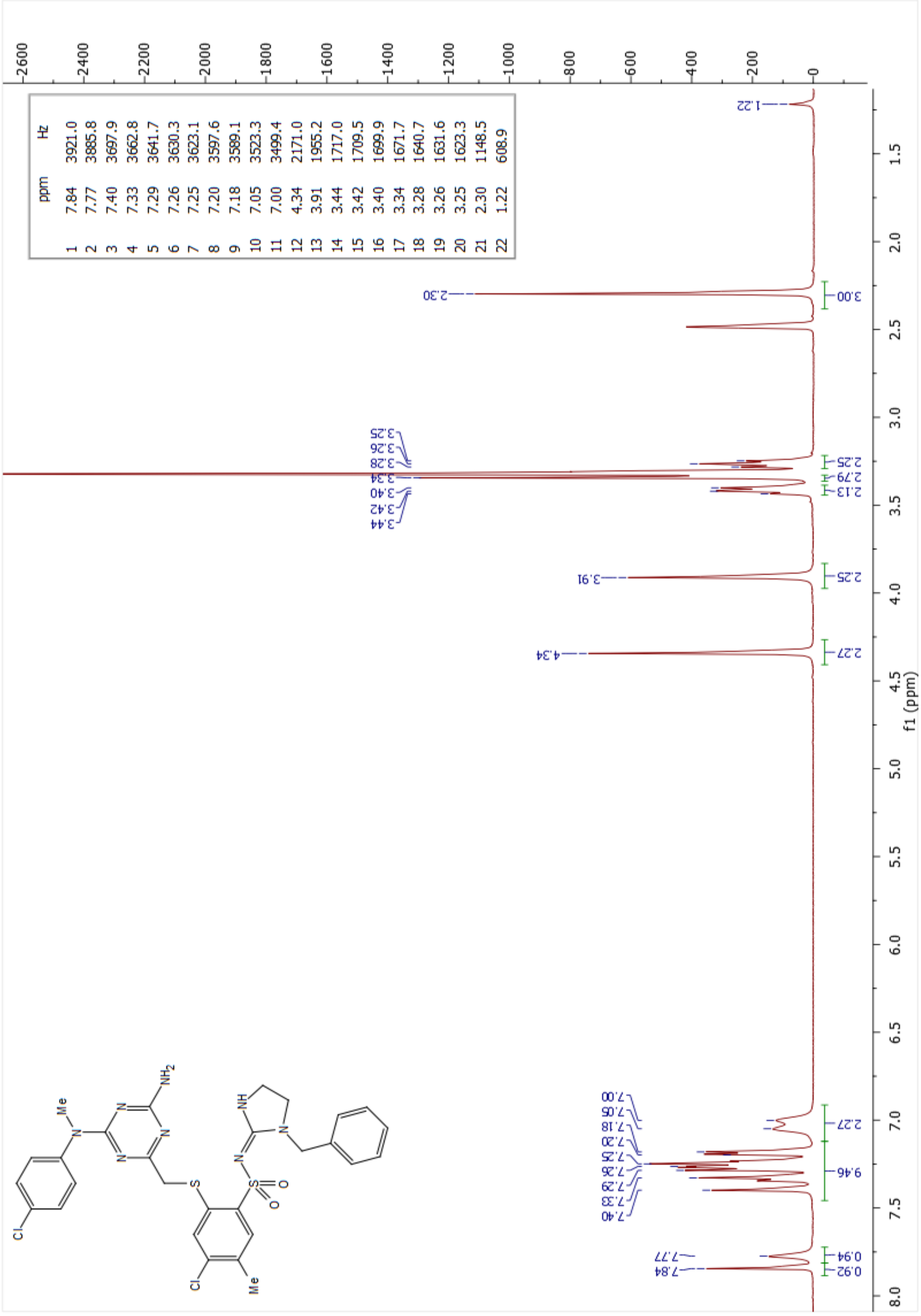

Spectrum 29. ^{1}H NMR of compound **55** (500 MHz, DMSO-d_6)

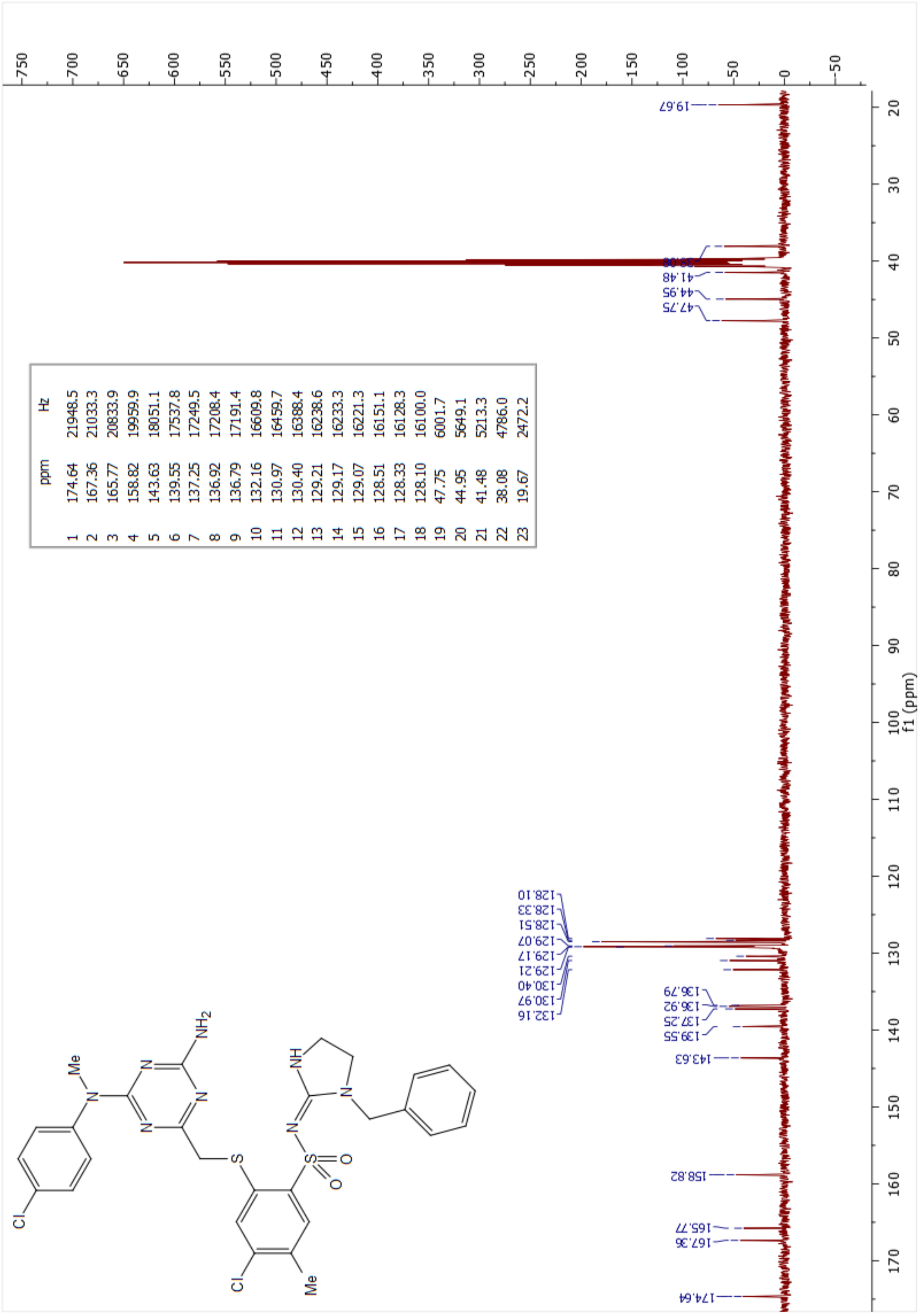

Spectrum 30. ^{13}C NMR of compound **55** (125 MHz, DMSO-d_6)

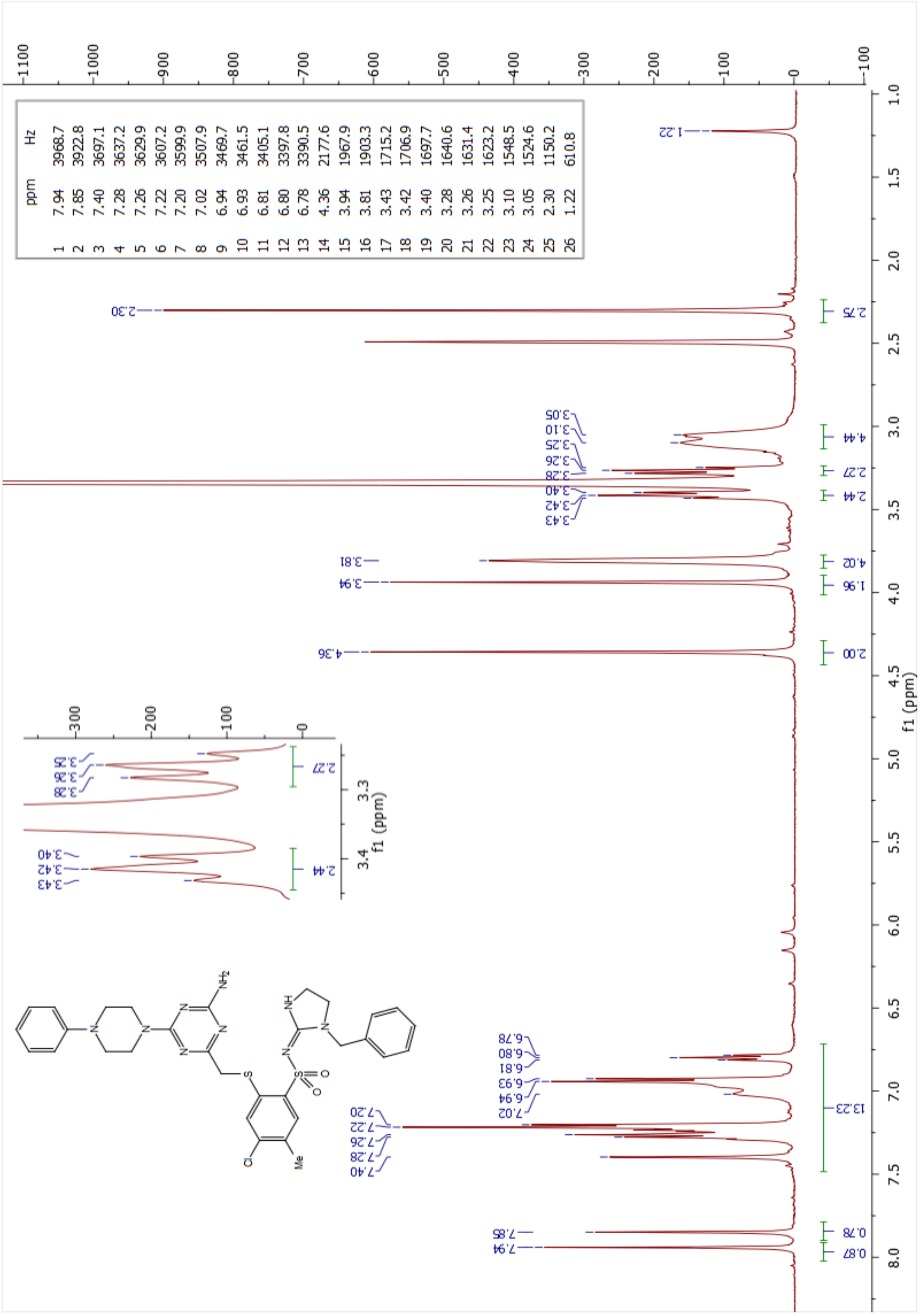

Spectrum 31. ^{1}H NMR of compound **57** (500 MHz, DMSO-d_6)

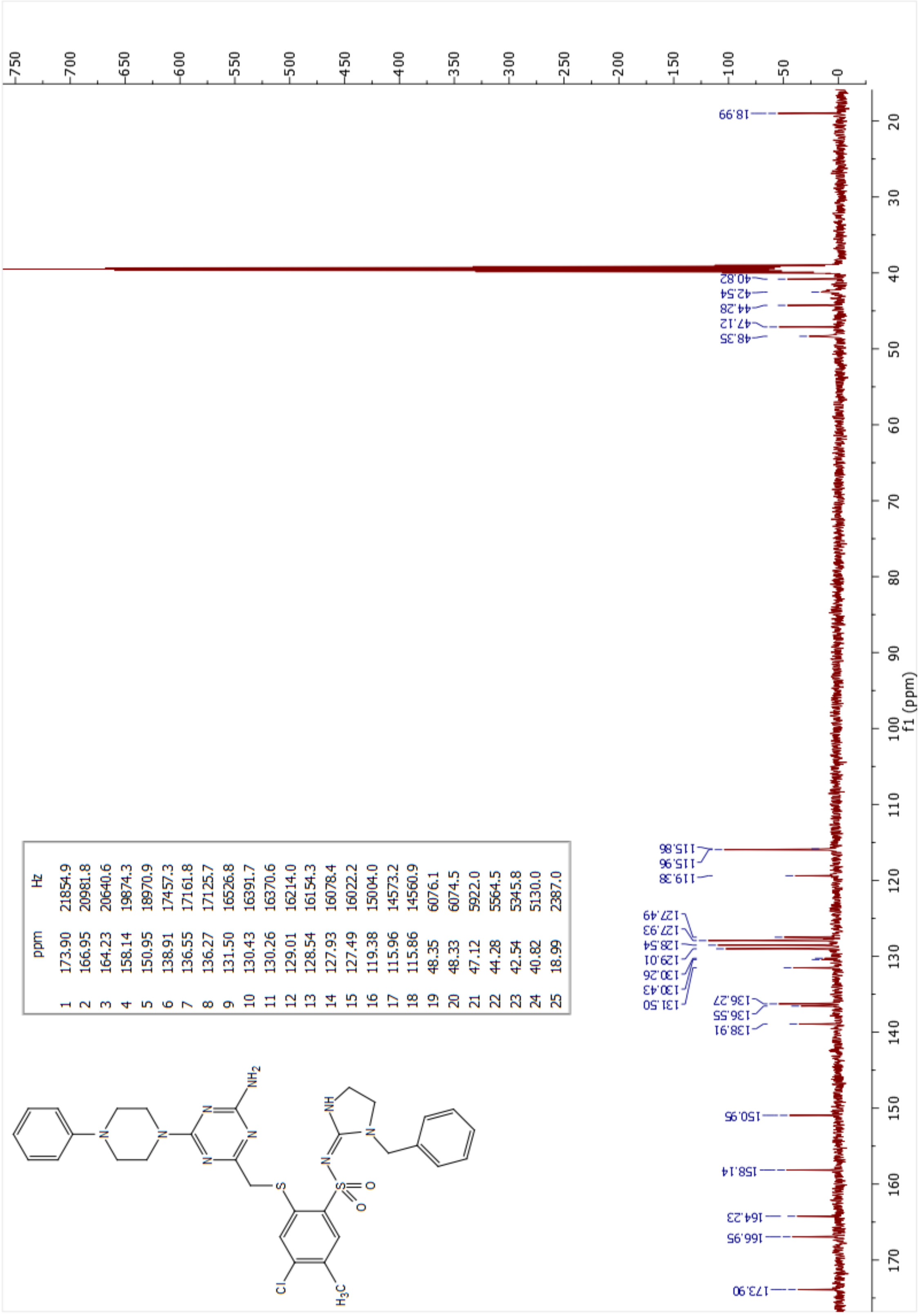

Spectrum 32. ^{13}C NMR of compound **57** (125 MHz, DMSO-d_6)

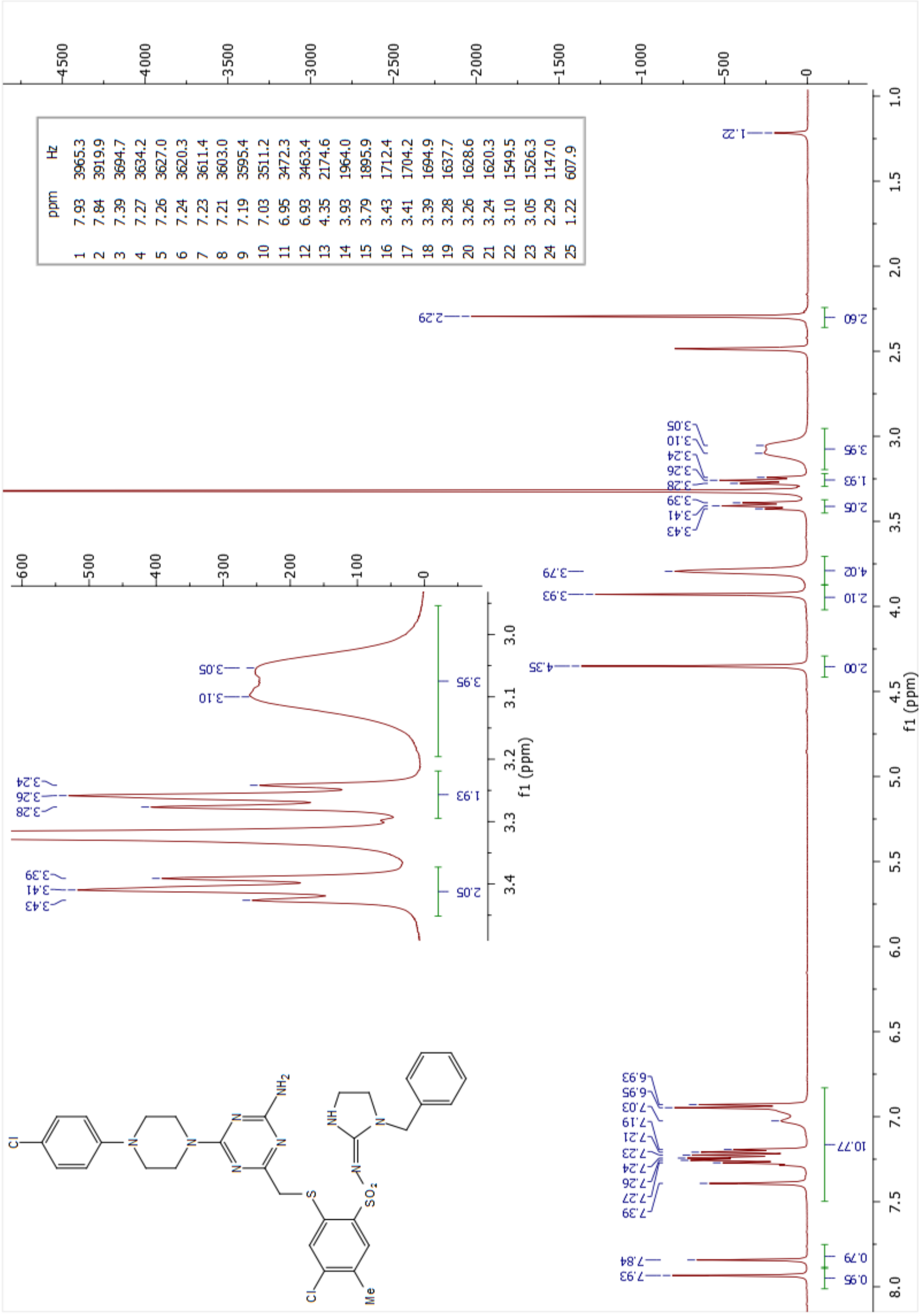

Spectrum 33. ^{1}H NMR of compound **62** (500 MHz, DMSO-d_6)

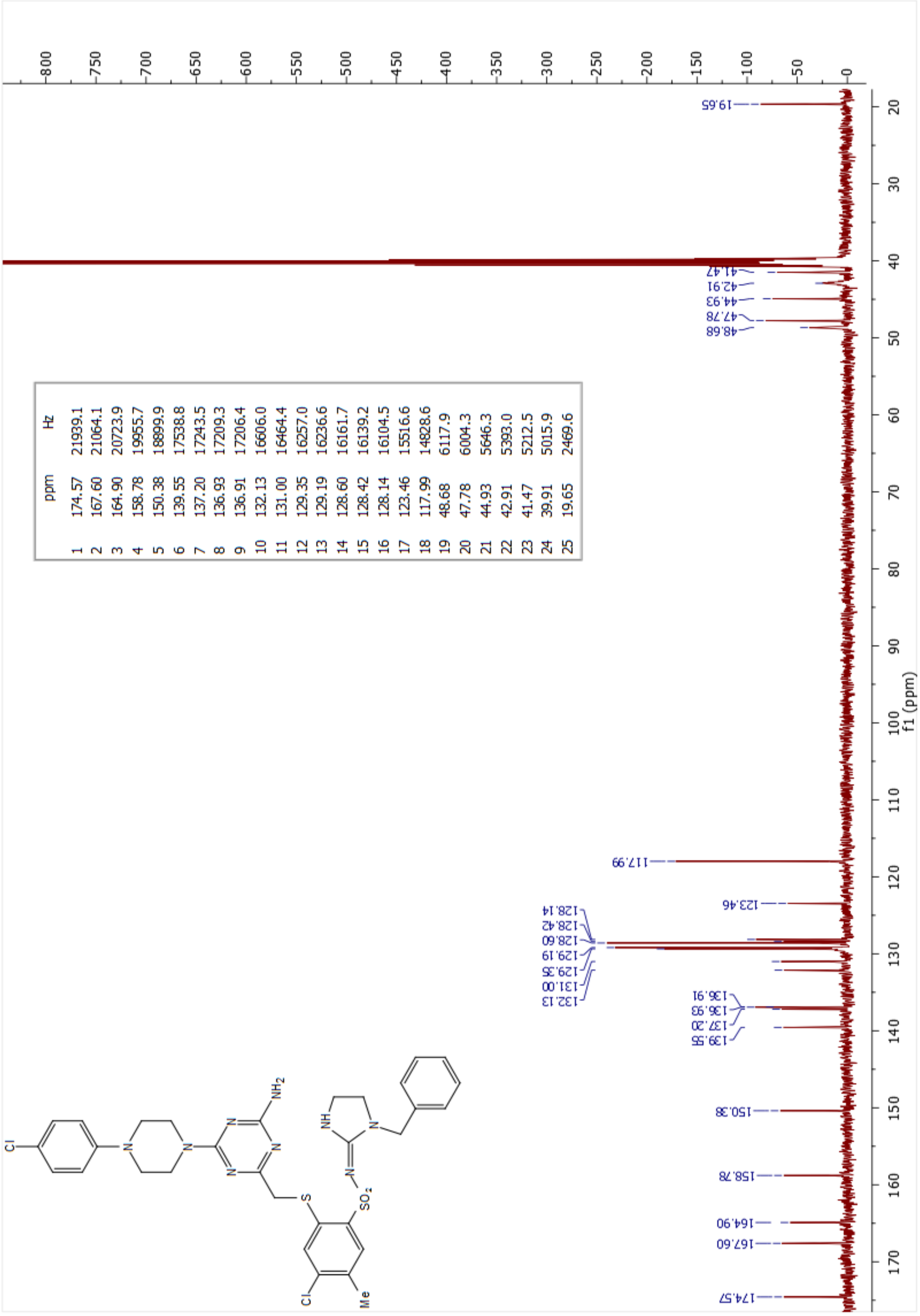

Spectrum 34. ^{13}C NMR of compound **62** (125 MHz, DMSO-d_6)

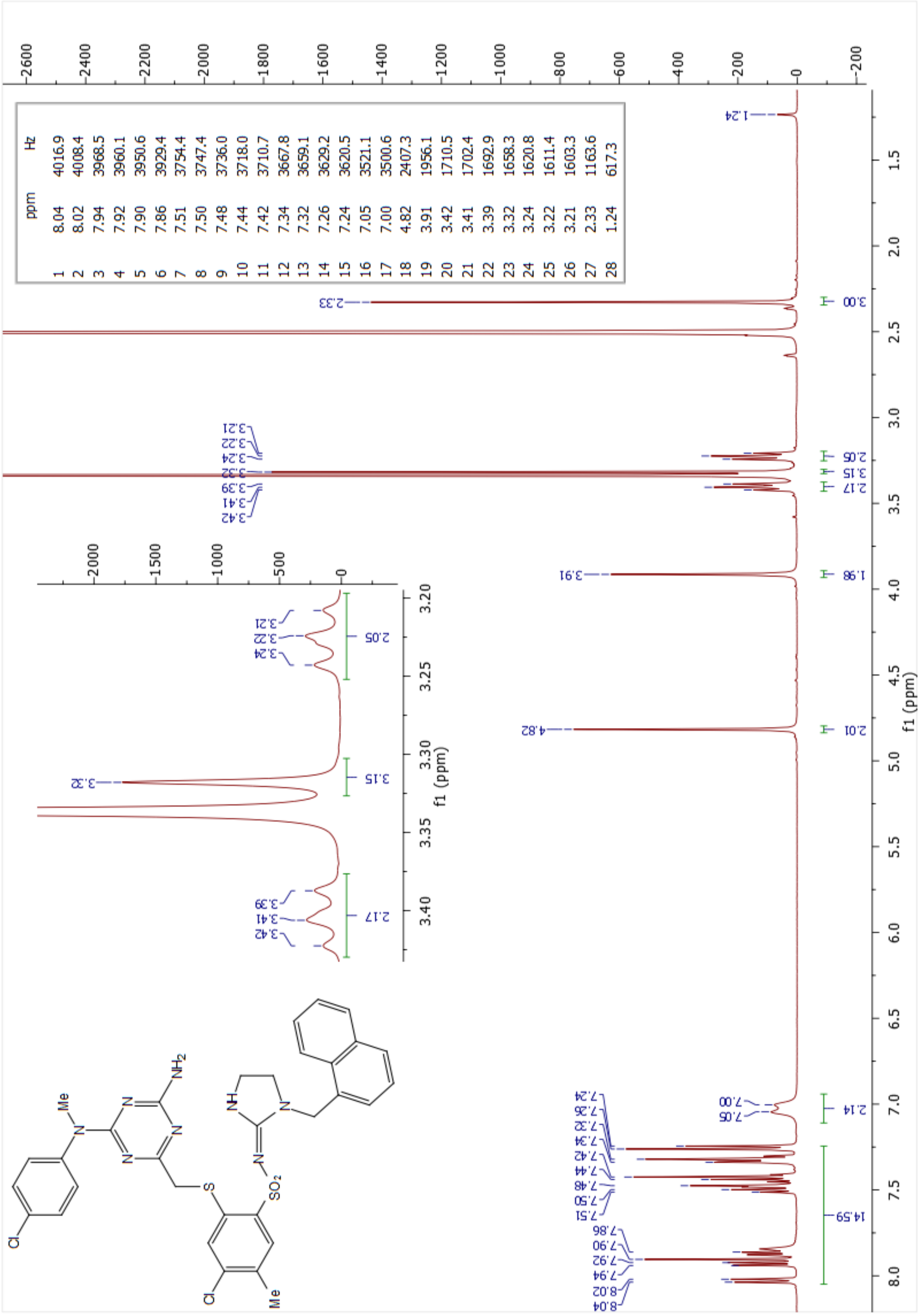

Spectrum 35. ^{1}H NMR of compound **76** (500 MHz, DMSO-d$_6$)

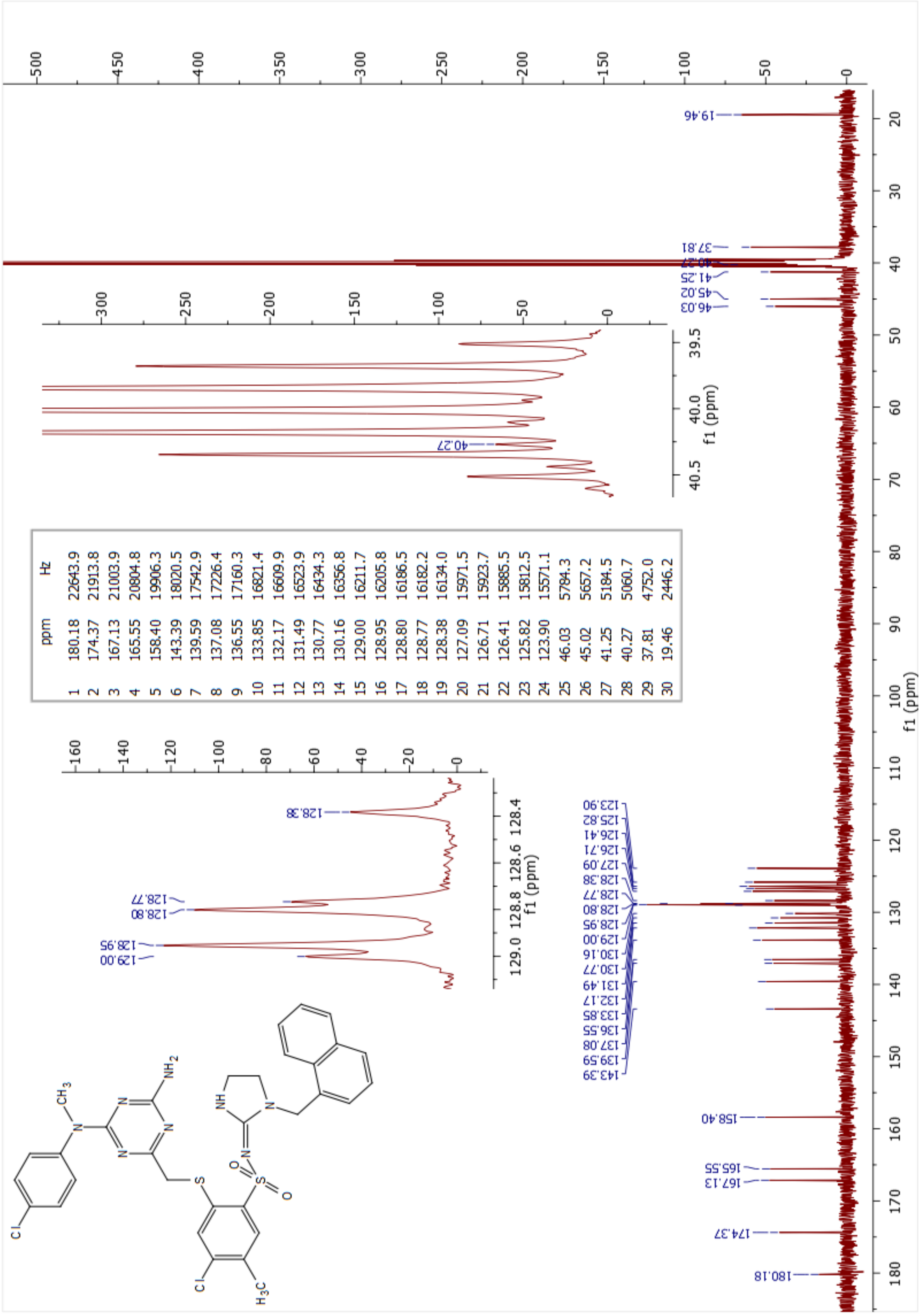

Spectrum 36. ^{13}C NMR of compound **76** (125 MHz, DMSO-d$_6$)

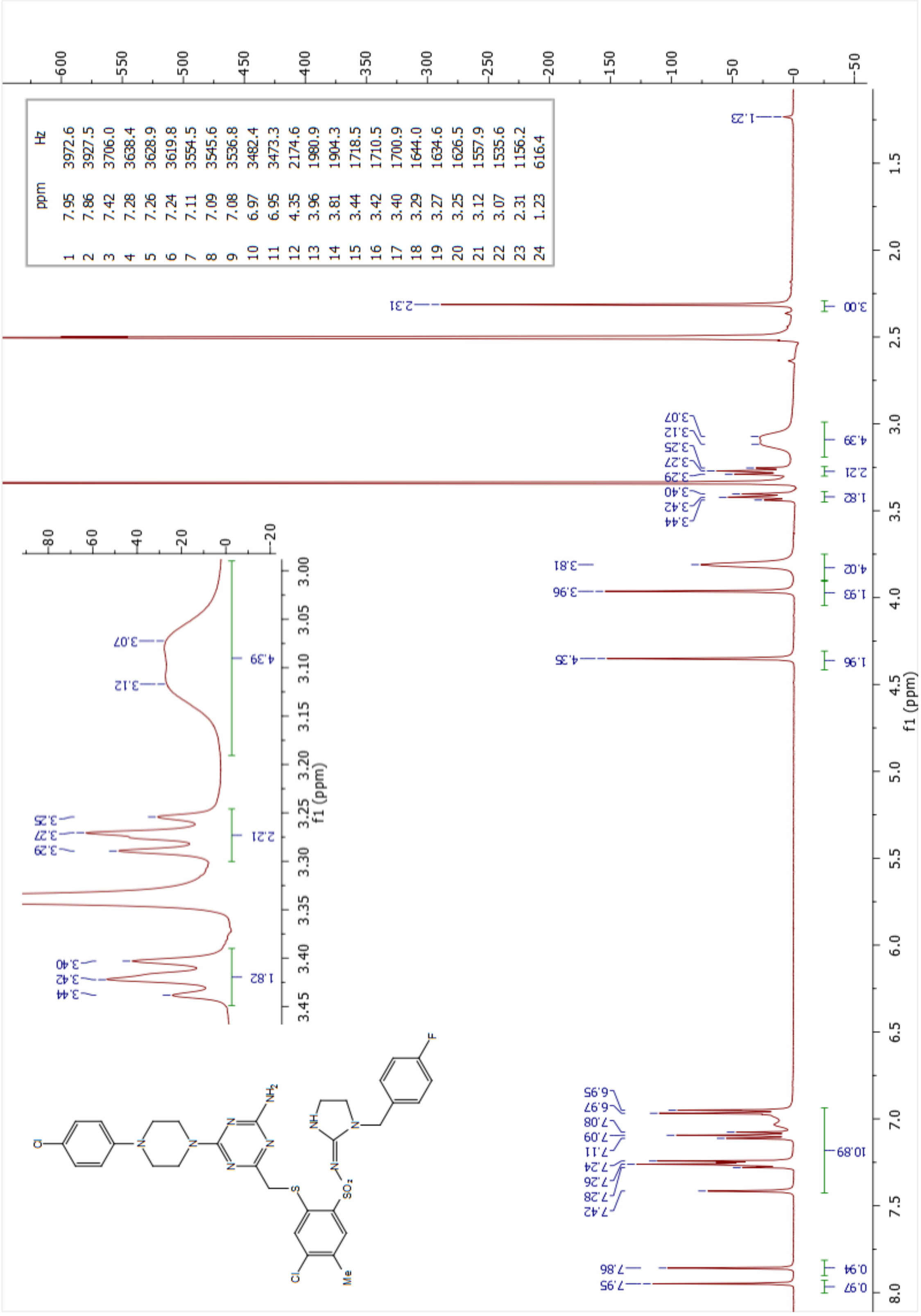

Spectrum 37. ^{1}H NMR of compound **96** (500 MHz, DMSO-d$_6$)

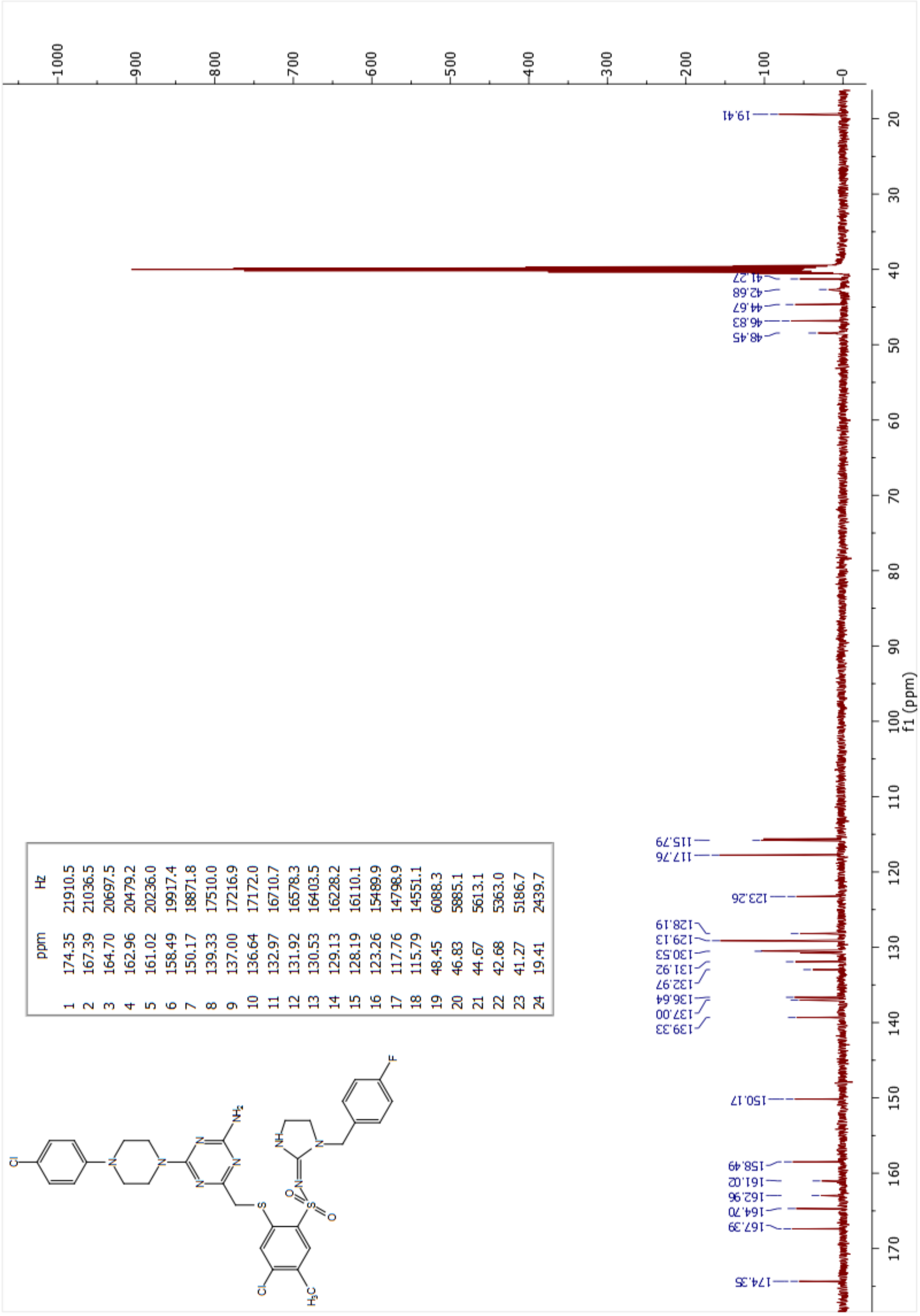

Spectrum 38. ^{13}C NMR of compound **96** (125 MHz, DMSO-d_6)

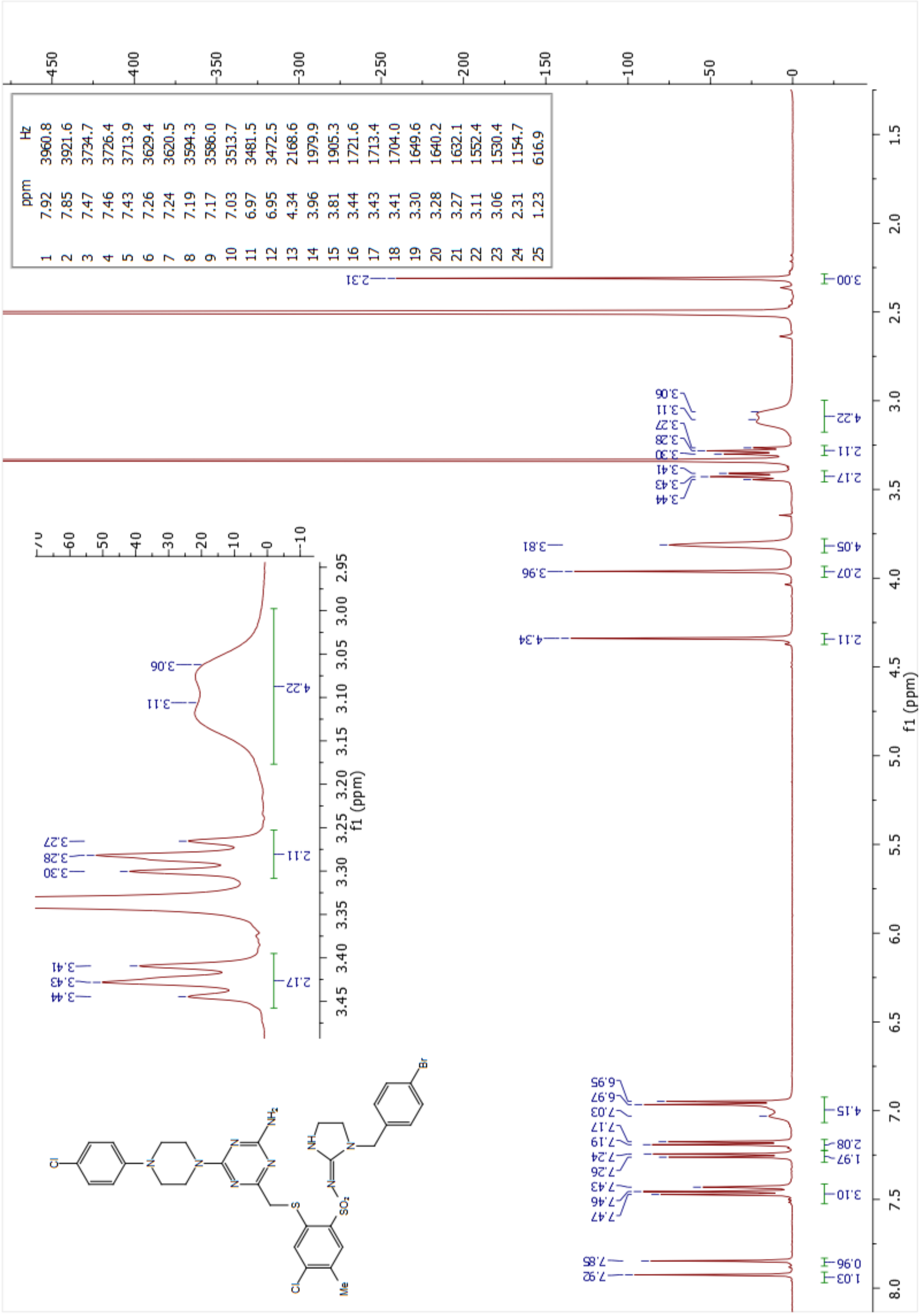

Spectrum 39. ^{1}H NMR of compound **109** (500 MHz, DMSO-d_6)

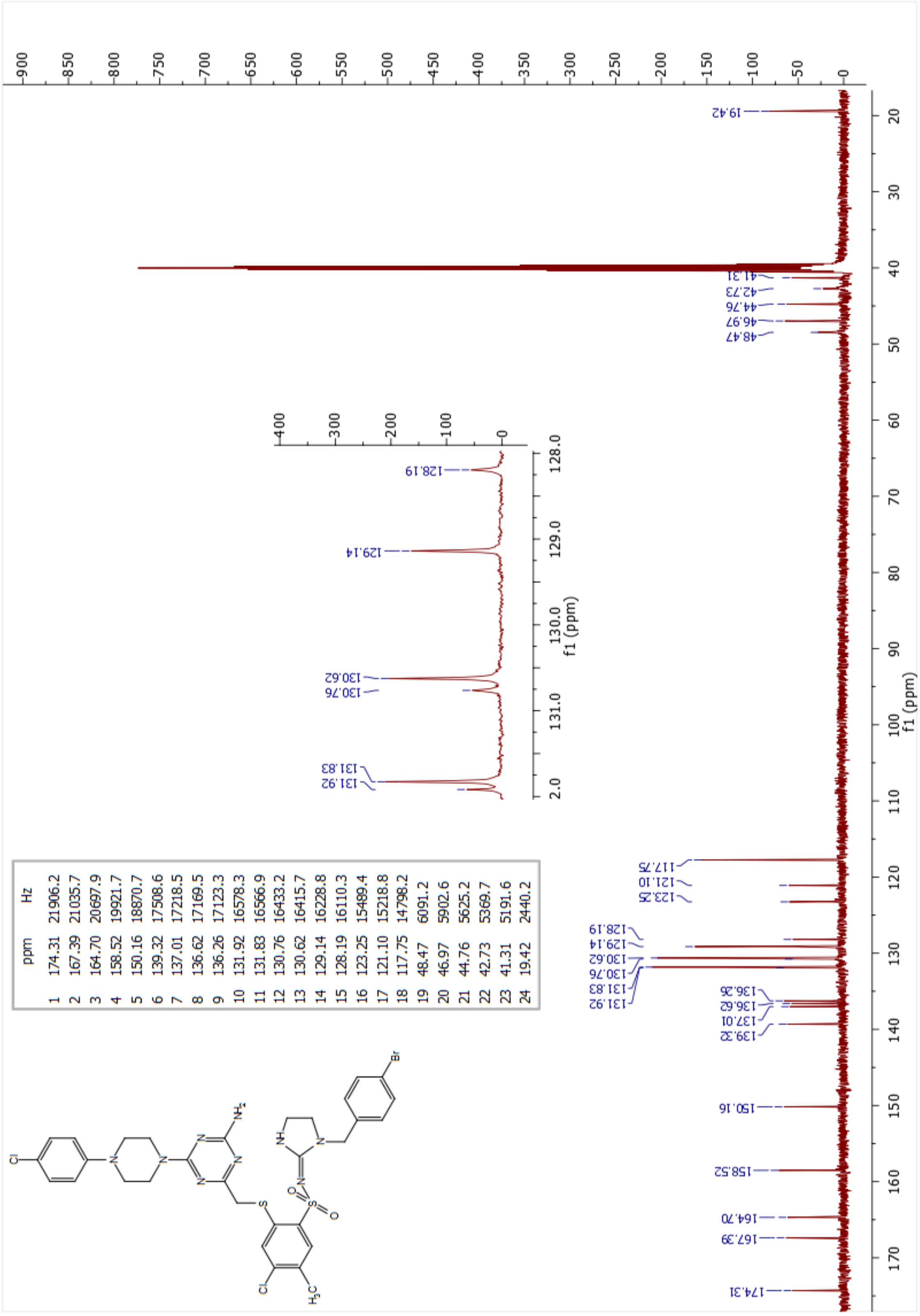

Spectrum 40. ^{13}C NMR of compound **109** (125 MHz, DMSO-d_6)

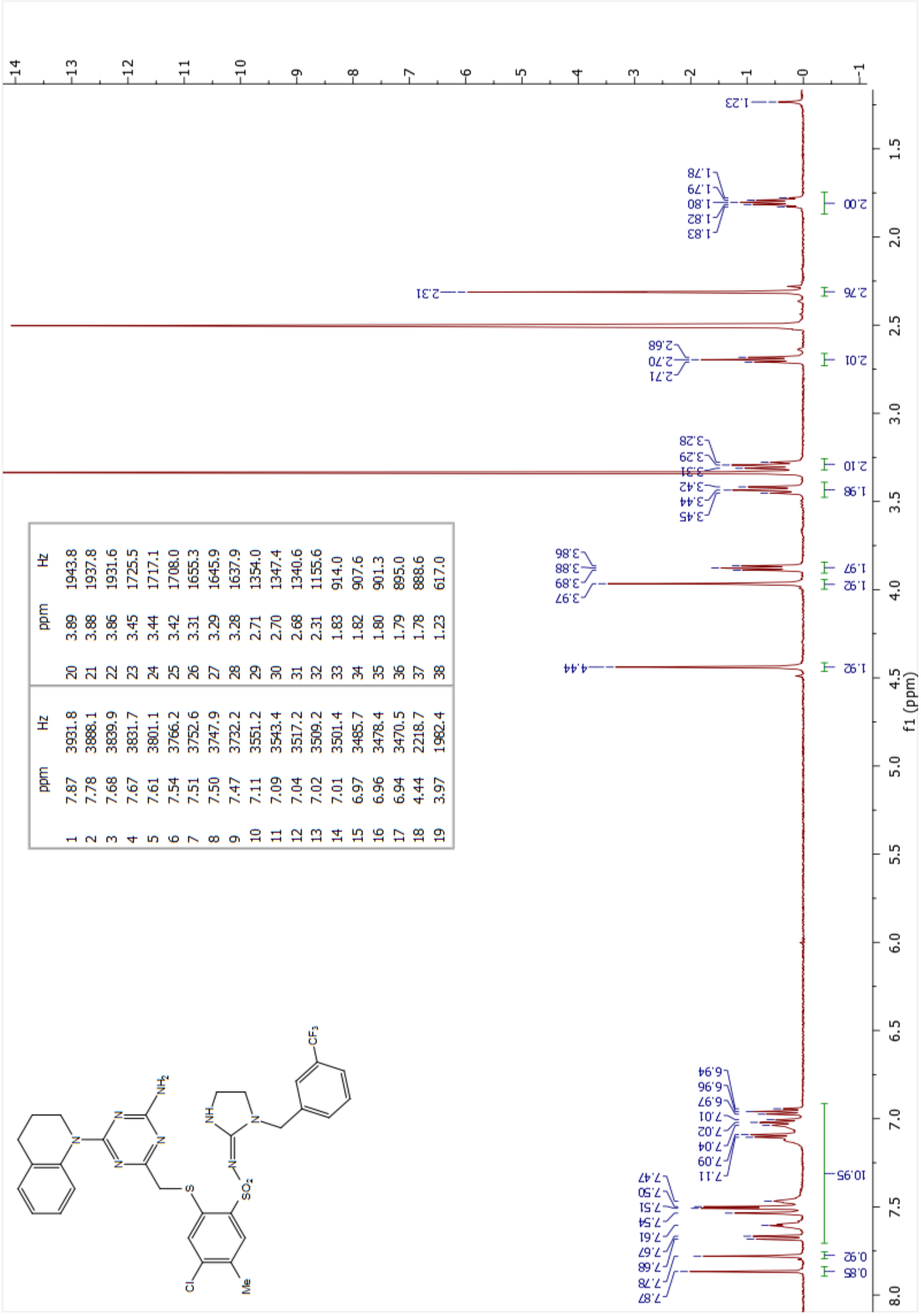

Spectrum 41. ^{1}H NMR of compound **112** (500 MHz, DMSO-d$_6$)

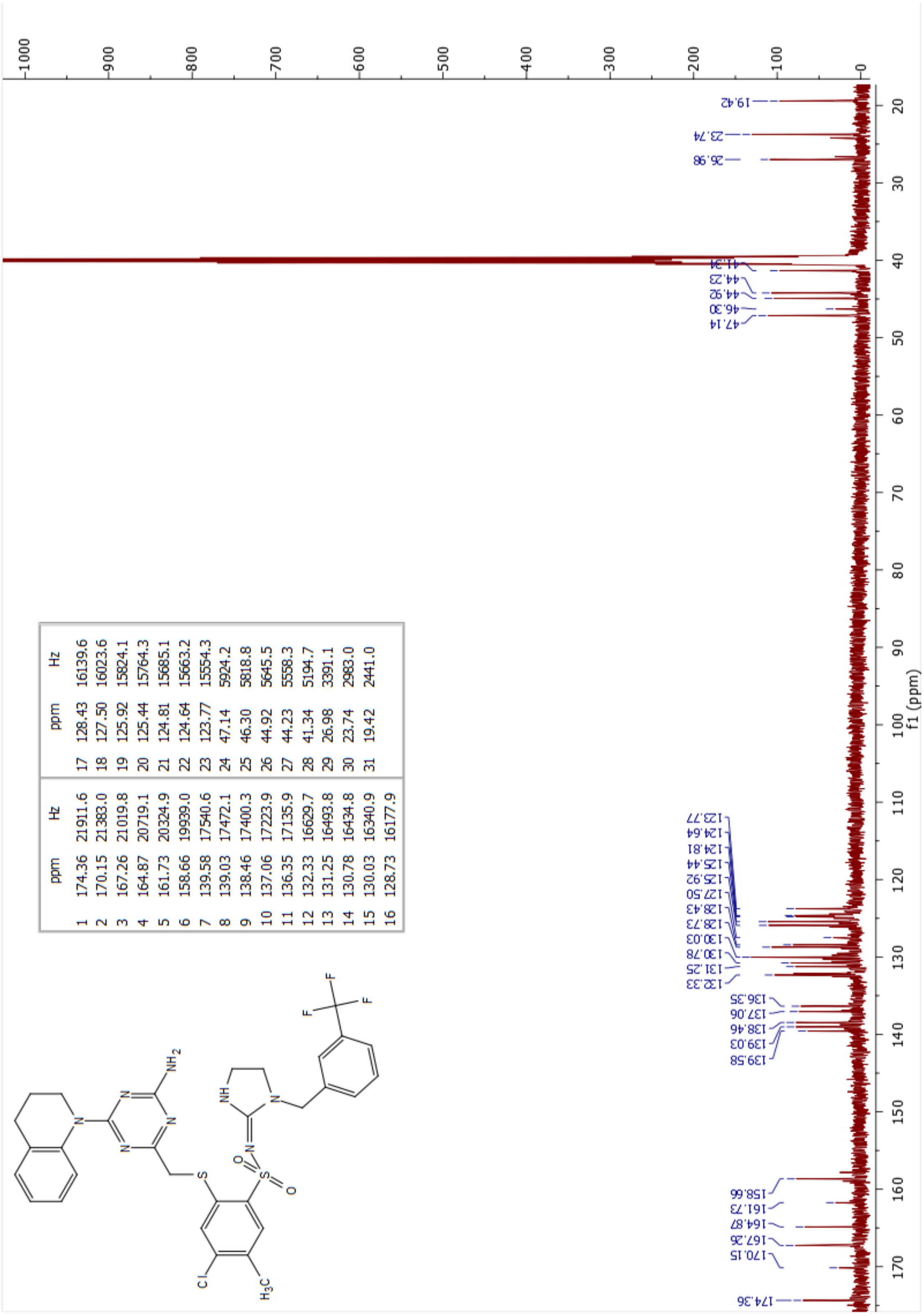

Spectrum 42. ^{13}C NMR of compound **112** (125 MHz, DMSO-d$_6$)

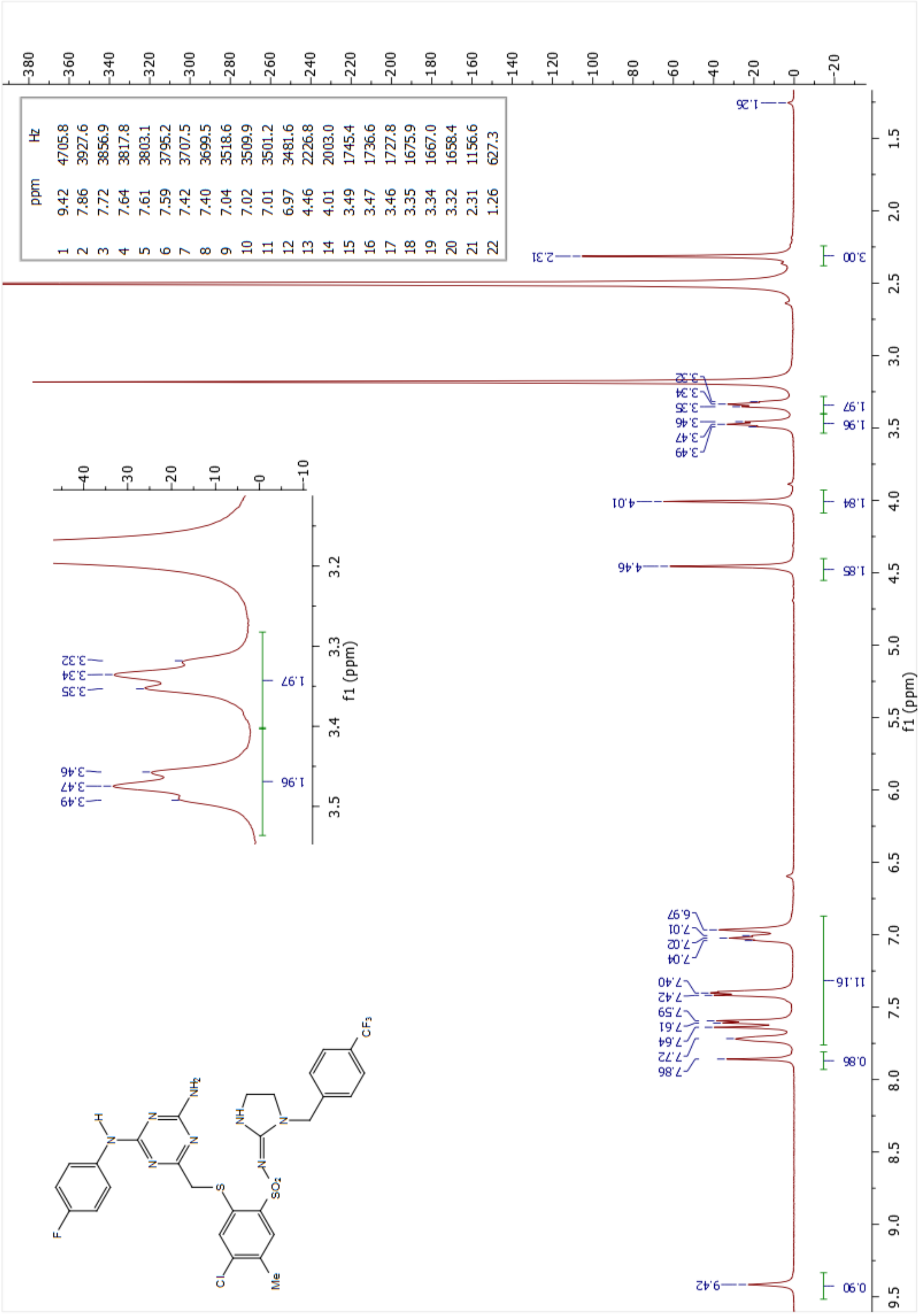

Spectrum 43. ^{1}H NMR of compound **123** (500 MHz, DMSO-d_6 in 60°C)

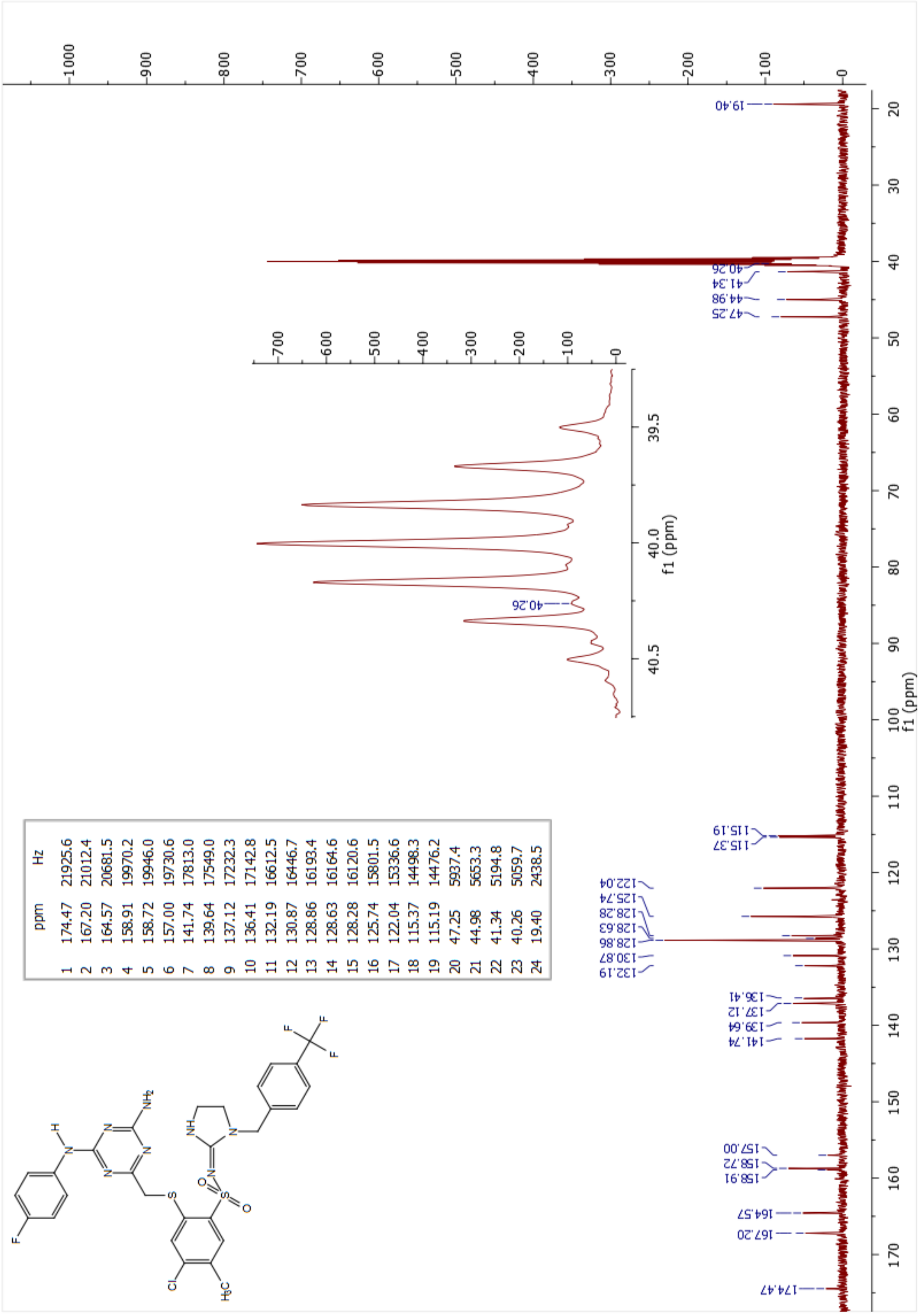

	ppm	Hz
1	174.47	21925.6
2	167.20	21012.4
3	164.57	20681.5
4	158.91	19970.2
5	158.72	19946.0
6	157.00	19730.6
7	141.74	17813.0
8	139.64	17549.0
9	137.12	17232.3
10	136.41	17142.8
11	132.19	16612.5
12	130.87	16446.7
13	128.86	16193.4
14	128.63	16164.6
15	128.28	16120.6
16	125.74	15801.5
17	122.04	15336.6
18	115.37	14498.3
19	115.19	14476.2
20	47.25	5937.4
21	44.98	5653.3
22	41.34	5194.8
23	40.26	5059.7
24	19.40	2438.5

Spectrum 44. ^{13}C NMR of compound **123** (125 MHz, DMSO-d_6)

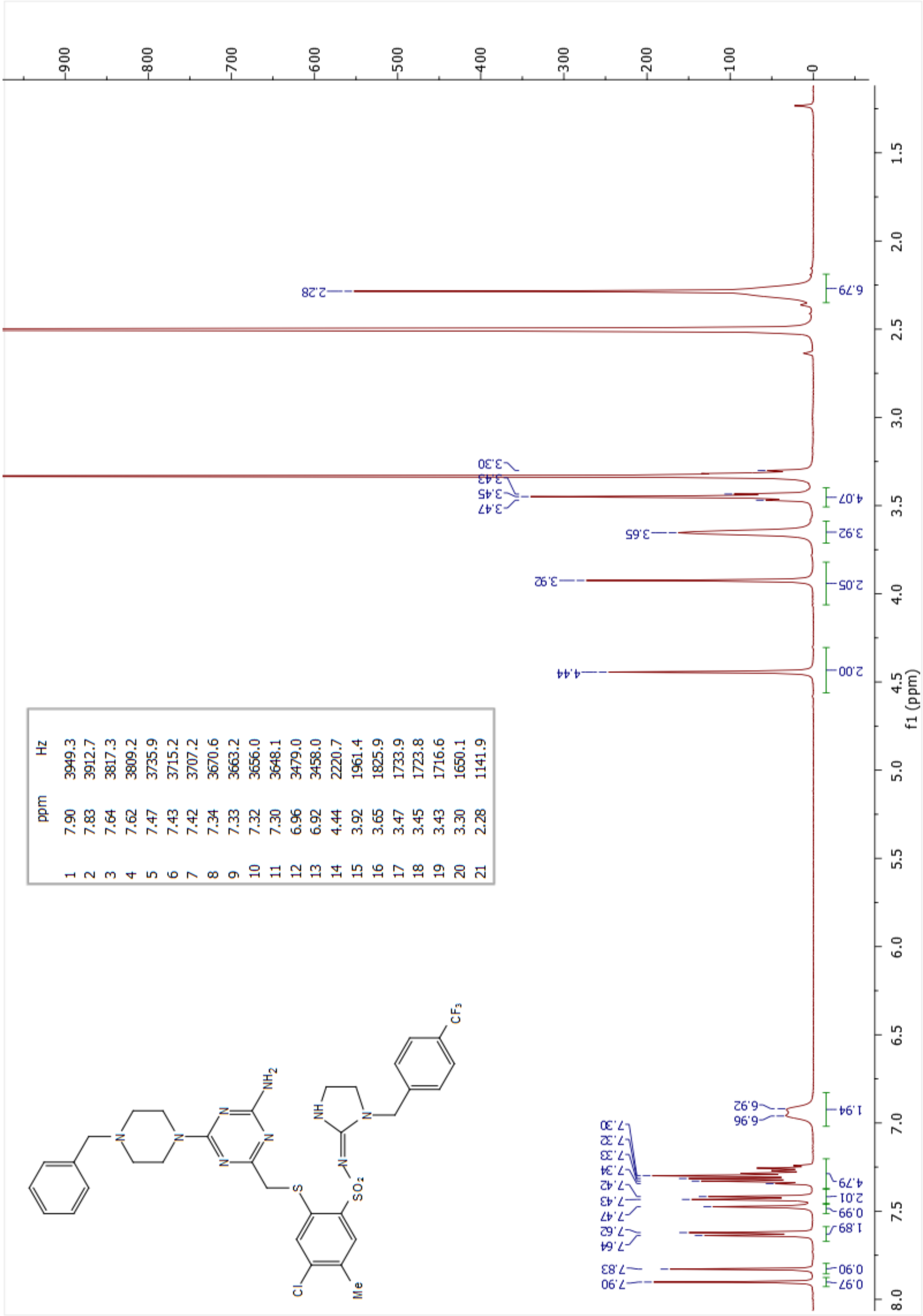

Spectrum 45. ^{1}H NMR of compound **140** (500 MHz, DMSO-d_6 in 60°C)

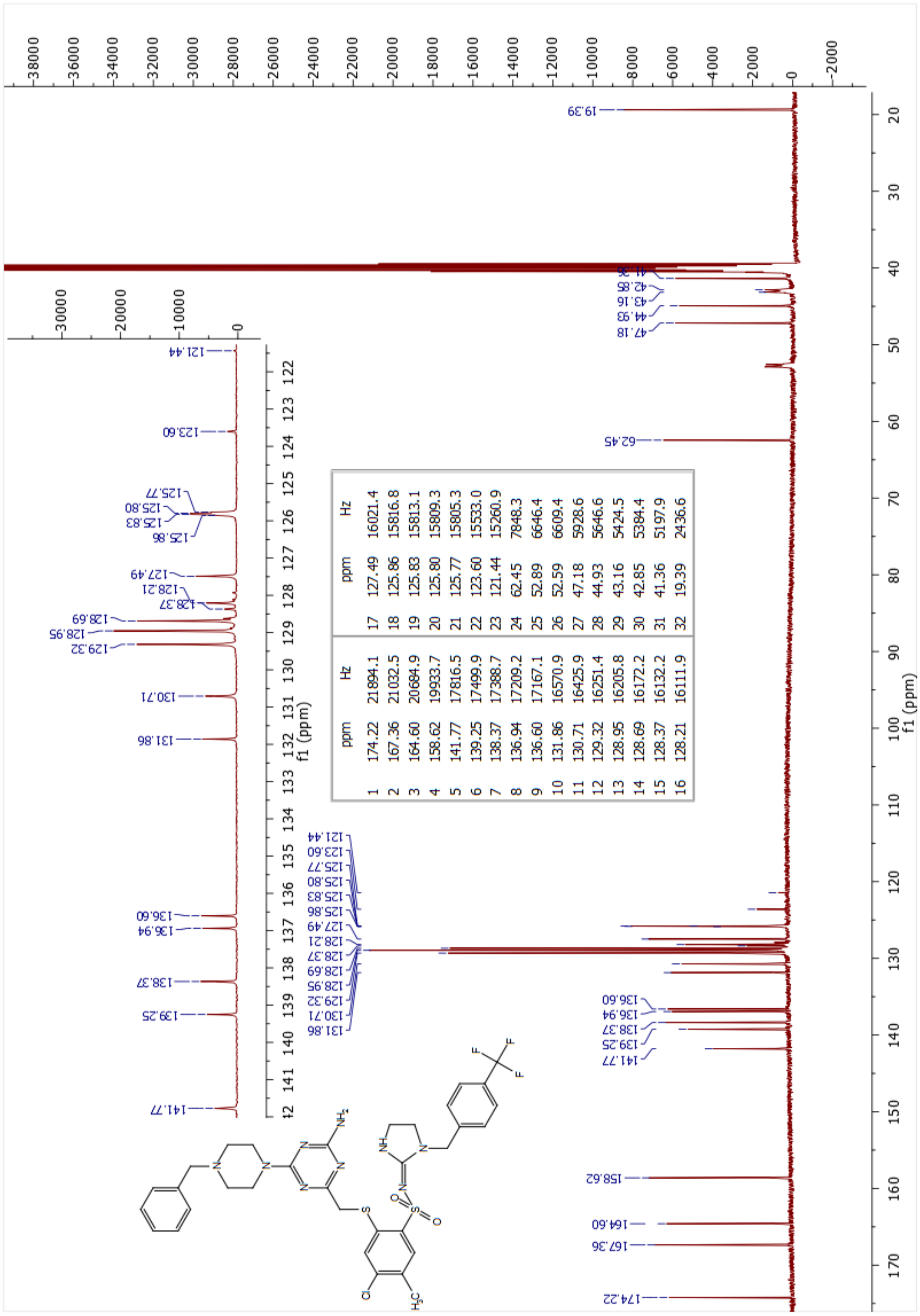

Spectrum 46. ^{13}C NMR of compound **140** (125 MHz, DMSO-d_6)

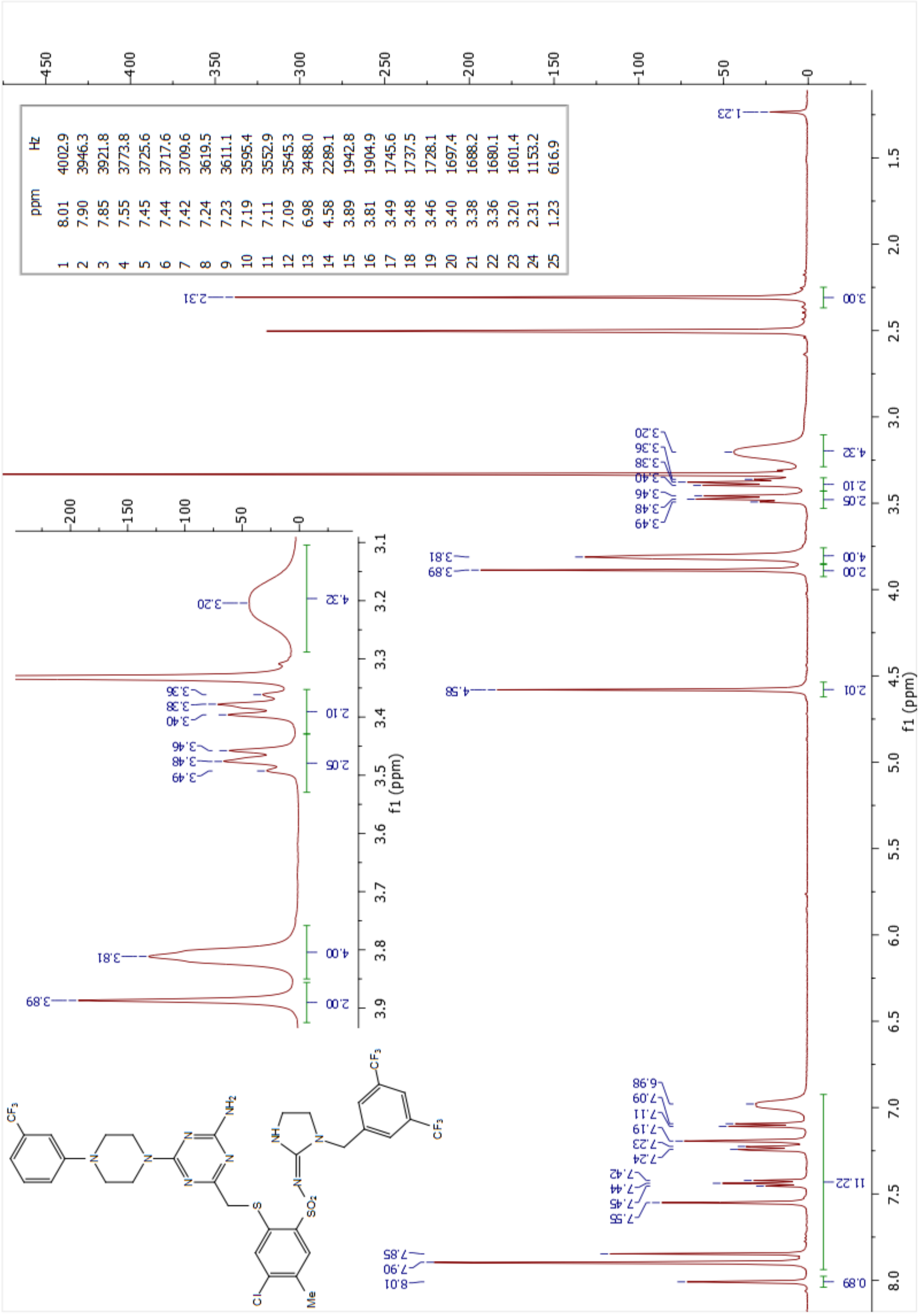

Spectrum 47. ^{1}H NMR of compound **153** (500 MHz, DMSO-d_6)

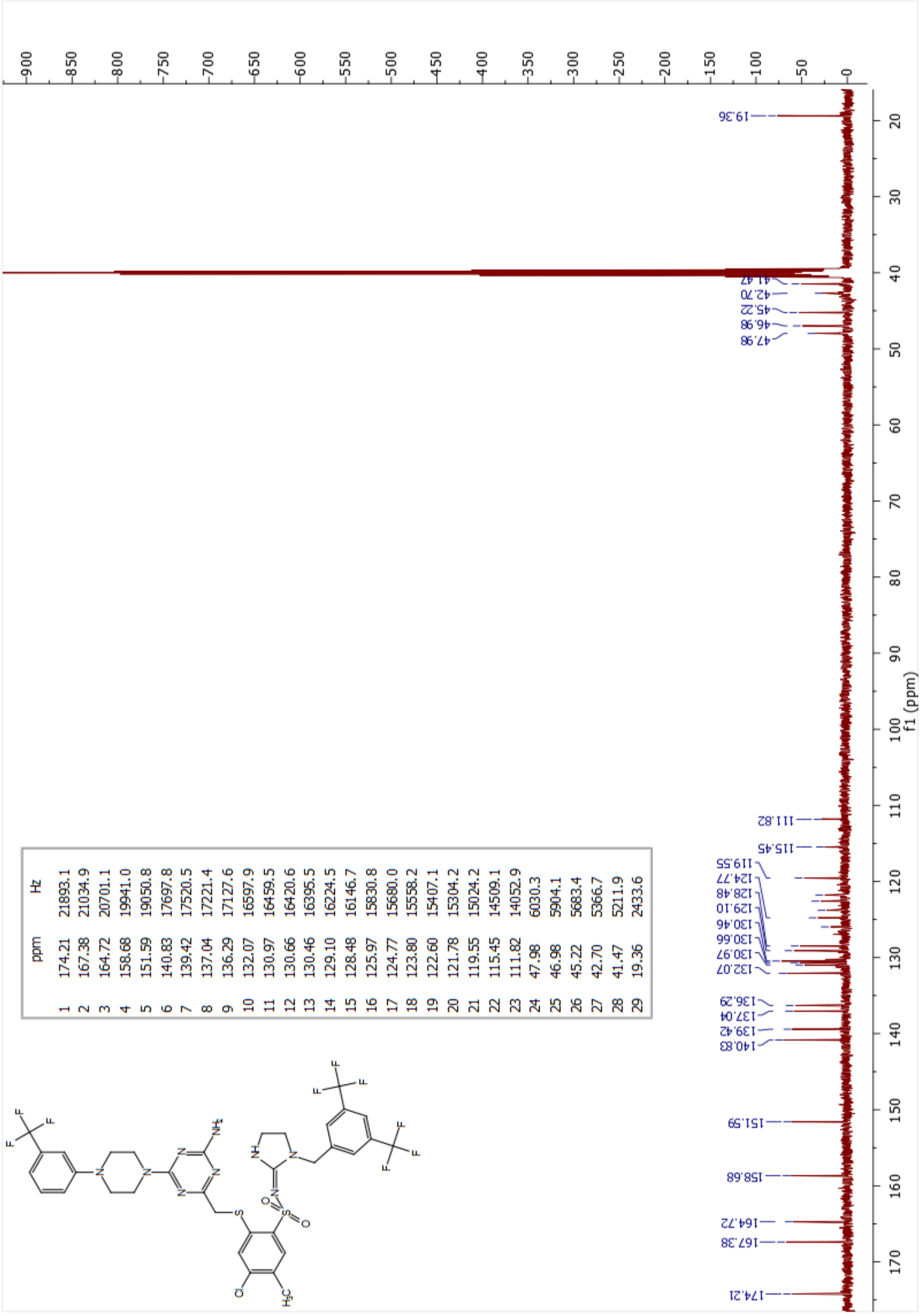

	ppm	Hz
1	174.21	21893.1
2	167.38	21034.9
3	164.72	20701.1
4	158.68	19941.0
5	151.59	19050.8
6	140.83	17697.8
7	139.42	17520.5
8	137.04	17221.4
9	136.29	17127.6
10	132.07	16597.9
11	130.97	16459.5
12	130.66	16420.6
13	130.46	16395.5
14	129.10	16224.5
15	128.48	16146.7
16	125.97	15830.8
17	124.77	15680.0
18	123.80	15558.2
19	122.60	15407.1
20	121.78	15304.2
21	119.55	15024.2
22	115.45	14509.1
23	111.82	14052.9
24	47.98	6030.3
25	46.98	5904.1
26	45.22	5683.4
27	42.70	5366.7
28	41.47	5211.9
29	19.36	2433.6

Spectrum 48. ^{13}C NMR of compound **153** (125 MHz, DMSO-d$_6$)

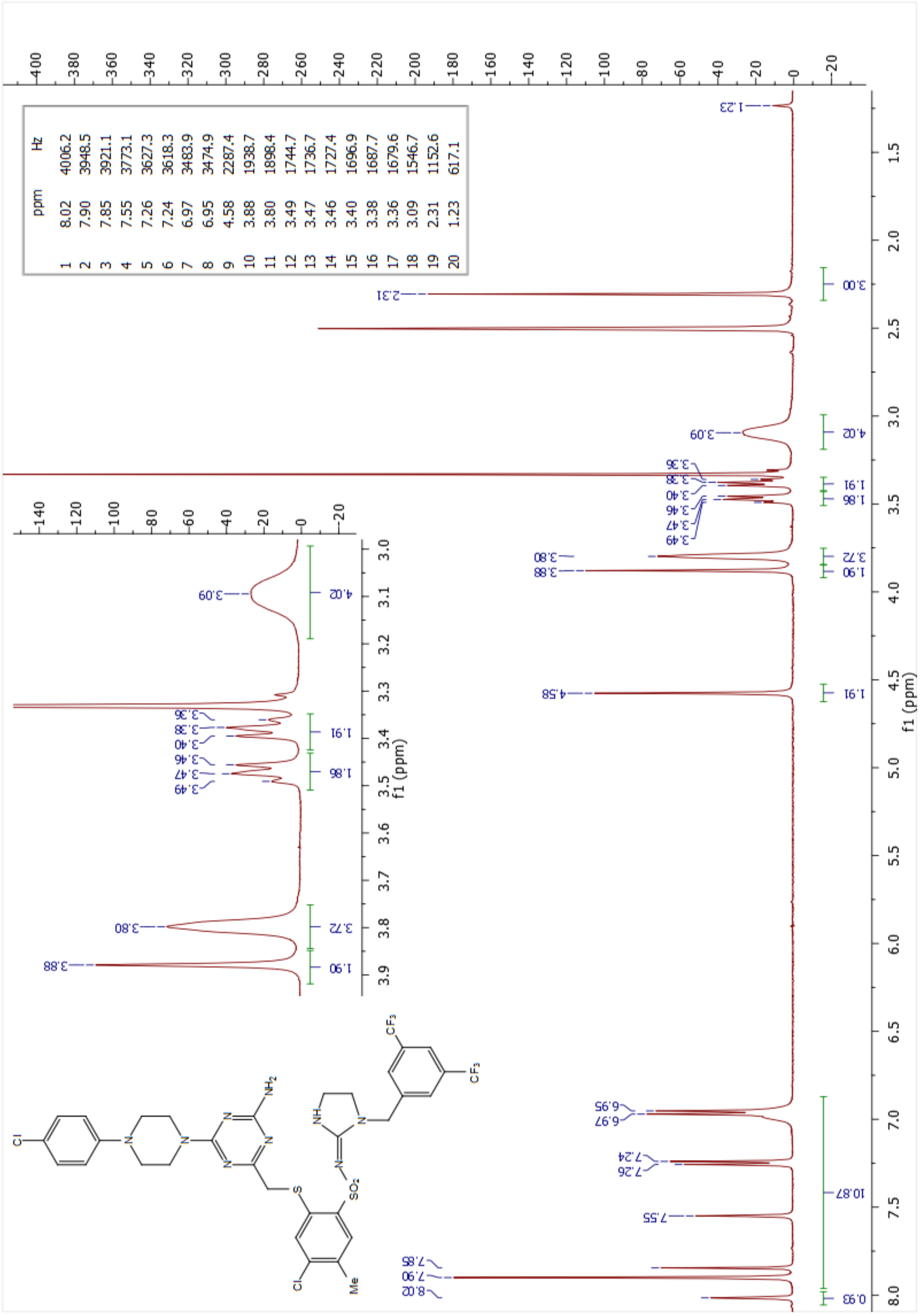

Spectrum 49. ^{1}H NMR of compound **154** (500 MHz, DMSO-d_6)

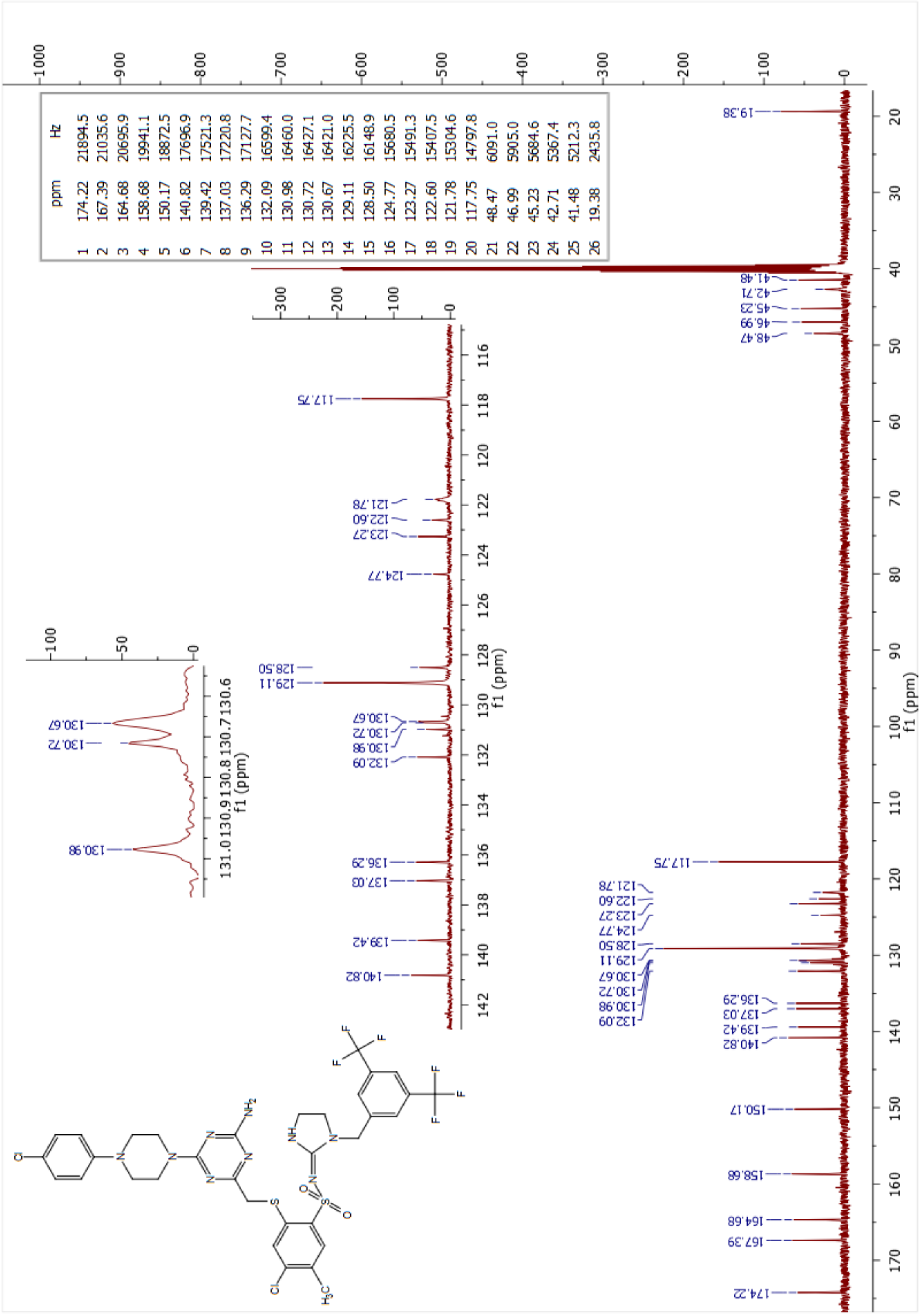

Spectrum 50. ^{13}C NMR of compound **154** (125 MHz, DMSO-d_6)

Crystallographic details (for experimental part)

Diffraction intensity data for **27** were collected on an IPDS 2T dual beam diffractometer (STOE & Cie GmbH, Darmstadt, Germany) at 120.0(2) K with CuKa radiation of a microfocus X-ray source (GeniX 3D Mo High Flux, Xenocs, Sassenage, 50 kV, 0.6 mA and λ = 1.54186 Å). Investigated crystals were thermostated under a nitrogen stream at 120 K using the CryoStream-800 device (Oxford CryoSystem, UK) during the entire experiment.

Data collection and data reduction were controlled by using the X-Area 1.75 program (STOE, 2015). Numerical absorption correction was performed by integration. The structure was solved using intrinsic phasing implemented in SHELXT and refined anisotropically using the program packages Olex2[1] and SHELX-2015[2,3]. Positions of the C–H hydrogen atoms were calculated geometrically taking into account isotropic temperature factors. All H-atoms were refined as riding on their parent atoms with the usual restraints.

Special treatment. Structure **27** was refined with usual procedures.

Crystallographic data for all structures reported in this paper have been deposited with the Cambridge Crystallographic Data Centre as supplementary publication No. CCDC 2154790. Copies of the data can be obtained free of charge on application to CCDC, 12 Union Road, Cambridge CB2 1EZ, UK (Fax: (+44) 1223-336-033; E mail: deposit@ccdc.cam.ac.uk).

***Table* S1.** Crystal data and structure refinement of **27**.

Chemical formula	$C_{21}H_{23}ClN_8O_2S_2·2(C_2H_6OS)$
M_r	675.30
Crystal system, space group	Triclinic, $P\bar{1}$
Temperature (K)	120
a, *b*, *c* (Å)	10.3691 (6), 12.5443 (7), 14.4587 (8)
α, β, γ (°)	102.829 (4), 102.955 (5), 112.543 (4)
V (Å^3)	1590.59 (17)
Z	2
Radiation type	Cu $K\alpha$
μ (mm^{-1})	3.90
Crystal size (mm)	0.11 × 0.09 × 0.04
Data collection:	
Diffractometer	STOE *IPDS* 2T
Absorption correction	Integration<br>STOE *X-RED32*, absorption correction by Gaussian integration, analogous to P. Coppens, "The Evaluation of Absorption and Extinction in Single-Crystal Structure Analysis", published in F. R. Ahmed (Editor), "Crystallographic Computing", Munksgaard, Copenhagen (1970), 255 – 270
T_{min}, T_{max}	0.734, 0.932
No. of measured, independent and observed [$I > 2\sigma(I)$] reflections	13598, 5172, 4530
R_{int}	0.046
$(\sin \theta/\lambda)_{max}$ (Å^{-1})	0.588
Refinement	
$R[F^2 > 2\sigma(F^2)]$, $wR(F^2)$, S	0.093, 0.289, 1.34
No. of reflections	5172
No. of parameters	394
H-atom treatment	H-atom parameters constrained
$\Delta\rangle_{max}$, $\Delta\rangle_{min}$ (e Å^{-3})	0.54, -1.14

Table S2. Hydrogen-bond geometry (Å,°) for **27**.

D—H···*A*	*D*—H	H···*A*	*D*···*A*	*D*—H···*A*
N8—H8*A*···S3^{i}	0.88	2.99	3.591 (3)	127
N8—H8*B*···N4^{i}	0.88	2.11	2.969 (4)	165
N2—H2···O1	0.88	2.30	2.805 (4)	117
N2—H2···O5	0.88	2.20	2.816 (4)	127
N3—H3···N5^{ii}	0.88	2.32	3.016 (4)	136
N7—H7···N1^{ii}	0.88	2.07	2.927 (5)	163
C2—H2*B*···S4^{iii}	0.99	2.77	3.762 (5)	176
C2—H2*B*···O5^{iii}	0.99	2.54	3.441 (6)	151
C6—H6···O4	0.95	2.43	3.362 (5)	166
C11—H11*B*···O4	0.99	2.30	3.270 (5)	165
C22—H22*B*···O2^{iv}	0.98	2.62	3.448 (5)	142

Symmetry codes: (i) -*x*+1, -*y*+1, -*z*+1; (ii) -*x*, -*y*, -*z*+1; (iii) -*x*+1, -*y*, -*z*+1; (iv) *x*, *y*+1, *z*.

References:

23. Pomarnacka, E.; Brzozowski, Z.; Filczewski, M.; Szymanska-Kosmala, M.; Oledzka, K. Various possibilities of using 6-chloro-3-methylthio-7-methyl-1,1-dioxo-1,4,2-benzodithiazine for the synthesis of 2-(4-chloro-2-mercapto-5-methylbenzenesulfonamido)imidazoline derivatives, and their pharmacological properties. *Acta Pol. Pharm.* **1985**, *42*, 501–510.
24. Pomarnacka, E.; Kornicka, A. Synthesis and *in vitro* anticancer and anti-HIV evaluation of new 2-mercaptobenzenesulfonamides. *Farmaco II* **2001**, *56*, 571–577.
31. Kozakiewicz, I. Syntezy niektórych pochodnych 2-[4-chloro-5-metylo-2-(2,4-diamino-1,3,5-triazyn-6-ylo)metylotiobenzenosulfonylo]iminoimidazolidyny. *Ann. Acad. Med. Gedan.* **1998**, 28, 155–159.
42. Brzozowski, Z.; Sławiński, J. 1,1-Dioxo-1,4,2-benzodithiazine derivatives. II Synthesis of some 3-mercapto-1,1-dioxo-1,4,2-benzodithiazine derivatives. *Acta Pol. Pharm.* **1984**, *41*, 133–139.
